# Cascade Cross‐Coupling of Dienes: Photoredox and Nickel Dual Catalysis

**DOI:** 10.1002/anie.201911109

**Published:** 2019-11-28

**Authors:** Long Huang, Chen Zhu, Liang Yi, Huifeng Yue, Rajesh Kancherla, Magnus Rueping

**Affiliations:** ^1^ Institute of Organic Chemistry RWTH Aachen University Landoltweg 1 52074 Aachen Germany; ^2^ KAUST Catalysis Center (KCC) King Abdullah University of Science and Technology (KAUST) Thuwal 23955-6900 Saudi Arabia

**Keywords:** cross-coupling, dienes, nickel, photocatalysis, radical reactions

## Abstract

Chemical transformations based on cascade reactions have the potential to simplify the preparation of diverse and architecturally complex molecules dramatically. Herein, we disclose an unprecedented and efficient method for the cross‐coupling of radical precursors, dienes, and electrophilic coupling partners via a photoredox‐ and nickel‐enabled cascade cross‐coupling process. The cascade reaction furnishes a diverse array of saturated carbo‐ and heterocyclic scaffolds, thus providing access to a quick gain in C−C bond saturation.

## Graphical abstract

**Increasing molecular complexity**: Radical precursors, dienes, and electrophilic coupling partners are transformed in a photoredox‐ and nickel‐enabled radical addition/cyclization/cross‐coupling process. The reaction takes place under mild conditions and furnishes a diverse array of saturated carbo‐ and heterocyclic scaffolds, thus providing a quick gain in C−C bond saturation.

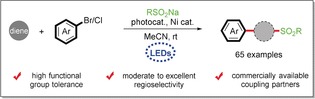

## Introduction

Among the many different approaches to describe molecular complexity, C−C bond saturation index (Fsp^3^) has recently been recognized as a key descriptor, owing to the good correlation between clinical success and increasing saturation content.^1^ Hence, there has been an ever‐increasing demand for the large and rapid increase of molecular complexity, enriched with saturated content, from simple and readily available feedstock chemicals in the field of medicinal chemistry.^2^ An appealing method to meet this demand is based on free‐radical cascade cyclizations, which are powerful and versatile methods for the construction of carbo‐ and heterocyclic ring systems found in drug molecules and natural products.^3^

In the past several years, visible light photocatalysis and nickel dual catalysis has emerged as a powerful tool in organic synthesis. In this context, the trapping of open‐shell species (carbon‐, nitrogen‐, sulfur‐, and phosphorus‐based radicals) by various approaches has been thoroughly explored.^4^ However, despite the previous success of cascade radical cyclizations in radical chemistry, their application in photoredox‐enabled cross‐coupling reactions has not previously been exploited until very recently.4s On the other hand, the coupling of conventional preformed organometallics (Mg, Zn, and B) with aryl halide electrophiles has been reported in the field of cascade cyclization/cross‐coupling.^5^ However, the requirement of activated structures leads to low efficiency as well as poor step and atom economies. Sulfone functional groups are embedded in a large number of pharmaceuticals, agrochemicals, and functional materials;^6^ meanwhile the sulfone group is recognized as a versatile building block in a variety of carbon–carbon bond‐forming reactions, such as fragment coupling and Julia olefination.^7^ In our continuing efforts to expand the area of visible light photocatalysis, we focus also on the development of efficient and practical methods for the synthesis of diverse sulfones under milder reaction conditions. Herein, we demonstrate that a dual photoredox/nickel^4^, ^8^‐enabled cascade cross‐coupling can forge two new C−C bonds and one C−S^9^ bond using simple radical precursors, dienes, and electrophilic coupling partners. This transformation generates three bonds in one synthetic step and allows a rapid increase of molecular complexity with respect to Fsp^3^ from simple and commercially available feedstock chemicals. Due to the mild nature of reaction conditions employed, a variety of functional groups are well‐tolerated making transition‐metal‐catalyzed reactions invaluable in the context of complex molecule synthesis.

**Scheme 1:**
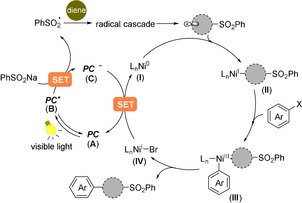
Our proposed radical cyclization/cross‐coupling cascade reaction enabled by photoredox/nickel dual catalysis.

Our proposed mechanism is shown in Scheme 1. Upon visible light irradiation, single electron transfer (SET) from the sodium sulfinate salt (for PhSO_2_^.^/PhSO_2_Na, *E*_1/2_^red^= +0.5 V vs. SCE in MeCN)^10^ to the highly oxidizing excited state **B** of the photocatalyst (PC) would generate the sulfonyl free radical (PhSO_2_^.^) along with the reduced form **C** of the photocatalyst. The S‐centered radical could add to a diene, followed by a radical cascade cyclization to deliver a C‐centered radical, which would be then intercepted by Ni^0^ species **I** to yield an alkyl–Ni^I^ intermediate **II**. Subsequent oxidative addition of an aryl halide Ar–X would form the Ni^III^ complex **III**, which yields the desired coupled product as well as Ni^I^ intermediate **IV** via reductive elimination. Lastly, the nickel and photoredox catalytic cycles end up simultaneously, via a single‐electron‐transfer event between Ni^I^ intermediate and the reduced form **C** of the photocatalyst.

## Results and Discussion

The optimization of the reaction conditions of the cascade cross‐coupling is briefly summarized in Table 1. The initial evaluation of the proposed coupling focused on the reaction between commercially available diethyl diallylmalonate (**1**), methyl 4‐bromobenzoate (**2 a**), and sodium benzenesulfinate (**3**). The optimized reaction conditions were established using 1 mol % [Ir(dtbpy)(bpy)_2_]PF_6_ (**PC‐I**, *E*_1/2_[Ir^*III^/Ir^II^ = +0.66 V vs. SCE in CH_3_CN]),^11^ 10 mol % NiCl_2_⋅6 H_2_O, and 15 mol % 4,4′‐di‐*tert*‐butyl‐2,2′‐bipyridine (dtbbpy) in a 0.1 m solution of acetonitrile at room temperature with blue LEDs irradiation for 20 h. Under these conditions, the desired product was formed in 91 % NMR yield with good regioselectivity (dr 88:12 with *cis* as major isomer) (Table 1, entry 1). Reaction with other photosensitizers such as [Ir(dF(CF_3_)ppy)_2_(dtbpy)]PF_6_ (**PC‐II**, *E*_1/2_[Ir^*III^/Ir^II^ =+ 1.21 V vs. SCE in CH_3_CN]),^12^ and organic dye **PC‐V** (*E*_1/2_[^*^PC/PC^−^=+1.35 V vs. SCE in MeCN])8a gave comparable yield; however, the yield decreased dramatically when the less oxidizing Ru‐based photocatalysts **PC‐III** and **PC‐IV** were employed (Table 1, entries 2–5). Among the various bi‐ and tridentate ligands evaluated, only the pyridine‐derived ligand class delivered substantial amounts of product **4** (Table 1, entries 6–10). As anticipated, control experiments performed without photocatalyst, Ni catalyst, light, or degassing all delivered no product (Table 1, entries 11–14).

**Table 1:** Optimization of the reaction conditions.^[a]^ 
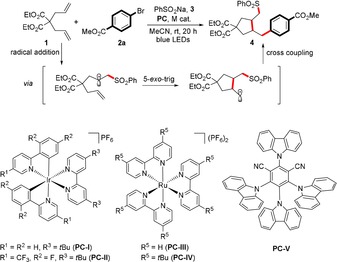

Entry	PC	M cat. (mol %)	Ligand (mol %)	Yield [%]^[b]^
1	**PC‐I**	NiCl_2_⋅6 H_2_O (10)	**L1** (15)	91
2	**PC‐II**	NiCl_2_⋅6 H_2_O (10)	**L1** (15)	88
3	**PC‐III**	NiCl_2_⋅6 H_2_O (10)	**L1** (15)	20
4	**PC‐IV**	NiCl_2_⋅6 H_2_O (10)	**L1** (15)	25
5^[c]^	**PC‐V**	NiCl_2_⋅6 H_2_O (10)	**L1** (15)	89
6	**PC‐I**	NiCl_2_⋅6 H_2_O (10)	**L2** (15)	89
7	**PC‐I**	NiCl_2_⋅6 H_2_O (10)	**L3** (15)	–
8	**PC‐I**	NiCl_2_⋅6 H_2_O (10)	**L4** (15)	45
9	**PC‐I**	NiCl_2_⋅6 H_2_O (10)	**L5** (15)	–
10	**PC‐I**	NiCl_2_⋅6 H_2_O (10)	**L6** (15)	trace
11	**PC‐I**	–	dtbpy (15)	–
12	–	NiCl_2_⋅6 H_2_O (10)	dtbpy (15)	–
13^[d]^	**PC‐I**	NiCl_2_⋅6 H_2_O (10)	dtbpy (15)	–
14^[e]^	**PC‐I**	NiCl_2_⋅6 H_2_O (10)	dtbpy (15)	–
15	–	Pd(OAc)_2_ (5)	XPhos (10)	–
				
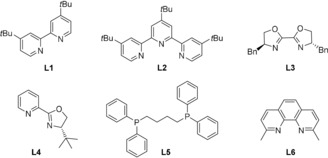				

[a] Reaction conditions: **1** (0.1 mmol, 1 equiv), **2 a** (2 equiv), **3** (2 equiv), [M] (10 mol %), PC (1 mol %), ligand (15 mol %), MeCN (1.0 mL, 0.1 m), rt, blue LEDs, 20 h. [b] Yields were determined by ^1^H NMR using 1,3,5‐trimethoxybenzene as an internal standard. [c] 2.5 mol % photocatalyst was used. [d] No light. [e] No degassing.

With the optimized reaction conditions in hand, we first examined the scope with respect to the diene component (Table 2). The transformation was tolerant to a wide range of electronically unbiased 1,6‐dienes, with ester, ketone, and acetal functional groups being amenable to this coupling protocol (**5**–**10**, 79–98 % yield). Heteroatom‐containing dienes such as diallyl amine and diallyl ether could be utilized, affording the corresponding highly functionalized pyrrolidine and tetrahydrofuran derivatives in similar efficiency albeit with lower diastereoselectivity (**11** and **12**, both 94 % yield). In these cases, a good preference for the *cis*‐3,4‐disubstituted isomers was observed and no 6‐*endo* products were detected. The diastereoselectivities are consistent with previous reports on the stereochemistry of radical cyclizations, suggesting that the generation of the carbon‐centered radical is independent of the nickel catalytic cycle. According to Baldwin's rules, we only observed the product resulting from the six‐membered‐ring cyclization in the reaction of diallyldiphenylsilane. Several considerations such as bond lengths, Si electronic effects, and conformation of the transition states were proposed to explain the thermodynamically more favorable 6‐*endo*‐*trig* cyclization.^13^ Moreover, the reaction is not limited to 1,6‐dienes. For example, selective 1,4‐difunctionalization took place in the case of conjugated 2,3‐dimethyl‐1,3‐butadiene, giving rise to a highly substituted allylsulfone (**14**, 64 %). 2,5‐Norbornadiene was transformed into a disubstituted tricyclic phenyl sulfone; this outcome resulted from the rapid interconversion between the norbornenyl and nortricyclyl radicals, with the nortricyclyl radical being favored, and is also in good agreement with earlier work on norbornadiene radical chemistry.^14^ Similarly, the use of 1,5‐cyclooctadiene allowed an impressive intramolecular cyclization prior to the cross‐coupling, leading to the formation of bicyclic product **15** with excellent stereoselectivity (60 %).

**Table 2:** Scope of the dienes.^[a–c]^

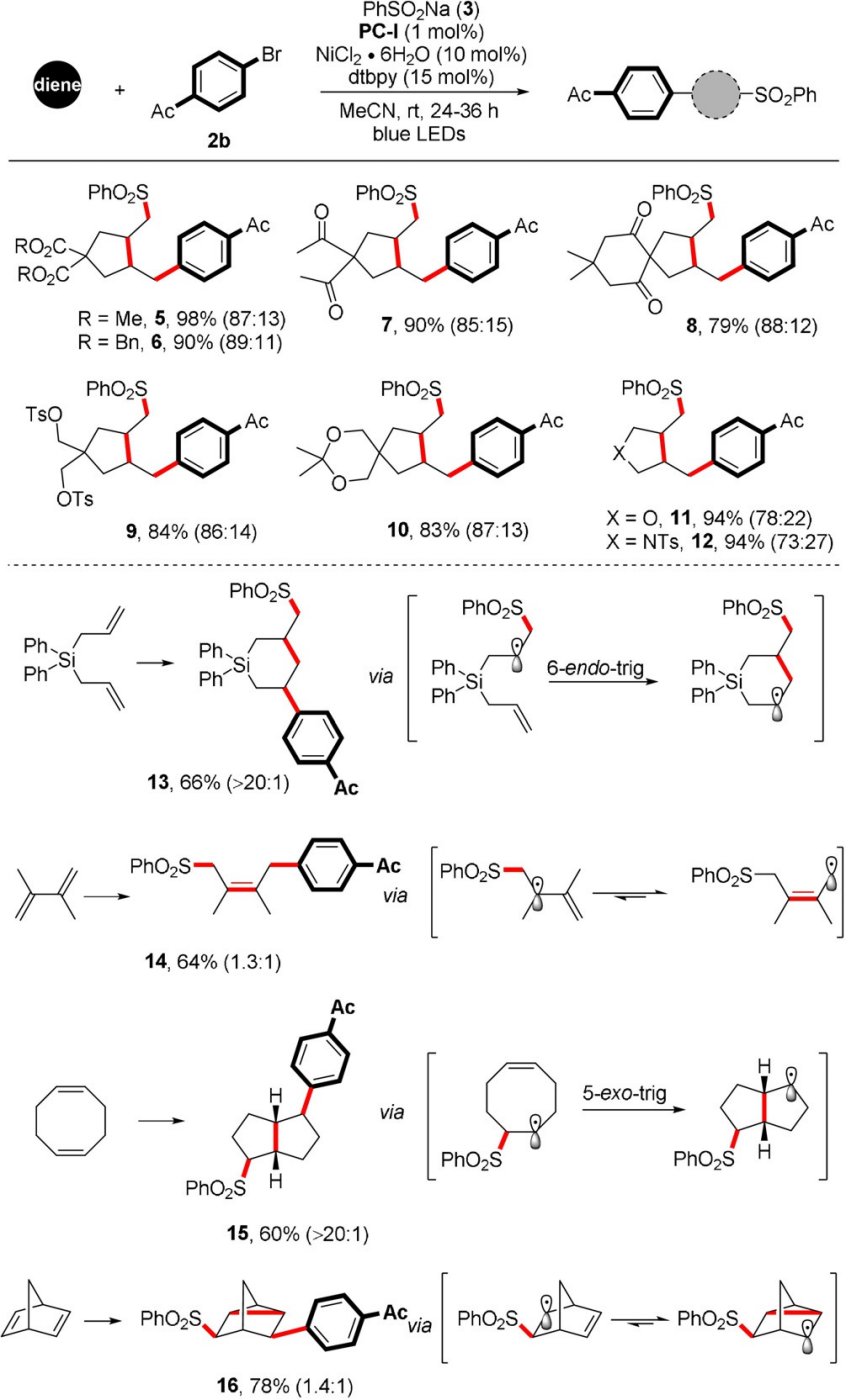

[a] Standard conditions: diene (0.4 mmol, 2 equiv), **2 b** (0.2 mmol), **3** (0.4 mmol, 2 equiv), NiCl_2_⋅6 H_2_O (10 mol %), **PC‐I** (1 mol %), dtbpy (15 mol %), MeCN (2.0 mL, 0.1 m), rt, blue LEDs, 24–36 h. [b] Yield after purification. [c] The regioselectivity was determined by NMR analysis (see the Supporting Information).

Next, we examined the generality of the multicomponent cross‐coupling with regard to the electrophilic coupling partner of this new reaction. As shown in Table 3, a variety of (hetero)aryl halides performed well in this cross‐coupling protocol. For example, electron‐deficient bromoarenes bearing ester, ketone, nitrile, aldehyde, trifluoromethyl, and fluoride were well tolerated (**17**–**22** and **4**, 80–99 % yield). Due to the relatively enhanced rate of oxidative addition of aryl bromide over aryl chloride, the cross‐coupling took place chemoselectively to allow the chlorine group to be retained with opportunity for further synthetic elaboration (**23**, 92 % yield). In this context, it is also noteworthy that a pinacolborate group can also be incorporated onto the arene ring (**24**, 97 % yield). Next, several electron‐neutral and electron‐rich arenes containing alkyl, aryl, as well as amino functionalities were employed and gave the corresponding products in excellent yields (**25**–**29**, 81–97 % yield). Moreover, substituents at the *meta* and *ortho* positions of the aromatic ring had no apparent effect on the efficiency of the coupling (**30**–**34**, 63–98 % yield). Polycyclic aromatic bromides derived from phthalimide, indanone, and naphthalene also served as effective coupling partners, resulting in excellent yields (**35**–**37**, 86–97 % yield). Heteroaromatic halides, which are common scaffolds in the preparation of medicinally relevant targets, such as pyridine, pyrimidine, quinoline, indole, and thiophene are all effective electrophiles in this protocol (**38**–**44**, 70–93 % yield). In addition, we were delighted to find that the current coupling can be further extended to the more abundant and diverse aryl chlorides. This is remarkable considering that the use of aryl chlorides in Ni‐catalyzed cross‐couplings is difficult and rather underdeveloped. A range of aryl chlorides underwent the cascade cross‐coupling to form the corresponding products with good efficiency (**4**, **17**, **18**, and **45**–**47**, 46–90 % yield). Finally, we demonstrated the value of this new method for medicinal chemistry by the rapid incorporation of C−C bond saturated ring systems to biologically active molecules. Pharmaceutically relevant aryl halides including fenofibrate, indomethacin, and cholesterol derivatives were all coupled with good efficiencies (**48**–**50**, 57–85 % yield).

**Table 3:** Scope of the aryl halides.^[a,b]^

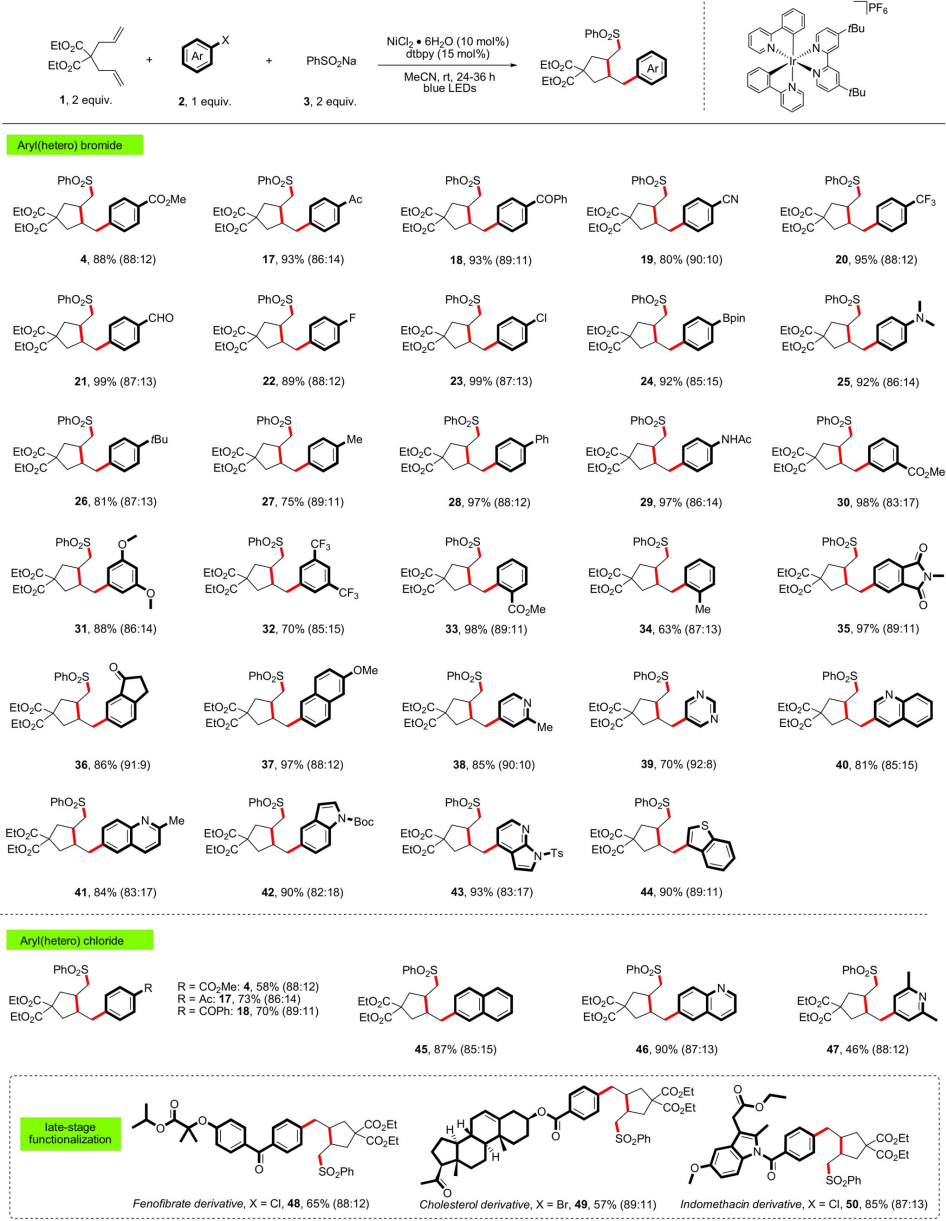

[a] Standard conditions: **1** (0.4 mmol, 2 equiv), (hetero)aryl halide **2** (0.2 mmol), **3** (0.4 mmol, 2 equiv), NiCl_2_⋅6 H_2_O (10 mol %), **PC‐I** (1 mol %), dtbpy (15 mol %), MeCN (2.0 mL, 0.1 m), rt, blue LEDs, 24–36 h; yield after purification. [b] The regioselectivity was determined by NMR analysis (see the Supporting Information). Boc=*tert*‐butyloxycarbonyl; Ts=toluenesulfonyl; Ac=acetyl.

With the above success, we next investigated a diverse set of either commercially available or readily accessible sulfinate salts to further highlight the versatility of this method. As indicated in Table 4, a wide variety of neutral, electron‐rich, and electron‐poor benzene sulfinates were compatible with the optimized conditions (**51**–**57**, 60–98 % yield). Although the lithium sulfinate is anionic, the use of additional 2 equiv Na_2_CO_3_ was essential to achieve an efficient reaction under the standard conditions (**5**, 92 % yield). In the cases of linear and cycloalkyl sodium sulfinates, the sulfonyl group was surprisingly preserved in contrast to the recent reports on desulfinative cross coupling with photoredox/Ni dual catalysis (**58**–**60**, 74–98 % yield).^15^ Moreover, heterocyclic derivatives such as pyridine‐3‐sulfinates and thiophene‐3‐sulfinates were all effective coupling partners in this protocol (**61** and **62**, 71 and 92 % yield, respectively).

**Table 4:** Scope of the sulfinate salts.^[a–c]^

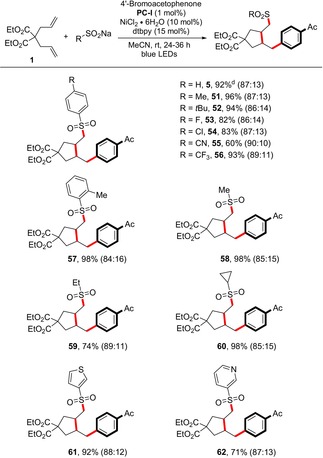

[a] Standard conditions: **1** (0.4 mmol, 2 equiv), **2** (0.2 mmol), sulfinate salt (0.4 mmol, 2 equiv), NiCl_2_⋅6 H_2_O (10 mol %), **PC‐I** (1 mol %), dtbpy (15 mol %), MeCN (2.0 mL, 0.1 m), rt, blue LEDs, 24–36 h [b] Yield after purification. [c] Regioselectivity was determined by NMR analysis (see the Supporting Information). [d] Lithium salt was used together with 2 equiv of Na_2_CO_3_.

**Scheme 2:**
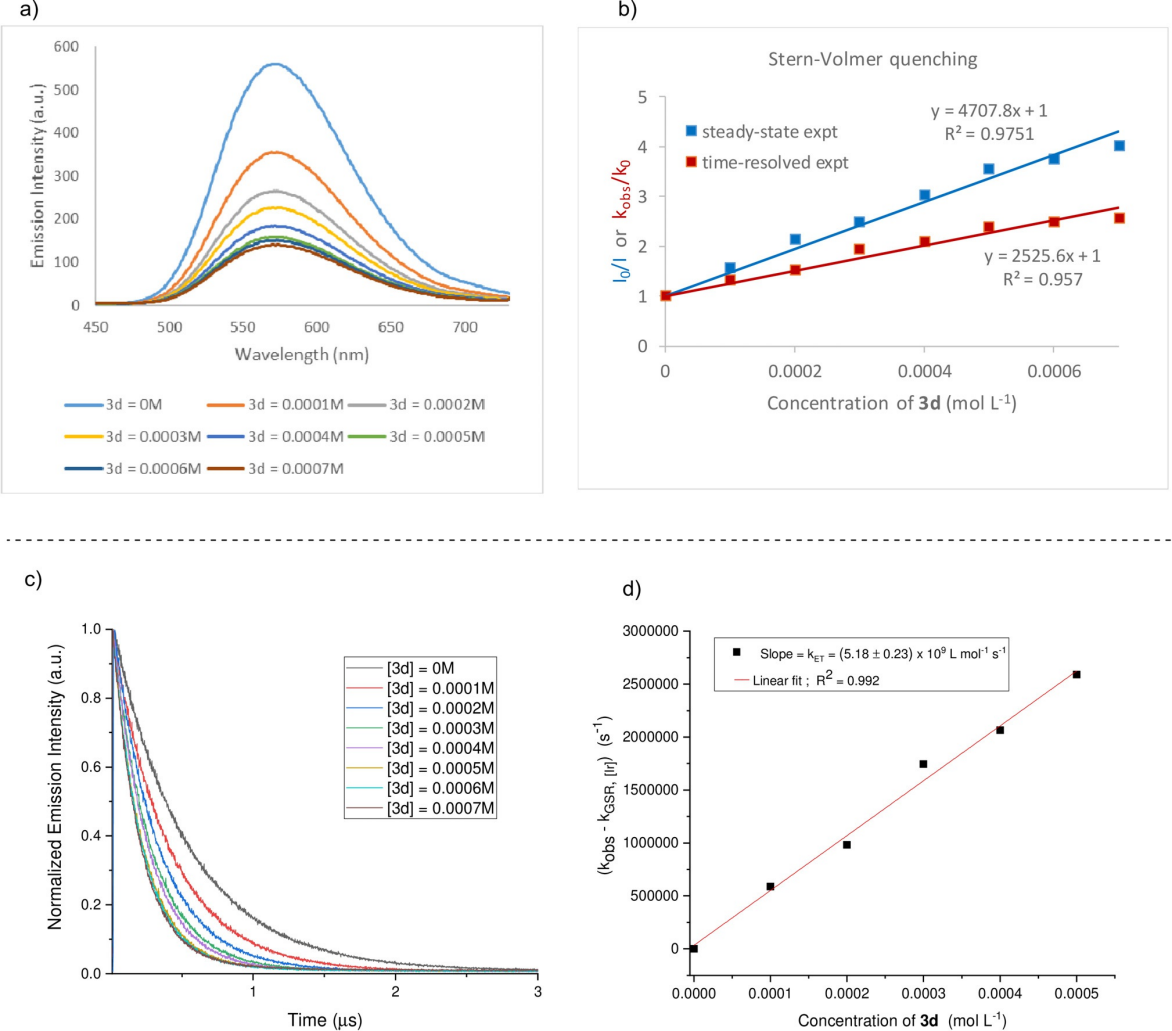
a) Steady‐state Stern–Volmer experiment of **PC‐I** [Ir(dtbpy)(bpy)_2_]PF_6_ and sodium 4‐cyano phenylsulfinate (**3 d**). b) Combined quenching data of steady‐state and time‐resolved studies. c) Phosphorescence lifetimes of *PC‐I (0.00001 m) at different concentrations of quencher **3 d**. d) Stern–Volmer analysis yielded a rate constant, *k*_ET_ of (5.18±0.23)×10^9^ L mol^−1^ s^−1^ by the SET between *PC‐I and **3 d**.

**Scheme 3:**
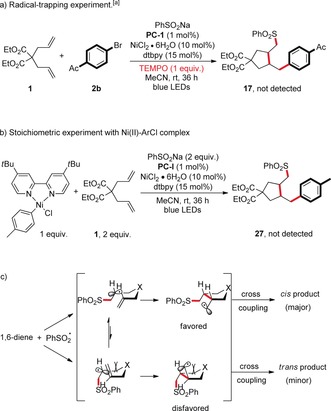
Preliminary experiments on the reaction mechanism. a) Reaction inhibition with a radical scavenger. b) Stoichiometric study with Ni^II^–ArCl complex. c) Origin of the diastereoselectivity in C−C bond formation with 1,6‐diene.

In order to gain more insight into the mechanism of the metalla‐photoredox three‐component‐coupling protocol, we conducted some preliminary mechanistic experiments (Scheme 2). Firstly, we performed cyclic voltammetry analysis with various aryl sulfinates. In comparison with the applied photocatalyst **PC‐I** [Ir(dtbpy)(bpy)_2_]PF_6_ (*E*_1/2_[Ir^*III^/Ir^II^ =+0.66 V vs. SCE in CH_3_CN]), the SET oxidation is thermodynamically feasible regardless of the electronic nature of the substituent on the aryl ring of sulfinate salt. The electron transfer step is promoted by the Ir^III^ photocatalyst **PC‐I**, which can be present as a long‐lived triplet excited state *PC‐I (*τ*_0_=535.17±1.54 ns, Figure S4) that can activate sodium 4‐cyanophenylsulfinate (**3 d**). To conform the quenching of the ^3*^ **PC‐I** by **3 d**, steady‐state Stern–Volmer luminescence quenching experiments were carried out by the addition of different concentrations of **3 d** to ^3*^ **PC‐I** which displayed a linear correlation (Scheme 2 a,b). The quenching study by time‐resolved emission spectroscopy also revealed a similar linear correlation where the excited‐state lifetime of ^3*^ **PC‐I** is quenched by the different concentrations of **3 d** (Scheme 2 b,c). Such a linear correlation in both steady‐state and time‐resolved experiments demonstrates that the quenching of excited‐state ^3*^ **PC‐I** by **3 d** is dynamic in nature and further confirms that there is no ground‐state association between the photocatalyst and **3 d** in solution. To shed more light on the underlying electron transfer event, the electron transfer (ET) rate constant *k*_ET_ was determined by time‐resolved emission measurements where the slope correlates with the ET rate constant *k*_ET_. A *k*_ET_ of (5.18±0.23)×10^9^ L mol^−1^ s^−1^ was determined with a good linear fit by plotting the difference between the observed rate constant (*k*_obs_) and the ground‐state recovery rate (*k*_GSR_) versus different concentrations of **3 d** (Scheme 2 d). When the reaction mixture of diene **1**, aryl bromide **2 b**, and PhSO_2_Na was treated under the standard conditions in the presence of 2,2,6,6‐tetramethyl‐1‐piperidinyloxy (TEMPO, 1 equiv) radical scavenger, no product was detected, implying that a radical process is involved in the catalytic cycle (Scheme 3 a). Importantly, our stoichiometric study with Ni^II^–ArCl (Scheme 3 b) failed to give the corresponding cross‐coupling product. This result indicates that a Ni^II^–aryl species is not involved in the catalytic cycle, making the alternative catalytic Ni^0^/Ni^II^/Ni^III^ pathway rather unlikely. As a result, our proposed mechanism involving first radical capture by Ni^0^ followed by oxidative addition with aryl halides is more operative. The stereochemical outcome of the cyclization of sulfonyl‐substituted 5‐hexenyl radicals can be rationalized by using the chair‐like transition state based on literature reports (Scheme 3 c).^16^ We assume that the *cis*‐favored selectivity is partly due to the steric effect of X substituent. This consideration was supported by the fact that diallyl malonate (Table 2, **5**) gave higher *cis*/*trans* ratio than the O‐tethered diene (Table 2, **11**).

**Scheme 4:**
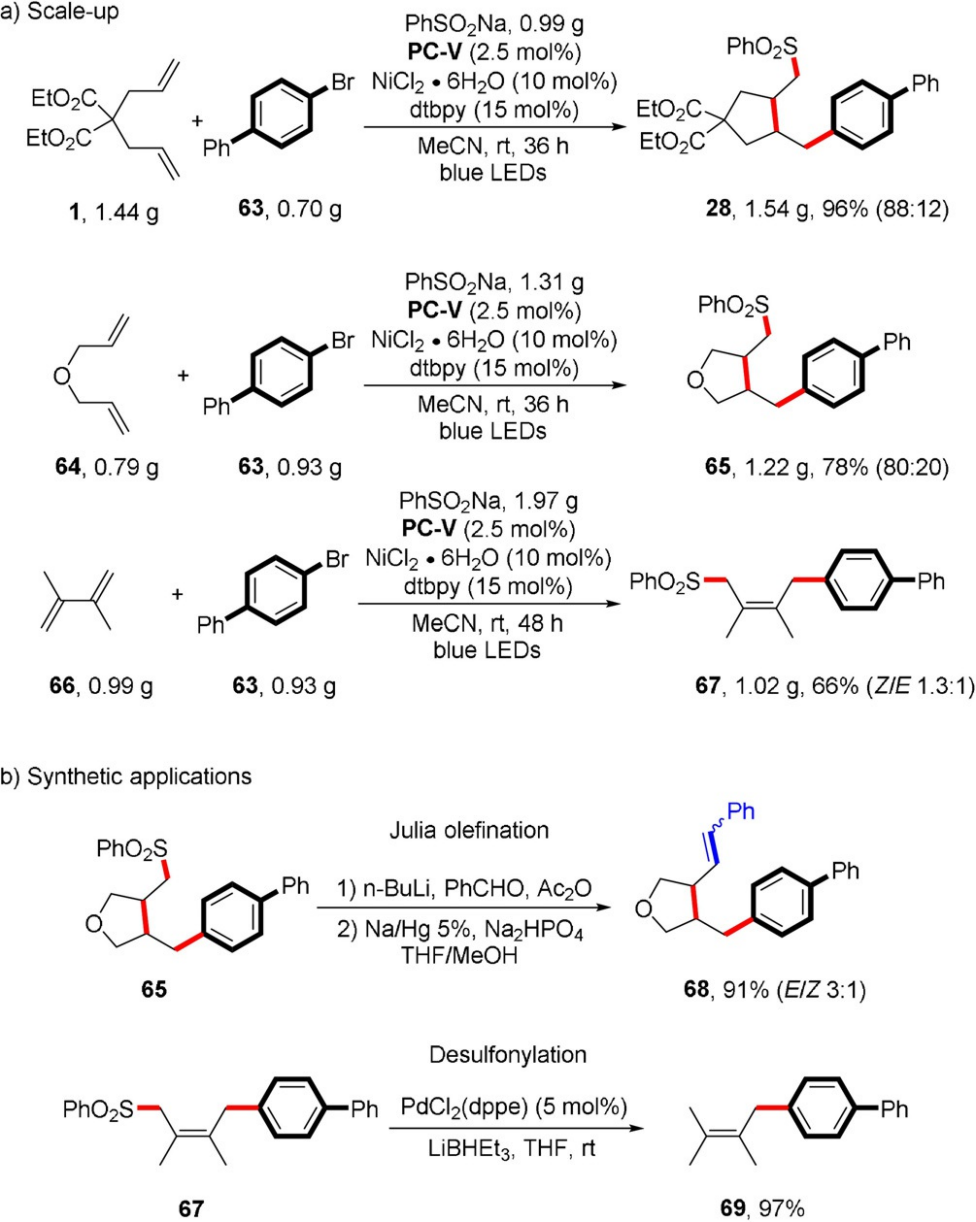
a) Reaction on large scale, under standard conditions: **63** (3 or 4 mmol, 1 equiv), diene (2 or 3 equiv), sulfinate salt (2 or 3 equiv), NiCl_2_⋅6 H_2_O (10 mol %), **PC‐V** (2.5 mol %), dtbpy (15 mol %), MeCN (0.1 m), rt, blue LEDs, 36 or 48 h. b) Synthetic transformations of sulfones **65** and **67**, see the Supporting Information for details.

To highlight the robustness of the reaction, we carried out the synthesis of a sulfone on a preparative scale (3 or 4 mmol). Due to the higher cost of the iridium‐based photocatalyst, we sought to use alternative sustainable organic photosensitizers. When we replaced iridium catalyst **PC‐I** with carbazolyl dicyanobenzene **PC‐V** under our optimal conditions, we obtained the expected sulfonylarylation products **28**, **65**, and **67** in comparable good efficiency (Scheme 4 a). To further demonstrate the synthetic utility of the present coupling method, sulfone **65** subjected to Julia olefination conditions to provide the *E* alkene **68** in good yield (Scheme 4 b).^17^ Moreover, the allylic sulfone **67** could be desulfonylated to afford the allylarene **69** by Pd‐catalyzed desulfonylation with LiEt_3_BH as reductant.^18^

## Conclusion

In conclusion, we have developed an unprecedented cascade cyclization/cross‐coupling of various dienes with substituted sulfinates and aryl(hetero) halides via the photoredox and nickel synergistic catalysis. The manifold forges three new bonds (one C−S and two C−C bonds) in one synthetic step and allows rapid increase of molecular complexity with respect to Fsp^3^ from simple and commercially available feedstock chemicals. A variety of carbo‐ and heterocyclic cores that are privileged motifs in pharmaceuticals, bioactive molecules, and natural products can be accessed with moderate to excellent selectivities. We anticipate that these attributes will lead to brisk exploitation in the field of radical cascade/cross‐coupling. In addition we performed a series of mechanistic investigations which supported the proposed radical cyclization cross‐coupling reaction pathway.

## Conflict of interest

The authors declare no conflict of interest.

## Supporting information

As a service to our authors and readers, this journal provides supporting information supplied by the authors. Such materials are peer reviewed and may be re‐organized for online delivery, but are not copy‐edited or typeset. Technical support issues arising from supporting information (other than missing files) should be addressed to the authors.

Supplementary
