# Supplementary material for: Cascade Cross‐Coupling of Dienes: Photoredox and Nickel Dual Catalysis
Source: Angew Chem Int Ed Engl. 2019 Nov 28;59(1):457–64. doi: 10.1002/anie.201911109 (PMC6973272; doi:10.1002/anie.201911109)
Supplement: Supplementary file 1 — Supplementary [file ANIE-59-457-s001.pdf]

Angewandte Chemie
Eine Zeitschrift der Gesellschaft Deutscher Chemiker

Supporting Information

Cascade Cross-Coupling of Dienes: Photoredox and Nickel Dual Catalysis

Long Huang, Chen Zhu, Liang Yi, Huifeng Yue, Rajesh Kancherla, and Magnus Rueping**

anie_201911109_sm_miscellaneous_information.pdf

Table of Contents

General Methods

Mechanistic studies

General procedure for the cross coupling reaction

Characterization of the products

References

NMR spectra

General Methods. Unless otherwise noted, all commercially available compounds were used as provided without further purification. Solvents for chromatography were technical grade and distilled prior to use. Dry Toluene, 1, 2-dichloroethane (DCE), chloroform, chlorobenzene used in reactions were obtained by distilling over calcium hydride and were stored over activated molecular sieves (4 Å). Dry isopropyl ether ($^{i}Pr_2O$), diethyl ether (Et_2O), tetrahydrofuran (THF) and toluene used in reactions were obtained by distilling over sodium-benzophenone ketyl. Analytical thin-layer chromatography (TLC) was performed on Macherey-Nagel silica gel 60 aluminium plates with F-254 indicator, visualised by UV irradiation. Column chromatography was performed using MN silica gel (particle size 0.040-0.063 mm). ^{1}H-NMR and ^{13}C-NMR spectra were recorded on a vnmrs-400 or vnmrs-600 spectrometer in $CDCl_3$ with residual proton signal of the deuterated solvents as the internal reference (δH = 7.26 ppm and δC = 77.16 ppm for $CDCl_3$). Data are reported in the following order: chemical shift (δ) in ppm; multiplicities of ^{1}H NMR are indicated s (singlet), d (doublet), t (triplet), q (quartet), m (multiplet), dd (doublet of doublet), tt (triplet of triplet), dt (doublet of triplet), td (triplet of doublet), qd (quartet of doublet); coupling constants (*J*) are in Hertz (Hz). All ^{13}C NMR spectra were measured with ^{1}H decoupling. IR spectra were recorded on a Jasco FT/IR-420 spectrometer and are reported in terms of frequency of absorption (cm^{-1}). Mass spectra were acquired on a Finnigan SSQ7000 (EI/CI) spectrometer and high resolution mass spectra on a Finnigan MAT 95 (EI/CI) or on a ThermoFisher Scientific LTQOrbitrap XL (ESI). Melting points were recorded on a Büchi 560 Melting Point Apparatus. The Blue LED strips 19.2 W/m were purchased from ledxon gmbh (Germany). $PhSO_2Na$ and TsNa were commercially available, the other sulfinic acid sodium salts were prepared from the corresponding sulfonyl chlorides according to a previous report.[1]

Stern-Volmer quenching experiments

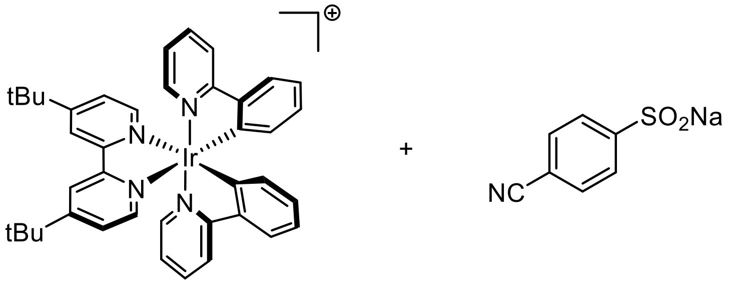

Ir-PC	CN-quencher	Ratio (Ir: CN)
0.00001M	0.0001	1:10
	0.0002	1:20
	0.0003	1:30
	0.0004	1:40
	0.0005	1:50
	0.0006	1:60
	0.0007	1:70

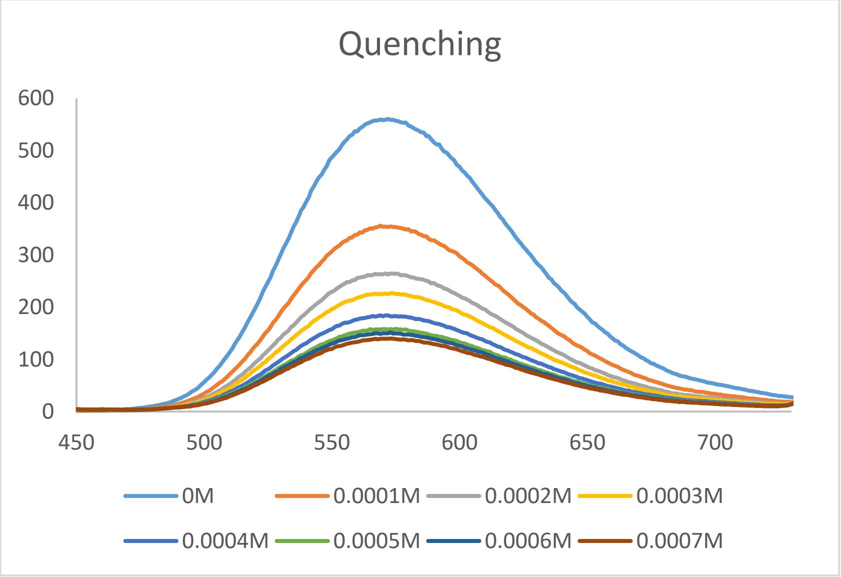

Fig. S1 | Emission spectra of PCI at different concentrations of sodium 4-cyanobenzenesulfinate

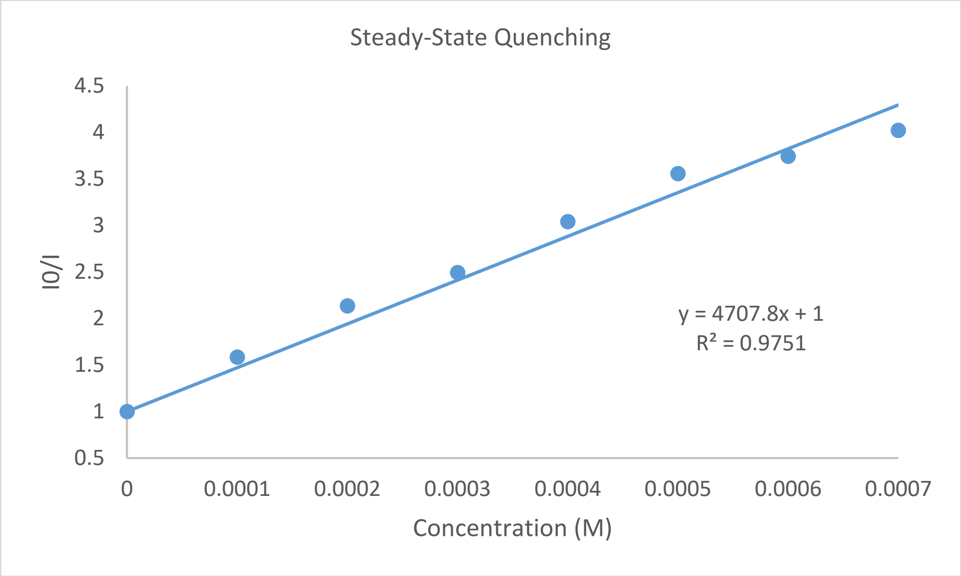

Fig. S2 | Stern-Volmer plot of PCI at different concentrations of sodium 4-cyanobenzenesulfinate

Cyclic Voltammetry measurements of aryl sulfinate salts

Cyclic voltammograms were taken on a PGSTAT101 from Metrohm Autolab using a platinum working electrode, a Ag^+(0.01 M $AgNO_3$, 0.1 M NBu_4PF_6, CH_3CN)/Ag as reference electrode, a platinum wire counter electrode and 0.1 M NBu_4PF_6 as supporting electrolyte. The solution was prepared in CH_3CN (10 mM) and degassed with nitrogen bubbling for 20 min prior to voltammetric studies. The scan rate was 20 mV/s. The potentials were given relative to the Fc/Fc^+ redox couple with ferrocene as internal standard. For conversion to SCE as reference, it is known that SCE is 400 mV more negative than Fc/Fc^+ in MeCN with NBu_4PF_6 as supporting electrolyte.

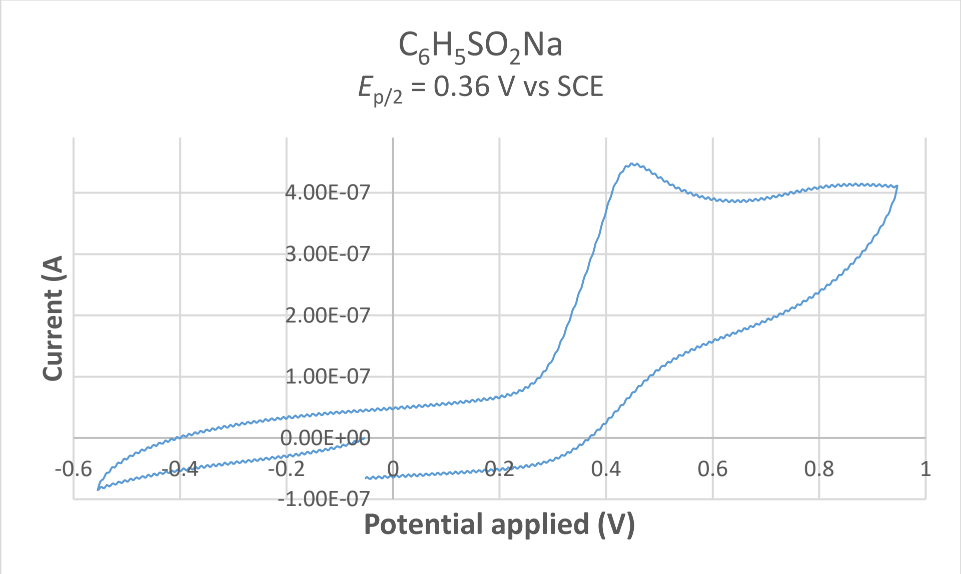

Fig. S3 | Cyclic voltammetry of sodium benzenesulfinate.

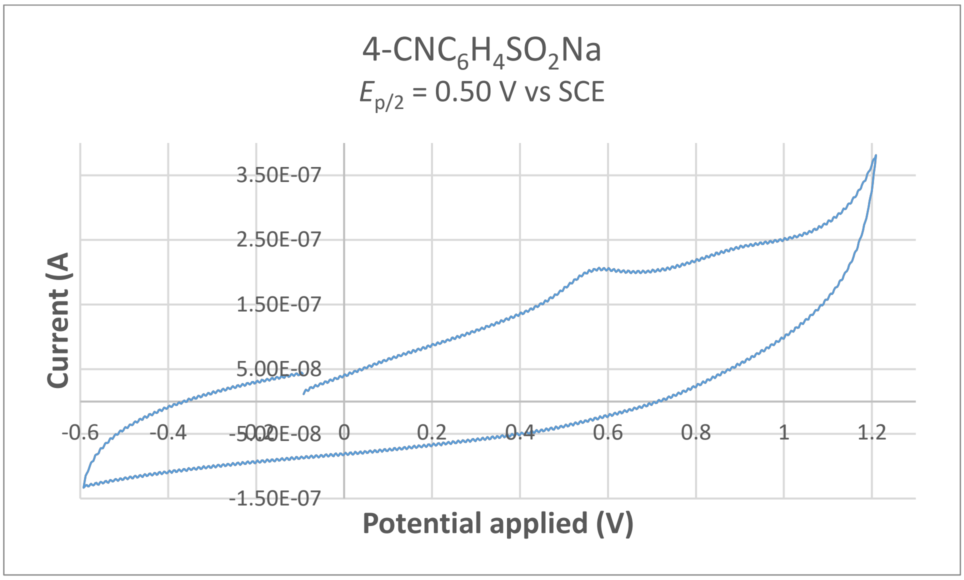

Fig. S4 | Cyclic voltammetry of sodium 4-cyanobenzenesulfinate.

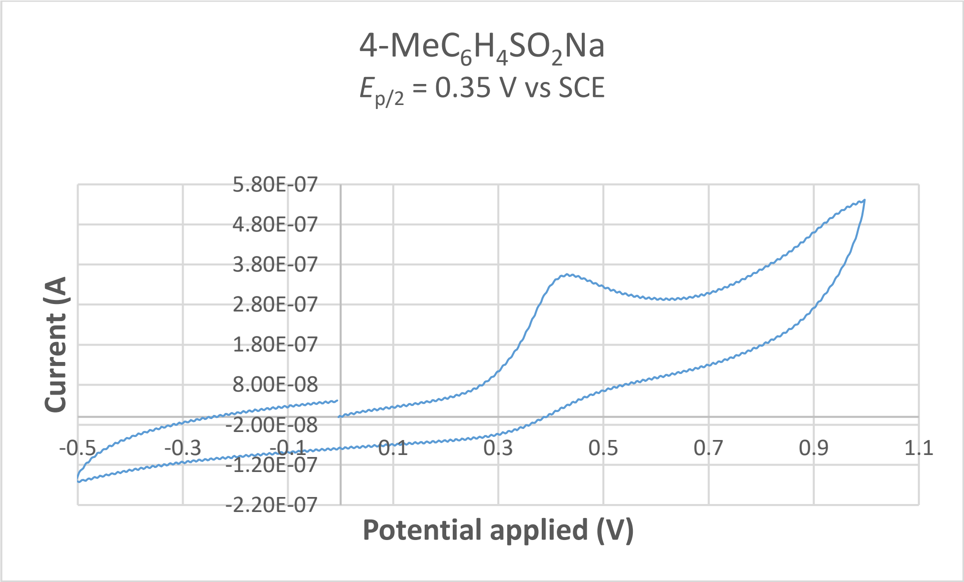

Fig. S5 | Cyclic voltammetry of sodium 4-methylbenzenesulfinate.

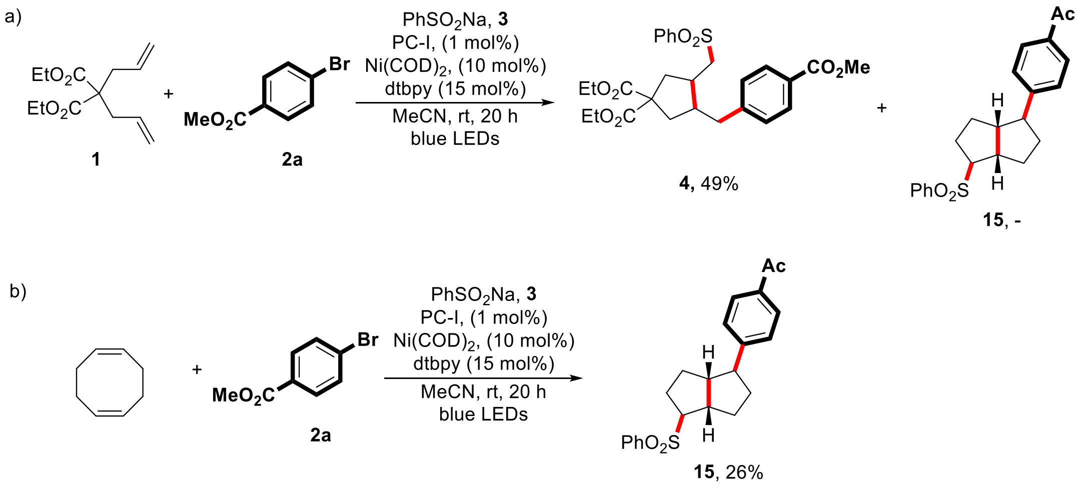

Fig. S6 | Control experiment with $Ni(COD)_2$ as the precatalyst. a) The use of 10 mol% $Ni(COD)_2$ resulted in only 58% yield for the template reaction of **1**, **2a** and **3**. Meanwhile, we do not observe any formation of **15**. b) Similarly, we also observed a severe decline of yield for the formation of **15**. These results indicate that a nickel (0) bounded diene intermediate is less likely involved in the reaction.

a)

Ir photocatalysts	yield (%)	E_T (kcal/mol)	$E_{1/2}$ (M*/M−)	$E_{1/2}$ (M*/M+)
**$[Ir(ppy)_2(bpy)](PF_6)$**	71	46.3	+0.68	-0.85
**$[Ir(ppy)_2(dtbbpy)](PF_6)$**	91	49.2	+0.66	-0.96
**$Ir(ppy)_3$**	82	55.2	+0.55	-1.97
**$Ir(dFCF_3ppy)_2(dtbbpy)PF_6$**	88	60.1	+1.42	-1.00
**$Ir(dFCF_3ppy)_2(bpy)PF_6$**	67	60.4	+0.97	-0.97

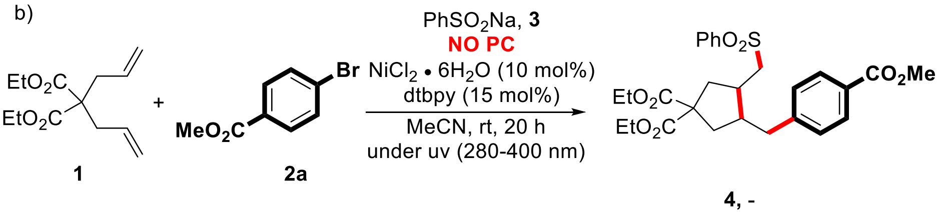

Fig. S7 | The pathway involving the triplet-triplet energy transfer is rather unlikely based on, a) Reactivity on various Ir photosensitizers' triplet excited-state energy E_T, a series of Ir-based photocatalysts were prepared and subjected to the standard reaction conditions. As shown above, we did not observe the good correlation between the reaction efficiency and the energy of the Ir-based excited state. In fact, photocatalyst with highest triplet energy gave the worst result. b) UVA and UVB irradiation of the reaction mixture in the absence of any photosensitizer also gave no cross coupled product.

General procedure for the cross coupling reaction

To a 15 mL vial equipped with a stir bar was added $NiCl_2$•$6H_2O$ (4.8 mg, 0.02 mmol, 10 mol%), 4,4'-di-tert-butyl-2,2'-bipyridine (8.1 mg, 0.03 mmol, 15 mol%), arylhalide (0.2 mmol, 1 equiv.), $[Ir(dtbbpy)(ppy)_2]PF_6$ (1.8 mg, 0.002 mmol, 1 mol%), diene (0.4 mmol, 2 equiv., volative diene was added after sparging) and sodium sulfinate salt (0.4 mmol, 2 equiv.). The vial was sealed, evacuated and backfilled with Argon three times, then 2 mL of MeCN was added and the reaction mixture sparged with Argon for 10 minutes. Afterwards, it was stirred and irradiated with the corresponding blue LEDs photoreactor. Upon completion, the reaction mixture was concentrated in vacuo and purified with column chromatography to afford the desired product.

General procedure for the 4 mmol scale-up cross coupling reaction

To a 100 mL flask equipped with a stir bar was added $NiCl_2$•$6H_2O$ (71.3 mg, 0.3 mmol, 0.1 equiv.) and 4,4'-di-tert-butyl-2,2'-bipyridine (121 mg, 0.45 mmol, 0.15 equiv), carbazolyl dicyanobenzene **PC-V** (59.2 mg, 0.075 mmol, 0.1 equiv.), 4-bromo-1,1'-biphenyl (699 mg, 3 mmol, 1 equiv.), diethyl diallylmalonate (1.44 g, 6 mmol, 2 equiv.) and $PhSO_2Na$ (985 mg, 6 mmol, 2 equiv.). The flask was sealed, evacuated and backfilled with argon three times, then 30 mL of MeCN was added. After sparging with argon for 20 minutes, the flask was stirred and irradiated with the corresponding blue LEDs photoreactor. After 36 h, the crude product was purified by flash column chromatography to afford **28** as a colorless oil in 96% yield (1.54 g).

Characterization of the cross coupling products

Diethyl 3-(4-(methoxycarbonyl)benzyl)-4-((phenylsulfonyl)methyl)cyclopentane-1,1-dicarboxylate,

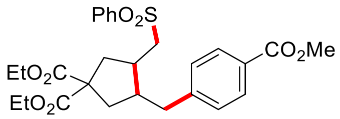

4: the title compound was prepared following the general procedure using $NiCl_2$•$6H_2O$ (4.8 mg, 0.02 mmol, 10 mol%), 4,4'-di-tert-butyl-2,2'-bipyridine (8.1 mg, 0.03 mmol, 15 mol%), methyl 4-bromobenzoate (43 mg, 0.2 mmol, 1 equiv.), $[Ir(dtbbpy)(ppy)_2]PF_6$ (1.8 mg, 0.002 mmol, 1 mol%), diethyl diallylmalonate (96.1 mg, 0.4 mmol, 2 equiv.) and $PhSO_2Na$ (65.7 mg, 0.4 mmol, 2 equiv.). The reaction mixture was stirred in MeCN (2 mL) and irradiated with a 10 W blue LED strip at room temperature for 24 h. The crude product was purified by flash column chromatography to afford 90.9 mg (88%, 88:12 d.r.) of **4** as a colorless oil. When methyl 4-chlorobenzoate (34.1 mg, 0.2 mmol, 1 equiv.) was employed, 60.2 mg (58%) of **4** were obtained.

^{1}H NMR (400 MHz, $CDCl_3$): δ 7.96 – 7.85(m, 4H), 7.64 (t, *J* = 7.4 Hz, 1H), 7.54 (t, *J* = 7.7 Hz, 2H), 7.19 (d, *J* = 8.1 Hz, 2H), 4.18 (q, *J* = 7.1 Hz, 2H), 4.12 (q, *J* = 6.9 Hz, 2H), 3.88 (s, 3H), 3.32 – 3.12 (m, 2H), 2.76 (dd, *J* = 13.0, 4.7 Hz, 1H), 2.64 –2.45 (m, 3H), 2.40 – 2.25 (m, 2H), 2.17 (dd, *J* = 14.3, 6.7 Hz, 1H), 2.04 (dd, *J* = 14.4, 5.4 Hz, 1H), 1.22 (t, *J* = 7.1 Hz, 3H), 1.18 (t, *J* = 7.1 Hz, 3H).

^{13}C NMR (101 MHz, $CDCl_3$): δ 172.4, 171.9, 166.9, 145.3, 139.4, 133.7, 129.7, 129.3, 128.9, 128.1, 127.8, 61.7, 61.6, 57.9, 55.9, 51.9, 43.3, 38.1, 37.3, 37.0, 34.4, 13.9 (2C).

IR (ATR) ν 3881, 3419, 2920, 2860, 2322, 2092, 1996, 1923, 1719, 1608, 1435, 1376, 1277, 1181, 1107, 1019, 965, 844, 759, 723 cm^{-1}.

HRMS (EI) for $C_{14}H_{18}O_2^+$ $(M)^+$: 218.1301; Found : 218.1300.

Dimethyl 3-(4-acetylbenzyl)-4-((phenylsulfonyl)methyl)cyclopentane-1,1-dicarboxylate, **5**: the title

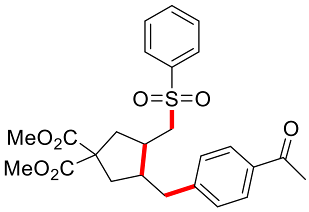

compound was prepared following the general procedure using $NiCl_2$•$6H_2O$ (4.8 mg, 0.02 mmol, 10 mol%), 4,4'-di-tert-butyl-2,2'-bipyridine (8.1 mg, 0.03 mmol, 15 mol%), 1-(4-bromophenyl)ethan-1-one (39.8 mg, 0.2 mmol, 1 equiv.), $[Ir(dtbbpy)(ppy)_2]PF_6$ (1.8 mg, 0.002 mmol, 1 mol%), dimethyl 2,2-diallylmalonate (84.9 mg, 0.4 mmol, 2 equiv.) and $PhSO_2Na$ (65.7 mg, 0.4 mmol, 2 equiv.). The reaction mixture was stirred in MeCN (2 mL) and irradiated with a 10 W blue LED strip at room temperature for 24 h. The crude product was purified by flash column chromatography to afford 92.5 mg (98%, 87:13 d.r.) of **5** as a white solid.

^{1}H NMR (400 MHz, $CDCl_3$): δ 7.87 (d, *J* = 7.3 Hz, 2H), 7.81 (d, *J* = 8.1 Hz, 2H), 7.67 – 7.58 (m, 1H), 7.53 (t, *J* = 7.6 Hz, 2H), 7.19 (d, *J* = 8.0 Hz, 2H), 3.70 (s, 3H), 3.64 (s, 3H), 3.24 (dd, *J* = 14.0, 5.9 Hz, 1H), 3.15 (dd, *J* = 14.0, 7.7 Hz, 1H), 2.76 (dd, *J* = 13.1, 4.7 Hz, 1H), 2.64 – 2.46 (m, 6H), 2.37 – 2.21 (m, 2H), 2.15 (dd, *J* = 14.4, 6.7 Hz, 1H), 2.04 (dd, *J* = 14.3, 5.4 Hz, 1H).

^{13}C NMR (101 MHz, $CDCl_3$): δ 197.8, 173.0, 172.5, 145.7, 139.6, 135.4, 133.9, 129.5, 129.2, 128.7, 128.0, 58.0, 56.1, 53.1, 53.0, 43.5, 38.3, 37.6, 37.1, 34.6, 26.6.

IR (ATR) ν 2951, 1729, 1679, 1604, 1440, 1361, 1263, 1147, 1082, 1019, 957, 913, 732 cm^{-1}.

HRMS (ESI) for $C_{25}H_{28}O_7NaS^+$ $(M+Na)^+$: 495.1435; Found : 495.1443.

Dibenzyl 3-(4-acetylbenzyl)-4-((phenylsulfonyl)methyl)cyclopentane-1,1-dicarboxylate, **6**: the title

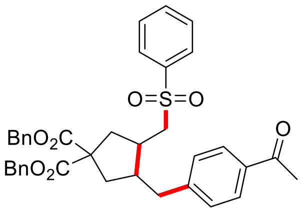

compound was prepared following the general procedure using $NiCl_2$•$6H_2O$ (4.8 mg, 0.02 mmol, 10 mol%), 4,4'-di-tert-butyl-2,2'-bipyridine (8.1 mg, 0.03 mmol, 15 mol%), 1-(4-bromophenyl)ethan-1-one (39.8 mg, 0.2 mmol, 1 equiv.), $[Ir(dtbbpy)(ppy)_2]PF_6$ (1.8 mg, 0.002 mmol, 1 mol%), dibenzyl 2,2-diallylmalonate (146 mg, 0.4 mmol, 2 equiv.) and $PhSO_2Na$ (65.7 mg, 0.4 mmol, 2 equiv.). The reaction mixture was stirred in MeCN (2 mL) and irradiated with a 10 W blue LED strip at room temperature for 24 h. The crude product was purified by flash column chromatography to afford 113 mg (90%, 89:11 d.r.) of **3a** as a white solid.

^{1}H NMR (600 MHz, $CDCl_3$): δ 7.91 – 7.87 (m, 2H), 7.81 (d, *J* = 8.3 Hz, 2H), 7.67 – 7.62 (m, 1H), 7.54 (t, *J* = 7.8 Hz, 2H), 7.34 – 7.30 (m, 3H), 7.30 – 7.27 (m, 3H), 7.25 – 7.21 (m, 2H), 7.20 – 7.15 (m, 4H), 5.16 – 5.08 (m, 2H), 5.08 – 5.02 (m, 2H), 3.27 (dd, *J* = 14.1, 6.0 Hz, 1H), 3.19 (dd, *J* = 14.1, 7.7 Hz, 1H), 2.78 (dd, *J* = 13.1, 4.6 Hz, 1H), 2.67 – 2.61 (m, 1H), 2.60 – 2.56 (m, 4H), 2.55 – 2.48 (m, 1H), 2.37 – 2.28 (m, 2H), 2.21 (dd, *J* = 14.3, 6.9 Hz, 1H), 2.09 (dd, *J* = 14.4, 5.2 Hz, 1H).

^{13}C NMR (151 MHz, CDCl$_3$): δ 197.8, 172.2, 171.7, 145.7, 139.5, 135.3, 135.2, 135.2, 133.9, 129.5, 129.2, 128.6, 128.6, 128.5, 128.4, 128.2, 128.1, 128.0, 67.6, 67.5, 58.2, 56.1, 43.5, 38.3, 37.4, 37.2, 34.4, 26.6.

IR (ATR) ν 3036, 2943, 1728, 1679, 1604, 1449, 1365, 1303, 1256, 1148, 1085, 1016, 955, 909, 736 cm^{-1}.

HRMS (ESI) for $C_{37}H_{36}O_7NaS^+$ (M+Na)$^+$: 647.2074; Found : 647.2072.

1,1'-(3-(4-Acetylbenzyl)-4-((phenylsulfonyl)methyl)cyclopentane-1,1-diyl)bis(ethan-1-one), **7**: the title compound was prepared following the general procedure using NiCl$_2$•6H$_2$O (4.8 mg, 0.02 mmol, 10 mol%), 4,4'-di-tert-butyl-2,2'-bipyridine (8.1 mg, 0.03 mmol, 15 mol%), 1-(4-bromophenyl)ethan-1-one (39.8 mg, 0.2 mmol, 1 equiv.), [Ir(dtbbpy)(ppy)$_2$]PF$_6$ (1.8 mg, 0.002 mmol, 1 mol%), 3,3-diallylpentane-2,4-dione (72.1 mg, 0.4 mmol, 2 equiv.) and PhSO$_2$Na (65.7 mg, 0.4 mmol, 2 equiv.). The reaction mixture was stirred in MeCN (2 mL) and irradiated with a 10 W blue LED strip at room temperature for 24 h. The crude product was purified by flash column chromatography to afford 78.9 mg (90%, 85:15 d.r.) of **7** as a colorless oil.

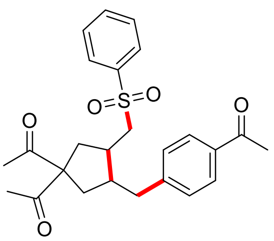

^{1}H NMR (400 MHz, CDCl$_3$): δ 7.87 (d, *J* = 7.1 Hz, 2H), 7.82 (d, *J* = 8.2 Hz, 2H), 7.67 – 7.59 (m, 1H), 7.58 – 7.49 (m, 2H), 7.20 (d, *J* = 8.1 Hz, 2H), 3.19 (dd, *J* = 14.0, 5.3 Hz, 1H), 3.05 (dd, *J* = 14.0, 7.4 Hz, 1H), 2.70 (dd, *J* = 13.1, 4.2 Hz, 1H), 2.54 (s, 3H), 2.52 – 2.35 (m, 3H), 2.22 – 2.02 (m, 6H), 1.98 (s, 3H), 1.90 (dd, *J* = 14.1, 6.7 Hz, 1H).

^{13}C NMR (101 MHz, CDCl$_3$): δ 204.5, 203.5, 197.8, 145.6, 139.5, 135.5, 134.0, 129.5, 129.3, 128.7, 128.0, 73.4, 56.1, 43.5, 37.1, 34.5, 34.4, 33.6, 26.9, 26.7, 26.2.

IR (ATR) ν 2930, 1687, 1604, 1433, 1360, 1298, 1143, 1082, 956, 853, 796, 737 cm^{-1}.

HRMS (ESI) for $C_{25}H_{28}O_5NaS^+$ (M+Na)$^+$: 463.1550; Found : 463.1543.

2-(4-Acetylbenzyl)-8,8-dimethyl-3-((phenylsulfonyl)methyl)spiro[4.5]decane-6,10-dione, **8**: the title compound was prepared following the general procedure using NiCl$_2$•6H$_2$O (4.8 mg, 0.02 mmol, 10 mol%), 4,4'-di-tert-butyl-2,2'-bipyridine (8.1 mg, 0.03 mmol, 15 mol%), 1-(4-bromophenyl)ethan-1-one (39.8 mg, 0.2 mmol, 1 equiv.), [Ir(dtbbpy)(ppy)$_2$]PF$_6$ (1.8 mg, 0.002 mmol, 1 mol%), 2,2-diallyl-5,5-dimethylcyclohexane-1,3-dione (88.1 mg, 0.4 mmol, 2 equiv.) and PhSO$_2$Na (65.7 mg, 0.4 mmol, 2 equiv.). The reaction mixture was stirred in MeCN (2 mL) and irradiated with a 10 W blue LED strip at room temperature for 24 h. The crude product was purified by flash column chromatography to afford 75.7 mg (79%, 88:12 d.r.) of **8** as a white solid.

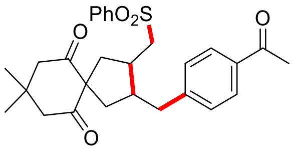

^{1}H NMR (600 MHz, CDCl$_3$): δ 7.90 – 7.86 (m, 2H), 7.82 (d, *J* = 8.3 Hz, 2H), 7.66 – 7.62 (m, 1H), 7.55 (t, *J* = 7.8 Hz, 2H), 7.16 (d, *J* = 8.1 Hz, 2H), 3.22 – 3.17 (m, 2H), 2.74 (dd, *J* = 13.2, 5.0 Hz, 1H), 2.63 –

2.58 (m, 2H), 2.58 – 2.52 (m, 5H), 2.51 (d, *J* = 6.4 Hz, 2H), 2.43 – 2.34 (m, 2H), 2.24 (dd, *J* = 13.5, 6.8 Hz, 1H), 1.93 (dd, *J* = 13.6, 6.8 Hz, 1H), 1.86 (dd, *J* = 13.7, 5.6 Hz, 1H), 0.92 (s, 3H), 0.92 (s, 3H).

^{13}C NMR (151 MHz, CDCl$_3$): δ 208.2, 207.1, 197.8, 145.8, 139.6, 135.4, 133.9, 129.5, 129.2, 128.7, 127.9, 69.3, 55.7, 51.8, 51.3, 44.2, 37.6, 36.0, 35.4, 34.4, 30.5, 28.6, 28.1, 26.7.

IR (ATR) ν 2954, 1683, 1605, 1447, 1362, 1301, 1261, 1143, 1081, 1017, 955, 913, 858, 795, 735 cm^{-1}.

HRMS (ESI) for $C_{28}H_{32}O_5NaS^+$ $(M+Na)^+$: 503.1863; Found : 503.1850.

(3-(4-Acetylbenzyl)-4-((phenylsulfonyl)methyl)cyclopentane-1,1-diyl)bis(methylene) bis(4-methyl-benzenesulfonate), **9**: the title compound was prepared following the general procedure using NiCl$_2$•6H$_2$O (4.8 mg, 0.02 mmol, 10 mol%), 4,4'-di-tert-butyl-2,2'-bipyridine (8.1 mg, 0.03 mmol, 15 mol%), 1-(4-bromophenyl)ethan-1-one (39.8 mg, 0.2 mmol, 1 equiv.), [Ir(dtbbpy)(ppy)$_2$]PF$_6$ (1.8 mg, 0.002 mmol, 1 mol%), 2,2-diallylpropane-1,3-diyl bis(4-methylbenzenesulfonate) (186 mg, 0.4 mmol, 2 equiv.) and PhSO$_2$Na (65.7 mg, 0.4 mmol, 2 equiv.). The reaction mixture was stirred in MeCN (2 mL) and irradiated with a 10 W blue LED strip at room temperature for 24 h. The crude product was purified by flash column chromatography to afford 121 mg (84%, 86:14 d.r.) of **9** as a white solid. **^{1}H NMR (600 MHz, CDCl$_3$):** δ 7.87 (dd, *J* = 8.2, 1.3 Hz, 2H), 7.81 (d, *J* = 8.1 Hz, 2H), 7.73 (d, *J* = 8.2 Hz, 2H), 7.66 (t, *J* = 8.3 Hz, 3H), 7.55 (t, *J* = 7.7 Hz, 2H), 7.35 (d, *J* = 8.0 Hz, 2H), 7.31 (d, *J* = 8.1 Hz, 2H), 7.12 (d, *J* = 8.1 Hz, 2H), 3.85 – 3.78 (m, 2H), 3.73 – 3.64 (m, 2H), 3.19 (dd, *J* = 14.0, 5.8 Hz, 1H), 3.05 (dd, *J* = 13.9, 8.2 Hz, 1H), 2.76 (dd, *J* = 13.4, 4.8 Hz, 1H), 2.59 – 2.51 (m, 4H), 2.47 – 2.42 (m, 7H), 2.24 (dd, *J* = 13.3, 11.3 Hz, 1H), 1.75 (dd, *J* = 14.2, 6.9 Hz, 1H), 1.65 (dd, *J* = 14.2, 9.2 Hz, 1H), 1.40 – 1.28 (m, 2H).

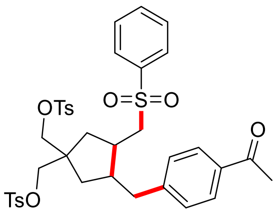

^{13}C NMR (151 MHz, CDCl$_3$): δ 197.7, 145.4, 145.3, 139.5, 135.4, 134.0, 132.2, 132.1, 130.1, 130.1, 129.5, 129.1, 128.7, 128.0, 127.9, 72.6, 71.8, 56.3, 44.9, 42.7, 36.1, 35.8, 35.2, 34.9, 26.7, 21.8, 21.7.

IR (ATR) □ 2939, 1678, 1600, 1448, 1356, 1302, 1173, 1093, 959, 819 cm^{-1}.

HRMS (ESI) for $C_{37}H_{40}O_9NaS_3^+$ $(M+Na)^+$: 747.1727; Found : 747.1727.

1-(4-((8,8-Dimethyl-3-((phenylsulfonyl)methyl)-7,9-dioxaspiro[4.5]decan-2-yl)methyl)phenyl) ethan-1-one, **10**: the title compound was prepared following the general procedure using NiCl$_2$•6H$_2$O (4.8 mg, 0.02 mmol, 10 mol%), 4,4'-di-tert-butyl-2,2'-bipyridine (8.1 mg, 0.03 mmol, 15 mol%), 1-(4-bromophenyl)ethan-1-one (39.8 mg, 0.2 mmol, 1 equiv.), [Ir(dtbbpy)(ppy)$_2$]PF$_6$ (1.8 mg, 0.002 mmol, 1 mol%), 5,5-diallyl-2,2-dimethyl-1,3-dioxane (78.5 mg, 0.4 mmol, 2 equiv.) and PhSO$_2$Na (65.7 mg, 0.4 mmol, 2 equiv.). The reaction mixture was stirred in MeCN (2 mL) and irradiated with a 10 W blue LED strip at room temperature for 24 h. The crude product was purified by flash column chromatography to afford 75.8 mg (83%, 87:13 d.r.) of **10** as a white solid.

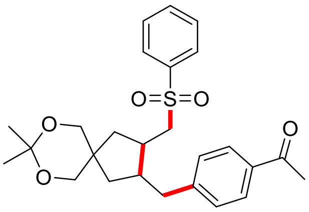

^{1}H NMR (600 MHz, CDCl$_3$): δ 7.92 – 7.87 (m, 2H), 7.83 (d, *J* = 8.2 Hz, 2H), 7.68 – 7.63 (m, 1H), 7.55 (t, *J* = 7.8 Hz, 2H), 7.17 (d, *J* = 8.0 Hz, 2H), 3.66 (s, 2H), 3.44 (s, 2H), 3.29 (dd, *J* = 14.1, 6.5 Hz, 1H), 3.08 (dd, *J* = 14.1, 7.2 Hz, 1H), 2.86 (dd, *J* = 13.3, 4.6 Hz, 1H), 2.56 (s, 3H), 2.55 – 2.46 (m, 2H), 2.24 (dd, *J* = 13.2, 11.0 Hz, 1H), 1.94 (dd, *J* = 13.8, 6.7 Hz, 1H), 1.56 (dd, *J* = 13.8, 9.3 Hz, 1H), 1.47 (dd, *J* = 14.2, 6.9 Hz, 1H), 1.35 (s, 3H), 1.33 (s, 3H), 1.21 (dd, *J* = 14.1, 4.7 Hz, 1H).

^{13}C NMR (151 MHz, CDCl$_3$): δ 197.8, 146.0, 139.7, 135.3, 133.9, 129.5, 129.2, 128.7, 127.9, 97.8, 71.3, 68.9, 56.9, 42.3, 40.3, 38.1, 36.8, 36.2, 35.7, 26.7, 24.6, 23.0.

IR (ATR) ν 3498, 2934, 2865, 1677, 1604, 1448, 1366, 1268, 1192, 1146, 1078, 1026, 915, 824, 730 cm^{-1}.

HRMS (ESI) for $C_{26}H_{32}O_5NaS^+$ $(M+Na)^+$: 479.1863; Found : 479.1863.

1-(4-((4-((Phenylsulfonyl)methyl)tetrahydrofuran-3-yl)methyl)phenyl)ethan-1-one, **11**: the title compound was prepared following the general procedure using $NiCl_2{\bullet}6H_2O$ (4.8 mg, 0.02 mmol, 10 mol%), 4,4'-di-tert-butyl-2,2'-bipyridine (8.1 mg, 0.03 mmol, 15 mol%), 1-(4-bromophenyl)ethan-1-one (39.8 mg, 0.2 mmol, 1 equiv.), [Ir(dtbbpy)(ppy)$_2$]PF$_6$ (1.8 mg, 0.002 mmol, 1 mol%), diallylether (39.3 mg, 0.4 mmol, 2 equiv.) and $PhSO_2Na$ (65.7 mg, 0.4 mmol, 2 equiv.). The reaction mixture was stirred in MeCN (2 mL) and irradiated with a 10 W blue LED strip at room temperature for 24 h. The crude product was purified by flash column chromatography to afford 67.4 mg (94%, 78:22 d.r.) of **11** as a white solid.

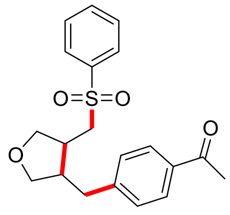

^{1}H NMR (600 MHz, CDCl$_3$): *cis* isomer δ 7.92 – 7.87 (m, 2H), 7.84 (d, *J* = 8.2 Hz, 2H), 7.69 – 7.65 (m, 1H), 7.57 (t, *J* = 7.8 Hz, 2H), 7.20 (d, *J* = 8.1 Hz, 2H), 3.99 (dd, *J* = 8.9, 7.3 Hz, 1H), 3.73 (dd, *J* = 8.8, 7.3 Hz, 1H), 3.67 (dd, *J* = 8.8, 6.0 Hz, 1H), 3.49 (dd, *J* = 8.8, 4.5 Hz, 1H), 3.32 (dd, *J* = 14.0, 5.2 Hz, 1H), 3.16 (dd, *J* = 14.0, 8.9 Hz, 1H), 2.86 – 2.76 (m, 2H), 2.73 – 2.63 (m, 1H), 2.56 (s, 3H), 2.45 (dd, *J* = 13.3, 11.0 Hz, 1H); *trans* isomer δ 7.82 (d, *J* = 8.2 Hz, 2H), 7.79 – 7.75 (m, 2H), 7.14 (d, *J* = 8.1 Hz, 2H), 4.10 (dd, *J* = 9.4, 7.4 Hz, 1H), 3.82 (dd, *J* = 9.0, 7.2 Hz, 1H), 3.59 (dd, *J* = 9.5, 6.4 Hz, 1H), 3.45 (dd, *J* = 9.0, 6.5 Hz, 1H), 3.02 (dd, *J* = 14.1, 9.2 Hz, 1H), 2.96 (dd, *J* = 14.1, 4.6 Hz, 1H), 2.57 (s, 3H), 2.40 – 2.32 (m, 1H), 2.30 – 2.24 (m, 1H), the remaining resonances are insufficiently resolved from those of the cis isomer to be reported.

^{13}C NMR (151 MHz, CDCl$_3$): *cis* isomer δ 197.7, 145.2, 139.3, 135.6, 134.1, 134.0, 129.6, 129.0, 128.8, 128.0, 71.4, 71.3, 54.9, 42.9, 36.6, 33.5, 26.7; *trans* isomer δ 197.6, 145.0, 139.1, 135.5, 134.1, 134.0, 129.5, 129.0, 128.8, 128.0, 72.9, 72.4, 59.4, 46.4, 39.4, 38.7 the remaining resonances are insufficiently resolved from those of the cis isomer to be reported.

IR (ATR) ν 2931, 2865, 1725, 1678, 1604, 1445, 1359, 1269, 1142, 1073, 958, 912, 796, 738 cm^{-1}.

HRMS (ESI) for $C_{20}H_{22}O_4NaS^+$ $(M+Na)^+$: 381.1131; Found : 381.1131.

1-(4-((4-((Phenylsulfonyl)methyl)-1-tosylpyrrolidin-3-yl)methyl)phenyl)ethan-1-one, **12**: the title

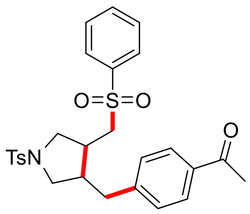

compound was prepared following the general procedure using $NiCl_2•6H_2O$ (4.8 mg, 0.02 mmol, 10 mol%), 4,4'-di-tert-butyl-2,2'-bipyridine (8.1 mg, 0.03 mmol, 15 mol%), 1-(4-bromophenyl)ethan-1-one (39.8 mg, 0.2 mmol, 1 equiv.), $[Ir(dtbbpy)(ppy)_2]PF_6$ (1.8 mg, 0.002 mmol, 1 mol%), N,N-diallyl-4-methylbenzenesulfonamide (101 mg, 0.4 mmol, 2 equiv.) and $PhSO_2Na$ (65.7 mg, 0.4 mmol, 2 equiv.). The reaction mixture was stirred in MeCN (2 mL) and irradiated with a 10 W blue LED strip at room temperature for 24 h. The crude product was purified by flash column chromatography to afford 96.3 mg (94%, 73:27 d.r.) of **12** as a white solid.

^{1}H NMR (400 MHz, $CDCl_3$): *cis* isomer δ 7.88 – 7.75 (m, 4H), 7.69 – 7.61 (m, 3H), 7.52 (t, *J* = 7.5 Hz, 2H), 7.30 (d, *J* = 7.6 Hz, 2H), 7.05 (d, *J* = 8.0 Hz, 2H), 3.47 (dd, *J* = 10.4, 7.3 Hz, 1H), 3.34 (dd, *J* = 10.3, 7.5 Hz, 1H), 3.17 (dd, *J* = 13.9, 5.3 Hz, 1H), 3.08 – 2.97 (m, 2H), 2.90 (dd, *J* = 13.9, 8.4 Hz, 1H), 2.70 (dd, *J* = 13.3, 4.5 Hz, 1H), 2.66 – 2.58 (m, 1H), 2.58 – 2.48 (m, 4H), 2.41 (s, 3H), 2.25 (dd, *J* = 13.1, 10.9 Hz, 1H; *trans* isomer δ 7.72 (d, *J* = 7.9 Hz, 2H), 3.61 (dd, *J* = 10.7, 7.2 Hz, 1H), 3.24 (dd, *J* = 10.1, 6.9 Hz, 1H), the remaining resonances are insufficiently resolved from those of the cis isomer to be reported.

^{13}C NMR (101 MHz, $CDCl_3$): *cis* isomer δ 197.6, 144.3, 143.9, 139.1, 135.7, 134.2, 133.8, 129.9, 129.6, 129.1, 128.8, 127.9, 127.5, 54.4, 50.8, 50.7, 42.5, 35.9, 33.2, 26.6, 21.6; *trans* isomer δ 197.5, 143.9, 138.9, 135.7, 134.1, 132.9, 129.9, 129.5, 128.9, 128.8, 127.7, 58.7, 52.6, 51.7, 44.8, 38.2, 38.0, the remaining resonances are insufficiently resolved from those of the cis isomer to be reported.

IR (ATR) ν 2932, 1725, 1678, 1603, 1446, 1304, 1150, 1087, 1032, 960, 911, 810, 732 cm^{-1}.

HRMS (ESI) for $C_{27}H_{29}O_5NNaS_2^+$ $(M+Na)^+$: 534.1379; Found : 534.1375.

1-(4-(1,1-diphenyl-5-((phenylsulfonyl)methyl)silinan-3-yl)phenyl)ethan-1-one, **13**: the title

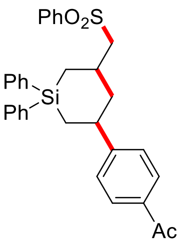

compound was prepared following the general procedure using $NiCl_2•6H_2O$ (4.8 mg, 0.02 mmol, 10 mol%), 4,4'-di-tert-butyl-2,2'-bipyridine (8.1 mg, 0.03 mmol, 15 mol%), 1-(4-bromophenyl)ethan-1-one (39.8 mg, 0.2 mmol, 1 equiv.), $[Ir(dtbbpy)(ppy)_2]PF_6$ (1.8 mg, 0.002 mmol, 1 mol%), diallyldiphenylsilane (106 mg, 0.4 mmol, 2 equiv.) and $PhSO_2Na$ (65.7 mg, 0.4 mmol, 2 equiv.). The reaction mixture was stirred in MeCN (2 mL) and irradiated with a 10 W blue LED strip at room temperature for 24 h. The crude product was purified by flash column chromatography to afford 69.6 mg (66%, the stereochemistry was assigned to be cis as confirmed by ^{1}H NMR, 2D NMR experiments by comparison with literature precedent)[2] of **13** as a colorless oil.

^{1}H NMR (600 MHz, $CDCl_3$): δ 7.93 – 7.84 (m, 4H), 7.63 (dd, *J* = 7.6, 1.8 Hz, 3H), 7.53 (t, *J* = 7.8 Hz, 2H), 7.51 – 7.40 (m, 5H), 7.38 – 7.33 (m, 1H), 7.35 – 7.29 (m, 2H), 7.28 (d, *J* = 8.3 Hz, 2H), 3.21 – 3.10 (m, 2H), 2.96 – 2.87 (m, 1H), 2.58 (s, 3H), 2.52 – 2.44 (m, 1H), 2.07 – 1.96 (m, 1H), 1.92 – 1.85 (m, 1H), 1.61 (dt, *J* = 14.6, 2.2 Hz, 1H), 1.52 – 1.43 (m, 1H), 1.26 (t, *J* = 14.0 Hz, 1H), 0.99 – 0.90 (m, 1H).

^{13}C NMR (151 MHz, CDCl$_3$): δ 197.9, 154.5, 140.1, 135.7, 135.3, 134.7, 134.3, 133.7, 133.6, 129.9, 129.8, 129.4, 128.9, 128.5, 128.1, 127.9, 126.6, 65.7, 43.8, 41.7, 32.1, 26.7, 19.1, 18.5.

IR (ATR) ν 3060, 2914, 1677, 1602, 1426, 1358, 1300, 1270, 1147, 1113, 967, 856, 775 cm^{-1}.

HRMS (ESI) for $C_{32}H_{32}O_3NaSSi^+$ $(M+Na)^+$: 547.1734; Found : 547.1724.

1-(4-(2,3-Dimethyl-4-(phenylsulfonyl)but-2-en-1-yl)phenyl)ethan-1-one, **14**: the title compound was

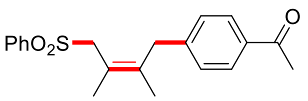

prepared following the general procedure using $NiCl_2$•$6H_2O$ (4.8 mg, 0.02 mmol, 10 mol%), 4,4'-di-tert-butyl-2,2'-bipyridine (8.1 mg, 0.03 mmol, 15 mol%), 1-(4-bromophenyl)ethan-1-one (39.8 mg, 0.2 mmol, 1 equiv.), [Ir(dtbbpy)(ppy)$_2$]PF$_6$ (1.8 mg, 0.002 mmol, 1 mol%), 2,3-dimethylbuta-1,3-diene (32.9 mg, 0.4 mmol, 2 equiv.) and $PhSO_2Na$ (65.7 mg, 0.4 mmol, 2 equiv.). The reaction mixture was stirred in MeCN (2 mL) and irradiated with a 10 W blue LED strip at room temperature for 24 h. The crude product was purified by flash column chromatography to afford 67.6 mg (64%, 1.3:1 *Z*/*E*, the stereochemistry was assigned by ^{1}H NMR and NOESY experiments) of **14** as a white solid.

^{1}H NMR (400 MHz, CDCl$_3$): *E* + *Z* isomer δ 7.90 – 7.75 (m, 7H_{E+Z}), 7.68 – 7.50 (m, 3.5H_{E+Z}), 7.49 – 7.40 (m, 1.75H_{E+Z}), 7.13 (d, *J* = 8.0 Hz, 1.5H_E), 7.08 (d, *J* = 7.9 Hz, 2H_Z), 3.94 (s, 2H_Z), 3.92 (s, 1.5H_E), 3.40 (s, 1.5H_E), 3.18 (s, 2H_Z), 2.56 (s, 2.2H_E), 2.53 (s, 3H_Z), 1.90 (d, *J* = 1.8 Hz, 2.2H_E), 1.84 (d, *J* = 1.5 Hz, 3H_Z), 1.55 (s, 3H_Z), 1.26 (s, 2.2H_E).

^{13}C NMR (100 MHz, CDCl$_3$): *E* + *Z* isomer δ 197.8 (2C), 145.0 (2C), 139.5, 139.3, 137.5, 137.4, 135.5, 135.4, 133.8, 133.7, 129.3, 129.2, 128.7 (2C), 128.6, 128.4, 128.3, 119.1, 118.9, 61.9, 61.7, 40.5, 40.0, 26.7 (2C), 20.1, 19.9, 19.1, 18.9.

IR (ATR) ν 2922, 1674, 1603, 1441, 1361, 1271, 1135, 1081, 1016, 958, 863, 742 cm^{-1}.

HRMS (ESI) for $C_{20}H_{22}O_3NaS^+$ $(M+Na)^+$: 365.1182; Found : 365.1182.

1-(4-((1R,3aR,4R,6aS)-4-(phenylsulfonyl)octahydropentalen-1-yl)phenyl)ethan-1-one, **15**: the title

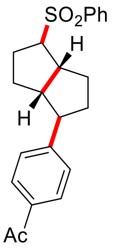

compound was prepared following the general procedure using $NiCl_2$•$6H_2O$ (4.8 mg, 0.02 mmol, 10 mol%), 4,4'-di-tert-butyl-2,2'-bipyridine (8.1 mg, 0.03 mmol, 15 mol%), 1-(4-bromophenyl)ethan-1-one (39.8 mg, 0.2 mmol, 1 equiv.), [Ir(dtbbpy)(ppy)$_2$]PF$_6$ (1.8 mg, 0.002 mmol, 1 mol%), 1,5-cyclooctadiene (43.3 mg, 0.4 mmol, 2 equiv.) and $PhSO_2Na$ (65.7 mg, 0.4 mmol, 2 equiv.). The reaction mixture was stirred in MeCN (2 mL) and irradiated with a 10 W blue LED strip at room temperature for 24 h. The crude product was purified by flash column chromatography to afford 43.8 mg (60%) of **16** as a colorless oil. The stereochemistry was assigned by ^{1}H NMR HSQC, NOESY experiments and by comparison with literature precedent.[4]

^{1}H NMR (600 MHz, CDCl$_3$): δ 7.95 – 7.90 (m, 2H), 7.87 (d, *J* = 8.4 Hz, 2H), 7.68 – 7.62 (m, 1H), 7.57 (t, *J* = 7.8 Hz, 2H), 7.25 (d, *J* = 8.3 Hz, 2H), 3.29 – 3.21 (m, 1H), 3.12 (tt, *J* = 8.4, 6.4 Hz, 1H), 2.75 – 2.66 (m, 2H), 2.57 (d, *J* = 1.0 Hz, 3H), 2.18 – 2.02 (m, 4H), 1.99 – 1.91 (m, 1H), 1.78 – 1.68 (m, 1H), 1.50 – 1.42 (m, 1H), 1.31 – 1.24 (m, 1H).

^{13}C NMR (151 MHz, CDCl$_3$): δ 197.7, 150.0, 138.8, 135.4, 133.6, 129.2, 128.6, 128.5, 127.3, 72.0, 52.8, 52.2, 45.1, 36.4, 32.8, 31.0, 29.0, 26.6.

IR (ATR) ν 2942, 2866, 1680, 1603, 1445, 1358, 1267, 1140, 1083, 1016, 955, 832, 758, 721 cm^{-1}.

HRMS (ESI) for $C_{22}H_{24}O_3NaS^+$ $(M+Na)^+$: 391.1338; Found : 391.1334.

1-(4-(5-(Phenylsulfonyl)tricyclo[2.2.1.02,6]heptan-3-yl)phenyl)ethan-1-one, **16**: the title compound

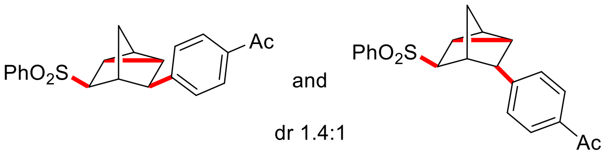

was prepared following the general procedure using NiCl$_2$•6H$_2$O (4.8 mg, 0.02 mmol, 10 mol%), 4,4'-di-tert-butyl-2,2'-bipyridine (8.1 mg, 0.03 mmol, 15 mol%), 1-(4-bromophenyl)ethan-1-one (39.8 mg, 0.2 mmol, 1 equiv.), [Ir(dtbbpy)(ppy)$_2$]PF$_6$ (1.8 mg, 0.002 mmol, 1 mol%), 2,5-norbornadiene (55.3 mg, 0.4 mmol, 2 equiv.) and PhSO$_2$Na (65.7 mg, 0.4 mmol, 2 equiv.). The reaction mixture was stirred in MeCN (2 mL) and irradiated with a 10 W blue LED strip at room temperature for 24 h. The crude product was purified by flash column chromatography to afford 54.7 mg (78%, 1.4:1 dr, the stereochemistry was assigned by ^{1}H NMR, NOESY experiments and by comparison with literature precedent)[3] of **15** as a white solid.

^{1}H NMR (600 MHz, CDCl$_3$): δ 7.93 (dd, *J* = 8.4, 1.3 Hz, 1.4H$_{minor}$), 7.88 – 7.81 (m, 3.4H$_{major+minor}$), 7.80 – 7.75 (m, 2H$_{major}$), 7.67 (t, *J* = 7.5 Hz, 0.7H$_{minor}$), 7.63 – 7.56 (m, 2.4H$_{major+minor}$), 7.50 (t, *J* = 7.8 Hz, 2H$_{major}$), 7.28 – 7.21 (m, 3.4H$_{major+minor}$), 3.28 (d, *J* = 1.3 Hz, 0.7H$_{minor}$), 3.07 (s, 1H$_{major}$), 2.92 (d, *J* = 1.6 Hz, 0.7H$_{minor}$), 2.86 (t, *J* = 1.2 Hz, 1H$_{major}$), 2.60 – 2.55 (m, 5.1H$_{major+minor}$), 2.53 (dt, *J* = 10.9, 1.7 Hz, 1H$_{major}$), 2.40 – 2.35 (m, 1.7H$_{major+minor}$), 2.19 (dt, *J* = 11.5, 1.6 Hz, 1H$_{minor}$), 1.77 – 1.64 (m, 5.1H$_{major+minor}$), 1.61 (d, *J* = 10.9 Hz, 1H$_{major}$), 1.18 (d, *J* = 11.5 Hz, 0.7H$_{minor}$).

^{13}C NMR (151 MHz, CDCl$_3$): δ 197.9, 197.8, 145.0, 140.0, 139.9, 135.9, 135.8, 133.8, 133.6, 129.4, 129.3, 128.6, 128.3, 128.2, 128.1, 128.0, 70.8, 67.0, 51.6, 50.5, 39.2, 38.9, 32.0, 26.7 (2C), 26.3, 16.4, 16.2, 14.2, 13.5, 12.8, 12.0.IR (ATR) ν 3064, 2937, 1678, 1603, 1443, 1357, 1267, 1143, 1084, 1015, 958, 816, 723 cm^{-1}. HRMS (ESI) for $C_{20}H_{22}O_3NaS^+$ $(M+Na)^+$: 365.1182; Found : 365.1182.

Diethyl 3-(4-acetylbenzyl)-4-((phenylsulfonyl)methyl)cyclopentane-1,1-dicarboxylate, **17**: the title

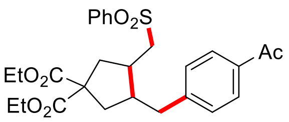

compound was prepared following the general procedure using NiCl$_2$•6H$_2$O (4.8 mg, 0.02 mmol, 10 mol%), 4,4'-di-tert-butyl-2,2'-bipyridine (8.1 mg, 0.03 mmol, 15 mol%), 1-(4-bromophenyl)ethan-1-one (39.8 mg, 0.2 mmol, 1 equiv.), [Ir(dtbbpy)(ppy)$_2$]PF$_6$ (1.8 mg, 0.002 mmol, 1 mol%), diethyl diallylmalonate (96.1 mg, 0.4 mmol, 2 equiv.) and PhSO$_2$Na (65.7 mg, 0.4 mmol, 2 equiv.). The reaction mixture was stirred in MeCN (2 mL) and irradiated with a 10 W blue LED strip at room temperature for 24 h. The crude product was purified by flash column chromatography to afford 98.1 mg (98%, 86:14 d.r.) of **17** as a colorless oil. When 1-(4-chlorophenyl)ethan-1-one (30.9 mg, 0.2 mmol, 1 equiv.) was employed, 73.4 mg (73%) of **17** were obtained.

^{1}H NMR (400 MHz, CDCl$_3$): δ 7.88 (d, *J* = 7.1 Hz, 2H), 7.82 (d, *J* = 8.0 Hz, 2H), 7.63 (t, *J* = 7.4 Hz, 1H), 7.54 (t, *J* = 7.7 Hz, 2H), 7.21 (d, *J* = 8.1 Hz, 2H), 4.17 (q, *J* = 7.1 Hz, 2H), 4.10 (qd, *J* = 7.1, 1.3 Hz, 3H), 3.25 (dd, *J* = 14.0, 5.9 Hz, 1H), 3.16 (dd, *J* = 14.0, 7.6 Hz, 1H), 2.76 (dd, *J* = 13.0, 4.5 Hz, 1H), 2.61 – 2.47 (m, 6H), 2.37 – 2.23 (m, 2H), 2.15 (dd, *J* = 14.3, 6.7 Hz, 1H), 2.04 (dd, *J* = 14.3, 5.3 Hz, 1H), 1.21 (t, *J* = 7.1 Hz, 3H), 1.16 (t, *J* = 7.1 Hz, 3H).

^{13}C NMR (101 MHz, CDCl$_3$): δ 197.8, 172.6, 172.1, 145.8, 139.6, 135.4, 133.9, 129.5, 129.3, 128.7, 128.0, 61.9, 61.8, 58.1, 56.2, 43.5, 38.3, 37.5, 37.2, 34.6, 26.7, 14.1 (2C).

IR (ATR) ν 2980, 1725, 1680, 1603, 1449, 1414, 1364, 1258, 1144, 1093, 1019, 958, 914, 859, 762, 734 cm^{-1}.

HRMS (ESI) for $C_{27}H_{33}O_7NNaS^+$ (M+Na)$^+$: 538.1870; Found : 538.1870.

Diethyl 3-(4-benzoylbenzyl)-4-((phenylsulfonyl)methyl)cyclopentane-1,1-dicarboxylate, **18**: the title

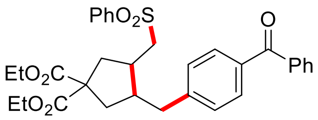

compound was prepared following the general procedure using $NiCl_2$•$6H_2O$ (4.8 mg, 0.02 mmol, 10 mol%), 4,4'-di-tert-butyl-2,2'-bipyridine (8.1 mg, 0.03 mmol, 15 mol%), 4-Bromobenzophenone (52.2 mg, 0.2 mmol, 1 equiv.), [Ir(dtbbpy)(ppy)$_2$]PF_6 (1.8 mg, 0.002 mmol, 1 mol%), diethyl diallylmalonate (96.1 mg, 0.4 mmol, 2 equiv.) and $PhSO_2Na$ (65.7 mg, 0.4 mmol, 2 equiv.). The reaction mixture was stirred in MeCN (2 mL) and irradiated with a 10 W blue LED strip at room temperature for 24 h. The crude product was purified by flash column chromatography to afford 104.7 mg (93%, 89:11 d.r., the stereochemistry was assigned by ^{1}H NMR, NOESY experiments and by comparison with literature precedent)[5] of **18** as a colorless oil. When 4-chlorobenzophenone (43.3 mg, 0.2 mmol, 1 equiv.) was employed, 78.5 mg (70%) of **18** were obtained.

^{1}H NMR (600 MHz, CDCl$_3$): δ 7.95 – 7.87 (m, 2H), 7.79 – 7.74 (m, 2H), 7.70 (d, *J* = 8.2 Hz, 2H), 7.66 – 7.62 (m, 1H), 7.59 – 7.53 (m, 3H), 7.46 (t, *J* = 7.7 Hz, 2H), 7.25 (d, *J* = 8.2 Hz, 2H), 4.19 (q, *J* = 7.1 Hz, 2H), 4.13 (qd, *J* = 7.1, 3.8 Hz, 2H), 3.29 (dd, *J* = 14.1, 6.0 Hz, 1H), 3.20 (dd, *J* = 14.1, 7.9 Hz, 1H), 2.81 (dd, *J* = 13.1, 4.6 Hz, 1H), 2.66 – 2.58 (m, 1H), 2.54 (dd, *J* = 13.7, 6.9 Hz, 2H), 2.37 (dd, *J* = 13.1, 11.1 Hz, 1H), 2.30 (dd, *J* = 14.0, 8.9 Hz, 1H), 2.20 (dd, *J* = 14.3, 6.9 Hz, 1H), 2.11 (dd, *J* = 14.3, 5.3 Hz, 1H), 1.23 (t, *J* = 7.2 Hz, 3H), 1.19 (t, *J* = 7.1 Hz, 3H).

^{13}C NMR (151 MHz, CDCl$_3$): δ 196.4, 172.6, 172.1, 145.2, 139.5, 137.8, 135.6, 133.9, 132.4, 130.5, 130.0, 129.5, 129.0, 128.3, 128.0, 61.9, 61.7, 58.1, 56.1, 43.5, 38.2, 37.5, 37.2, 34.6, 14.1, 14.0.

IR (ATR) ν 2979, 1725, 1654, 1601, 1448, 1375, 1262, 1147, 1087, 1015, 928, 859, 785, 697 cm^{-1}.

HRMS (ESI) for $C_{32}H_{34}O_7NaS^+$ (M+Na)$^+$: 585.1917; Found : 509.1908.

Diethyl 3-(4-cyanobenzyl)-4-((phenylsulfonyl)methyl)cyclopentane-1,1-dicarboxylate, **19**: the title

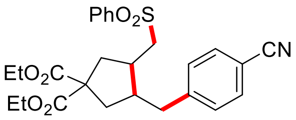

compound was prepared following the general procedure using $NiCl_2$•$6H_2O$ (4.8 mg, 0.02 mmol, 10 mol%), 4,4'-di-tert-butyl-2,2'-bipyridine (8.1 mg, 0.03 mmol, 15 mol%), methyl 4-bromobenzonitrile (36.4 mg, 0.2 mmol, 1

equiv.), [Ir(dtbbpy)(ppy)$_2$]PF$_6$ (1.8 mg, 0.002 mmol, 1 mol%), diethyl diallylmalonate (96.1 mg, 0.4 mmol, 2 equiv.) and PhSO$_2$Na (65.7 mg, 0.4 mmol, 2 equiv.). The reaction mixture was stirred in MeCN (2 mL) and irradiated with a 10 W blue LED strip at room temperature for 24 h. The crude product was purified by flash column chromatography to afford 77.4 mg (80%, 90:10 d.r.) of **19** as a colorless oil.

^{1}H NMR (400 MHz, CDCl$_3$): δ 7.94 – 7.85 (m, 2H), 7.65 (t, *J* = 7.4 Hz, 1H), 7.61 – 7.47 (m, 4H), 7.27 (d, *J* = 7.9 Hz, 2H), 4.16 (q, *J* = 7.1 Hz, 2H), 4.10 (qd, *J* = 7.1, 2.0 Hz, 2H), 3.23 (dd, *J* = 14.1, 6.7 Hz, 1H), 3.17 (dd, *J* = 14.0, 6.8 Hz, 1H), 2.81 (dd, *J* = 13.0, 4.2 Hz, 1H), 2.65 –2.46 (m, 3H), 2.37 – 2.26 (m, 1H), 2.24 – 2.02 (m, 3H), 1.20 (t, *J* = 7.1 Hz, 3H), 1.16 (t, *J* = 7.1 Hz, 3H).

^{13}C NMR (101 MHz, CDCl$_3$): δ 172.6, 172.0, 145.9, 139.5, 134.0, 132.3, 130.0, 129.5, 128.0, 119.0, 110.2, 61.9, 61.8, 57.9, 56.0, 43.4, 38.3, 37.3 (2C), 34.4, 14.1, 14.0.

IR (ATR) ν 2981, 2935, 2227, 1724, 1607, 1505, 1447, 1409, 1368, 1301, 1257, 1145, 1090, 1026, 913, 859, 794, 733 690 cm^{-1}.

HRMS (ESI) for $C_{26}H_{29}O_6NNaS^+$ (M+Na)$^+$: 506.1608; Found : 506.1607.

Diethyl 3-((phenylsulfonyl)methyl)-4-(4-(trifluoromethyl)benzyl)cyclopentane-1,1-dicarboxylate, **20**:

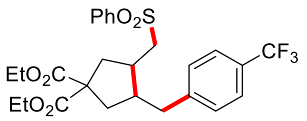

the title compound was prepared following the general procedure using NiCl$_2$•6H$_2$O (4.8 mg, 0.02 mmol, 10 mol%), 4,4'-di-tert-butyl-2,2'-bipyridine (8.1 mg, 0.03 mmol, 15 mol%), 4-bromobenzotrifluoride (45.0 mg, 0.2 mmol, 1 equiv.), [Ir(dtbbpy)(ppy)$_2$]PF$_6$ (1.8 mg, 0.002 mmol, 1 mol%), diethyl diallylmalonate (96.1 mg, 0.4 mmol, 2 equiv.) and PhSO$_2$Na (65.7 mg, 0.4 mmol, 2 equiv.). The reaction mixture was stirred in MeCN (2 mL) and irradiated with a 10 W blue LED strip at room temperature for 24 h. The crude product was purified by flash column chromatography to afford 100.1 mg (95%, 88:12 d.r.) of **20** as a colorless oil.

^{1}H NMR (400 MHz, CDCl$_3$): δ 7.93 – 7.84 (m, 2H), 7.68 – 7.61 (m, 1H), 7.55 (t, *J* = 7.6 Hz, 2H), 7.48 (d, *J* = 8.0 Hz, 2H), 7.25 (d, *J* = 8.1 Hz, 2H), 4.18 (q, *J* = 7.1 Hz, 2H), 4.11 (qd, *J* = 7.1 Hz, 1.6 Hz, 2H), 3.25 (dd, *J* = 14.0, 6.0 Hz, 1H), 3.17 (dd, *J* = 14.0, 7.3 Hz, 1H), 2.78 (dd, *J* = 13.1, 4.4 Hz, 1H), 2.64 – 2.45 (m, 3H), 2.34 (dd, *J* = 13.0, 10.9 Hz, 1H), 2.26 (dd, *J* = 13.5, 8.5 Hz, 1H), 2.15 (dd, *J* = 14.3, 6.7 Hz, 1H), 2.06 (dd, *J* = 14.3, 5.3 Hz, 1H), 1.22 (t, *J* = 7.1 Hz, 3H), 1.18 (t, *J* = 7.1 Hz, 3H).

^{13}C {^{19}F, ^{1}H} NMR (101 MHz, CDCl$_3$): δ 172.7, 172.1, 144.2, 139.6, 134.0, 129.5, 129.4, 128.6, 128.1, 125.5, 61.9, 61.8, 58.1, 56.1, 43.6, 38.3, 37.5, 37.2, 34.3, 14.1 (2C).

^{19}F NMR (376 MHz, CDCl$_3$): *cis* isomer δ -62.40; *trans* isomer δ -62.43.

IR (ATR) ν 2981, 1726, 1617, 1450, 1314, 1258, 1116, 1022, 861, 744, 690 cm^{-1}.

HRMS (ESI) for $C_{26}H_{29}O_6F_3NaS^+$ (M+Na)$^+$: 549.1529; Found : 549.1539.

Diethyl 3-(4-formylbenzyl)-4-((phenylsulfonyl)methyl)cyclopentane-1,1-dicarboxylate, **21**: the title

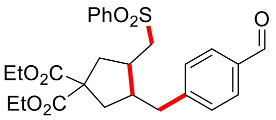

compound was prepared following the general procedure using $NiCl_2$•$6H_2O$ (4.8 mg, 0.02 mmol, 10 mol%), 4,4'-di-tert-butyl-2,2'-bipyridine (8.1 mg, 0.03 mmol, 15 mol%), 4-bromobenzaldehyde (37.0 mg, 0.2 mmol, 1 equiv.), $[Ir(dtbbpy)(ppy)_2]PF_6$ (1.8 mg, 0.002 mmol, 1 mol%), diethyl diallylmalonate (96.1 mg, 0.4 mmol, 2 equiv.) and $PhSO_2Na$ (65.7 mg, 0.4 mmol, 2 equiv.). The reaction mixture was stirred in MeCN (2 mL) and irradiated with a 10 W blue LED strip at room temperature for 24 h. The crude product was purified by flash column chromatography to afford 96.9 mg (99%, 87:13 d.r.) of **21** as a colorless oil.

^{1}H NMR (400 MHz, $CDCl_3$): δ 9.92 (s, 1H), 7.92 – 7.84 (m, 2H), 7.73 (d, J = 8.2 Hz, 2H), 7.66 – 7.59 (m, 1H), 7.53 (t, J = 7.6 Hz, 2H), 7.29 (d, J = 7.9 Hz, 2H), 4.16 (q, J = 7.0 Hz, 2H), 4.09 (qd, J = 7.1, 1.4 Hz, 2H), 3.21 (dd, J = 14.0, 6.7 Hz, 2H), 2.80 (dd, J = 13.0, 4.4 Hz, 1H), 2.64 – 2.46 (m, 4H), 2.34 (dd, J = 13.0, 11.0 Hz, 1H), 2.24 (dd, J = 13.8, 8.7 Hz, 1H), 2.13 (dd, J = 14.1, 6.9 Hz, 1H), 2.09 – 1.97 (m, 1H), 1.20 (t, J = 7.1 Hz, 3H), 1.15 (t, J = 7.1 Hz, 3H).

^{13}C NMR (101 MHz, $CDCl_3$): δ 192.0, 172.6, 172.0, 147.5, 139.6, 134.8, 133.9, 130.0, 129.8, 129.5, 128.0, 61.9, 61.7, 58.0, 56.1, 43.5, 38.3, 37.4, 37.2, 34.6, 14.1, 14.0.

IR (ATR) ν 2979, 1721, 1604, 1448, 1373, 1301, 1258, 1148, 1089, 1015, 854, 784, 747, 690 cm^{-1}.

HRMS (ESI) for $C_{26}H_{30}O_7NaS^+$ $(M+Na)^+$: 509.1604; Found : 509.1599.

Diethyl 3-(4-fluorobenzyl)-4-((phenylsulfonyl)methyl)cyclopentane-1,1-dicarboxylate, **22**: the title

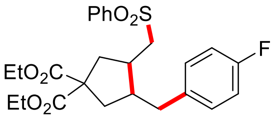

compound was prepared following the general procedure using $NiCl_2$•$6H_2O$ (4.8 mg, 0.02 mmol, 10 mol%), 4,4'-di-tert-butyl-2,2'-bipyridine (8.1 mg, 0.03 mmol, 15 mol%), 1-bromo-4-fluorobenzene (35 mg, 0.2 mmol, 1 equiv.), $[Ir(dtbbpy)(ppy)_2]PF_6$ (1.8 mg, 0.002 mmol, 1 mol%), diethyl diallylmalonate (96.1 mg, 0.4 mmol, 2 equiv.) and $PhSO_2Na$ (65.7 mg, 0.4 mmol, 2 equiv.). The reaction mixture was stirred in MeCN (2 mL) and irradiated with a 10 W blue LED strip at room temperature for 24 h. The crude product was purified by flash column chromatography to afford 85.0 mg (89%, 88:12 d.r.) of **22** as a colorless oil.

^{1}H NMR (400 MHz, $CDCl_3$): δ 7.87 (d, *J* = 7.2 Hz, 2H), 7.64 (t, *J* = 7.4 Hz, 1H), 7.59 – 7.50 (m, 2H), 7.10 – 7.02 (m, 2H), 6.90 (t, *J* = 8.7 Hz, 2H), 4.20 – 4.08 (m, 4H), 3.24 (dd, *J* = 13.9, 5.6 Hz, 1H), 3.15 (dd, *J* = 14.0, 7.7 Hz, 1H), 2.66 (dd, *J* = 13.3, 4.9 Hz, 1H), 2.59 – 2.47 (m, 2H), 2.43 (dt, *J* = 11.2, 5.3 Hz, 1H), 2.30 – 2.20 (m, 2H), 2.15 (dd, *J* = 14.3, 6.8 Hz, 1H), 2.05 (dd, *J* = 14.3, 5.7 Hz, 1H), 1.21 (t, *J* = 7.1 Hz, 3H), 1.17 (t, *J* = 7.1 Hz, 3H).

^{13}C {^{19}F, ^{1}H} NMR (101 MHz, $CDCl_3$): δ 172.7, 172.2, 161.5, 139.6, 135.5, 133.9, 130.4, 129.5, 128.0, 115.3, 61.9, 61.7, 58.2, 56.1, 43.9, 38.3, 37.5, 37.1, 33.7, 14.1 (2C).

^{13}C NMR (101 MHz, $CDCl_3$): δ 172.7, 172.2, 161.5 (J_{C-F} = 160.2 Hz), 139.6, 135.5 (J_{C-F} = 3.3 Hz), 133.9, 130.4 (J_{C-F} = 7.7 Hz), 129.5, 128.0, 115.3 (J_{C-F} = 21.2 Hz),, 61.9, 61.7, 58.2, 56.1, 43.9, 38.3, 37.5, 37.1, 33.7, 14.1 (2C).

^{19}F NMR (376 MHz, $CDCl_3$): δ -117.1 - -117.2.

IR (ATR) ν 2980, 1725, 1601, 1509, 1450, 1370, 1301, 1257, 1147, 1091, 1017, 855, 747 cm^{-1}.

HRMS (ESI) for $C_{25}H_{29}O_6FNaS^+$ $(M+Na)^+$: 499.1561; Found : 499.1552.

Diethyl 3-(4-chlorobenzyl)-4-((phenylsulfonyl)methyl)cyclopentane-1,1-dicarboxylate, **23**: the title

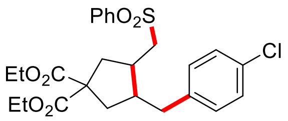

compound was prepared following the general procedure using $NiCl_2$•$6H_2O$ (4.8 mg, 0.02 mmol, 10 mol%), 4,4'-di-tert-butyl-2,2'-bipyridine (8.1 mg, 0.03 mmol, 15 mol%), 1-bromo-4-chlorobenzene (38.3 mg, 0.2 mmol, 1 equiv.), $[Ir(dtbbpy)(ppy)_2]PF_6$ (1.8 mg, 0.002 mmol, 1 mol%), diethyl diallylmalonate (96.1 mg, 0.4 mmol, 2 equiv.) and $PhSO_2Na$ (65.7 mg, 0.4 mmol, 2 equiv.). The reaction mixture was stirred in MeCN (2 mL) and irradiated with a 10 W blue LED strip at room temperature for 24 h. The crude product was purified by flash column chromatography to afford 97.1 mg (99%, 87:13 d.r.) of **23** as a colorless oil.

^{1}H NMR (400 MHz, $CDCl_3$): δ 7.86 (d, *J* = 7.3 Hz, 2H), 7.63 (t, *J* = 7.4 Hz, 1H), 7.53 (t, *J* = 7.6 Hz, 2H), 7.17 (d, *J* = 8.3 Hz, 2H), 7.03 (d, *J* = 8.3 Hz, 2H), 4.20 – 4.08 (m, 4H), 3.22 (dd, *J* = 14.0, 5.5 Hz, 1H), 3.14 (dd, *J* = 14.0, 7.7 Hz, 1H), 2.65 (dd, *J* = 13.1, 5.0 Hz, 1H), 2.58 – 2.40 (m, 3H), 2.33 – 2.20 (m, 2H), 2.14 (dd, *J* = 14.2, 6.8 Hz, 1H), 2.04 (dd, *J* = 14.3, 5.6 Hz, 1H), 1.24 – 1.14 (m, 6H).

^{13}C NMR (101 MHz, $CDCl_3$): δ 172.6, 172.1, 139.5, 138.4, 133.9, 132.0, 130.4, 129.5, 128.6, 128.0, 61.8, 61.7, 58.1, 56.1, 43.6, 38.2, 37.5, 37.1, 33.9, 14.1 (2C).

IR (ATR) ν 2978, 1725, 1452, 1375, 1258, 1148, 1091, 1015, 858, 745, cm^{-1}.

HRMS (ESI) for $C_{25}H_{29}O_6ClNaS^+$ $(M+Na)^+$: 515.1266; Found : 515.1266.

Diethyl 3-((phenylsulfonyl)methyl)-4-(4-(4,4,5,5-tetramethyl-1,3,2-dioxaborolan-2-yl)benzyl)

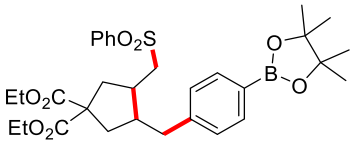

cyclopentane-1,1-dicarboxylate, **24**: the title compound was prepared following the general procedure using $NiCl_2$•$6H_2O$ (4.8 mg, 0.02 mmol, 10 mol%), 4,4'-di-tert-butyl-2,2'-bipyridine (8.1 mg, 0.03 mmol, 15 mol%), 2-(4-bromophenyl)-4,4,5,5-tetramethyl-1,3,2-dioxaborolane (56.6 mg, 0.2 mmol, 1 equiv.), $[Ir(dtbbpy)(ppy)_2]PF_6$ (1.8 mg, 0.002 mmol, 1 mol%), diethyl diallylmalonate (96.1 mg, 0.4 mmol, 2 equiv.) and $PhSO_2Na$ (65.7 mg, 0.4 mmol, 2 equiv.). The reaction mixture was stirred in MeCN (2 mL) and irradiated with a 10 W blue LED strip at room temperature for 24 h. The crude prozduct was purified by flash column chromatography to afford 107 mg (92%, 85:15 d.r.) of **24** as a colorless oil.

^{1}H NMR (400 MHz, $CDCl_3$): δ 7.89 – 7.81 (m, 2H), 7.69 – 7.58 (m, 3H), 7.52 (t, *J* = 7.6 Hz, 2H), 7.07 (d, *J* = 7.7 Hz, 2H), 4.20 – 4.13 (m, 2H), 4.14 – 4.07 (m, 2H), 3.25 (dd, *J* = 14.0, 4.9 Hz, 1H), 3.14 (dd, *J* = 14.0, 8.1 Hz, 1H), 2.66 (dd, *J* = 13.0, 4.9 Hz, 1H), 2.58 – 2.42 (m, 3H), 2.39 – 2.26 (m, 2H), 2.18 (dd, *J* = 14.3, 6.8 Hz, 1H), 2.01 (dd, *J* = 14.3, 5.9 Hz, 1H), 1.31 (s, 12H), 1.21 (t, *J* = 7.1 Hz, 3H), 1.17 (t, *J* = 7.1 Hz, 3H).

^{13}C NMR (101 MHz, $CDCl_3$): δ 172.6, 172.2, 143.2, 139.6, 135.0, 133.8, 129.4, 128.4, 128.0, 83.8, 61.8, 61.7, 58.2, 56.2, 43.6, 38.2, 37.6, 37.0, 35.0, 24.9, 14.1 (2C).

IR (ATR) ν 2981, 2169, 1726, 1611, 1452, 1360, 1307, 1258, 1145, 1089, 1020, 961, 914, 858, 792, 735 cm^{-1}.

HRMS (ESI) for $C_{31}H_{41}O_8BNaS^+$ $(M+Na)^+$: 607.2507; Found : 607.2524.

Diethyl 3-(4-(dimethylamino)benzyl)-4-((phenylsulfonyl)methyl)cyclopentane-1,1-dicarboxylate, **25**:

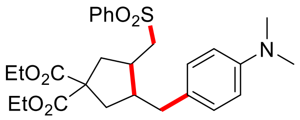

the title compound was prepared following the general procedure using $NiCl_2•6H_2O$ (4.8 mg, 0.02 mmol, 10 mol%), 4,4'-di-tert-butyl-2,2'-bipyridine (8.1 mg, 0.03 mmol, 15 mol%), 4-bromo-N,N-dimethylaniline (40.0 mg, 0.2 mmol, 1 equiv.), $[Ir(dtbbpy)(ppy)_2]PF_6$ (1.8 mg, 0.002 mmol, 1 mol%), diethyl diallylmalonate (96.1 mg, 0.4 mmol, 2 equiv.) and $PhSO_2Na$ (65.7 mg, 0.4 mmol, 2 equiv.). The reaction mixture was stirred in MeCN (2 mL) and irradiated with a 10 W blue LED strip at room temperature for 24 h. The crude product was purified by flash column chromatography to afford 92.2 mg (92%, 86:14 d.r.) of **25** as a colorless oil.

^{1}H NMR (400 MHz, $CDCl_3$): δ 7.86 (d, *J* = 7.3 Hz, 2H), 7.62 (t, *J* = 7.4 Hz, 1H), 7.52 (t, *J* = 7.6 Hz, 2H), 6.93 (d, *J* = 8.5 Hz, 2H), 6.61 (d, *J* = 8.5 Hz, 2H), 4.22 – 4.07 (m, 4H), 3.28 (dd, *J* = 14.1, 4.3 Hz, 1H), 3.14 (dd, *J* = 14.0, 8.6 Hz, 1H), 2.88 (s, 6H), 2.59 – 2.45 (m, 3H), 2.44 – 2.27 (m, 2H), 2.25 – 2.12 (m, 2H), 2.11 – 1.99 (m, 1H), 1.22 (t, *J* = 7.2 Hz, 3H), 1.18 (t, *J* = 7.1 Hz, 3H).

^{13}C NMR (101 MHz, $CDCl_3$): δ 172.7, 172.3, 149.2, 139.7, 133.7, 129.5, 129.4, 128.0, 127.6, 112.9, 61.7, 61.6, 58.4, 56.2, 44.0, 40.8, 38.2, 37.7, 36.9, 33.8, 14.1 (2C).

IR (ATR) ν 2977, 2930, 2803, 1725, 1613, 1521, 1450, 1361, 1302, 1255, 1145, 1091, 944, 857, 801, 746, 689 cm^{-1}.

HRMS (ESI) for $C_{27}H_{36}O_6NS^+$ $(M+H)^+$: 502.2252; Found : 502.2251.

Diethyl 3-(4-(tert-butyl)benzyl)-4-((phenylsulfonyl)methyl)cyclopentane-1,1-dicarboxylate, **26**: the

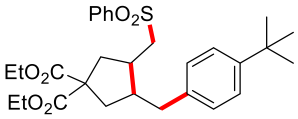

title compound was prepared following the general procedure using $NiCl_2•6H_2O$ (4.8 mg, 0.02 mmol, 10 mol%), 4,4'-di-tert-butyl-2,2'-bipyridine (8.1 mg, 0.03 mmol, 15 mol%), 1-bromo-4-tert-butylbenzene (42.6 mg, 0.2 mmol, 1 equiv.), $[Ir(dtbbpy)(ppy)_2]PF_6$ (1.8 mg, 0.002 mmol, 1 mol%), diethyl diallylmalonate (96.1 mg, 0.4 mmol, 2 equiv.) and $PhSO_2Na$ (65.7 mg, 0.4 mmol, 2 equiv.). The reaction mixture was stirred in MeCN (2 mL) and irradiated with a 10 W blue LED strip at room temperature for 24 h. The crude product was purified by flash column chromatography to afford 83.3 mg (81%, 87:13 d.r.) of **26** as a colorless oil.

^{1}H NMR (400 MHz, $CDCl_3$): δ 7.94 – 7.85 (m, 2H), 7.64 (t, *J* = 7.4 Hz, 1H), 7.54 (t, *J* = 7.6 Hz, 2H), 7.24 (d, *J* = 8.2 Hz, 2H), 7.01 (d, *J* = 8.3 Hz, 2H), 4.22 – 4.07 (m, 4H), 3.28 (dd, *J* = 14.0, 4.9 Hz, 1H), 3.16 (dd, *J* = 14.0, 8.5 Hz, 1H), 2.67 – 2.40 (m, 4H), 2.37 – 2.16 (m, 3H), 2.11 – 2.00 (m, 1H), 1.27 (s, 9H), 1.22 (t, *J* = 7.1 Hz, 3H), 1.18 (t, *J* = 7.1 Hz, 3H).

^{13}C NMR (101 MHz, $CDCl_3$): δ 172.7, 172.3, 149.0, 139.7, 136.7, 133.8, 129.4, 128.5, 128.1, 125.4, 61.8, 61.7, 58.3, 56.3, 43.9, 38.2, 37.7, 37.0, 34.4, 34.3, 31.5, 14.1 (2C).

IR (ATR) ν 2960, 1726, 1513, 1452, 1368, 1303, 1256, 1147, 1094, 1018, 858, 744, 688 cm^{-1}.

HRMS (ESI) for $C_{29}H_{38}O_6NaS^+$ $(M+Na)^+$: 537.2281; Found : 537.2278.

Diethyl 3-(4-methylbenzyl)-4-((phenylsulfonyl)methyl)cyclopentane-1,1-dicarboxylate, **27**: the title

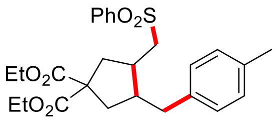

compound was prepared following the general procedure using $NiCl_2$•$6H_2O$ (4.8 mg, 0.02 mmol, 10 mol%), 4,4'-di-tert-butyl-2,2'-bipyridine (8.1 mg, 0.03 mmol, 15 mol%), 1-bromo-4-methylbenzene (34.2 mg, 0.2 mmol, 1 equiv.), [Ir(dtbbpy)(ppy)$_2$]PF_6 (1.8 mg, 0.002 mmol, 1 mol%), diethyl 2,2-diallylmalonate (96.1 mg, 0.4 mmol, 2 equiv.) and $PhSO_2Na$ (65.7 mg, 0.4 mmol, 2 equiv.). The reaction mixture was stirred in MeCN (2 mL) and irradiated with a 10 W blue LED strip at room temperature for 24 h. The crude product was purified by flash column chromatography to afford 71.1 mg (75%, 89:11 d.r.) of **27** as a white solid.

^{1}H NMR (600 MHz, $CDCl_3$): δ 7.92 – 7.86 (m, 2H), 7.65 (t, *J* = 7.5 Hz, 1H), 7.55 (t, *J* = 7.7 Hz, 2H), 7.04 (d, *J* = 7.8 Hz, 2H), 6.98 (d, *J* = 7.8 Hz, 2H), 4.19 (q, *J* = 7.1 Hz, 2H), 4.13 (qd, *J* = 7.2, 2.8 Hz, 2H), 3.28 (dd, *J* = 14.1, 4.9 Hz, 1H), 3.17 (dd, *J* = 14.0, 8.6 Hz, 1H), 2.63 (dd, *J* = 13.3, 5.1 Hz, 1H), 2.59 – 2.49 (m, 2H), 2.48 – 2.41 (m, 1H), 2.34 (dd, *J* = 13.7, 8.0 Hz, 1H), 2.32 – 2.24 (m, 4H), 2.20 (dd, *J* = 14.3, 6.9 Hz, 1H), 2.07 (dd, *J* = 14.3, 6.1 Hz, 1H), 1.24 (t, *J* = 7.1 Hz, 3H), 1.20 (t, *J* = 7.1 Hz, 3H).

^{13}C NMR (151 MHz, $CDCl_3$): δ 172.7, 172.3, 139.6, 136.7, 135.7, 133.8, 129.4, 129.2, 128.8, 128.0, 61.8, 61.7, 58.3, 56.2, 43.9, 38.2, 37.6, 37.0, 34.3, 21.1, 14.1 (2C).

IR (ATR) ν 3637, 3550, 3453, 2982, 2931, 1907, 1727, 1585, 1515, 1448, 1369, 1304, 1258, 1148, 1092, 1028, 915, 859, 794, 747 cm^{-1}.

HRMS (ESI) for $C_{26}H_{32}O_6NaS^+$ $(M+Na)^+$: 495.1812; Found : 495.1789.

Diethyl 3-([1,1'-biphenyl]-4-ylmethyl)-4-((phenylsulfonyl)methyl)cyclopentane-1,1-dicarboxylate,

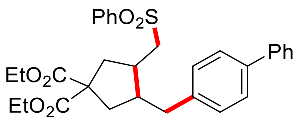

28: the title compound was prepared following the general procedure using $NiCl_2$•$6H_2O$ (4.8 mg, 0.02 mmol, 10 mol%), 4,4'-di-tert-butyl-2,2'-bipyridine (8.1 mg, 0.03 mmol, 15 mol%), 4-bromo-1,1'-biphenyl (46.6 mg, 0.2 mmol, 1 equiv.), [Ir(dtbbpy)(ppy)$_2$]PF_6 (1.8 mg, 0.002 mmol, 1 mol%), diethyl diallylmalonate (96.1 mg, 0.4 mmol, 2 equiv.) and $PhSO_2Na$ (65.7 mg, 0.4 mmol, 2 equiv.). The reaction mixture was stirred in MeCN (2 mL) and irradiated with a 10 W blue LED strip at room temperature for 24 h. The crude product was purified by flash column chromatography to afford 104 mg (97%, 88:12 d.r.) of **28** as a colorless oil.

^{1}H NMR (400 MHz, $CDCl_3$): δ 7.89 (d, *J* = 7.3 Hz, 2H), 7.62 (t, *J* = 7.5 Hz, 1H), 7.59 – 7.50 (m, 4H), 7.46 (d, *J* = 8.0 Hz, 2H), 7.41 (t, *J* = 7.7 Hz, 2H), 7.35 – 7.28 (m, 1H), 7.18 (d, *J* = 7.9 Hz, 2H), 4.20 (q, *J* = 7.1 Hz, 2H), 4.13 (qd, *J* = 7.0, 6.4, 1.5 Hz, 2H), 3.30 (dd, *J* = 14.0, 5.1 Hz, 1H), 3.19 (dd, *J* = 14.1, 8.0 Hz, 1H), 2.72 (dd, *J* = 13.3, 5.0 Hz, 1H), 2.64 – 2.48 (m, 3H), 2.41 – 2.30 (m, 2H), 2.24 (dd, *J* = 14.3, 6.7 Hz, 1H), 2.12 (dd, *J* = 14.2, 5.8 Hz, 1H), 1.24 (t, *J* = 7.1 Hz, 3H), 1.19 (t, *J* = 7.1 Hz, 3H).

^{13}C NMR (101 MHz, $CDCl_3$): δ 172.7, 172.2, 140.9, 139.6, 139.1, 139.0, 133.8, 129.4 (2C), 129.3, 128.8, 128.0, 127.2, 127.0, 61.8, 61.7, 58.3, 56.2, 43.7, 38.2, 37.7, 37.0, 34.3, 14.1 (2C).

IR (ATR) ν 2979, 1724, 1590, 1452, 1372, 1257, 1145, 1088, 1022, 858, 752, 690 cm^{-1}.

HRMS (ESI) for $C_{31}H_{34}O_6NaS^+$ $(M+Na)^+$: 557.1968; Found : 557.1964.

Diethyl 3-(4-acetamidobenzyl)-4-((phenylsulfonyl)methyl)cyclopentane-1,1-dicarboxylate, **29**: the

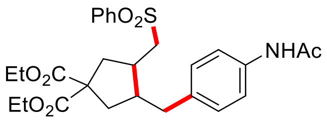

title compound was prepared following the general procedure using $NiCl_2$•$6H_2O$ (4.8 mg, 0.02 mmol, 10 mol%), 4,4'-di-tert-butyl-2,2'-bipyridine (8.1 mg, 0.03 mmol, 15 mol%), N-(4-bromophenyl)acetamide (42.8 mg, 0.2 mmol, 1 equiv.), [Ir(dtbbpy)(ppy)$_2$]PF_6 (1.8 mg, 0.002 mmol, 1 mol%), diethyl diallylmalonate (96.1 mg, 0.4 mmol, 2 equiv.) and $PhSO_2Na$ (65.7 mg, 0.4 mmol, 2 equiv.). The reaction mixture was stirred in MeCN (2 mL) and irradiated with a 10 W blue LED strip at room temperature for 24 h. The crude product was purified by flash column chromatography to afford 99 mg (97%, 86:14 d.r.) of **29** as a colorless oil.

^{1}H NMR (400 MHz, $CDCl_3$): δ 7.91 – 7.82 (m, 2H), 7.77 (s, 1H), 7.62 (t, *J* = 7.5 Hz, 1H), 7.56 – 7.48 (m, 2H), 7.36 (d, *J* = 8.1 Hz, 2H), 6.99 (d, *J* = 8.1 Hz, 2H), 4.18 – 4.12 (m, 2H), 4.12 – 4.06 (m, 2H), 3.24 (dd, *J* = 14.0, 5.1 Hz, 1H), 3.13 (dd, *J* = 14.0, 8.1 Hz, 1H), 2.64 – 2.56 (m, 1H), 2.56 – 2.44 (m, 2H), 2.40 (dt, *J* = 11.4, 5.8 Hz, 1H), 2.33 – 2.19 (m, 2H), 2.15 (dd, *J* = 14.4, 6.6 Hz, 1H), 2.09 (s, 3H), 2.01 (dd, *J* = 14.3, 5.9 Hz, 1H), 1.20 (d, *J* = 7.4 Hz, 3H), 1.17 – 1.13 (m, 3H).

^{13}C NMR (101 MHz, $CDCl_3$): δ 172.6, 172.2, 168.7, 139.5, 136.4, 135.6, 133.9, 129.5, 129.3, 128.0, 120.1, 61.8, 61.7, 58.2, 56.2, 43.7, 38.2, 37.6, 36.9, 34.1, 24.5, 14.1, 14.0.

IR (ATR) ν 3349, 2933, 2535, 2166, 2036, 1722, 1604, 1452, 1371, 1258, 1145, 1091, 1014, 858, 743 cm^{-1}.

HRMS (ESI) for $C_{27}H_{33}O_7NNaS^+$ $(M+Na)^+$: 538.1870; Found : 538.1870.

Diethyl 3-(3-(methoxycarbonyl)benzyl)-4-((phenylsulfonyl)methyl)cyclopentane-1,1-dicarboxylate,

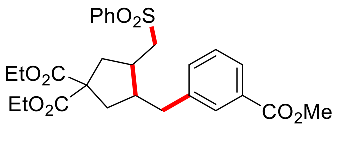

30: the title compound was prepared following the general procedure using $NiCl_2$•$6H_2O$ (4.8 mg, 0.02 mmol, 10 mol%), 4,4'-di-tert-butyl-2,2'-bipyridine (8.1 mg, 0.03 mmol, 15 mol%), methyl 3-bromobenzoate (43 mg, 0.2 mmol, 1 equiv.), [Ir(dtbbpy)(ppy)$_2$]PF_6 (1.8 mg, 0.002 mmol, 1 mol%), diethyl diallylmalonate (96.1 mg, 0.4 mmol, 2 equiv.) and $PhSO_2Na$ (65.7 mg, 0.4 mmol, 2 equiv.). The reaction mixture was stirred in MeCN (2 mL) and irradiated with a 10 W blue LED strip at room temperature for 24 h. The crude product was purified by flash column chromatography to afford 101 mg (98%, 83:17 d.r.) of **30** as a colorless oil.

^{1}H NMR (600 MHz, $CDCl_3$): δ 7.87 (d, *J* = 7.1 Hz, 2H), 7.82 (d, *J* = 6.9 Hz, 1H), 7.73 (s, 1H), 7.63 (t, *J* = 7.5 Hz, 1H), 7.53 (t, *J* = 7.8 Hz, 2H), 7.34 – 7.27 (m, 2H), 4.18 (qd, *J* = 7.1, 2.8 Hz, 2H), 4.14 – 4.08 (m, 2H), 3.87 (s, 3H), 3.26 (dd, *J* = 14.1, 5.5 Hz, 1H), 3.17 (dd, *J* = 14.1, 8.3 Hz, 1H), 2.71 (dd, *J* = 13.2,

4.8 Hz, 1H), 2.59 – 2.53 (m, 1H), 2.53 – 2.44 (m, 2H), 2.37 – 2.30 (m, 2H), 2.18 (dd, *J* = 14.3, 6.9 Hz, 1H), 2.01 (dd, *J* = 14.3, 5.6 Hz, 1H), 1.21 (t, *J* = 7.1 Hz, 3H), 1.17 (t, *J* = 7.1 Hz, 3H).

^{13}C NMR (151 MHz, $CDCl_3$): δ 172.7, 172.2, 167.1, 140.3, 139.6, 134.0, 133.7, 130.4, 130.0, 129.5, 128.7, 128.1, 127.7, 62.0, 61.8, 58.2, 56.2, 52.2, 43.7, 38.3, 37.5, 37.1, 34.5, 14.1 (2C).

IR (ATR) ν 2978, 1720, 1588, 1446, 1370, 1265, 1191, 1147, 1095, 988, 859, 746 cm^{-1}.

HRMS (ESI) for $C_{27}H_{32}O_8NaS^+$ $(M+Na)^+$: 539.1710; Found : 539.1710.

Diethyl 3-(3,5-dimethoxybenzyl)-4-((phenylsulfonyl)methyl)cyclopentane-1,1-dicarboxylate, **31**: the title compound was prepared following the general procedure using

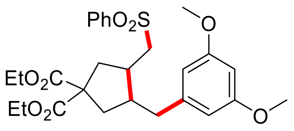

$NiCl_2$•$6H_2O$ (4.8 mg, 0.02 mmol, 10 mol%), 4,4'-di-tert-butyl-2,2'-bipyridine (8.1 mg, 0.03 mmol, 15 mol%), 1-bromo-3,5-dimethoxybenzene (43.4 mg, 0.2 mmol, 1 equiv.), [Ir(dtbbpy)(ppy)$_2$]PF_6 (1.8 mg, 0.002 mmol, 1 mol%), diethyl diallylmalonate (96.1 mg, 0.4 mmol, 2 equiv.) and $PhSO_2Na$ (65.7 mg, 0.4 mmol, 2 equiv.). The reaction mixture was stirred in MeCN (2 mL) and irradiated with a 10 W blue LED strip at room temperature for 24 h. The crude product was purified by flash column chromatography to afford 91.5 mg (88%, 86:14 d.r.) of **31** as a colorless oil.

^{1}H NMR (400 MHz, $CDCl_3$): δ 7.86 (d, *J* = 8.4 Hz, 2H), 7.66 – 7.58 (m, 1H), 7.53 (t, *J* = 7.7 Hz, 2H), 6.29 – 6.22 (m, 3H), 4.23 – 4.05 (m, 4H), 3.72 (d, *J* = 0.9 Hz, 6H), 3.25 (dd, *J* = 14.0, 5.0 Hz, 1H), 3.13 (dd, *J* = 14.0, 8.4 Hz, 1H), 2.63 – 2.40 (m, 4H), 2.31 (dd, *J* = 13.8, 8.1 Hz, 1H), 2.27 – 2.15 (m, 2H), 2.06 (dd, *J* = 14.2, 6.0 Hz, 1H), 1.21 (t, *J* = 6.0 Hz, 3H), 1.17 (t, *J* = 6.0 Hz, 3H).

^{13}C NMR (101 MHz, $CDCl_3$): δ 172.6, 172.2, 160.9, 142.2, 139.6, 133.8, 129.4, 128.0, 107.0, 98.2, 61.8, 61.7, 58.3, 56.2, 55.3, 43.5, 38.2, 37.7, 37.0, 35.1, 14.1 (2C).

IR (ATR) ν 2947, 1725, 1597, 1455, 1259, 1148, 1058, 932, 847, 742, 691 cm^{-1}.

HRMS (ESI) for $C_{27}H_{34}O_8NaS^+$ $(M+Na)^+$: 541.1867; Found : 541.1862.

Diethyl 3-(3,5-bis(trifluoromethyl)benzyl)-4-((phenylsulfonyl)methyl)cyclopentane-1,1-dicarboxylate, **32**: the title compound was prepared following the general procedure

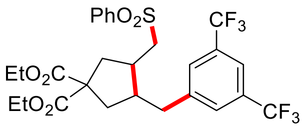

using $NiCl_2$•$6H_2O$ (4.8 mg, 0.02 mmol, 10 mol%), 4,4'-di-tert-butyl-2,2'-bipyridine (8.1 mg, 0.03 mmol, 15 mol%), 1-bromo-3,5-bis(trifluoromethyl)benzene (58.6 mg, 0.2 mmol, 1 equiv.), [Ir(dtbbpy)(ppy)$_2$]PF_6 (1.8 mg, 0.002 mmol, 1 mol%), diethyl diallylmalonate (96.1 mg, 0.4 mmol, 2 equiv.) and $PhSO_2Na$ (65.7 mg, 0.4 mmol, 2 equiv.). The reaction mixture was stirred in MeCN (2 mL) and irradiated with a 10 W blue LED strip at room temperature for 24 h. The crude product was purified by flash column chromatography to afford 83.3 mg (70%, 85:15 d.r.) of **32** as a colorless oil.

^{1}H NMR (400 MHz, $CDCl_3$): δ 7.98 – 7.87 (m, 2H), 7.73 – 7.62 (m, 2H), 7.62 –7.51 (m, 4H), 4.26 – 4.08 (m, 4H), 3.23 (qd, *J* = 14.1, 7.0 Hz, 2H), 2.87 (dd, *J* = 13.0, 3.9 Hz, 1H), 2.71 – 2.58 (m, 1H), 2.53

(dd, J = 13.8, 6.8 Hz, 2H), 2.39 (t, J = 12.4 Hz, 1H), 2.26 (dd, J = 13.9, 10.1 Hz, 1H), 2.18 (dd, J = 14.5, 6.8 Hz, 1H), 2.01 (dd, J = 14.5, 3.9 Hz, 1H), 1.21 (t, J = 6.0 Hz, 3H), 1.18 (t, J = 5.9 Hz, 3H).

^{13}C {^{19}F, ^{1}H} NMR (101 MHz, CDCl$_3$): δ 172.7, 172.0, 142.7, 139.6, 134.1, 131.7, 129.6, 129.4, 128.0, 123.4, 120.4, 62.1, 61.9, 57.9, 56.1, 43.3, 38.3, 37.5, 37.1, 33.8, 14.1 (2C).

^{19}F NMR (376 MHz, CDCl$_3$): *cis* isomer δ -62.83; *trans* isomer δ -62.86.

IR (ATR) ν 2982, 2175, 1726, 1453, 1376, 1270, 1128, 899, 854, 745, 688 cm^{-1}.

HRMS (ESI) for $C_{27}H_{28}O_6F_6NaS^+$ (M+Na)$^+$: 617.1403; Found : 617.1398.

Diethyl 3-(2-(methoxycarbonyl)benzyl)-4-((phenylsulfonyl)methyl)cyclopentane-1,1-dicarboxylate,

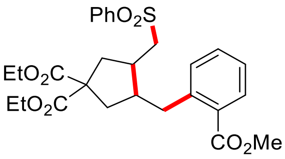

33: the title compound was prepared following the general procedure using NiCl$_2$•6H$_2$O (4.8 mg, 0.02 mmol, 10 mol%), 4,4'-di-tert-butyl-2,2'-bipyridine (8.1 mg, 0.03 mmol, 15 mol%), methyl 2-bromobenzoate (43 mg, 0.2 mmol, 1 equiv.), [Ir(dtbbpy)(ppy)$_2$]PF$_6$ (1.8 mg, 0.002 mmol, 1 mol%), diethyl diallylmalonate (96.1 mg, 0.4 mmol, 2 equiv.) and PhSO$_2$Na (65.7 mg, 0.4 mmol, 2 equiv.). The reaction mixture was stirred in MeCN (2 mL) and irradiated with a 10 W blue LED strip at room temperature for 24 h. The crude product was purified by flash column chromatography to afford 101 mg (98%, 89:11 d.r.) of **33** as a colorless oil.

^{1}H NMR (400 MHz, CDCl$_3$): δ 7.95 – 7.82 (m, 3H), 7.65 – 7.58 (m, 1H), 7.53 (t, J = 7.7 Hz, 2H), 7.36 (td, J = 7.5, 1.5 Hz, 1H), 7.27 – 7.18 (m, 2H), 4.19 – 4.05 (m, 4H), 3.80 (s, 3H), 3.47 (dd, J = 14.1, 2.6 Hz, 1H), 3.25 – 3.12 (m, 2H), 2.63 – 2.51 (m, 2H), 2.50 – 2.41 (m, 1H), 2.41 – 2.31 (m, 1H), 2.31 – 2.21 (m, 1H), 2.16 – 2.00 (m, 2H), 1.21 (t, J = 7.2 Hz, 3H), 1.15 (t, J = 7.1 Hz, 3H).

^{13}C NMR (101 MHz, CDCl$_3$): δ 172.7, 172.2, 167.6, 142.1, 139.7, 133.7, 132.1, 131.7, 131.2, 129.4, 129.2, 128.1, 126.4, 61.7, 61.6 (2C), 58.6, 56.7, 52.0, 44.2, 38.2, 37.3, 37.0, 33.0, 14.1, 14.0.

IR (ATR) ν 2981, 1721, 1446, 1368, 1299, 1254, 1144, 1086, 913, 859, 793, 734 cm^{-1}.

HRMS (ESI) for $C_{27}H_{32}O_8NaS^+$ (M+Na)$^+$: 539.1710; Found : 539.1705.

Diethyl 3-(2-methylbenzyl)-4-((phenylsulfonyl)methyl)cyclopentane-1,1-dicarboxylate, **34**: the title

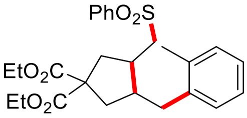

compound was prepared following the general procedure using NiCl$_2$•6H$_2$O (4.8 mg, 0.02 mmol, 10 mol%), 4,4'-di-tert-butyl-2,2'-bipyridine (8.1 mg, 0.03 mmol, 15 mol%), 1-bromo-2-methylbenzene (34.2 mg, 0.2 mmol, 1 equiv.), [Ir(dtbbpy)(ppy)$_2$]PF$_6$ (1.8 mg, 0.002 mmol, 1 mol%), diethyl diallylmalonate (96.1 mg, 0.4 mmol, 2 equiv.) and PhSO$_2$Na (65.7 mg, 0.4 mmol, 2 equiv.). The reaction mixture was stirred in MeCN (2 mL) and irradiated with a 10 W blue LED strip at room temperature for 24 h. The crude product was purified by flash column chromatography to afford 59.3 mg (63%, 87:13 d.r.) of **34** as a colorless oil.

^{1}H NMR (400 MHz, CDCl$_3$): δ 7.89 (d, J = 7.4 Hz, 2H), 7.64 (t, J = 7.3 Hz, 1H), 7.55 (t, J = 7.6 Hz, 2H), 7.12 – 6.99 (m, 4H), 4.21 – 4.14 (m, 2H), 4.12 (qd, J = 7.1, 1.3 Hz, 2H), 3.26 (dd, J = 14.0, 4.6 Hz, 1H), 3.18 (dd, J = 14.0, 9.1 Hz, 1H), 2.72 – 2.57 (m, 2H), 2.55 – 2.43 (m, 2H), 2.38 (dd, J = 14.1, 8.0 Hz,

1H), 2.33 – 2.17 (m, 5H), 2.02 (dd, J = 14.2, 6.8 Hz, 1H), 1.22 (t, J = 7.1 Hz, 3H), 1.18 (t, J = 7.1 Hz, 3H).

^{13}C NMR (101 MHz, CDCl$_3$): δ 172.7, 172.2, 139.7, 137.8, 136.2, 133.9, 130.5, 129.5 (2C), 128.1, 126.4, 126.0, 61.8, 61.7, 58.5, 56.3, 42.0, 38.3, 37.7, 37.0, 32.5, 19.6, 14.1 (2C).

IR (ATR) ν 2977, 1725, 1452, 1371, 1302, 1257, 1147, 1092, 1027, 913, 859, 739 cm^{-1}.

HRMS (ESI) for $C_{26}H_{32}O_6NaS^+$ (M+Na)$^+$: 495.1812; Found : 495.1806.

Diethyl 3-((2-methyl-1,3-dioxoisoindolin-5-yl)methyl)-4-((phenylsulfonyl)methyl)cyclopentane-1,1-dicarboxylate, **35**: the title compound was prepared following the general procedure using $NiCl_2$•$6H_2O$ (4.8 mg, 0.02 mmol, 10 mol%), 4,4'-di-tert-butyl-2,2'-bipyridine (8.1 mg, 0.03 mmol, 15 mol%), 5-bromo-2-methylisoindoline-1,3-dione (48.0 mg, 0.2 mmol, 1 equiv.), [Ir(dtbbpy)(ppy)$_2$]PF$_6$ (1.8 mg, 0.002 mmol, 1 mol%), diethyl diallylmalonate (96.1 mg, 0.4 mmol, 2 equiv.) and $PhSO_2Na$ (65.7 mg, 0.4 mmol, 2 equiv.). The reaction mixture was stirred in MeCN (2 mL) and irradiated with a 10 W blue LED strip at room temperature for 24 h. The crude product was purified by flash column chromatography to afford 105 mg (97%, 89:11 d.r.) of **35** as a colorless oil.

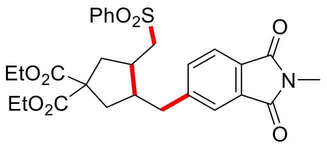

^{1}H NMR (600 MHz, CDCl$_3$): δ 7.89 (d, J = 7.1 Hz, 2H), 7.69 (d, J = 7.6 Hz, 1H), 7.67 – 7.62 (m, 1H), 7.60 – 7.52 (m, 3H), 7.50 (dd, J = 7.7, 1.5 Hz, 1H), 4.18 (q, J = 7.1 Hz, 2H), 4.14 – 4.07 (m, 2H), 3.25 (dd, J = 14.1, 6.8 Hz, 1H), 3.19 (dd, J = 14.1, 7.2 Hz, 1H), 3.12 (s, 3H), 2.88 (dd, J = 13.0, 4.2 Hz, 1H), 2.65 – 2.57 (m, 1H), 2.57 – 2.49 (m, 2H), 2.41 (dd, J = 13.0, 11.5 Hz, 1H), 2.23 (dd, J = 14.0, 9.6 Hz, 1H), 2.12 (dd, J = 14.3, 6.7 Hz, 1H), 2.03 (dd, J = 14.4, 4.5 Hz, 1H), 1.21 (t, J = 7.1 Hz, 3H), 1.16 (t, J = 7.1 Hz, 3H).

^{13}C NMR (151 MHz, CDCl$_3$): δ 172.6, 171.9, 168.5, 168.4, 147.4, 139.4, 134.7, 134.0, 132.7, 130.3, 129.5, 129.5, 128.0, 127.9, 123.7, 123.3, 62.0, 61.8, 57.9, 56.0, 43.5, 38.2, 37.3, 37.2, 34.5, 23.9, 14.1, 14.0.

IR (ATR) ν 2981, 2938, 1767, 1709, 1620, 1443, 1378, 1301, 1255, 1145, 1092, 1007, 913, 858, 789, 731 cm^{-1}. HRMS (ESI) for $C_{28}H_{31}O_8NNaS^+$ (M+Na)$^+$: 564.1663; Found : 564.1662.

Diethyl 3-((1-oxo-2,3-dihydro-1H-inden-5-yl)methyl)-4-((phenylsulfonyl)methyl)cyclopentane-1,1-dicarboxylate, **36**: the title compound was prepared following the general procedure using $NiCl_2$•$6H_2O$ (4.8 mg, 0.02 mmol, 10 mol%), 4,4'-di-tert-butyl-2,2'-bipyridine (8.1 mg, 0.03 mmol, 15 mol%), 5-bromo-2,3-dihydro-1H-inden-1-one (42.2 mg, 0.2 mmol, 1 equiv.), [Ir(dtbbpy)(ppy)$_2$]PF$_6$ (1.8 mg, 0.002 mmol, 1 mol%), diethyl diallylmalonate (96.1 mg, 0.4 mmol, 2 equiv.) and $PhSO_2Na$ (65.7 mg, 0.4 mmol, 2 equiv.). The reaction mixture was stirred in MeCN (2 mL) and irradiated with a 10 W blue LED strip at room temperature for 24 h. The crude product was purified by flash column chromatography to afford 88.3 mg (86%, 91:9 d.r.) of **36** as a colorless oil.

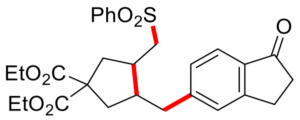

^{1}H NMR (600 MHz, CDCl$_3$): δ 7.93 – 7.87 (m, 2H), 7.68 – 7.62 (m, 1H), 7.62 – 7.53 (m, 3H), 7.41 – 7.35 (m, 1H), 7.30 – 7.24 (m, 1H), 4.17 (qd, *J* = 7.1, 1.8 Hz, 2H), 4.14 – 4.09 (m, 3H), 3.28 (dd, *J* = 14.1, 6.0 Hz, 1H), 3.21 (dd, *J* = 14.1, 7.6 Hz, 1H), 3.10 – 2.99 (m, 2H), 2.83 (dd, *J* = 13.5, 4.3 Hz, 1H), 2.70 – 2.59 (m, 4H), 2.51 (dd, *J* = 14.0, 6.7 Hz, 1H), 2.38 (dd, *J* = 13.5, 11.2 Hz, 1H), 2.30 (dd, *J* = 14.0, 9.0 Hz, 1H), 2.22 (dd, *J* = 14.4, 7.0 Hz, 1H), 2.06 (dd, *J* = 14.4, 5.3 Hz, 1H), 1.21 (t, *J* = 7.2 Hz, 3H), 1.17 (t, *J* = 7.2 Hz, 3H).

^{13}C NMR (151 MHz, CDCl$_3$): δ 207.3, 172.6, 172.0, 154.0, 139.5, 137.7, 137.4, 135.1, 134.0, 129.5, 129.4, 128.0, 127.7, 121.8, 61.9, 61.8, 58.1, 56.0, 41.4, 38.3, 37.5, 37.2, 36.2, 31.0, 24.7, 14.1, 14.0.

IR (ATR) ν 2978, 2933, 2178, 1714, 1591, 1447, 1370, 1257, 1146, 1089, 1025, 859, 781, 746 cm^{-1}.

HRMS (ESI) for $C_{28}H_{32}O_7NaS^+$ $(M+Na)^+$: 535.1761; Found : 535.1756.

Diethyl 3-((6-methoxynaphthalen-2-yl)methyl)-4-((phenylsulfonyl)methyl)cyclopentane-1,1-dicarboxylate, **37**: the title compound was prepared following the general procedure using NiCl$_2$•6H$_2$O (4.8 mg, 0.02 mmol, 10 mol%), 4,4'-di-tert-butyl-2,2'-bipyridine (8.1 mg, 0.03 mmol, 15 mol%), 2-bromo-6-methoxynaphthalene (65.7 mg, 0.2 mmol, 1 equiv.), [Ir(dtbbpy)(ppy)$_2$]PF$_6$ (1.8 mg, 0.002 mmol, 1 mol%), diethyl diallylmalonate (96.1 mg, 0.4 mmol, 2 equiv.) and PhSO$_2$Na (65.7 mg, 0.4 mmol, 2 equiv.). The reaction mixture was stirred in MeCN (2 mL) and irradiated with a 10 W blue LED strip at room temperature for 24 h. The crude product was purified by flash column chromatography to afford 105 mg (97%, 88:12 d.r.) of **37** as a colorless oil.

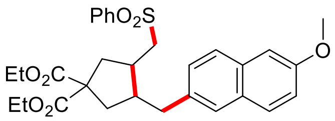

^{1}H NMR (600 MHz, CDCl$_3$): δ 7.85 (d, *J* = 7.7 Hz, 2H), 7.68 – 7.60 (m, 2H), 7.58 (t, *J* = 7.4 Hz, 1H), 7.51 – 7.41 (m, 3H), 7.21 (dd, *J* = 8.4, 1.8 Hz, 1H), 7.16 – 7.06 (m, 2H), 4.21 (qd, *J* = 7.2, 2.6 Hz, 2H), 4.16 – 4.10 (m, 2H), 3.90 (s, 3H), 3.33 (dd, *J* = 14.0, 4.1 Hz, 1H), 3.24 – 3.15 (m, 1H), 2.79 (dd, *J* = 13.3, 4.6 Hz, 1H), 2.63 – 2.50 (m, 3H), 2.50 – 2.37 (m, 2H), 2.22 (dd, *J* = 14.4, 6.1 Hz, 1H), 2.12 (dd, *J* = 14.3, 5.3 Hz, 1H), 1.24 (t, *J* = 7.2 Hz, 3H), 1.19 (t, *J* = 7.1 Hz, 3H).

^{13}C NMR (151 MHz, CDCl$_3$): δ 172.7, 172.2, 157.3, 139.4, 134.9, 133.7, 133.2, 129.3, 129.0 (2C), 127.9, 127.8, 127.1, 127.0, 118.8, 105.6, 61.8, 61.7, 58.2, 56.1, 55.4, 43.6, 38.2, 37.7, 36.9, 34.6, 14.1, 14.0.

IR (ATR) ν 2973, 1725, 1606, 1456, 1379, 1255, 1148, 1024, 855, 745 cm^{-1}.

HRMS (ESI) for $C_{30}H_{34}O_7NaS^+$ $(M+Na)^+$: 561.1918; Found : 561.1918.

Diethyl 3-((2-methylpyridin-4-yl)methyl)-4-((phenylsulfonyl)methyl)cyclopentane-1,1-dicarboxylate, **38**: the title compound was prepared following the general procedure using NiCl$_2$•6H$_2$O (4.8 mg, 0.02 mmol, 10 mol%), 4,4'-di-tert-butyl-2,2'-bipyridine (8.1 mg, 0.03 mmol, 15 mol%), 4-bromo-2-methylpyridine (34.4 mg, 0.2 mmol, 1 equiv.), [Ir(dtbbpy)(ppy)$_2$]PF$_6$ (1.8 mg, 0.002 mmol, 1 mol%), diethyl diallylmalonate (96.1 mg, 0.4 mmol, 2 equiv.) and PhSO$_2$Na (65.7 mg, 0.4 mmol, 2 equiv.). The reaction mixture was stirred in

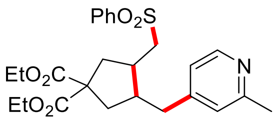

MeCN (2 mL) and irradiated with a 10 W blue LED strip at room temperature for 24 h. The crude product was purified by flash column chromatography to afford 80.1 mg (85%, 90:10 d.r.) of **38** as a colorless oil.

^{1}H NMR (600 MHz, $CDCl_3$): δ 8.32 (d, *J* = 5.2 Hz, 1H), 7.92 – 7.85 (m, 2H), 7.68 – 7.61 (m, 1H), 7.54 (t, *J* = 7.8 Hz, 2H), 6.92 (s, 1H), 6.86 (dd, *J* = 5.2, 1.6 Hz, 1H), 4.17 (q, *J* = 7.1 Hz, 2H), 4.11 (qd, *J* = 7.1, 2.7 Hz, 2H), 3.21 (dd, *J* = 14.1, 6.3 Hz, 1H), 3.15 (dd, *J* = 14.1, 7.7 Hz, 1H), 2.66 (dd, *J* = 13.1, 4.7 Hz, 1H), 2.61 – 2.54 (m, 1H), 2.53 – 2.45 (m, 5H), 2.28 – 2.19 (m, 2H), 2.16 (dd, *J* = 14.3, 7.0 Hz, 1H), 2.03 (dd, *J* = 14.3, 5.3 Hz, 1H), 1.21 (t, *J* = 7.1 Hz, 3H), 1.17 (t, *J* = 7.1 Hz, 3H).

^{13}C NMR (151 MHz, $CDCl_3$): δ 172.6, 172.0, 158.4, 149.3, 149.2, 139.5, 133.9, 129.5, 127.9, 123.9, 121.5, 61.9, 61.8, 58.0, 56.0, 42.8, 38.2, 37.5, 37.1, 33.8, 24.4, 14.1, 14.0.

IR (ATR) ν 2979, 2499, 1723, 1606, 1560, 1448, 1370, 1257, 1147, 1092, 1012, 933, 859, 747 cm^{-1}.

HRMS (ESI) for $C_{25}H_{32}O_6NS^+$ $(M+H)^+$: 474.1945; Found : 474.1945.

Diethyl 3-((phenylsulfonyl)methyl)-4-(pyrimidin-5-ylmethyl)cyclopentane-1,1-dicarboxylate, **39**:

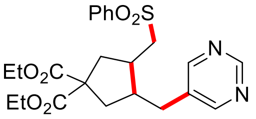

the title compound was prepared following the general procedure using $NiCl_2•6H_2O$ (4.8 mg, 0.02 mmol, 10 mol%), 4,4'-di-tert-butyl-2,2'-bipyridine (8.1 mg, 0.03 mmol, 15 mol%), 5-bromopyrimidine (31.8 mg, 0.2 mmol, 1 equiv.), $[Ir(dtbbpy)(ppy)_2]PF_6$ (1.8 mg, 0.002 mmol, 1 mol%), diethyl diallylmalonate (96.1 mg, 0.4 mmol, 2 equiv.) and $PhSO_2Na$ (65.7 mg, 0.4 mmol, 2 equiv.). The reaction mixture was stirred in MeCN (2 mL) and irradiated with a 10 W blue LED strip at room temperature for 24 h. The crude product was purified by flash column chromatography to afford 64.3 mg (70%, 92:8 d.r.) of **39** as a white solid.

^{1}H NMR (600 MHz, $CDCl_3$): δ 9.06 (s, 1H), 8.58 (s, 2H), 7.94 – 7.89 (m, 2H), 7.66 (t, *J* = 7.5 Hz, 1H), 7.57 (t, *J* = 7.8 Hz, 2H), 4.18 (qd, *J* = 7.1, 1.6 Hz, 2H), 4.12 (qd, *J* = 7.2, 4.9 Hz, 2H), 3.26 (dd, *J* = 14.1, 7.3 Hz, 1H), 3.21 (dd, *J* = 14.1, 6.9 Hz, 1H), 2.79 (dd, *J* = 13.4, 3.9 Hz, 1H), 2.68 – 2.60 (m, 1H), 2.56 (dd, *J* = 13.9, 6.9 Hz, 1H), 2.54 – 2.48 (m, 1H), 2.28 – 2.21 (m, 1H), 2.20 – 2.10 (m, 2H), 2.07 (dd, *J* = 14.5, 4.2 Hz, 1H), 1.21 (t, *J* = 7.1 Hz, 3H), 1.17 (t, *J* = 7.1 Hz, 3H).

^{13}C NMR (151 MHz, $CDCl_3$): δ 172.6, 171.8, 157.4, 157.1, 139.5, 134.1, 133.4, 129.6, 128.0, 62.1, 61.9, 57.8, 56.0, 43.1, 38.2, 37.5, 37.1, 28.9, 14.1, 14.0.

IR (ATR) ν 2971, 1722, 1562, 1447, 1412, 1266, 1197, 1129, 1088, 1017, 926, 847, 796, 729 cm^{-1}.

HRMS (ESI) for $C_{23}H_{28}O_6N_2NaS^+$ $(M+Na)^+$: 483.1560; Found : 483.1550.

Diethyl 3-((phenylsulfonyl)methyl)-4-(quinolin-3-ylmethyl)cyclopentane-1,1-dicarboxylate, **40**: the

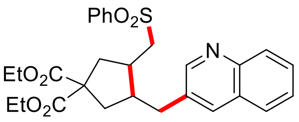

title compound was prepared following the general procedure using $NiCl_2•6H_2O$ (4.8 mg, 0.02 mmol, 10 mol%), 4,4'-di-tert-butyl-2,2'-bipyridine (8.1 mg, 0.03 mmol, 15 mol%), 3-bromoquinoline (65.7 mg, 0.2 mmol, 1 equiv.), $[Ir(dtbbpy)(ppy)_2]PF_6$ (1.8 mg, 0.002 mmol, 1 mol%), diethyl diallylmalonate (96.1 mg, 0.4 mmol, 2 equiv.) and $PhSO_2Na$ (65.7 mg, 0.4 mmol, 2 equiv.). The reaction mixture was stirred in MeCN

(2 mL) and irradiated with a 10 W blue LED strip at room temperature for 24 h. The crude product was purified by flash column chromatography to afford 82.8 mg (81%, 85:15 d.r.) of **40** as a colorless oil.

^{1}H NMR (400 MHz, CDCl$_3$): δ 8.67 (d, *J* = 2.2 Hz, 1H), 8.04 (d, *J* = 8.5 Hz, 1H), 7.93 – 7.83 (m, 3H), 7.72 (d, *J* = 7.9 Hz, 1H), 7.66 – 7.60 (m, 1H), 7.60 – 7.54 (m, 1H), 7.53 – 7.44 (m, 3H), 4.19 (q, *J* = 7.1 Hz, 2H), 4.10 (qd, *J* = 7.0, 2.9 Hz, 2H), 3.30 (dd, *J* = 14.1, 5.3 Hz, 1H), 3.21 (dd, *J* = 14.1, 7.0 Hz, 1H), 2.89 (dd, *J* = 13.3, 3.9 Hz, 1H), 2.68 – 2.52 (m, 3H), 2.46 (dd, *J* = 13.2, 10.6 Hz, 1H), 2.31 (dd, *J* = 13.7, 8.2 Hz, 1H), 2.18 (dd, *J* = 14.4, 6.3 Hz, 1H), 2.09 (dd, *J* = 14.4, 4.8 Hz, 1H), 1.21 (t, *J* = 7.0 Hz, 3H), 1.16 (t, *J* = 7.1 Hz, 3H).

^{13}C NMR (101 MHz, CDCl$_3$): δ 172.6, 172.0, 152.0, 147.0, 139.5, 135.3, 133.9, 132.6, 129.5, 129.2, 129.0, 128.1, 128.0, 127.5, 126.8, 62.0, 61.8, 58.1, 56.1, 43.4, 38.3, 37.5, 37.2, 31.9, 14.1, 14.0.

IR (ATR) ν 2981, 2935, 1723, 1494, 1447, 1369, 1301, 1256, 1145, 1090, 1026, 913, 859, 787, 744 cm^{-1}.

HRMS (ESI) for $C_{28}H_{32}O_6NS^+$ $(M+H)^+$: 510.1945; Found : 510.1942.

Diethyl 3-((2-methylquinolin-6-yl)methyl)-4-((phenylsulfonyl)methyl)cyclopentane-1,1-dicarboxylate, **41**: the title compound was prepared following the general procedure using $NiCl_2$•$6H_2O$ (4.8 mg, 0.02 mmol, 10 mol%), 4,4'-di-tert-butyl-2,2'-bipyridine (8.1 mg, 0.03 mmol, 15 mol%), 6-bromo-2-methylquinoline (65.7 mg, 0.2 mmol, 1 equiv.), [Ir(dtbbpy)(ppy)$_2$]PF_6 (1.8 mg, 0.002 mmol, 1 mol%), diethyl diallylmalonate (96.1 mg, 0.4 mmol, 2 equiv.) and $PhSO_2Na$ (65.7 mg, 0.4 mmol, 2 equiv.). The reaction mixture was stirred in MeCN (2 mL) and irradiated with a 10 W blue LED strip at room temperature for 24 h. The crude product was purified by flash column chromatography to afford 87.6 mg (84%, 83:17 d.r.) of **41** as a colorless oil.

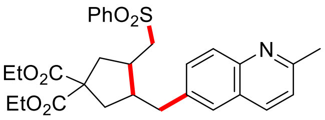

^{1}H NMR (600 MHz, CDCl$_3$): δ 7.93 (d, *J* = 8.3 Hz, 1H), 7.90 – 7.80 (m, 3H), 7.57 (t, *J* = 7.5 Hz, 1H), 7.51 – 7.42 (m, 4H), 7.23 (d, *J* = 8.5 Hz, 1H), 4.19 (q, *J* = 7.1 Hz, 2H), 4.11 (qd, J = 7.1, 5.1 Hz, 2H), 3.30 (dd, *J* = 14.1, 5.0 Hz, 1H), 3.20 (dd, *J* = 14.0, 7.3 Hz, 1H), 2.85 (dd, *J* = 13.2, 4.3 Hz, 1H), 2.70 (s, 3H), 2.63 – 2.51 (m, 3H), 2.46 (dd, *J* = 13.3, 10.1 Hz, 1H), 2.33 (dd, *J* = 13.5, 7.5 Hz, 1H), 2.18 (dd, *J* = 14.5, 6.3 Hz, 1H), 2.12 (dd, *J* = 14.3, 5.2 Hz, 1H), 1.21 (t, *J* = 7.1 Hz, 3H), 1.16 (t, *J* = 7.1 Hz, 3H).

^{13}C NMR (151 MHz, CDCl$_3$): δ 172.7, 172.2, 158.5, 146.8, 139.4, 137.3, 135.8, 133.8, 130.9, 129.4, 128.8, 127.9, 126.9, 126.5, 122.1, 61.8, 61.7, 58.1, 56.0, 43.5, 38.2, 37.6, 37.1, 34.4, 25.4, 14.1, 14.0.

IR (ATR) ν 2980, 2932, 1724, 1600, 1494, 1448, 1371, 1303, 1257, 1146, 1090, 1026, 909, 829, 735 cm^{-1}.

HRMS (ESI) for $C_{29}H_{34}O_6NS^+$ $(M+H)^+$: 524.2101; Found : 524.2088.

Diethyl 3-((1-(tert-butoxycarbonyl)-1H-indol-5-yl)methyl)-4-((phenylsulfonyl)methyl)cyclopentane-1,1-dicarboxylate, **42**: the title compound was prepared following the general procedure using $NiCl_2$•$6H_2O$ (4.8 mg, 0.02 mmol, 10 mol%), 4,4'-di-tert-butyl-2,2'-bipyridine (8.1 mg, 0.03 mmol, 15 mol%), tert-butyl 5-

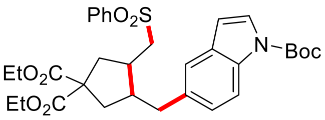

bromo-1H-indole-1-carboxylate (59.2 mg, 0.2 mmol, 1 equiv.), [Ir(dtbbpy)(ppy)$_2$]PF$_6$ (1.8 mg, 0.002 mmol, 1 mol%), diethyl diallylmalonate (96.1 mg, 0.4 mmol, 2 equiv.) and $PhSO_2Na$ (65.7 mg, 0.4 mmol, 2 equiv.). The reaction mixture was stirred in MeCN (2 mL) and irradiated with a 10 W blue LED strip at room temperature for 24 h. The crude product was purified by flash column chromatography to afford 108 mg (90%, 82:18 d.r.) of **42** as a colorless oil.

^{1}H NMR (400 MHz, CDCl$_3$): δ 7.96 (d, *J* = 8.6 Hz, 1H), 7.89 – 7.79 (m, 2H), 7.62 – 7.52 (m, 2H), 7.48 (t, *J* = 7.7 Hz, 2H), 7.26 (s, 1H), 7.02 (dd, *J* = 8.5, 1.7 Hz, 1H), 6.48 – 6.42 (m, 1H), 4.19 (q, *J* = 7.1 Hz, 2H), 4.11 (qd, *J* = 7.1, 2.1 Hz, 2H), 3.29 (dd, *J* = 14.1, 4.4 Hz, 1H), 3.17 (dd, *J* = 14.1, 7.7 Hz, 1H), 2.74 (dd, *J* = 13.1, 4.5 Hz, 1H), 2.60 – 2.46 (m, 3H), 2.44 – 2.32 (m, 2H), 2.18 (dd, *J* = 14.1, 6.3 Hz, 1H), 2.09 (dd, *J* = 14.2, 5.6 Hz, 1H), 1.64 (s, 9H), 1.22 (t, *J* = 7.1 Hz, 3H), 1.17 (t, *J* = 7.1 Hz, 3H).

^{13}C NMR (101 MHz, CDCl$_3$): δ 172.7, 172.3, 149.8, 139.6, 134.1, 133.7, 130.9, 129.3, 128.0, 126.2, 125.3, 121.0, 115.1, 107.2, 83.7, 61.8, 61.7, 58.3, 56.2, 44.1, 38.3, 37.7, 37.0, 34.6, 28.3, 14.1 (2C).

IR (ATR) ν 2979, 1725, 1460, 1368, 1310, 1256, 1145, 1087, 1023, 910, 853, 734 cm^{-1}.

HRMS (ESI) for $C_{32}H_{39}O_8NNaS^+$ (M+Na)$^+$: 620.2289; Found : 620.2294.

Diethyl 3-((phenylsulfonyl)methyl)-4-((1-tosyl-1H-pyrrolo[2,3-b]pyridin-4-yl)methyl)cyclopentane - 1,1-dicarboxylate, **43**: the title compound was prepared following the general procedure using $NiCl_2•6H_2O$ (4.8 mg, 0.02 mmol, 10 mol%), 4,4'-di-tert-butyl-2,2'-bipyridine (8.1 mg, 0.03 mmol, 15 mol%), 4-bromo-1-tosyl-1H-pyrrolo[2,3-b]pyridine (70.2 mg, 0.2 mmol, 1 equiv.), [Ir(dtbbpy)(ppy)$_2$]PF$_6$ (1.8 mg, 0.002 mmol, 1 mol%), diethyl diallylmalonate (96.1 mg, 0.4 mmol, 2 equiv.) and $PhSO_2Na$ (65.7 mg, 0.4 mmol, 2 equiv.). The reaction mixture was stirred in MeCN (2 mL) and irradiated with a 10 W blue LED strip at room temperature for 24 h. The crude product was purified by flash column chromatography to afford 121 mg (93%, 83:17 d.r.) of **43** as a white foam.

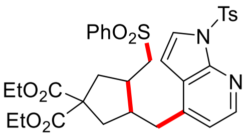

^{1}H NMR (400 MHz, CDCl$_3$): δ 8.25 (d, *J* = 5.0 Hz, 1H), 8.01 (d, *J* = 8.2 Hz, 2H), 7.88 – 7.80 (m, 2H), 7.66 – 7.57 (m, 2H), 7.49 (t, *J* = 7.7 Hz, 2H), 7.21 (d, *J* = 8.1 Hz, 2H), 6.96 (d, *J* = 5.0 Hz, 1H), 6.80 (d, *J* = 4.0 Hz, 1H), 4.14 (tt, *J* = 7.2, 3.5 Hz, 2H), 4.07 (q, *J* = 7.0 Hz, 2H), 3.26 (dd, *J* = 14.1, 7.4 Hz, 1H), 3.18 (dd, *J* = 14.1, 6.5 Hz, 1H), 3.00 (dd, *J* = 13.0, 4.2 Hz, 1H), 2.73 – 2.63 (m, 1H), 2.59 (dt, *J* = 9.7, 6.4 Hz, 1H), 2.50 – 2.38 (m, 2H), 2.30 (s, 3H), 2.25 (dd, *J* = 13.9, 10.1 Hz, 1H), 2.15 – 2.07 (m, 1H), 1.98 (dd, *J* = 14.5, 4.3 Hz, 1H), 1.18 (d, *J* = 7.0 Hz, 3H), 1.13 (d, *J* = 7.3 Hz, 3H).

^{13}C NMR (101 MHz, CDCl$_3$): δ 172.5, 171.9, 147.3, 145.1, 144.8, 142.7, 139.4, 135.5, 134.0, 129.6, 129.5, 128.0, 127.8, 125.8, 122.7, 119.6, 104.0, 61.9, 61.7, 57.8, 55.8, 41.7, 38.2, 37.5, 37.3, 31.4, 21.6, 14.0 (2C).

IR (ATR) ν 2932, 1724, 1590, 1518, 1450, 1368, 1257, 1145, 1087, 1025, 858, 809, 733 cm^{-1}.

HRMS (ESI) for $C_{33}H_{36}O_8N_2NaS_2^+$ (M+Na)$^+$: 675.1805; Found : 675.1803.

Diethyl 3-(benzo[b]thiophen-3-ylmethyl)-4-((phenylsulfonyl)methyl)cyclopentane-1,1-dicarboxylate, 44: the title compound was prepared following the general procedure using $NiCl_2$•$6H_2O$ (4.8 mg, 0.02 mmol, 10 mol%), 4,4'-di-tert-butyl-2,2'-bipyridine (8.1 mg, 0.03 mmol, 15 mol%), 3-bromobenzo[b]thiophene (42.6 mg, 0.2 mmol, 1 equiv.), [Ir(dtbbpy)(ppy)2]PF_6 (1.8 mg, 0.002 mmol, 1 mol%), diethyl diallylmalonate (96.1 mg, 0.4 mmol, 2 equiv.) and $PhSO_2Na$ (65.7 mg, 0.4 mmol, 2 equiv.). The reaction mixture was stirred in MeCN (2 mL) and irradiated with a 10 W blue LED strip at room temperature for 24 h. The crude product was purified by flash column chromatography to afford 93.1 mg (90%, 89:11 d.r.) of **44** as a white solid.

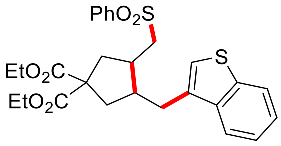

^1H NMR (600 MHz, $CDCl_3$): δ 7.92 – 7.88 (m, 2H), 7.86 – 7.80 (m, 2H), 7.65 (t, J = 7.5 Hz, 1H), 7.55 (t, J = 7.8 Hz, 2H), 7.39 – 7.35 (m, 1H), 7.35 – 7.31 (m, 1H), 7.12 (s, 1H), 4.21 – 4.16 (m, 2H), 4.14 (qd, J = 7.3, 3.0 Hz, 2H), 3.35 (dd, J = 14.1, 6.0 Hz, 1H), 3.23 (dd, J = 14.1, 7.9 Hz, 1H), 2.95 (dd, J = 14.0, 4.9 Hz, 1H), 2.76 – 2.70 (m, 1H), 2.66 (td, J = 8.3, 6.3 Hz, 1H), 2.59 – 2.53 (m, 2H), 2.35 (dd, J = 14.0, 9.2 Hz, 1H), 2.26 (dd, J = 14.4, 7.0 Hz, 1H), 2.18 (dd, J = 14.3, 5.3 Hz, 1H), 1.22 (t, J = 7.1 Hz, 3H), 1.19 (t, J = 7.1 Hz, 3H).

^{13}C NMR (151 MHz, $CDCl_3$): δ 172.7, 172.1, 140.5, 139.5, 138.8, 133.9, 133.9, 129.5, 128.0, 124.3, 124.1, 123.0, 122.9, 121.9, 61.9, 61.7, 58.2, 56.1, 41.2, 38.2, 38.0, 37.0, 27.7, 14.1 (2C).

IR (ATR) ν 2978, 1724, 1448, 1368, 1300, 1256, 1145, 1086, 1022, 857, 746 cm^{-1}.

HRMS (ESI) for $C_{27}H_{30}O_6NaS_2^+$ $(M+Na)^+$: 537.1376; Found : 537.1376.

Diethyl 3-(naphthalen-2-ylmethyl)-4-((phenylsulfonyl)methyl)cyclopentane-1,1-dicarboxylate, 45: the title compound was prepared following the general procedure using $NiCl_2$•$6H_2O$ (4.8 mg, 0.02 mmol, 10 mol%), 4,4'-di-tert-butyl-2,2'-bipyridine (8.1 mg, 0.03 mmol, 15 mol%), 2-chloronaphthalene (32.5 mg, 0.2 mmol, 1 equiv.), $[Ir(dtbbpy)(ppy)_2]PF_6$ (1.8 mg, 0.002 mmol, 1 mol%), diethyl diallylmalonate (96.1 mg, 0.4 mmol, 2 equiv.) and $PhSO_2Na$ (65.7 mg, 0.4 mmol, 2 equiv.). The reaction mixture was stirred in MeCN (2 mL) and irradiated with a 10 W blue LED strip at room temperature for 24 h. The crude product was purified by flash column chromatography to afford 78 mg (77%, 85:15 d.r.) of **45** as a colorless oil.

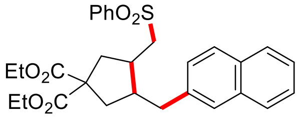

^1H NMR (400 MHz, $CDCl_3$): δ 7.87 – 7.80 (m, 2H), 7.77 (dd, J = 8.0, 1.6 Hz, 1H), 7.74 – 7.69 (m, 2H), 7.58 – 7.50 (m, 2H), 7.47 – 7.39 (m, 4H), 7.24 (dd, J = 8.4, 1.8 Hz, 1H), 4.21 (qd, J = 7.1, 1.5 Hz, 2H), 4.16 – 4.09 (m, 2H), 3.32 (dd, J = 14.1, 4.7 Hz, 1H), 3.19 (dd, J = 14.0, 7.7 Hz, 1H), 2.82 (dd, J = 13.0, 4.6 Hz, 1H), 2.63 – 2.37 (m, 5H), 2.23 (dd, J = 14.4, 6.4 Hz, 1H), 2.11 (dd, J = 14.2, 5.8 Hz, 1H), 1.23 (t, J = 7.1 Hz, 3H), 1.18 (t, J = 7.1 Hz, 3H).

^{13}C NMR (101 MHz, $CDCl_3$): δ 172.7, 172.3, 139.5, 137.3, 133.7, 133.6, 132.2, 129.3, 128.2, 128.0, 127.7, 127.5, 127.3 (2C), 126.1, 125.5, 61.9, 61.7, 58.3, 56.1, 43.5, 38.2, 37.8, 37.0, 34.9, 14.1 (2C).

IR (ATR) ν 2981, 2934, 1724, 1447, 1368, 1302, 1256, 1146, 1090, 1027, 910, 859, 812, 735 cm^{-1}.

HRMS (ESI) for $C_{29}H_{32}O_6NaS^+$ $(M+Na)^+$: 531.1912; Found : 531.1812.

Diethyl 3-((phenylsulfonyl)methyl)-4-(quinolin-6-ylmethyl)cyclopentane-1,1-dicarboxylate, **46**: the

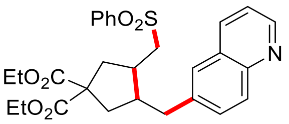

title compound was prepared following the general procedure using $NiCl_2$•$6H_2O$ (4.8 mg, 0.02 mmol, 10 mol%), 4,4'-di-tert-butyl-2,2'-bipyridine (8.1 mg, 0.03 mmol, 15 mol%), 6-chloroquinoline (32.7 mg, 0.2 mmol, 1 equiv.), $[Ir(dtbbpy)(ppy)_2]PF_6$ (1.8 mg, 0.002 mmol, 1 mol%), diethyl diallylmalonate (96.1 mg, 0.4 mmol, 2 equiv.) and $PhSO_2Na$ (65.7 mg, 0.4 mmol, 2 equiv.). The reaction mixture was stirred in MeCN (2 mL) and irradiated with a 10 W blue LED strip at room temperature for 24 h. The crude product was purified by flash column chromatography to afford 92.1 mg (90%, 87:13 d.r.) of **46** as a colorless oil.

^{1}H NMR (400 MHz, $CDCl_3$): δ 8.83 (dd, *J* = 4.3, 1.7 Hz, 1H), 8.03 (d, *J* = 8.1 Hz, 1H), 7.95 (d, *J* = 8.6 Hz, 1H), 7.83 (d, *J* = 7.2 Hz, 2H), 7.58 – 7.51 (m, 2H), 7.51 – 7.41 (m, 3H), 7.36 – 7.31 (m, 1H), 4.18 (q, *J* = 7.1 Hz, 2H), 4.14 – 4.06 (m, 2H), 3.29 (dd, *J* = 13.9, 4.9 Hz, 1H), 3.19 (dd, *J* = 13.9, 7.0 Hz, 1H), 2.87 (dd, *J* = 13.0, 4.1 Hz, 1H), 2.64 – 2.44 (m, 4H), 2.37 – 2.27 (m, 1H), 2.17 (dd, *J* = 14.4, 6.4 Hz, 1H), 2.10 (dd, *J* = 14.3, 5.3 Hz, 1H), 1.20 (t, *J* = 7.1 Hz, 3H), 1.15 (t, *J* = 7.1 Hz, 3H).

^{13}C NMR (101 MHz, $CDCl_3$): δ 172.7, 172.1, 149.9, 147.2, 139.4, 138.4, 135.7, 133.8, 131.1, 129.6, 129.4, 128.3, 127.9, 127.2, 121.3, 61.9, 61.7, 58.2, 56.1, 43.5, 38.3, 37.6, 37.1, 34.5, 14.1, 14.0.

IR (ATR) ν 2977, 1725, 1585, 1452, 1374, 1452, 1259, 1148, 1095, 1020, 910, 844, 735 cm^{-1}.

HRMS (ESI) for $C_{28}H_{32}O_6NS^+$ $(M+H)^+$: 510.1945; Found : 510.1948.

Diethyl 3-((2,6-dimethylpyridin-4-yl)methyl)-4-((phenylsulfonyl)methyl)cyclopentane-1,1-dicarboxylate, **47**: the title compound was prepared following the general

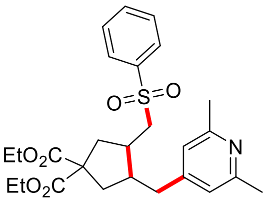

procedure using $NiCl_2$•$6H_2O$ (4.8 mg, 0.02 mmol, 10 mol%), 4,4'-di-tert-butyl-2,2'-bipyridine (8.1 mg, 0.03 mmol, 15 mol%), 4-chloro-2,6-dimethylpyridine (28.3 mg, 0.2 mmol, 1 equiv.), $[Ir(dtbbpy)(ppy)_2]PF_6$ (1.8 mg, 0.002 mmol, 1 mol%), diethyl diallylmalonate (96.1 mg, 0.4 mmol, 2 equiv.) and $PhSO_2Na$ (65.7 mg, 0.4 mmol, 2 equiv.). The reaction mixture was stirred in MeCN (2 mL) and irradiated with a 10 W blue LED strip at room temperature for 24 h. The crude product was purified by flash column chromatography to afford 44.8 mg (46%, 88:12 d.r.) of **47** as a colorless oil.

^{1}H NMR (400 MHz, $CDCl_3$): δ 7.88 (d, *J* = 7.1 Hz, 2H), 7.65 (t, *J* = 7.4 Hz, 1H), 7.56 (t, *J* = 7.6 Hz, 2H), 6.74 (s, 2H), 4.19 (q, *J* = 7.0 Hz, 2H), 4.13 (q, *J* = 7.0 Hz, 2H), 3.22 (dd, *J* = 14.0, 6.1 Hz, 1H), 3.15 (dd, *J* = 14.0, 7.6 Hz, 1H), 2.66 – 2.55 (m, 2H), 2.54 – 2.43 (m, 8H), 2.28 (dd, *J* = 13.9, 8.7 Hz, 1H), 2.25 – 2.16 (m, 2H), 2.03 (dd, *J* = 14.3, 5.4 Hz, 1H), 1.26 – 1.21 (m, 3H), 1.19 (d, *J* = 7.1 Hz, 3H).

^{13}C NMR (101 MHz, $CDCl_3$): δ 172.6, 172.1, 157.7, 149.6, 139.6, 133.9, 131.0, 129.5, 128.0, 121.0, 61.9, 61.8, 58.1, 56.1, 42.8, 38.2, 37.6, 37.1, 33.9, 24.4, 14.1 (2C).

IR (ATR) ν 2933, 1725, 1606, 1568, 1447, 1375, 1259, 1146, 1087, 1027, 918, 859, 738 cm^{-1}.

HRMS (ESI) for $C_{26}H_{34}O_6NS^+$ $(M+Na)^+$: 488.2101; Found : 488.2094.

Diethyl 3-(4-(4-((1-isopropoxy-2-methyl-1-oxopropan-2-yl)oxy)benzoyl)benzyl)-4-((phenylsulfonyl) methyl)cyclopentane-1,1-dicarboxylate, **48**: the title compound was prepared following the general procedure using $NiCl_2•6H_2O$ (4.8 mg, 0.02 mmol, 10 mol%), 4,4'-di-tert-butyl-2,2'-bipyridine (8.1 mg, 0.03 mmol, 15 mol%), fenofibrate (72.2 mg, 0.2 mmol, 1 equiv.), $[Ir(dtbbpy)(ppy)_2]PF_6$ (1.8 mg, 0.002 mmol, 1 mol%), diethyl 2,2-diallylmalonate (96.1 mg, 0.4 mmol, 2 equiv.) and $PhSO_2Na$ (65.7 mg, 0.4 mmol, 2 equiv.). The reaction mixture was stirred in MeCN (2 mL) and irradiated with a 10 W blue LED strip at room temperature for 24 h. The crude product was purified by flash column chromatography to afford 92.2 mg (65%, 88:12 d.r.) of **48** as a white solid.

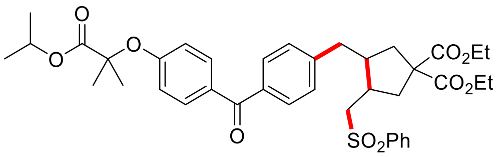

^{1}H NMR (600 MHz, $CDCl_3$): δ 7.90 (dd, *J* = 8.3, 1.3 Hz, 2H), 7.72 (d, *J* = 8.8 Hz, 2H), 7.64 (d, *J* = 7.8 Hz, 3H), 7.56 (t, *J* = 7.6 Hz, 2H), 7.23 (d, *J* = 8.0 Hz, 2H), 6.84 (d, *J* = 8.8 Hz, 2H), 5.12 – 5.01 (m, 1H), 4.19 (q, *J* = 7.1 Hz, 2H), 4.16 – 4.10 (m, 2H), 3.28 (dd, *J* = 14.1, 5.9 Hz, 1H), 3.19 (dd, *J* = 14.1, 7.9 Hz, 1H), 2.79 (dd, *J* = 13.2, 4.7 Hz, 1H), 2.65 – 2.58 (m, 1H), 2.57 – 2.49 (m, 2H), 2.36 (dd, *J* = 13.1, 11.1 Hz, 1H), 2.30 (dd, *J* = 14.0, 8.9 Hz, 1H), 2.19 (dd, *J* = 14.3, 6.8 Hz, 1H), 2.10 (dd, *J* = 14.3, 5.4 Hz, 1H), 1.64 (s, 6H), 1.23 (t, *J* = 7.1 Hz, 3H), 1.20 – 1.16 (m, 9H).

^{13}C NMR (151 MHz, $CDCl_3$): δ 195.2, 173.2, 172.6, 172.1, 159.5, 144.6, 139.5, 136.2, 133.9, 132.0, 130.7, 130.2, 129.5, 128.9, 128.0, 117.2, 79.4, 69.4, 61.9, 61.8, 58.1, 56.1, 43.6, 38.2, 37.5, 37.2, 34.6, 25.4, 21.6, 14.1 (2C).

IR (ATR) ν 2985, 2939, 1721, 1644, 1599, 1502, 1446, 1375, 1251, 1178, 1143, 1098, 1020, 969, 928, 852, 820, 792, 753 cm^{-1}.

HRMS (ESI) for $C_{39}H_{46}O_{10}NaS^+$ $(M+Na)^+$: 729.2704; Found : 729.2678.

Diethyl 3-(4-((((3S,8S,9S,10R,13S,14S,17S)-17-acetyl-10,13-dimethyl-2,3,4,7,8,9,10,11,12,13,14,15,16,17 -tetradecahydro-1H-cyclopenta[a]phenanthren-3-yl)oxy)carbonyl)benzyl)-4-((phenylsulfonyl)methyl) cyclopentane-1,1-dicarboxylate, **49**: the title compound was prepared following the general procedure using $NiCl_2•6H_2O$ (4.8 mg, 0.02 mmol, 10 mol%), 4,4'-di-tert-butyl-2,2'-bipyridine (8.1 mg, 0.03 mmol, 15 mol%), (3S,8S,9S,10R,13S,14S,17S)-17-acetyl-10,13-dimethyl-2,3,4,7,8,9,10,11,12,13,14,15,16,17-tetradecahydro-1H-cyclopenta[a]phenanthren-3-yl 4-bromobenzoate (99.9 mg, 0.2 mmol, 1 equiv.), $[Ir(dtbbpy)(ppy)_2]PF_6$ (1.8 mg, 0.002 mmol, 1 mol%), diethyl 2,2-diallylmalonate (96.1 mg, 0.4 mmol, 2 equiv.) and $PhSO_2Na$ (65.7 mg, 0.4 mmol, 2 equiv.). The reaction mixture was stirred in MeCN (2 mL) and irradiated with a 10 W blue LED strip at room temperature for 24 h. The crude product was purified by flash column chromatography to afford 91.4 mg (57%, 89:11 d.r.) of **49** as a white solid.

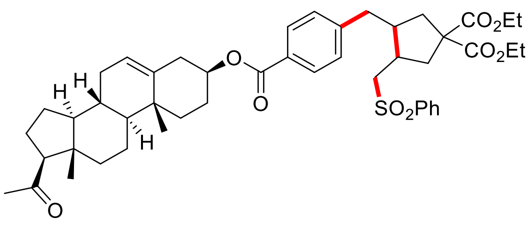

^{1}H NMR (600 MHz, CDCl$_3$): δ 7.94 – 7.83 (m, 4H), 7.64 (t, *J* = 7.4 Hz, 1H), 7.55 (t, *J* = 7.7 Hz, 2H), 7.18 (d, *J* = 8.0 Hz, 2H), 5.46 – 5.35 (m, 1H), 4.89 – 4.77 (m, 1H), 4.18 (q, *J* = 7.1 Hz, 2H), 4.15 – 4.09 (m, 2H), 3.25 (dd, *J* = 14.1, 5.7 Hz, 1H), 3.17 (dd, *J* = 14.1, 8.0 Hz, 1H), 2.75 (dd, *J* = 13.3, 4.8 Hz, 1H), 2.60 – 2.48 (m, 4H), 2.44 (d, *J* = 8.1 Hz, 2H), 2.39 – 2.27 (m, 2H), 2.21 – 2.13 (m, 2H), 2.11 (s, 3H), 2.07 – 1.96 (m, 4H), 1.94 – 1.86 (m, 1H), 1.74 – 1.55 (m, 5H), 1.52 – 1.41 (m, 3H), 1.24 – 1.20 (m, 5H), 1.20 – 1.15 (m, 4H), 1.07 – 0.99 (m, 4H), 0.62 (s, 3H).

^{13}C NMR (151 MHz, CDCl$_3$): δ 209.7, 172.6, 172.1, 166.0, 145.3, 139.7, 139.5, 133.9, 129.8, 129.5, 129.0, 128.8, 128.0, 122.5, 74.4, 63.7, 61.9, 61.7, 58.1, 56.9, 56.1, 49.9, 44.1, 43.5, 38.8, 38.2 (2C), 37.5, 37.1, 36.7, 34.6, 31.9 (2C), 31.7, 27.9, 24.6, 22.9, 21.1, 19.5, 14.1 (2C), 13.3.

IR (ATR) ν 3530, 2942, 2173, 1714, 1611, 1451, 1366, 1261, 1147, 1106, 1016, 947, 915, 858, 738 cm^{-1}.

HRMS (ESI) for $C_{47}H_{60}O_9NaS^+$ $(M+Na)^+$: 823.3850; Found : 823.3880.

Diethyl 3-(4-(3-(2-ethoxy-2-oxoethyl)-5-methoxy-2-methyl-1H-indole-1-carbonyl)benzyl)-4-((phenylsulfonyl)methyl)cyclopentane-1,1-dicarboxylate, 50:

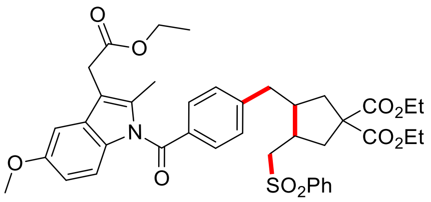

the title compound was prepared following the general procedure using NiCl$_2$•6H$_2$O (4.8 mg, 0.02 mmol, 10 mol%), 4,4'-di-tert-butyl-2,2'-bipyridine (8.1 mg, 0.03 mmol, 15 mol%), indomethacin ester (77.2 mg, 0.2 mmol, 1 equiv.), [Ir(dtbbpy)(ppy)$_2$]PF$_6$ (1.8 mg, 0.002 mmol, 1 mol%), diethyl 2,2-diallylmalonate (96.1 mg, 0.4 mmol, 2 equiv.) and PhSO$_2$Na (65.7 mg, 0.4 mmol, 2 equiv.). The reaction mixture was stirred in MeCN (2 mL) and irradiated with a 10 W blue LED strip at room temperature for 24 h. The crude product was purified by flash column chromatography to afford 124 mg (85%, 87:13 d.r.) of **50** as a white solid.

^{1}H NMR (600 MHz, CDCl$_3$): δ 7.94 – 7.88 (m, 2H), 7.65 (t, *J* = 7.5 Hz, 1H), 7.59 (d, *J* = 8.1 Hz, 2H), 7.56 (t, *J* = 7.8 Hz, 2H), 7.26 (d, *J* = 8.0 Hz, 2H), 6.96 (d, *J* = 2.5 Hz, 1H), 6.88 (d, *J* = 9.0 Hz, 1H), 6.64 (dd, *J* = 9.0, 2.6 Hz, 1H), 4.21 – 4.12 (m, 6H), 3.81 (s, 3H), 3.64 (s, 2H), 3.28 (dd, *J* = 14.1, 6.2 Hz, 1H), 3.20 (dd, *J* = 14.1, 7.8 Hz, 1H), 2.84 (dd, *J* = 13.1, 4.6 Hz, 1H), 2.67 – 2.59 (m, 1H), 2.59 – 2.50 (m, 2H), 2.42 – 2.33 (m, 4H), 2.29 (dd, *J* = 14.0, 9.1 Hz, 1H), 2.20 (dd, *J* = 14.3, 6.9 Hz, 1H), 2.08 (dd, *J* = 14.3, 5.2 Hz, 1H), 1.26 – 1.18 (m, 9H).

^{13}C NMR (151 MHz, CDCl$_3$): δ 172.6, 172.0, 171.0, 169.3, 155.9, 145.6, 139.5, 136.0, 133.9, 133.6, 131.0, 130.6, 130.0, 129.5, 129.4, 128.0, 115.0, 112.3, 111.6, 101.1, 61.9, 61.8, 61.0, 58.0, 56.1, 55.7, 43.5, 38.2, 37.4, 37.2, 34.5, 30.5, 14.3, 14.1, 14.0, 13.4.

IR (ATR) ν 2979, 1726, 1682, 1607, 1462, 1364, 1307, 1255, 1149, 1064, 1029, 914, 854, 800, 731 cm^{-1}.

HRMS (ESI) for $C_{40}H_{46}O_{10}NS^+$ $(M+H)^+$: 732.2837; Found : 732.2847.

Diethyl 3-(4-acetylbenzyl)-4-(tosylmethyl)cyclopentane-1,1-dicarboxylate, **51**: the title compound was prepared following the general procedure using $NiCl_2•6H_2O$ (4.8 mg, 0.02 mmol, 10 mol%), 4,4'-di-tert-butyl-2,2'-bipyridine (8.1 mg, 0.03 mmol, 15 mol%), 1-(4-bromophenyl)ethan-1-one (39.8 mg, 0.2 mmol, 1 equiv.), $[Ir(dtbbpy)(ppy)_2]PF_6$ (1.8 mg, 0.002 mmol, 1 mol%), diethyl diallylmalonate (96.1 mg, 0.4 mmol, 2 equiv.) and sodium 4-methylbenzenesulfinate (71.3 mg, 0.4 mmol, 2 equiv.). The reaction mixture was stirred in MeCN (2 mL) and irradiated with a 10 W blue LED strip at room temperature for 24 h. The crude product was purified by flash column chromatography to afford 98.4 mg (96%, 87:13 d.r.) of **51** as a white solid.

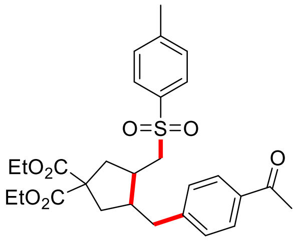

^{1}H NMR (400 MHz, CDCl$_3$): δ 7.81 (d, *J* = 8.0 Hz, 2H), 7.74 (d, *J* = 8.0 Hz, 2H), 7.31 (d, *J* = 8.0 Hz, 2H), 7.20 (d, *J* = 7.9 Hz, 2H), 4.16 (q, *J* = 7.0 Hz, 2H), 4.10 (q, *J* = 6.9 Hz, 2H), 3.22 (dd, *J* = 14.1, 5.7 Hz, 1H), 3.13 (dd, *J* = 14.0, 7.2 Hz, 1H), 2.75 (dd, *J* = 13.0, 4.4 Hz, 1H), 2.53 (s, 6H), 2.41 (s, 3H), 2.36 – 2.21 (m, 2H), 2.13 (dd, *J* = 14.2, 6.7 Hz, 1H), 2.03 (dd, *J* = 14.3, 5.1 Hz, 1H), 1.20 (t, *J* = 7.1 Hz, 3H), 1.15 (t, *J* = 7.1 Hz, 3H).

^{13}C NMR (101 MHz, CDCl$_3$): δ 197.8, 172.6, 172.1, 145.9, 144.9, 136.6, 135.4, 130.1, 129.3, 128.6, 128.0, 61.8, 61.7, 58.1, 56.2, 43.5, 38.2, 37.4, 37.3, 34.5, 26.6, 21.7, 14.1, 14.0.

IR (ATR) ν 2980, 1725, 1680, 1603, 1449, 1414, 1364, 1258, 1144, 1093, 1019, 958, 914, 859, 813, 762, 734 cm^{-1}.

HRMS (ESI) for $C_{28}H_{34}O_7NaS^+$ $(M+Na)^+$: 537.1918; Found : 537.1917.

Diethyl 3-(4-acetylbenzyl)-4-(((4-(tert-butyl)phenyl)sulfonyl)methyl)cyclopentane-1,1-dicarboxylate, **52**: the title compound was prepared following the general procedure using $NiCl_2•6H_2O$ (4.8 mg, 0.02 mmol, 10 mol%), 4,4'-di-tert-butyl-2,2'-bipyridine (8.1 mg, 0.03 mmol, 15 mol%), 1-(4-bromophenyl)ethan-1-one (39.8 mg, 0.2 mmol, 1 equiv.), $[Ir(dtbbpy)(ppy)_2]PF_6$ (1.8 mg, 0.002 mmol, 1 mol%), diethyl diallylmalonate (96.1 mg, 0.4 mmol, 2 equiv.) and sodium 4-*tert*butylbenzenesulfinate (88.1 mg, 0.4 mmol, 2 equiv.). The reaction mixture was stirred in MeCN (2 mL) and irradiated with a 10 W blue LED strip at room temperature for 24 h. The crude product was purified by flash column chromatography to afford 106 mg (93%, 86:14 d.r.) of **52** as a white solid.

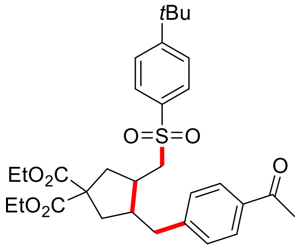

^{1}H NMR (400 MHz, CDCl$_3$): δ 7.85 – 7.74 (m, 4H), 7.53 (d, *J* = 8.6 Hz, 2H), 7.21 (d, *J* = 8.1 Hz, 2H), 4.16 (q, *J* = 7.1 Hz, 2H), 4.09 (qd, *J* = 7.4, 1.1 Hz, 2H), 3.25 (dd, *J* = 14.0, 6.3 Hz, 1H), 3.15 (dd, *J* = 14.0, 7.6 Hz, 1H), 2.76 (dd, *J* = 13.0, 4.4 Hz, 1H), 2.66 – 2.57 (m, 1H), 2.57 – 2.45 (m, 5H), 2.35 – 2.22 (m, 2H), 2.14 (dd, *J* = 14.4, 6.8 Hz, 1H), 2.03 (dd, *J* = 14.3, 5.1 Hz, 1H), 1.31 (s, 9H), 1.20 (t, *J* = 7.1 Hz, 3H), 1.16 (t, *J* = 7.1 Hz, 3H).

^{13}C NMR (101 MHz, CDCl$_3$): δ 197.8, 172.6, 172.1, 157.9, 145.9, 136.7, 135.4, 129.3, 128.6, 127.9, 126.5, 61.8, 61.7, 58.1, 56.2, 43.4, 38.3, 37.4, 37.2, 35.3, 34.5, 31.1, 26.6, 14.1, 14.0.

IR (ATR) ν 2965, 1726, 1681, 1603, 1456, 1403, 1365, 1303, 1258, 1151, 1103, 1018, 958, 914, 849, 778, 730 cm^{-1}.

HRMS (ESI) for $C_{31}H_{40}O_7NaS^+$ $(M+Na)^+$: 579.2387; Found : 579.2380

Diethyl 3-(4-acetylbenzyl)-4-(((4-(tert-butyl)phenyl)sulfonyl)methyl)cyclopentane-1,1-dicarboxylate, **53**:

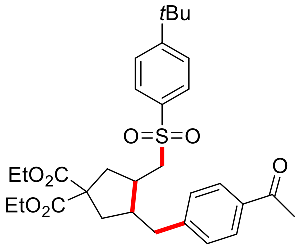

the title compound was prepared following the general procedure using $NiCl_2•6H_2O$ (4.8 mg, 0.02 mmol, 10 mol%), 4,4'-di-tert-butyl-2,2'-bipyridine (8.1 mg, 0.03 mmol, 15 mol%), 1-(4-bromophenyl)ethan-1-one (39.8 mg, 0.2 mmol, 1 equiv.), $[Ir(dtbbpy)(ppy)_2]PF_6$ (1.8 mg, 0.002 mmol, 1 mol%), diethyl diallylmalonate (96.1 mg, 0.4 mmol, 2 equiv.) and sodium 4-*tert*butylbenzenesulfinate (88.1 mg, 0.4 mmol, 2 equiv.). The reaction mixture was stirred in MeCN (2 mL) and irradiated with a 10 W blue LED strip at room temperature for 24 h. The crude product was purified by flash column chromatography to afford 106 mg (93%, 86:14 d.r.) of **53** as a white solid.

^{1}H NMR (400 MHz, $CDCl_3$): δ 7.85 – 7.74 (m, 4H), 7.53 (d, *J* = 8.6 Hz, 2H), 7.21 (d, *J* = 8.1 Hz, 2H), 4.16 (q, *J* = 7.1 Hz, 2H), 4.09 (qd, *J* = 7.4, 1.1 Hz, 2H), 3.25 (dd, *J* = 14.0, 6.3 Hz, 1H), 3.15 (dd, *J* = 14.0, 7.6 Hz, 1H), 2.76 (dd, *J* = 13.0, 4.4 Hz, 1H), 2.66 – 2.57 (m, 1H), 2.57 – 2.45 (m, 5H), 2.35 – 2.22 (m, 2H), 2.14 (dd, *J* = 14.4, 6.8 Hz, 1H), 2.03 (dd, *J* = 14.3, 5.1 Hz, 1H), 1.31 (s, 9H), 1.20 (t, *J* = 7.1 Hz, 3H), 1.16 (t, *J* = 7.1 Hz, 3H).

^{13}C NMR (101 MHz, $CDCl_3$): δ 197.8, 172.6, 172.1, 157.9, 145.9, 136.7, 135.4, 129.3, 128.6, 127.9, 126.5, 61.8, 61.7, 58.1, 56.2, 43.4, 38.3, 37.4, 37.2, 35.3, 34.5, 31.1, 26.6, 14.1, 14.0.

IR (ATR) ν 2965, 1726, 1681, 1603, 1456, 1403, 1365, 1303, 1258, 1151, 1103, 1018, 958, 914, 849, 778, 730 cm^{-1}.

HRMS (ESI) for $C_{31}H_{40}O_7NaS^+$ $(M+Na)^+$: 579.2387; Found : 579.2380

Diethyl 3-(4-acetylbenzyl)-4-(((4-chlorophenyl)sulfonyl)methyl)cyclopentane-1,1-dicarboxylate, **54**:

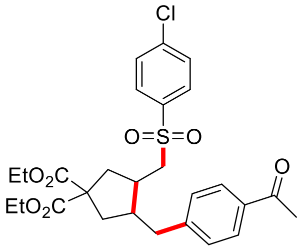

the title compound was prepared following the general procedure using $NiCl_2•6H_2O$ (4.8 mg, 0.02 mmol, 10 mol%), 4,4'-di-tert-butyl-2,2'-bipyridine (8.1 mg, 0.03 mmol, 15 mol%), 1-(4-bromophenyl)ethan-1-one (39.8 mg, 0.2 mmol, 1 equiv.), $[Ir(dtbbpy)(ppy)_2]PF_6$ (1.8 mg, 0.002 mmol, 1 mol%), diethyl diallylmalonate (96.1 mg, 0.4 mmol, 2 equiv.) and sodium 4-chlorobenzenesulfinate (79.4 mg, 0.4 mmol, 2 equiv.). The reaction mixture was stirred in MeCN (2 mL) and irradiated with a 10 W blue LED strip at room temperature for 24 h. The crude product was purified by flash column chromatography to afford 89.3 mg (83%, 87:13 d.r.) of **54** as a white solid.

^{1}H NMR (400 MHz, $CDCl_3$): δ 7.86 – 7.77 (m, 4H), 7.50 (d, *J* = 8.6 Hz, 2H), 7.20 (d, *J* = 8.0 Hz, 2H), 4.16 (q, *J* = 7.2 Hz, 2H), 4.10 (qd, *J* = 7.1, 1.4 Hz, 2H), 3.23 (dd, *J* = 14.1, 5.4 Hz, 1H), 3.16 (dd, *J* =

14.1, 7.3 Hz, 1H), 2.74 (dd, J = 13.0, 4.5 Hz, 1H), 2.58 – 2.46 (m, 6H), 2.38 – 2.23 (m, 2H), 2.15 (dd, J = 14.2, 6.6 Hz, 1H), 2.04 (dd, J = 14.3, 5.3 Hz, 1H), 1.21 (t, J = 7.1 Hz, 3H), 1.16 (t, J = 7.1 Hz, 3H).

^{13}C NMR (101 MHz, CDCl$_3$): δ 197.8, 172.6, 172.1, 145.6, 140.7, 138.0, 135.5, 129.8, 129.6, 129.2, 128.7, 61.9, 61.8, 58.1, 56.2, 43.5, 38.2, 37.5, 37.1, 34.6, 26.6, 14.1, 14.0.

IR (ATR) ν 2978, 2932, 2171, 1723, 1590, 1458, 1369, 1254, 1144, 1079, 1012, 951, 832, 782 cm^{-1}.

HRMS (ESI) for $C_{27}H_{31}O_7ClNaS^+$ (M+Na)$^+$: 557.1371; Found : 557.1382.

Diethyl 3-(4-acetylbenzyl)-4-(((4-cyanophenyl)sulfonyl)methyl)cyclopentane-1,1-dicarboxylate, **55**:

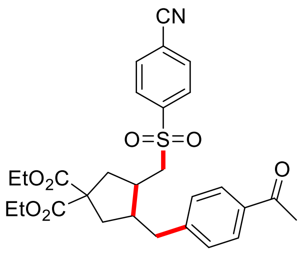

the title compound was prepared following the general procedure using NiCl$_2$•6H$_2$O (4.8 mg, 0.02 mmol, 10 mol%), 4,4'-di-tert-butyl-2,2'-bipyridine (8.1 mg, 0.03 mmol, 15 mol%), 1-(4-bromophenyl)ethan-1-one (39.8 mg, 0.2 mmol, 1 equiv.), [Ir(dtbbpy)(ppy)$_2$]PF$_6$ (1.8 mg, 0.002 mmol, 1 mol%), diethyl diallylmalonate (96.1 mg, 0.4 mmol, 2 equiv.) and sodium 4-cyanobenzenesulfinate (75.7 mg, 0.4 mmol, 2 equiv.). The reaction mixture was stirred in MeCN (2 mL) and irradiated with a 10 W blue LED strip at room temperature for 24 h. The crude product was purified by flash column chromatography to afford 62.8 mg (60%, 90:10 d.r.) of **55** as a white solid.

^{1}H NMR (400 MHz, CDCl$_3$): δ 8.01 (d, J = 8.5 Hz, 2H), 7.88 – 7.80 (m, 4H), 7.22 (d, J = 8.2 Hz, 2H), 4.17 (q, J = 7.1 Hz, 2H), 4.11 (qd, J = 7.1, 1.4 Hz, 2H), 3.29 – 3.17 (m, 2H), 2.75 (dd, J = 13.0, 4.5 Hz, 1H), 2.62 – 2.46 (m, 6H), 2.36 (dd, J = 13.0, 10.7 Hz, 1H), 2.26 (dd, J = 13.8, 8.2 Hz, 1H), 2.16 (dd, J = 14.4, 6.8 Hz, 1H), 2.04 (dd, J = 14.3, 5.5 Hz, 1H), 1.21 (t, J = 7.1 Hz, 3H), 1.17 (t, J = 7.1 Hz, 3H).

^{13}C NMR (101 MHz, CDCl$_3$): δ 197.8, 172.6, 172.0, 145.5, 143.6, 135.6, 133.3, 129.2, 128.9, 128.7, 117.8, 117.1, 62.0, 61.8, 58.1, 56.0, 43.7, 38.2, 37.5, 37.1, 34.6, 26.7, 14.1 (2C).

IR (ATR) ν 2978, 2234, 1724, 1680, 1605, 1452, 1405, 1367, 1258, 1144, 1098, 1017, 958, 915, 850, 803, 732 cm^{-1}.

HRMS (ESI) for $C_{28}H_{31}O_7NNaS^+$ (M+Na)$^+$: 548.1713; Found : 548.1702.

Diethyl 3-(4-acetylbenzyl)-4-(((4-(trifluoromethyl)phenyl)sulfonyl)methyl)cyclopentane-1,1-dicarboxylate, **56**:

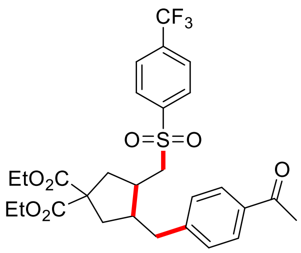

the title compound was prepared following the general procedure using NiCl$_2$•6H$_2$O (4.8 mg, 0.02 mmol, 10 mol%), 4,4'-di-tert-butyl-2,2'-bipyridine (8.1 mg, 0.03 mmol, 15 mol%), 1-(4-bromophenyl)ethan-1-one (39.8 mg, 0.2 mmol, 1 equiv.), [Ir(dtbbpy)(ppy)$_2$]PF$_6$ (1.8 mg, 0.002 mmol, 1 mol%), diethyl diallylmalonate (96.1 mg, 0.4 mmol, 2 equiv.) and sodium 4-trifluoromethylbenzenesulfinate (92.9 mg, 0.4 mmol, 2 equiv.). The reaction mixture was stirred in MeCN (2 mL) and irradiated with a 10 W blue LED strip at room temperature for 24 h. The crude product was purified by flash column chromatography to afford 106 mg (93%, 89:11 d.r.) of **56** as a white solid.

^{1}H NMR (400 MHz, CDCl$_3$): δ 8.02 (d, *J* = 8.1 Hz, 2H), 7.81 (t, *J* = 7.6 Hz, 4H), 7.21 (d, *J* = 8.0 Hz, 2H), 4.17 (q, *J* = 7.1 Hz, 2H), 4.11 (qd, *J* = 7.1, 1.6 Hz, 2H), 3.27 (dd, *J* = 14.0, 5.7 Hz, 1H), 3.20 (dd, *J* = 14.0, 7.5 Hz, 1H), 2.75 (dd, *J* = 13.1, 4.6 Hz, 1H), 2.63 – 2.45 (m, 6H), 2.40 – 2.25 (m, 2H), 2.16 (dd, *J* = 14.4, 6.7 Hz, 1H), 2.04 (dd, *J* = 14.3, 5.5 Hz, 1H), 1.21 (t, *J* = 7.1 Hz, 3H), 1.16 (t, *J* = 7.1 Hz, 3H).
^{13}C NMR (101 MHz, CDCl$_3$): δ 197.7, 172.6, 172.0, 145.6, 143.1, 135.6, 135.5, 129.2, 128.8, 128.7, 126.6, 61.9, 61.8, 58.1, 56.0, 43.6, 38.2, 37.5, 37.1, 34.6, 26.6, 14.1, 14.0.
^{19}F NMR (376 MHz, CDCl$_3$) δ -63.2.
IR (ATR) ν 2982, 2931, 1722, 1683, 1605, 1458, 1405, 1367, 1314, 1254, 1132, 1058, 1013, 952, 845, 798, 705 cm^{-1}.
HRMS (ESI) for $C_{28}H_{31}O_7F_3NaS^+$ $(M+Na)^+$: 591.1635; Found : 591.1629.

Diethyl 3-(4-acetylbenzyl)-4-((o-tolylsulfonyl)methyl)cyclopentane-1,1-dicarboxylate, **57**: the title compound was prepared following the general procedure using NiCl$_2$•6H$_2$O (4.8 mg, 0.02 mmol, 10 mol%), 4,4'-di-tert-butyl-2,2'-bipyridine (8.1 mg, 0.03 mmol, 15 mol%), 1-(4-bromophenyl)ethan-1-one (39.8 mg, 0.2 mmol, 1 equiv.), [Ir(dtbbpy)(ppy)$_2$]PF$_6$ (1.8 mg, 0.002 mmol, 1 mol%), diethyl diallylmalonate (96.1 mg, 0.4 mmol, 2 equiv.) and sodium 2-methylbenzenesulfinate (71.3 mg, 0.4 mmol, 2 equiv.). The reaction mixture was stirred in MeCN (2 mL) and irradiated with a 10 W blue LED strip at room temperature for 24 h. The crude product was purified by flash column chromatography to afford 101 mg (98%, 84:16 d.r.) of **57** as a white solid.

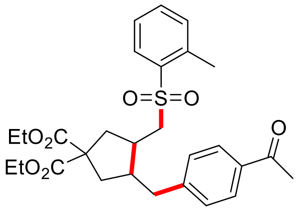

^{1}H NMR (400 MHz, CDCl$_3$): δ 7.96 (dd, *J* = 7.9, 1.4 Hz, 1H), 7.81 (d, *J* = 8.1 Hz, 2H), 7.48 (td, *J* = 7.5, 1.4 Hz, 1H), 7.34 (t, *J* = 7.5 Hz, 1H), 7.29 (d, *J* = 7.8 Hz, 1H), 7.20 (d, *J* = 8.0 Hz, 2H), 4.15 (q, *J* = 7.1 Hz, 2H), 4.12 – 4.06 (m, 2H), 3.24 (dd, *J* = 14.0, 6.1 Hz, 1H), 3.17 (dd, *J* = 14.0, 7.8 Hz, 1H), 2.77 (dd, *J* = 13.1, 4.8 Hz, 1H), 2.63 (s, 3H), 2.58 – 2.44 (m, 5H), 2.39 – 2.32 (m, 1H), 2.32 – 2.22 (m, 2H), 2.15 (dd, *J* = 14.4, 6.8 Hz, 1H), 2.02 (dd, *J* = 14.3, 5.8 Hz, 1H), 1.19 (t, *J* = 7.0 Hz, 3H), 1.15 (t, *J* = 7.1 Hz, 3H).
^{13}C NMR (101 MHz, CDCl$_3$): δ 197.7, 172.5, 171.9, 145.7, 137.9, 137.6, 135.3, 133.8, 132.8, 130.0, 129.1, 128.5, 126.7, 61.7, 61.6, 58.0, 55.0, 43.4, 38.3, 37.4, 36.9, 34.5, 26.5, 20.4, 14.0, 13.9.
IR (ATR) ν 2979, 1725, 1682, 1605, 1456, 1364, 1258, 1147, 1054, 1025, 914, 860, 745 cm^{-1}.
HRMS (ESI) for $C_{28}H_{34}O_7NaS^+$ $(M+Na)^+$: 537.1918; Found : 537.1917.

Diethyl 3-(4-acetylbenzyl)-4-((methylsulfonyl)methyl)cyclopentane-1,1-dicarboxylate, **58**: the title compound was prepared following the general procedure using NiCl$_2$•6H$_2$O (4.8 mg, 0.02 mmol, 10 mol%), 4,4'-di-tert-butyl-2,2'-bipyridine (8.1 mg, 0.03 mmol, 15 mol%), 1-(4-bromophenyl)ethan-1-one (39.8 mg, 0.2 mmol, 1 equiv.), [Ir(dtbbpy)(ppy)$_2$]PF$_6$ (1.8 mg, 0.002 mmol, 1 mol%), diethyl diallylmalonate (96.1 mg, 0.4 mmol, 2 equiv.) and sodium methanesulfinate (40.8 mg, 0.4 mmol, 2 equiv.). The reaction mixture was stirred in MeCN (2 mL) and irradiated with a 10 W blue LED strip at

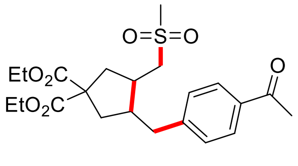

room temperature for 24 h. The crude product was purified by flash column chromatography to afford 86.1 mg (98%, 85:15 d.r.) of **58** as a white solid.

^{1}H NMR (400 MHz, CDCl$_3$): δ 7.83 (d, *J* = 8.2 Hz, 2H), 7.25 (d, *J* = 8.2 Hz, 2H), 4.16 (q, *J* = 7.1 Hz, 2H), 4.10 (q, *J* = 7.1 Hz, 2H), 3.19 (dd, *J* = 13.9, 6.4 Hz, 1H), 3.11 (dd, *J* = 13.9, 7.8 Hz, 1H), 2.90 (s, 3H), 2.84 – 2.78 (m, 1H), 2.75 – 2.66 (m, 1H), 2.61 – 2.50 (m, 5H), 2.39 – 2.27 (m, 2H), 2.18 (dd, *J* = 14.3, 6.9 Hz, 1H), 2.05 (dd, *J* = 14.4, 5.2 Hz, 1H), 1.20 (t, *J* = 7.1 Hz, 3H), 1.16 (t, *J* = 7.1 Hz, 3H).

^{13}C NMR (101 MHz, CDCl$_3$): δ 197.8, 172.6, 172.1, 145.8, 135.5, 129.3, 128.6, 61.9, 61.8, 58.1, 54.4, 43.5, 41.7, 38.4, 37.4, 37.0, 34.5, 26.6, 14.1, 14.0.

IR (ATR) ν 2935, 1724, 1680, 1605, 1452, 1364, 1260, 1175, 1131, 1020, 961, 861, 733 cm^{-1}.

HRMS (ESI) for $C_{22}H_{30}O_7NaS^+$ $(M+Na)^+$: 461.1604; Found : 461.1598.

Diethyl 3-(4-acetylbenzyl)-4-((ethylsulfonyl)methyl)cyclopentane-1,1-dicarboxylate, **59**: the title

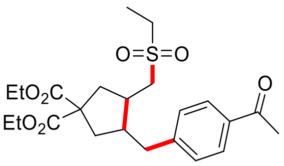

compound was prepared following the general procedure using $NiCl_2•6H_2O$ (4.8 mg, 0.02 mmol, 10 mol%), 4,4'-di-tert-butyl-2,2'-bipyridine (8.1 mg, 0.03 mmol, 15 mol%), 1-(4-bromophenyl)ethan-1-one (39.8 mg, 0.2 mmol, 1 equiv.), $[Ir(dtbbpy)(ppy)_2]PF_6$ (1.8 mg, 0.002 mmol, 1 mol%), diethyl diallylmalonate (96.1 mg, 0.4 mmol, 2 equiv.) and sodium ethanesulfinate (46.4 mg, 0.4 mmol, 2 equiv.). The reaction mixture was stirred in MeCN (2 mL) and irradiated with a 10 W blue LED strip at room temperature for 24 h. The crude product was purified by flash column chromatography to afford 66.6 mg (74%, 89:11 d.r.) of **59** as a white solid.

^{1}H NMR (400 MHz, CDCl$_3$): δ 7.85 (d, *J* = 8.3 Hz, 2H), 7.27 (d, *J* = 8.1 Hz, 2H), 4.18 (q, *J* = 7.1 Hz, 2H), 4.12 (q, *J* = 7.1 Hz, 2H), 3.11 (dd, *J* = 13.7, 6.8 Hz, 1H), 3.08 – 3.03 (m, 1H), 2.99 (q, *J* = 7.6 Hz, 2H), 2.85 (dd, *J* = 12.9, 4.4 Hz, 1H), 2.80 – 2.68 (m, 1H), 2.64 – 2.51 (m, 5H), 2.41 – 2.28 (m, 2H), 2.20 (dd, *J* = 14.3, 7.0 Hz, 1H), 2.07 (dd, *J* = 14.4, 5.1 Hz, 1H), 1.38 (t, *J* = 7.5 Hz, 3H), 1.22 (t, *J* = 7.1 Hz, 3H), 1.18 (t, *J* = 7.1 Hz, 3H).

^{13}C NMR (101 MHz, CDCl$_3$): δ 197.9, 172.7, 172.1, 145.9, 135.5, 129.4, 128.7, 61.9, 61.8, 58.1, 51.5, 48.4, 43.6, 38.6, 37.5, 36.6, 34.6, 26.7, 14.1 (2C), 6.8.

IR (ATR) ν 2976, 1725, 1681, 1605, 1451, 1366, 1259, 1174, 1125, 1029, 960, 914, 860, 802, 728 cm^{-1}.

HRMS (ESI) for $C_{23}H_{32}O_7NaS^+$ $(M+Na)^+$: 475.1761; Found : 475.1750.

Diethyl 3-(4-acetylbenzyl)-4-((cyclopropylsulfonyl)methyl)cyclopentane-1,1-dicarboxylate, **60**: the

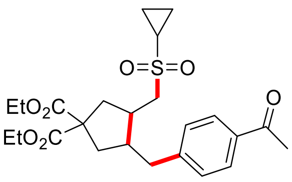

title compound was prepared following the general procedure using $NiCl_2•6H_2O$ (4.8 mg, 0.02 mmol, 10 mol%), 4,4'-di-tert-butyl-2,2'-bipyridine (8.1 mg, 0.03 mmol, 15 mol%), 1-(4-bromophenyl)ethan-1-one (39.8 mg, 0.2 mmol, 1 equiv.), $[Ir(dtbbpy)(ppy)_2]PF_6$ (1.8 mg, 0.002 mmol, 1 mol%), diethyl diallylmalonate (96.1 mg, 0.4 mmol, 2 equiv.) and sodium cyclopropanesulfinate (51.3 mg, 0.4 mmol, 2 equiv.). The reaction mixture was stirred in MeCN (2 mL) and irradiated with a 10 W blue LED

strip at room temperature for 24 h. The crude product was purified by flash column chromatography to afford 90.8 mg (98%, 85:15 d.r.) of **60** as a white solid.

^{1}H NMR (400 MHz, CDCl$_3$): δ 7.83 (d, *J* = 8.1 Hz, 2H), 7.26 (d, *J* = 8.1 Hz, 2H), 4.17 (q, *J* = 7.1 Hz, 2H), 4.11 (q, *J* = 7.2 Hz, 2H), 3.20 (dd, *J* = 13.9, 6.5 Hz, 1H), 3.12 (dd, *J* = 13.9, 7.8 Hz, 1H), 2.87 – 2.70 (m, 2H), 2.63 – 2.49 (m, 5H), 2.43 – 2.26 (m, 3H), 2.17 (dd, *J* = 14.4, 6.9 Hz, 1H), 2.07 (dd, *J* = 14.3, 5.1 Hz, 1H), 1.25 – 1.12 (m, 8H), 1.05 – 0.96 (m, 2H).

^{13}C NMR (101 MHz, CDCl$_3$): δ 197.8, 172.6, 172.1, 145.9, 135.4, 129.3, 128.6, 61.9, 61.7, 58.1, 53.5, 43.6, 38.5, 37.4, 36.9, 34.5, 30.2, 26.6, 14.1, 14.0, 4.9.

IR (ATR) ν 2977, 1725, 1682, 1605, 1451, 1364, 1260, 1176, 1126, 1029, 959, 883, 728 cm^{-1}.

HRMS (ESI) for $C_{24}H_{32}O_7NaS^+$ $(M+Na)^+$: 487.1761; Found : 487.1758.

Diethyl 3-(4-acetylbenzyl)-4-((thiophen-3-ylsulfonyl)methyl)cyclopentane-1,1-dicarboxylate, **61**: the

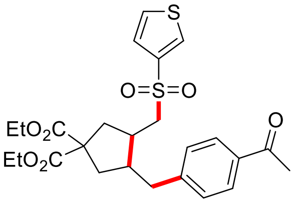

title compound was prepared following the general procedure using NiCl$_2$•6H$_2$O (4.8 mg, 0.02 mmol, 10 mol%), 4,4'-di-tert-butyl-2,2'-bipyridine (8.1 mg, 0.03 mmol, 15 mol%), 1-(4-bromophenyl)ethan-1-one (39.8 mg, 0.2 mmol, 1 equiv.), [Ir(dtbbpy)(ppy)$_2$]PF$_6$ (1.8 mg, 0.002 mmol, 1 mol%), diethyl diallylmalonate (96.1 mg, 0.4 mmol, 2 equiv.) and sodium thiophene-3-sulfinate (68.1 mg, 0.4 mmol, 2 equiv.). The reaction mixture was stirred in MeCN (2 mL) and irradiated with a 10 W blue LED strip at room temperature for 24 h. The crude product was purified by flash column chromatography to afford 93.6 mg (92%, 88:12 d.r.) of **61** as a white solid.

^{1}H NMR (400 MHz, CDCl$_3$): δ 7.82 (d, *J* = 8.2 Hz, 2H), 7.69 (dd, *J* = 5.0, 1.3 Hz, 1H), 7.65 (dd, *J* = 3.7, 1.3 Hz, 1H), 7.22 (d, *J* = 8.1 Hz, 2H), 7.14 – 7.10 (m, 1H), 4.16 (q, *J* = 7.0 Hz, 2H), 4.13 – 4.07 (m, 2H), 3.37 (dd, *J* = 14.1, 5.8 Hz, 1H), 3.27 (dd, *J* = 14.1, 7.8 Hz, 1H), 2.77 (dd, *J* = 13.0, 4.5 Hz, 1H), 2.64 – 2.57 (m, 1H), 2.57 – 2.47 (m, 5H), 2.39 – 2.30 (m, 1H), 2.26 (dd, *J* = 13.9, 8.9 Hz, 1H), 2.15 (dd, *J* = 14.3, 6.8 Hz, 1H), 2.04 (dd, *J* = 14.3, 5.3 Hz, 1H), 1.20 (t, *J* = 7.1 Hz, 3H), 1.16 (t, *J* = 7.1 Hz, 3H).

^{13}C NMR (101 MHz, CDCl$_3$): δ 197.8, 172.5, 172.1, 145.7, 140.5, 135.4, 134.3, 134.2, 129.3, 128.6, 128.1, 61.9, 61.7, 58.1, 57.7, 43.5, 38.2, 37.6, 37.5, 34.6, 26.6, 14.1, 14.0.

IR (ATR) ν 2979, 1724, 1680, 1605, 1453, 1406, 1363, 1308, 1258, 1139, 1016, 958, 914, 858, 730 cm^{-1}.

HRMS (ESI) for $C_{25}H_{30}O_7NaS_2^+$ $(M+Na)^+$: 529.1325; Found : 529.1325.

Diethyl 3-(4-acetylbenzyl)-4-((pyridin-3-ylsulfonyl)methyl)cyclopentane-1,1-dicarboxylate, **62**: the

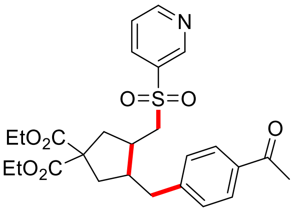

title compound was prepared following the general procedure using NiCl$_2$•6H$_2$O (4.8 mg, 0.02 mmol, 10 mol%), 4,4'-di-tert-butyl-2,2'-bipyridine (8.1 mg, 0.03 mmol, 15 mol%), 1-(4-bromophenyl)ethan-1-one (39.8 mg, 0.2 mmol, 1 equiv.), [Ir(dtbbpy)(ppy)$_2$]PF$_6$ (1.8 mg, 0.002 mmol, 1 mol%), diethyl diallylmalonate (96.1 mg, 0.4 mmol, 2 equiv.) and sodium pyridine-3-sulfinate (66.1 mg, 0.4 mmol, 2 equiv.). The reaction mixture was stirred in MeCN (2 mL) and irradiated

with a 10 W blue LED strip at room temperature for 24 h. The crude product was purified by flash column chromatography to afford 70.8 mg (71%, 87:13 d.r.) of **62** as a white solid.

^{1}H NMR (400 MHz, CDCl$_3$): δ 9.08 (d, *J* = 2.3 Hz, 1H), 8.84 (dd, *J* = 4.8, 1.6 Hz, 1H), 8.15 (dt, *J* = 8.0, 2.0 Hz, 1H), 7.82 (d, *J* = 8.1 Hz, 2H), 7.48 (dd, *J* = 8.1, 4.8 Hz, 1H), 7.21 (d, *J* = 8.0 Hz, 2H), 4.17 (q, *J* = 7.2 Hz, 2H), 4.14 – 4.07 (m, 2H), 3.32 – 3.18 (m, 2H), 2.75 (dd, *J* = 13.1, 4.7 Hz, 1H), 2.63 – 2.47 (m, 6H), 2.37 (dd, *J* = 13.0, 10.7 Hz, 1H), 2.29 (dd, *J* = 13.7, 8.0 Hz, 1H), 2.17 (dd, *J* = 14.3, 6.7 Hz, 1H), 2.04 (dd, *J* = 14.3, 5.5 Hz, 1H), 1.21 (t, *J* = 7.1 Hz, 3H), 1.17 (t, *J* = 7.1 Hz, 3H).

^{13}C NMR (101 MHz, CDCl$_3$): δ 197.8, 172.6, 172.1, 145.9, 135.4, 129.3, 128.6, 61.9, 61.7, 58.1, 53.5, 43.6, 38.5, 37.4, 36.9, 34.5, 30.2, 26.6, 14.1, 14.0, 4.9.

IR (ATR) ν 2936, 1725, 1682, 1574, 1457, 1418, 1365, 1260, 1156, 11106, 1020, 859, 704 cm^{-1}.

HRMS (ESI) for $C_{26}H_{31}O_7NNaS^+$ (M+Na)$^+$: 524.1713; Found : 524.1714.

3-([1,1'-Biphenyl]-4-ylmethyl)-4-((phenylsulfonyl)methyl)tetrahydrofuran, **65**: the title compound

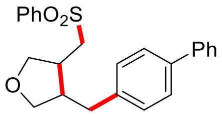

was prepared following the general procedure using NiCl$_2$•6H$_2$O (95.1 mg, 0.4 mmol, 10 mol%), 4,4'-di-tert-butyl-2,2'-bipyridine (161 mg, 0.6 mmol, 15 mol%), 4-bromo-1,1'-biphenyl (932 mg, 4 mmol, 1 equiv.), dicyanobenzene **PC-V** (78.9 mg, 0.1 mmol, 2.5 mol%), diallylether (785 mg, 8 mmol, 2 equiv.) and PhSO$_2$Na (1313 mg, 8 mmol, 2 equiv.). The reaction mixture was stirred in MeCN (40 mL) and irradiated with a 10 W blue LED strip at room temperature for 36 h. The crude product was purified by flash column chromatography to afford 1220 mg (78%, 80:20 d.r.) of **3a** as a colorless oil.

^{1}H NMR (600 MHz, CDCl$_3$): *cis* isomer δ 7.92 (d, *J* = 7.9 Hz, 2H), 7.67 (t, *J* = 7.4 Hz, 1H), 7.61 – 7.54 (m, 4H), 7.53 – 7.42 (m, 4H), 7.39 – 7.32 (m, 1H), 7.19 (d, *J* = 7.6 Hz, 2H), 4.03 (t, *J* = 7.9 Hz, 1H), 3.84 – 3.79 (m, 1H), 3.79 – 3.72 (m, 1H), 3.59 (dd, *J* = 8.4, 4.7 Hz, 1H), 3.39 (dd, *J* = 13.9, 4.6 Hz, 1H), 3.20 (dd, *J* = 14.0, 9.4 Hz, 1H), 2.88 – 2.81 (m, 1H), 2.81 – 2.67 (m, 2H), 2.47 (dd, *J* = 13.3, 10.6 Hz, 1H); *trans* isomer δ 7.77 (d, *J* = 8.0 Hz, 2H), 7.62 (t, *J* = 7.4 Hz, 1H), 7.14 (d, *J* = 8.0 Hz, 2H), 4.20 – 4.13 (m, 1H), 3.91 (t, *J* = 8.1 Hz, 1H), 3.66 (dd, *J* = 9.4, 6.7 Hz, 1H), 3.51 (dd, *J* = 8.4, 7.0 Hz, 1H), 3.02 – 2.95 (m, 2H), 2.43 – 2.37 (m, 1H), 2.32 – 2.24 (m, 1H), the remaining resonances are insufficiently resolved from those of the cis isomer to be reported.

^{13}C NMR (151 MHz, CDCl$_3$): *cis* isomer δ 140.7, 139.4, 138.5, 134.0, 129.5, 129.2, 128.9, 128.0, 127.4, 127.0, 71.6, 71.4, 55.0, 43.2, 36.6, 33.2; *trans* isomer δ 140.6, 139.2, 138.3, 133.9, 129.4, 128.9, 128.0, 127.3, 127.0, 73.0, 72.6, 59.5, 46.7, 39.4, 38.3. the remaining resonances are insufficiently resolved from those of the cis isomer to be reported.

IR (ATR) ν 2931, 2857, 1485, 1446, 1406, 1306, 1145, 1084, 1048, 1011, 908, 863, 732 cm^{-1};

HRMS (ESI) for $C_{24}H_{24}O_3NaS^+$ (M+Na)$^+$: 415.1338; Found : 415.1335.

4-(2,3-Dimethyl-4-(phenylsulfonyl)but-2-en-1-yl)-1,1'-biphenyl, **67**: the title compound was prepared following

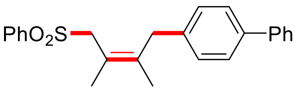

the general procedure using $NiCl_2$•$6H_2O$ (95.1 mg, 0.4 mmol, 10 mol%), 4,4'-di-tert-butyl-2,2'-bipyridine (161 mg, 0.6 mmol, 15 mol%), 4-bromo-1,1'-biphenyl (932 mg, 4 mmol, 1 equiv.), dicyanobenzene **PC-V** (78.9 mg, 0.1 mmol, 2.5 mol%), 2,3-dimethylbuta-1,3-diene (986 mg, 12 mmol, 3 equiv.) and $PhSO_2Na$ (1970 mg, 12 mmol, 3 equiv.). The reaction mixture was stirred in MeCN (40 mL) and irradiated with a 10 W blue LED strip at room temperature for 48 h. The crude product was purified by flash column chromatography to afford 1018 mg (66%, 1.3:1 *Z*/*E*) of **67** as a white solid.

^{1}H NMR (400 MHz, $CDCl_3$): *E* + *Z* isomer δ 8.19 – 7.80 (m, 3.5H_{E+Z}), 7.80 – 6.87 (m, 21H_{E+Z}), 4.04 (s, 2H_Z), 4.00 (s, 1.5H_E), 3.44 (s, 1.5H_E), 3.18 (s, 2H_Z), 2.02 (s, 2.2H_E), 1.94 (s, 3H_Z), 1.67 (s, 3H_Z), 1.30 (s, 2.2H_E).

^{13}C NMR (100 MHz, $CDCl_3$): *E* + *Z* isomer δ 140.9 (2C), 139.4, 139.2, 139.1, 139.0, 138.3, 138.2, 138.1, 133.7, 133.6, 129.2, 129.1, 129.0, 128.8 (2C), 128.5, 128.4, 127.2, 127.1 (2C), 127.0, 118.3, 118.2, 62.0, 61.7, 40.1, 39.4, 19.9, 19.8, 19.1, 18.7.

IR (ATR) ν 3058, 2928, 1588, 1485, 1446, 1405, 1302, 1238, 1177, 1141, 1082, 1001, 843, 745, 688 cm^{-1};

HRMS (ESI) for $C_{24}H_{24}O_2NaS^+$ $(M+Na)^+$: 399.1389; Found : 399.1404.

3-([1,1'-Biphenyl]-4-ylmethyl)-4-styryltetrahydrofuran, **68**: To a flame-dried round bottom flask equipped with

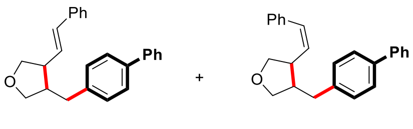

68' 68''

a stirring bar was charged with 3-([1,1'-biphenyl]-4-ylmethyl)-4-((phenylsulfonyl)methyl)tetrahydrofuran (**65**, 393 mg, 1 mmol, 1 equiv.) in 2 mL under argon. The solution was cooled to -78 ºC, n-BuLi (0.69 mL, 1.6 M in hexane, 1.1 mmol, 1.1 equiv.) was added dropwise. The resutlting yellow solution was stirred for 40 min at the same temperature. Benzaldehyde (117 mg, 1.1 mmol, 1.1 equiv.) in THF (1 mL) was added next. After 1 h, acetic anhydride (204 mg, 2 mmol, 2 equiv.) was added slowly. The mixture was slowly warmed to room temperature and run overnight. After quenching with saturated aqueous NH_4Cl, the mixture was extracted with ethyl acetate 3 times. The organic phase was dried over Na_2SO_4, filtered and concentrated under reduced pressure. The crude acetoxy sulfone was obtained in 528 mg (98% yield) and used for the next step without further purification. Next, to a solution of a mixture of crude acetoxy sulfone (102 mg, 0.2 mmol, 1 equiv) in THF (0.8 mL) methanol (2 mL) at room temperature, was added Na_2HPO_4 (114 mg, 0.8 mmol, 4 equiv.) and 5% Na/Hg (402 mg). The suspension was stirred for 1.5 h. The mixture was then concentrated and purified by flash column chromatography to afford separable *E*/*Z* isomers **68’** (*E* isomer, 46.4 mg, d.r. 5:1, 68%) and **68”** (*Z* isomer, 15.6 mg, d.r. 3.7:1, 23%) both as white solid.

(*E*)-3-([1,1'-biphenyl]-4-ylmethyl)-4-styryltetrahydrofuran, 68’: ^{1}H NMR (600 MHz, $CDCl_3$): *cis* isomer δ 7.62 (d, *J* = 7.6 Hz, 2H), 7.54 (d, *J* = 8.2 Hz, 2H), 7.49 – 7.41 (m, 4H), 7.40 – 7.32 (m, 3H), 7.31 – 7.24 (m, 3H), 6.49 (d, *J* = 16.1 Hz, 1H), 6.33 (dd, *J* = 15.7, 9.2 Hz, 1H), 4.08 (dd, *J* = 8.9, 6.5 Hz, 1H), 3.97 (t, *J* = 7.8 Hz, 1H), 3.89 (dd, *J* = 8.3, 4.6 Hz, 1H), 3.72 (t, *J* = 7.9 Hz, 1H), 3.17 – 3.09 (m, 1H), 2.89 (dd, *J* = 13.8, 6.1 Hz, 1H), 2.81 – 2.72 (m, 1H), 2.64 (dd, *J* = 13.7, 9.3 Hz, 1H); *trans* isomer δ 7.58 (d, *J* = 7.6 Hz, 2H), 7.33 (t, *J* = 7.6 Hz, 2H), 6.45 (d, *J* = 15.7 Hz, 1H), 6.09 (dd, *J* = 15.7, 9.0 Hz, 1H), 4.14 (t, *J* = 8.2 Hz, 1H), 4.03 (t, *J* = 8.0 Hz, 1H), 4.20 – 4.13 (m, 1H), 3.70 – 3.63 (m, 2H), 3.00 (dd, *J* = 13.8, 5.4 Hz, 1H), 2.67 (dd, *J* = 13.8, 9.2 Hz, 1H), 2.48 – 2.40 (m, 1H), the remaining resonances are insufficiently resolved from those of the cis isomer to be reported.

^{13}C NMR (151 MHz, $CDCl_3$): *cis* isomer δ 141.0, 139.9, 139.1, 137.3, 132.2, 129.2, 128.8, 128.7, 127.9, 127.5, 127.2 (2C), 127.1, 126.3, 73.2, 72.3, 46.4, 45.6, 34.3; *trans* isomer δ 139.4, 139.3, 137.2, 131.8, 129.6, 128.8, 128.7,

127.5, 127.3, 127.1, 126.2, 73.7, 73.3, 49.9, 47.9, 37.7. the remaining resonances are insufficiently resolved from those of the cis isomer to be reported.

IR (ATR) ν 3027, 2924, 2853, 1597, 1486, 1447, 1046, 967, 912, 850, 815, 749, 691 cm^{-1};

HRMS (ESI) for $C_{25}H_{24}ONa^+$ $(M+Na)^+$: 363.1719; Found : 363.1715.

(Z)-3-([1,1'-biphenyl]-4-ylmethyl)-4-styryltetrahydrofuran, 68'': ^{1}H NMR (600 MHz, $CDCl_3$): *cis* isomer δ 7.57 (d, *J* = 7.3 Hz, 2H), 7.49 (d, *J* = 8.1 Hz, 2H), 7.46 – 7.42 (m, 2H), 7.37 – 7.31 (m, 3H), 7.28 – 7.25 (m, 1H), 7.23 (d, *J* = 7.8 Hz, 4H), 6.68 (d, *J* = 11.7 Hz, 1H), 5.79 (t, *J* = 11.1 Hz, 1H), 4.04 (t, *J* = 7.4 Hz, 1H), 3.88 (dd, *J* = 9.1, 6.5 Hz, 1H), 3.75 (dd, *J* = 8.9, 5.5 Hz, 1H), 3.70 (dd, *J* = 8.4, 6.4 Hz, 1H), 3.55 – 3.48 (m, 1H), 2.93 (dd, *J* = 13.2, 4.8 Hz, 1H), 2.74 – 2.61 (m, 2H); *trans* isomer δ 7.20 (d, *J* = 7.4 Hz, 2H), 7.12 (d, *J* = 8.1 Hz, 2H), 6.57 (d, *J* = 11.3 Hz, 1H), 5.56 (t, *J* = 10.7 Hz, 1H), 4.13 (t, *J* = 8.1 Hz, 1H), 3.98 (t, *J* = 7.8 Hz, 1H), 3.62 (t, *J* = 8.3 Hz, 1H), 3.57 (t, *J* = 8.3 Hz, 1H), 3.20 – 3.12 (m, 1H), 2.85 (dd, *J* = 13.8, 5.5 Hz, 1H), 2.54 (dd, *J* = 13.8, 9.2 Hz, 1H), 2.42 – 2.35 (m, 1H), the remaining resonances are insufficiently resolved from those of the cis isomer to be reported.

^{13}C NMR (151 MHz, $CDCl_3$): *cis* isomer δ 141.1, 140.0, 139.1, 137.2, 132.1, 131.5, 131.5, 129.9, 129.3, 128.9, 128.7, 128.6, 128.4, 127.3, 127.2, 127.1, 127.1, 74.0, 72.5, 45.5, 41.1, 34.2; *trans* isomer δ 139.2, 73.4 (2C), 48.9, 44.4, 37.4. the remaining resonances are insufficiently resolved from those of the cis isomer to be reported.

IR (ATR) ν 3023, 2925, 2853, 2324, 2093, 1941, 1599, 1486, 1445, 1049, 914, 856, 802, 696 cm^{-1};

HRMS (ESI) for $C_{25}H_{24}ONa^+$ $(M+Na)^+$: 363.1719; Found : 363.1715.

4-(2,3-Dimethylbut-2-en-1-yl)-1,1'-biphenyl, **69**: To a flame-dried 15 mL vial equipped with a stir bar was added

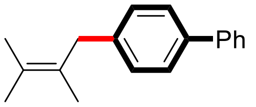

$PdCl_2(MeCN)_2$ (5.2 mg, 0.02 mmol, 10 mol%) and 1,2-bis(diphenylphosphino)ethane (8.0 mg, 0.02 mmol, 10 mol%) under argon, 0.5 mL DCM was added and the reaction mixture was stirred for 15 mins. The solvent was removed by sparging with argon, then a solution of 4-(2,3-dimethyl-4-(phenylsulfonyl)but-2-en-1-yl)-1,1'-biphenyl (75.3 mg, 0.2 mmol, 1 equiv.) in 2 mL THF was added. Fllowed by dropwise addition of the Super-Hydride® solution (0.4 mL, 1 M, 2 equiv.). The reaction was stirred for 1.5 h, and then NaOH (2.5%) was added. The mixture was extracted with DCM, then dried over anhydrous Na_2SO_4. Afterwards the reaction mixture was concentrated in vacuo and purified with column chromatography to afford the desired product **69** as a white solid in 97% yield (46.0 mg).

^{1}H NMR (400 MHz, $CDCl_3$): δ 7.62 (d, *J* = 7.6 Hz, 2H), 7.54 (d, *J* = 7.9 Hz, 2H), 7.45 (t, *J* = 7.6 Hz, 2H), 7.35 (t, *J* = 7.4 Hz, 1H), 7.26 (d, *J* = 7.8 Hz, 2H), 3.47 (s, 2H), 1.86 (s, 3H), 1.81 (s, 3H), 1.68 (s, 3H).

^{13}C NMR (101 MHz, $CDCl_3$): δ 141.3, 140.3, 138.8, 129.0, 128.8, 127.1 (2C), 126.4, 126.1, 39.9, 20.9, 20.8, 18.6.

IR (ATR) ν 3833, 3388, 2984, 2912, 2727, 2327, 2109, 1907, 1804, 1730, 1667, 1601, 1480, 1442, 1256, 1194, 1152, 1116, 1073, 1005, 902, 815, 749, 693 cm^{-1};

HRMS (EI) for $C_{18}H_{20}^+$ $(M)^+$: 236.1560; Found : 236.1565.

References:

[1] A. U. Meyer, K. Straková, T. Slanina, B. König, *Chem. Eur. J.* **2016**, *22*, 8694-8699.

[2] J. R. Hwu, B.-L. Chen, S.-S. Shiao, *J. Org. Chem.* **1995**, *60*, 2448-2455.

[3] R. A. Gancarz, J. L. Kice, *J. Org. Chem.* **1981**, *46*, 4899-4906.

[4] R. Dowbenko, *Tetrahedron* **1964**, *20*, 1843-1858.

[5] C.-P. Chuang, *Synth. Commun.* **1992**, *22*, 3151-3158.

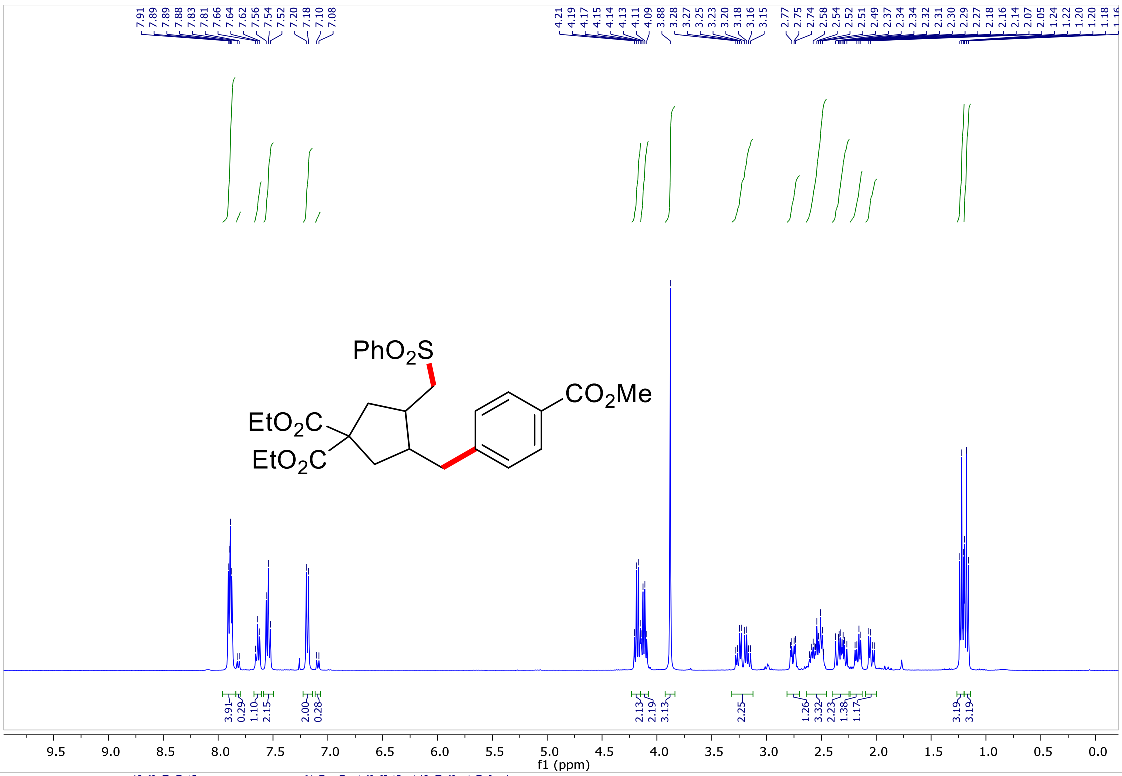
7.91 7.89 7.89 7.88 7.83 7.81 7.66 7.64 7.62 7.56 7.54 7.52 7.20 7.18 7.10 7.08
4.21 4.19 4.17 4.15 4.14 4.13 4.11 4.09 3.88 3.28 3.27 3.25 3.23 3.20 3.18 3.16 3.15
2.77 2.75 2.74 2.58 2.54 2.52 2.51 2.49 2.37 2.34 2.34 2.32 2.31 2.30 2.29 2.27 2.18 2.16 2.14 2.07 2.05 1.24 1.22 1.20 1.18 [illegible]
PhO$_2$S
CO$_2$Me
EtO$_2$C
EtO$_2$C
3.91 0.29 1.10 2.15 2.00 0.28
2.13 2.19 3.13 2.25 1.26 3.32 2.23 1.38 1.17 3.19 3.19
9.5 9.0 8.5 8.0 7.5 7.0 6.5 6.0 5.5 5.0 4.5 4.0 3.5 3.0 2.5 2.0 1.5 1.0 0.5 0.0
f1 (ppm)

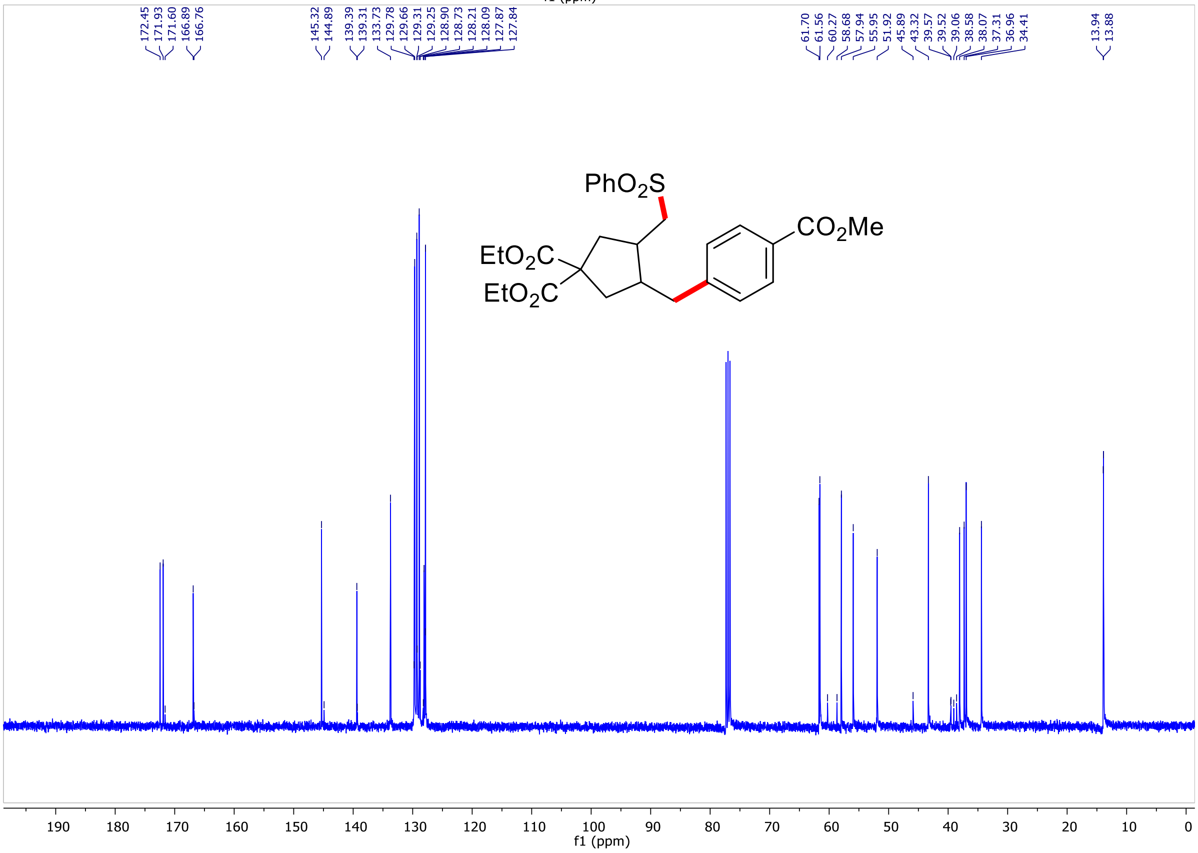
172.45 171.93 171.60 166.89 166.76
145.32 144.89 139.39 139.31 133.73 129.78 129.66 129.31 129.25 128.90 128.73 128.21 128.09 127.87 127.84
61.70 61.56 60.27 58.68 57.94 55.95 51.92 45.89 43.32 39.57 39.52 39.06 38.58 38.07 37.31 36.96 34.41
13.94 13.88
PhO$_2$S
CO$_2$Me
EtO$_2$C
EtO$_2$C
190 180 170 160 150 140 130 120 110 100 90 80 70 60 50 40 30 20 10 0
f1 (ppm)

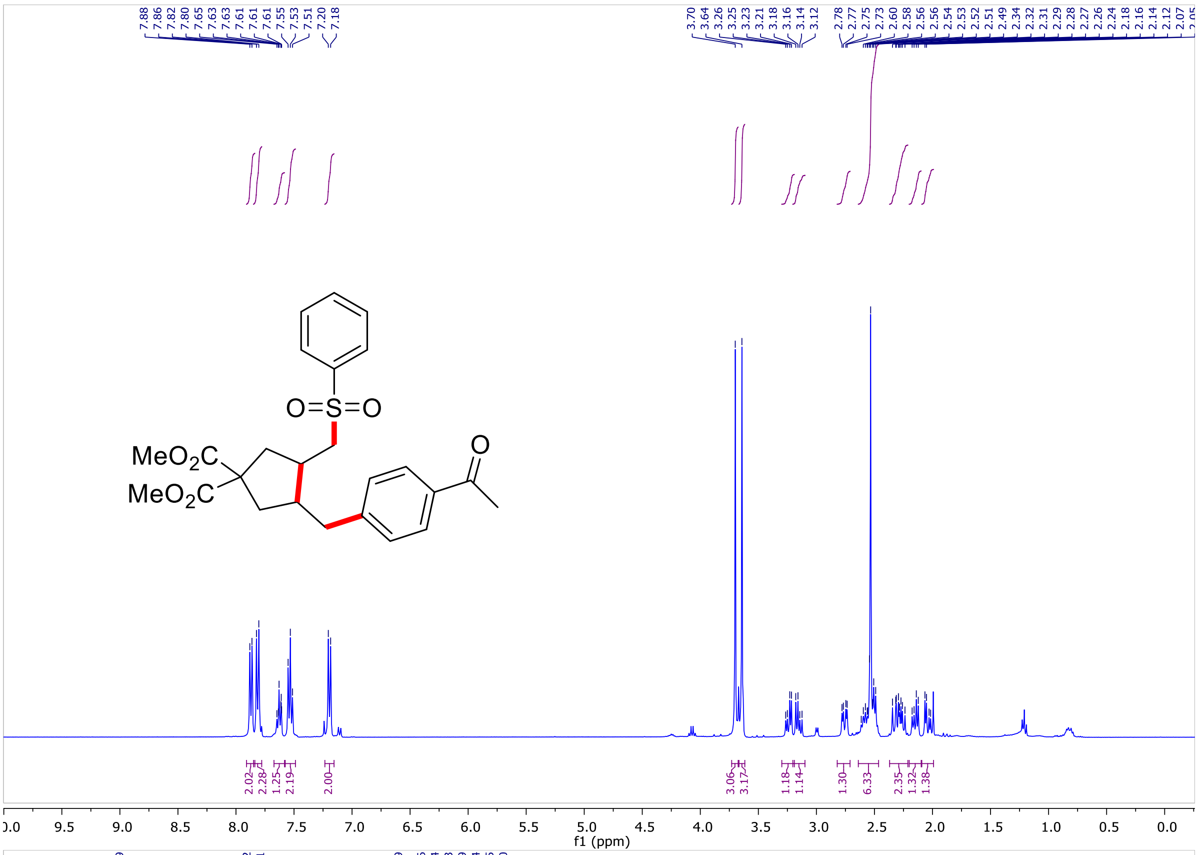
7.88
7.86
7.82
7.80
7.65
7.63
7.63
7.61
7.61
7.61
7.55
7.53
7.51
7.20
7.18
3.70
3.64
3.26
3.25
3.23
3.21
3.18
3.16
3.14
3.12
2.78
2.77
2.75
2.73
2.60
2.58
2.56
2.56
2.54
2.53
2.52
2.51
2.49
2.34
2.32
2.31
2.29
2.28
2.27
2.26
2.24
2.18
2.16
2.14
2.12
2.07
O=S=O
MeO2C
MeO2C
O
2.02
2.28
1.25
2.19
2.00
3.06
3.17
1.18
1.14
1.30
6.33
2.35
1.32
1.38
f1 (ppm)

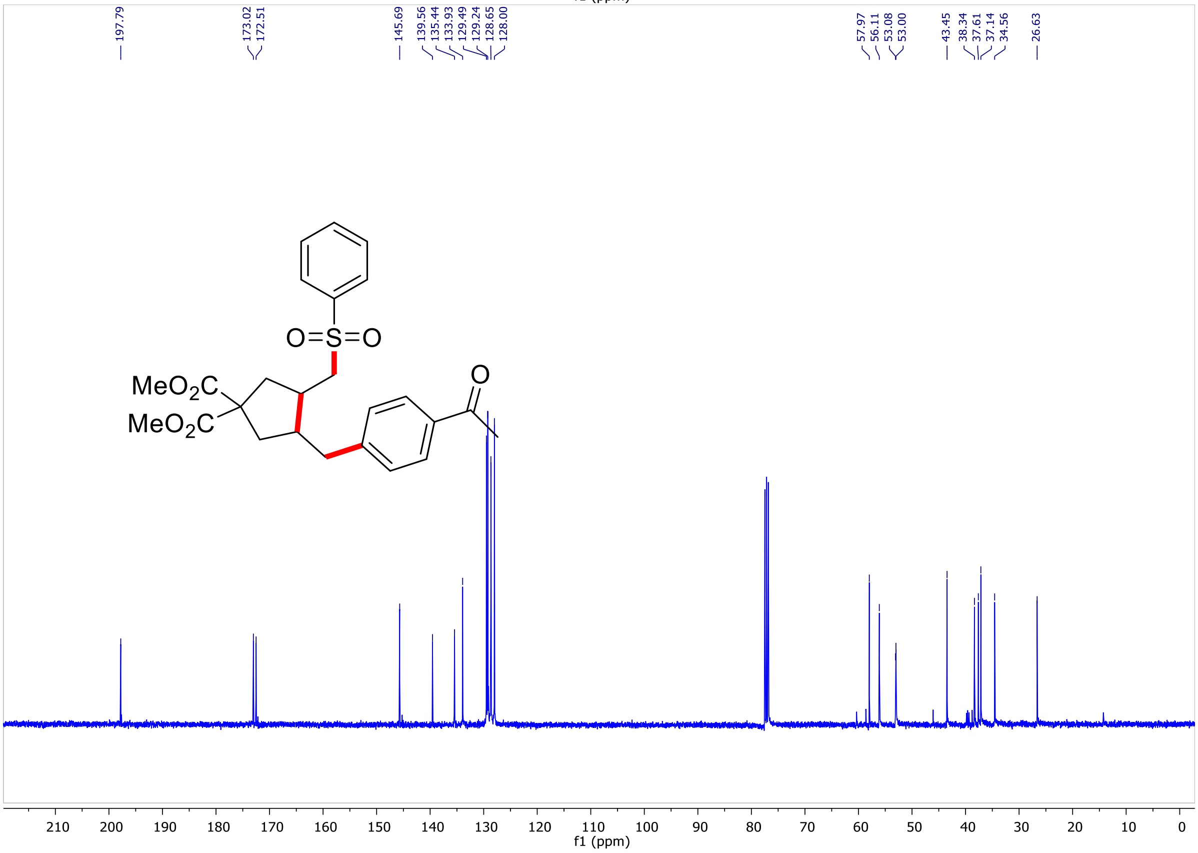
197.79
173.02
172.51
145.69
139.56
135.44
133.93
129.49
129.24
128.65
128.00
57.97
56.11
53.08
53.00
43.45
38.34
37.61
37.14
34.56
26.63
O=S=O
MeO2C
MeO2C
O
f1 (ppm)

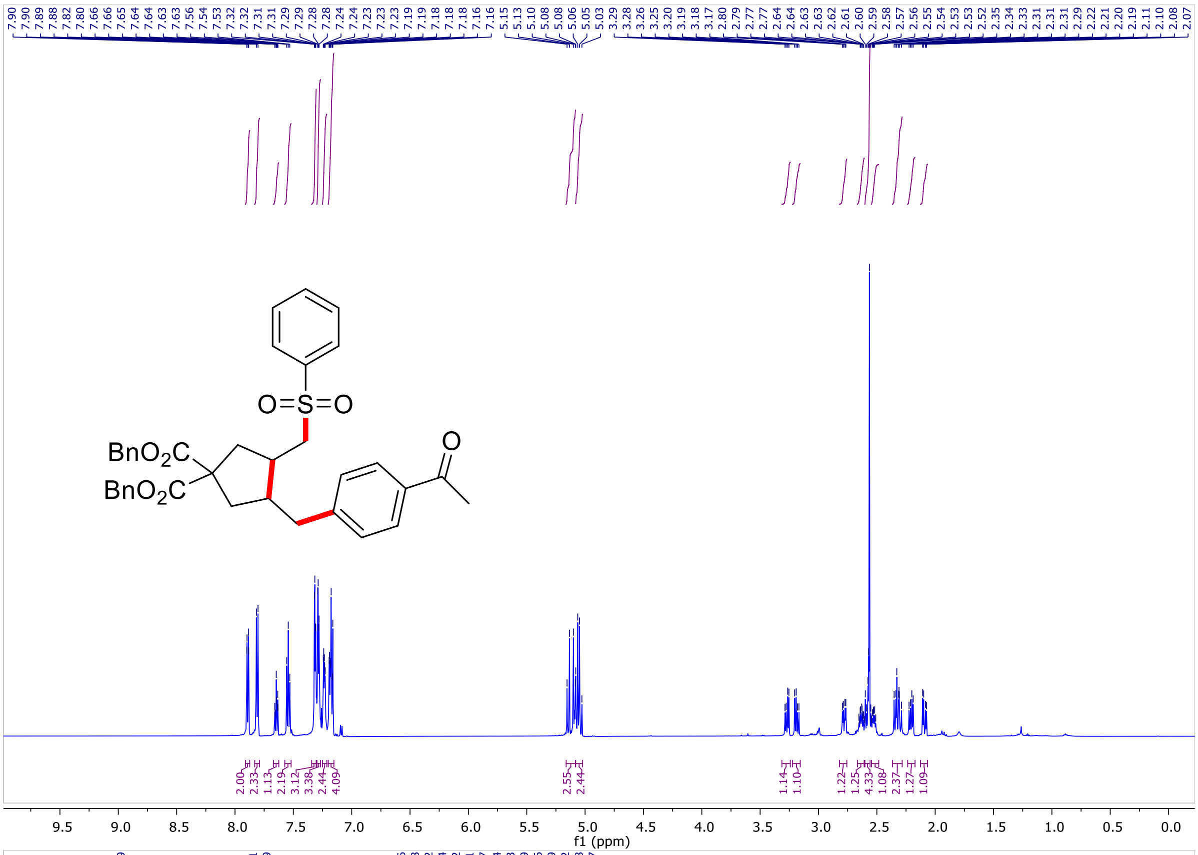

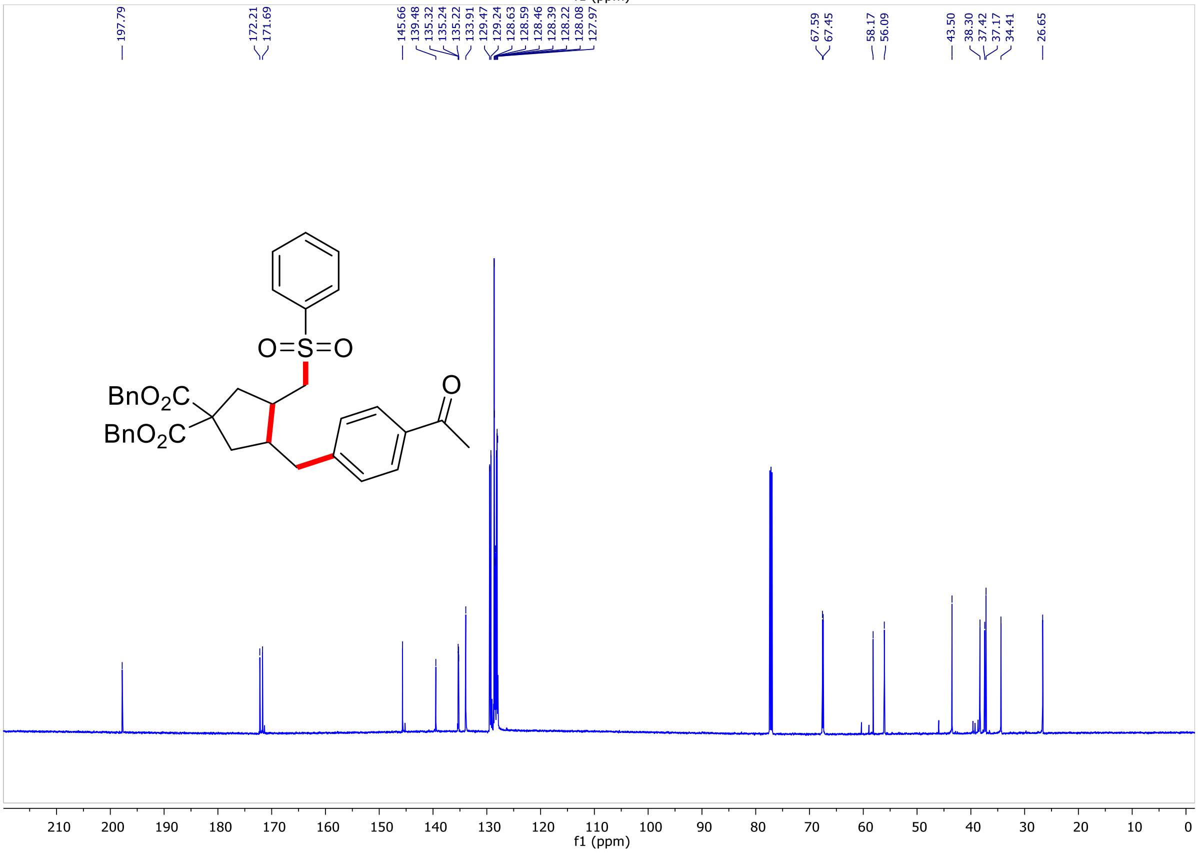

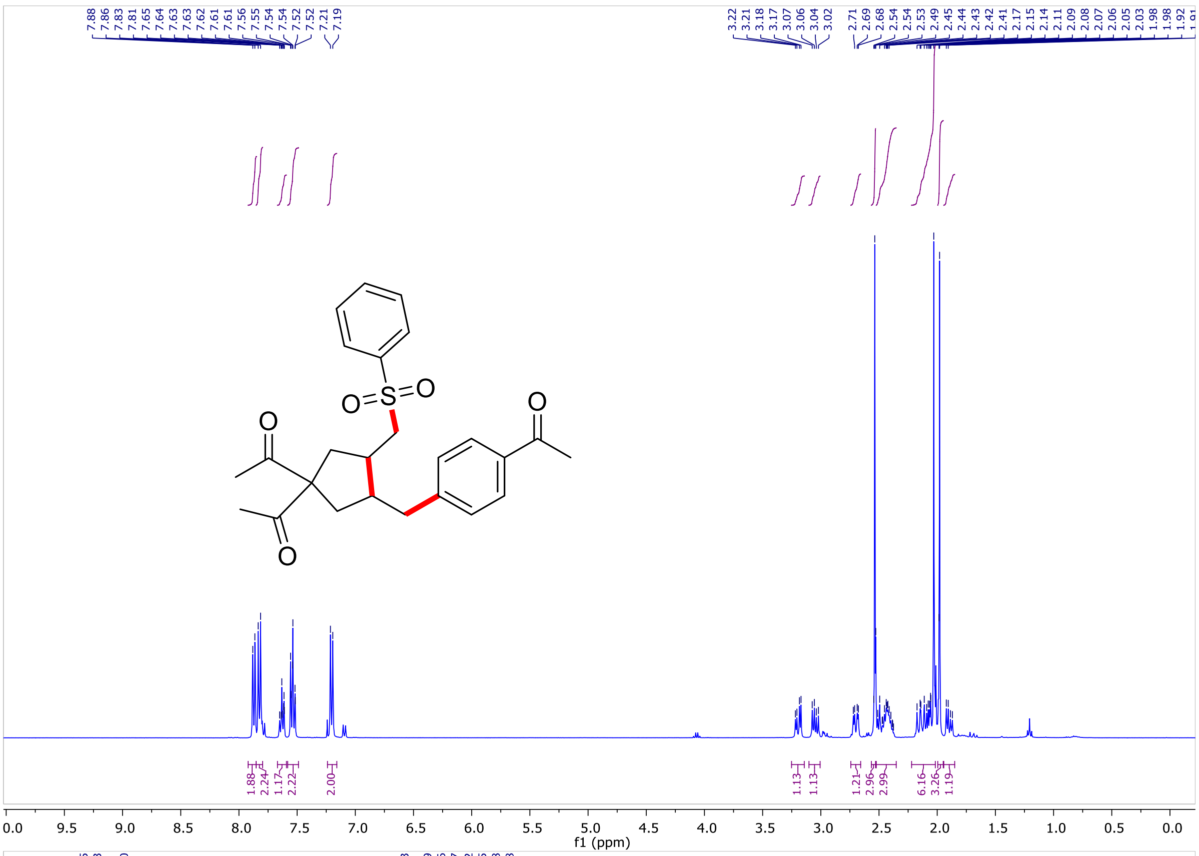

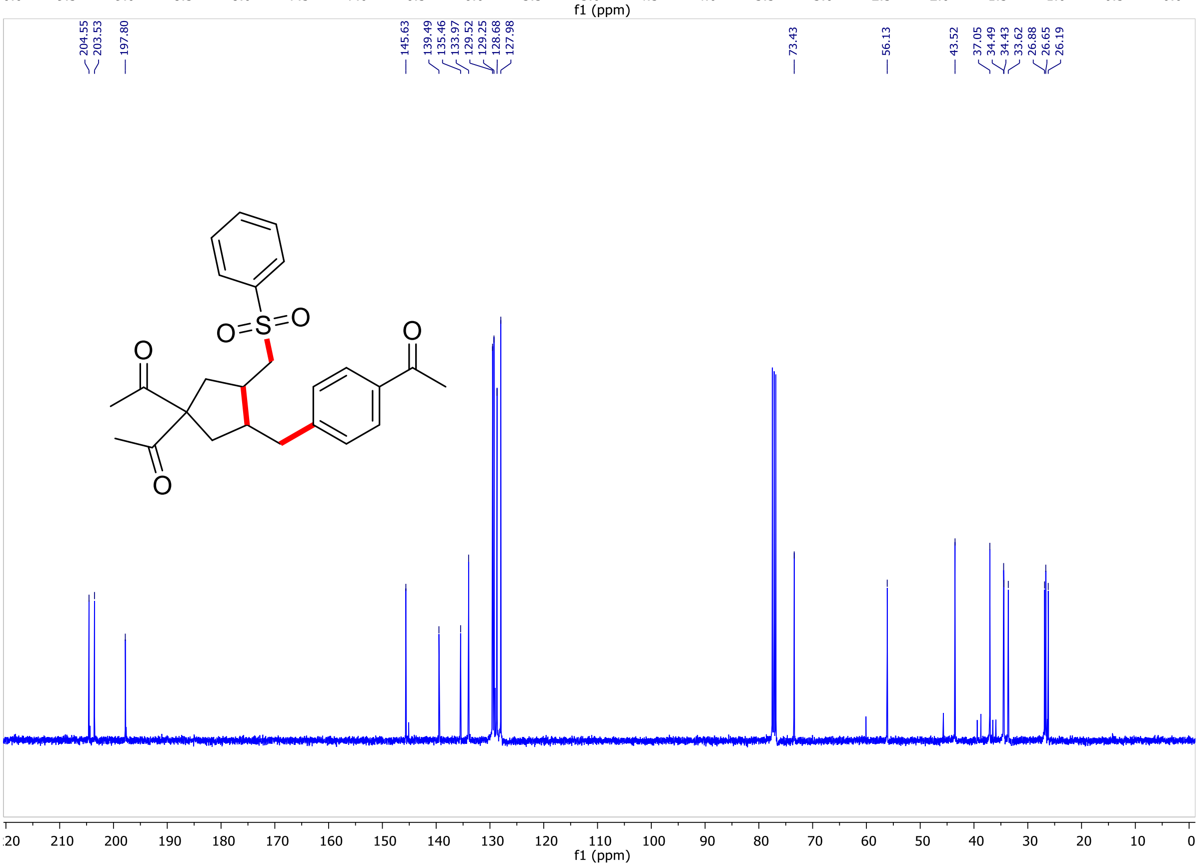

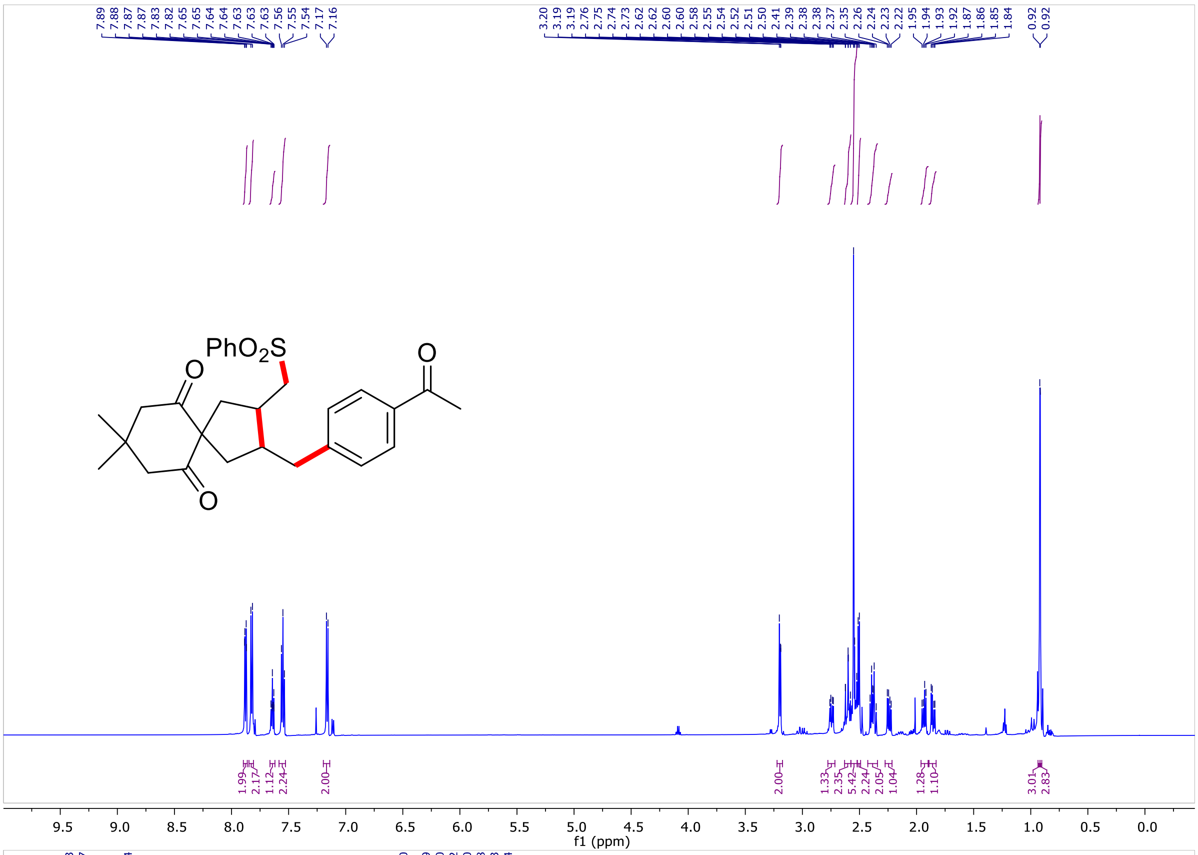
7.89 7.88 7.87 7.87 7.83 7.82 7.65 7.65 7.64 7.64 7.63 7.63 7.63 7.56 7.55 7.54 7.17 7.16
3.20 3.19 3.19 2.76 2.75 2.74 2.73 2.62 2.62 2.60 2.60 2.58 2.55 2.54 2.52 2.51 2.50 2.41 2.39 2.38 2.38 2.37 2.35 2.26 2.24 2.23 2.22 1.95 1.94 1.93 1.92 1.87 1.86 1.85 1.84 0.92 0.92
PhO2S
O
O
O
1.99 2.17 1.12 2.24 2.00 2.00 1.33 2.35 5.42 2.24 2.05 1.04 1.28 1.10 3.01 2.83
9.5 9.0 8.5 8.0 7.5 7.0 6.5 6.0 5.5 5.0 4.5 4.0 3.5 3.0 2.5 2.0 1.5 1.0 0.5 0.0
f1 (ppm)

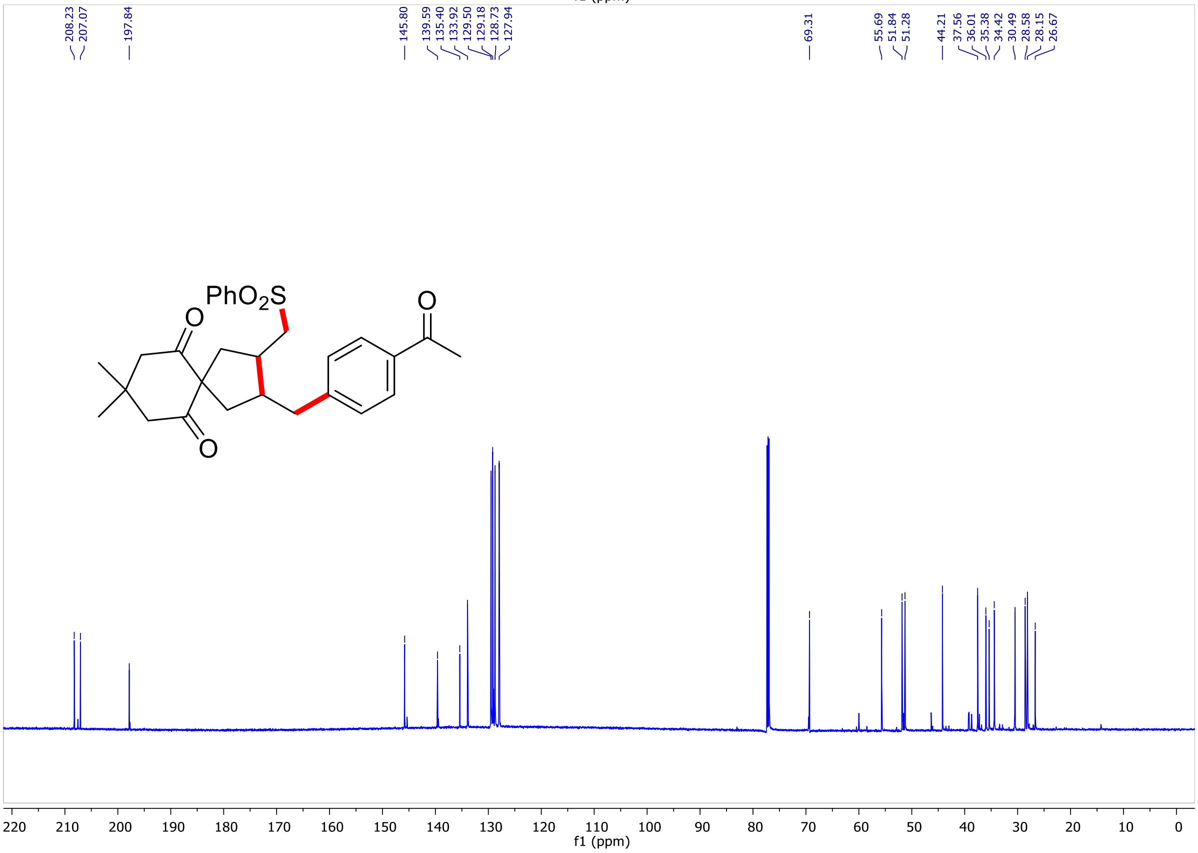
208.23 207.07 197.84 145.80 139.59 135.40 133.92 129.50 129.18 128.73 127.94 69.31 55.69 51.84 51.28 44.21 37.56 36.01 35.38 34.42 30.49 28.58 28.15 26.67
PhO2S
O
O
O
220 210 200 190 180 170 160 150 140 130 120 110 100 90 80 70 60 50 40 30 20 10 0
f1 (ppm)

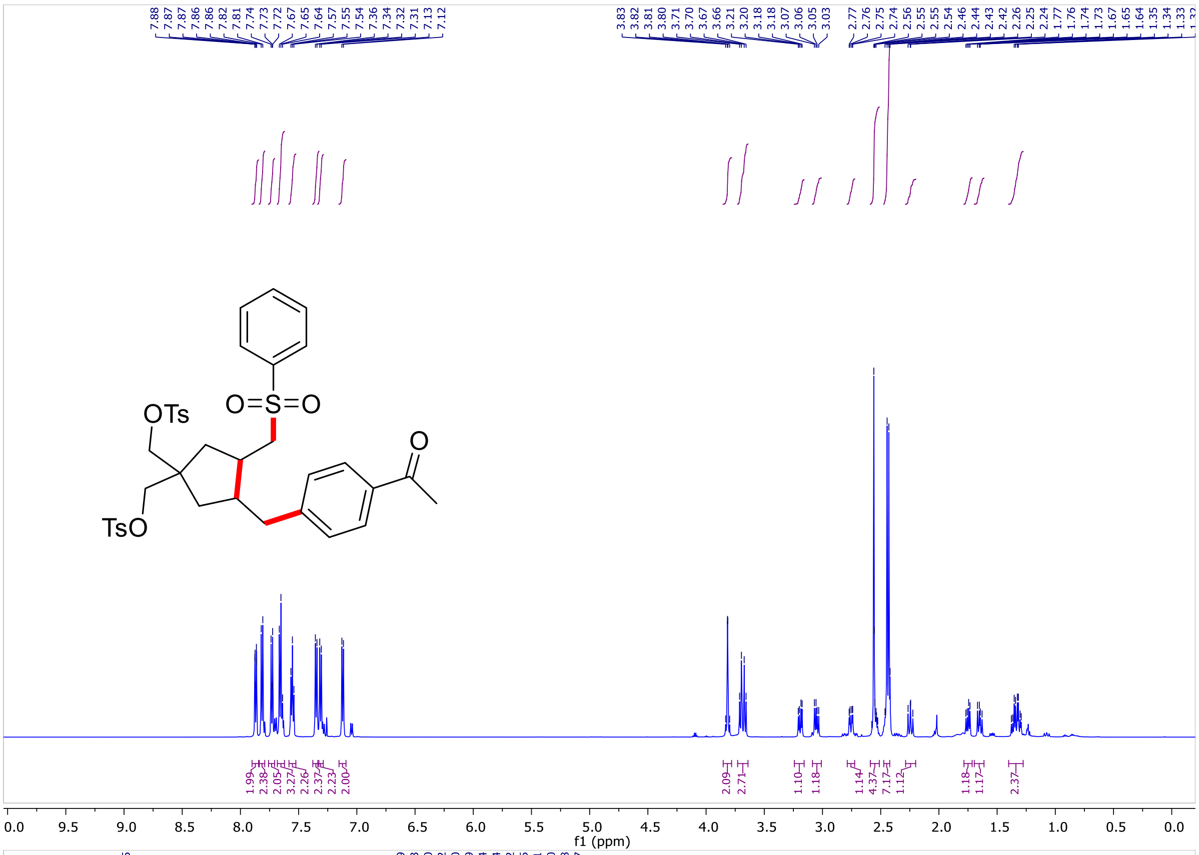
7.88 7.87 7.87 7.86 7.86 7.82 7.81 7.74 7.73 7.72 7.67 7.65 7.64 7.57 7.55 7.54 7.36 7.34 7.32 7.31 7.13 7.12
3.83 3.82 3.81 3.80 3.71 3.70 3.67 3.66 3.21 3.20 3.18 3.18 3.07 3.06 3.05 3.03 2.77 2.76 2.75 2.74 2.56 2.55 2.55 2.54 2.46 2.44 2.43 2.42 2.26 2.25 2.24 1.77 1.76 1.74 1.73 1.67 1.65 1.64 1.35 1.34 1.33
OTs
O=S=O
O
TsO
1.99 2.38 2.05 3.27 2.26 2.37 2.23 2.00 2.09 2.71 1.10 1.18 1.14 4.37 7.17 1.12 1.18 1.17 2.37
f1 (ppm)

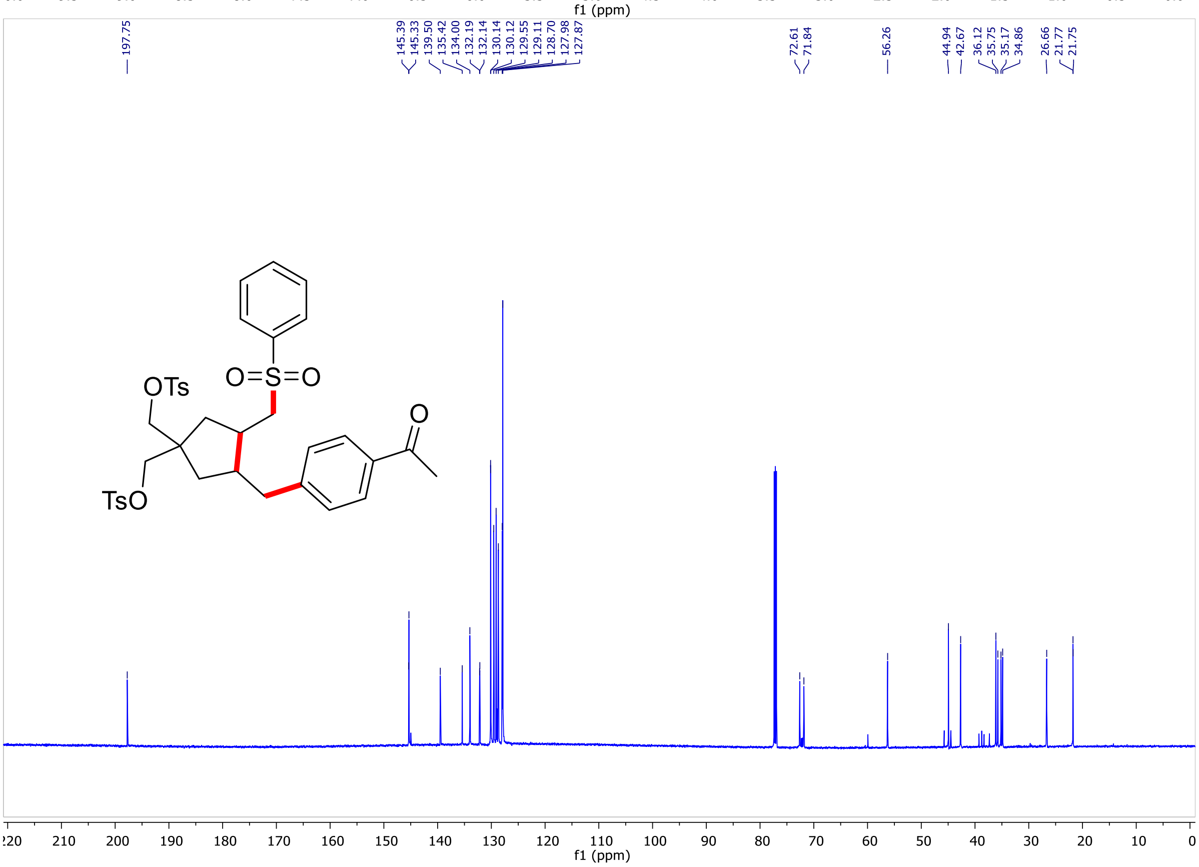
197.75
145.39 145.33 139.50 135.42 134.00 132.19 132.14 130.14 130.12 129.55 129.11 128.70 127.98 127.87
72.61 71.84
56.26
44.94 42.67 36.12 35.75 35.17 34.86 26.66 21.77 21.75
OTs
O=S=O
O
TsO
f1 (ppm)

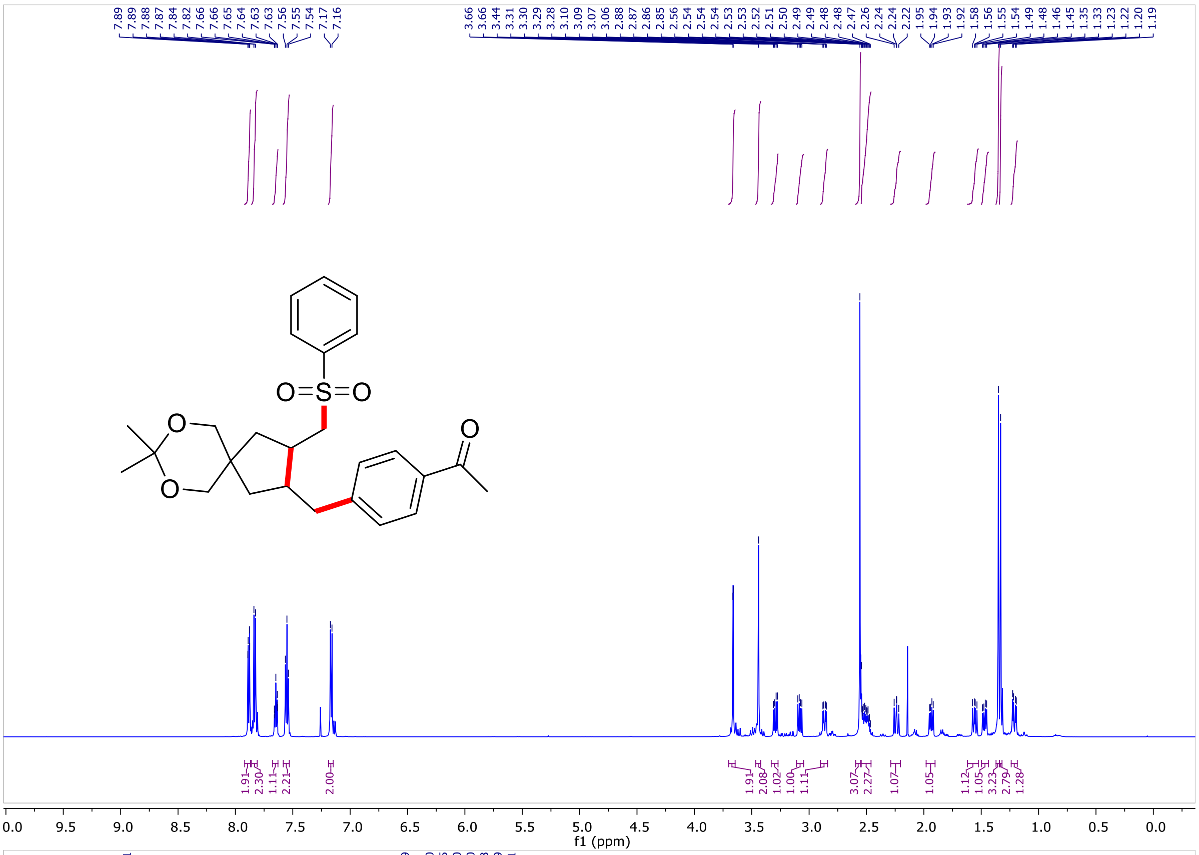
7.89 7.89 7.88 7.87 7.84 7.82 7.66 7.66 7.65 7.64 7.63 7.63 7.56 7.55 7.54 7.17 7.16
3.66 3.66 3.44 3.31 3.30 3.29 3.28 3.10 3.09 3.07 3.06 2.88 2.87 2.86 2.85 2.56 2.54 2.54 2.54 2.53 2.53 2.52 2.51 2.50 2.49 2.49 2.48 2.48 2.47 2.26 2.24 2.24 2.22 1.95 1.94 1.93 1.92 1.58 1.56 1.55 1.54 1.49 1.48 1.46 1.45 1.35 1.33 1.23 1.22 1.20 1.19
O=S=O
O
O
O
1.91 2.30 1.11 2.21 2.00
1.91 2.08 1.02 1.00 1.11 3.07 2.27 1.07 1.05 1.12 1.05 3.23 2.79 1.28
f1 (ppm)

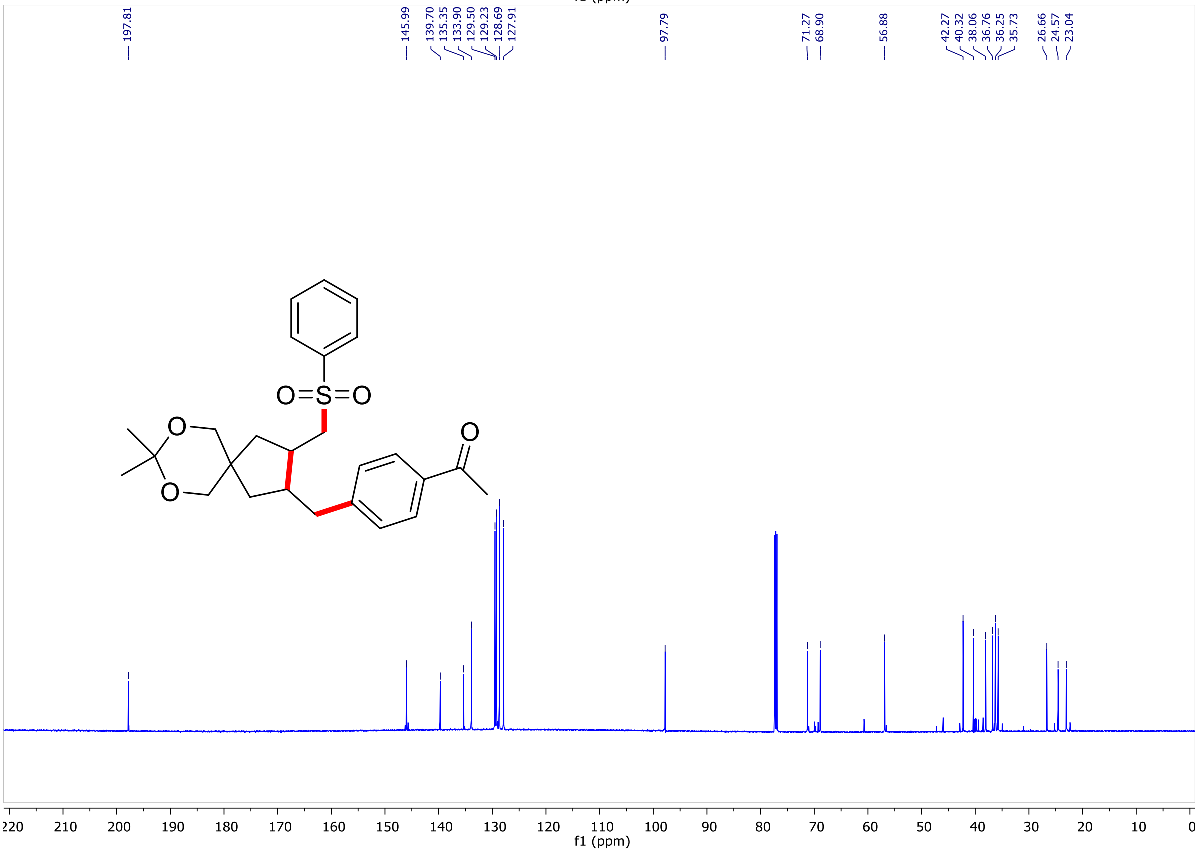
197.81
145.99 139.70 135.35 133.90 129.50 129.23 128.69 127.91
97.79
71.27 68.90
56.88
42.27 40.32 38.06 36.76 36.25 35.73
26.66 24.57 23.04
O=S=O
O
O
O
f1 (ppm)

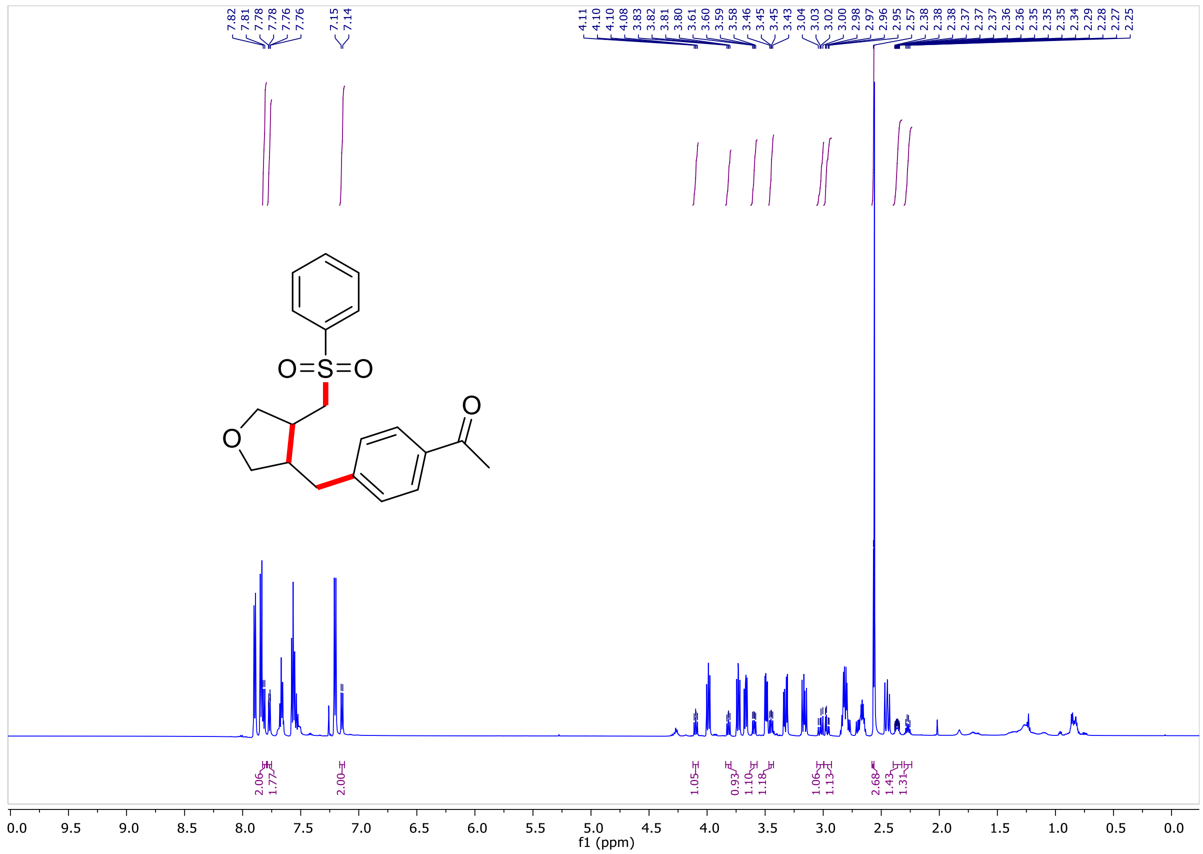

trans isomer

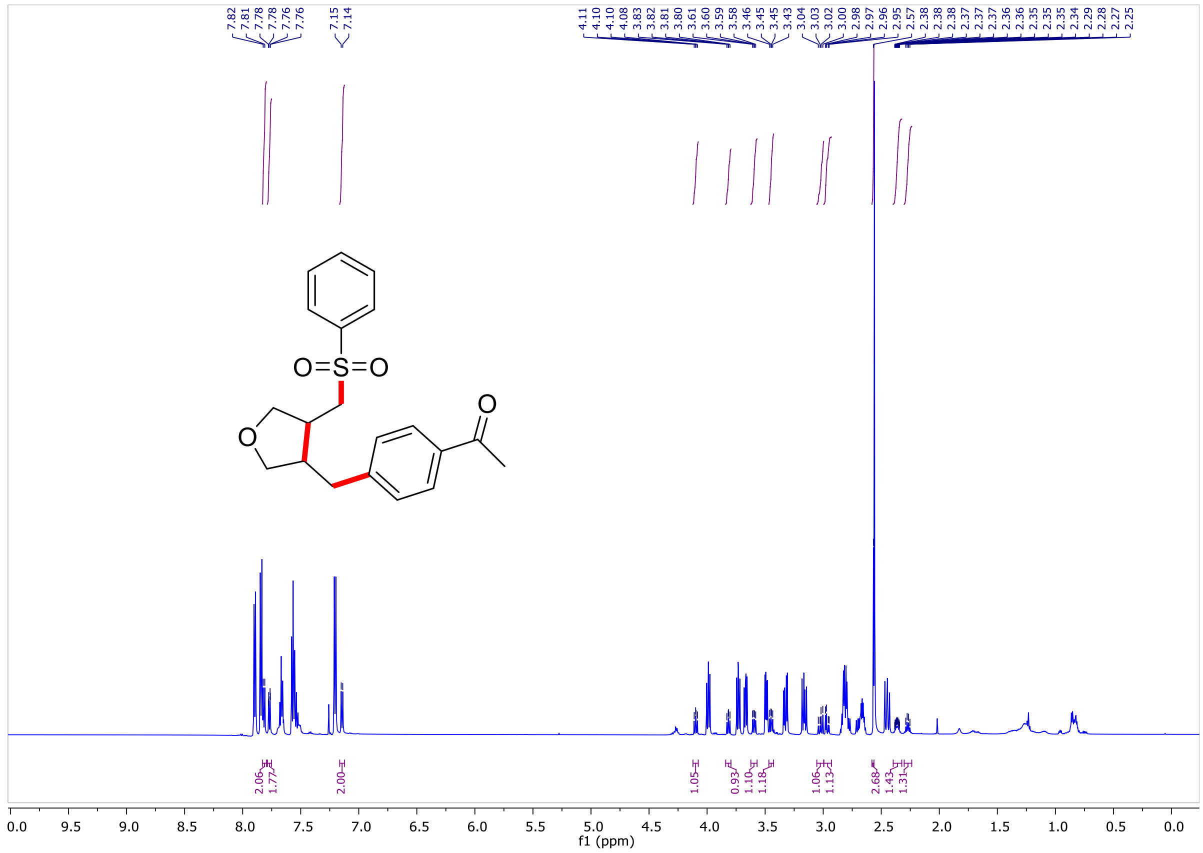

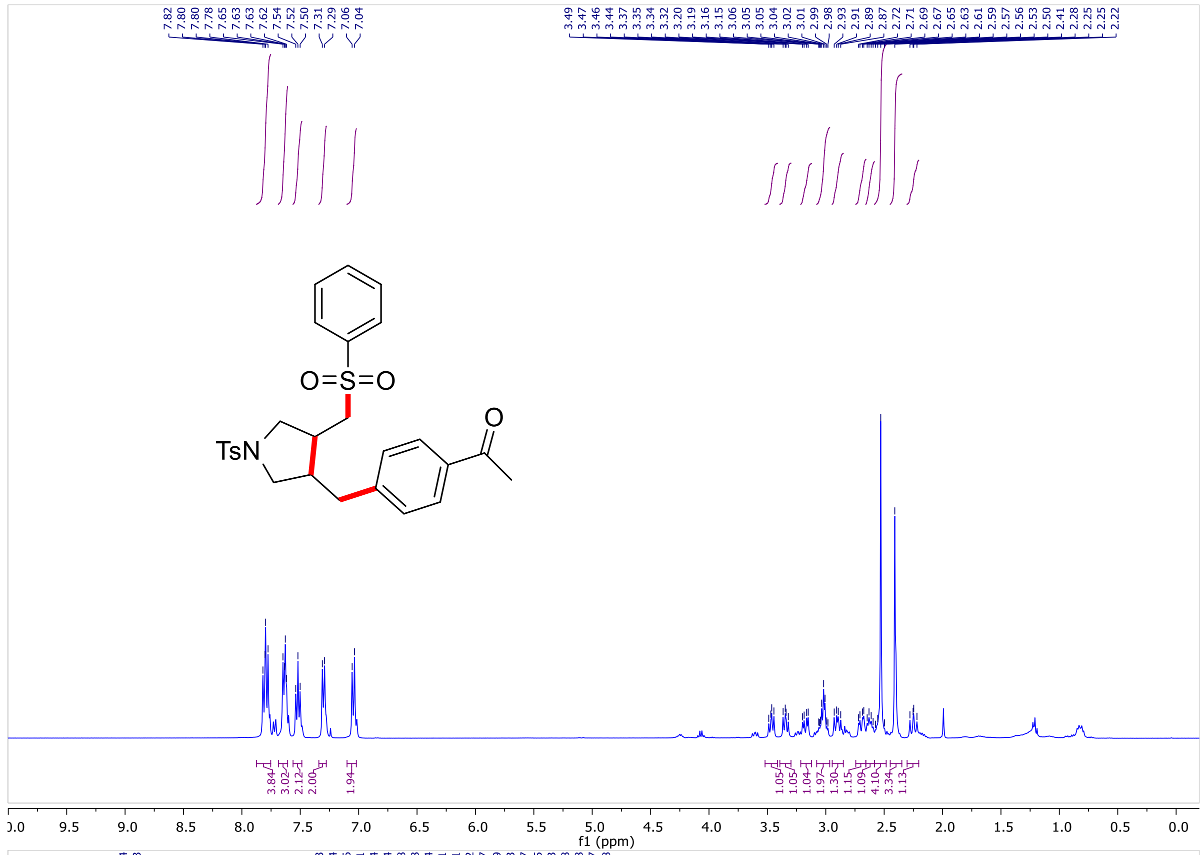
7.82 7.80 7.80 7.78 7.65 7.63 7.63 7.62 7.54 7.52 7.50 7.31 7.29 7.06 7.04
3.49 3.47 3.46 3.44 3.37 3.35 3.34 3.32 3.20 3.19 3.16 3.15 3.06 3.05 3.05 3.04 3.02 3.01 2.99 2.98 2.93 2.91 2.89 2.87 2.72 2.71 2.69 2.67 2.65 2.63 2.61 2.59 2.57 2.56 2.53 2.50 2.41 2.28 2.25 2.25 2.22
O
O=S=O
TsN
3.84 3.02 2.12 2.00 1.94
1.05 1.05 1.04 1.97 1.30 1.15 1.09 4.10 3.34 1.13
f1 (ppm)

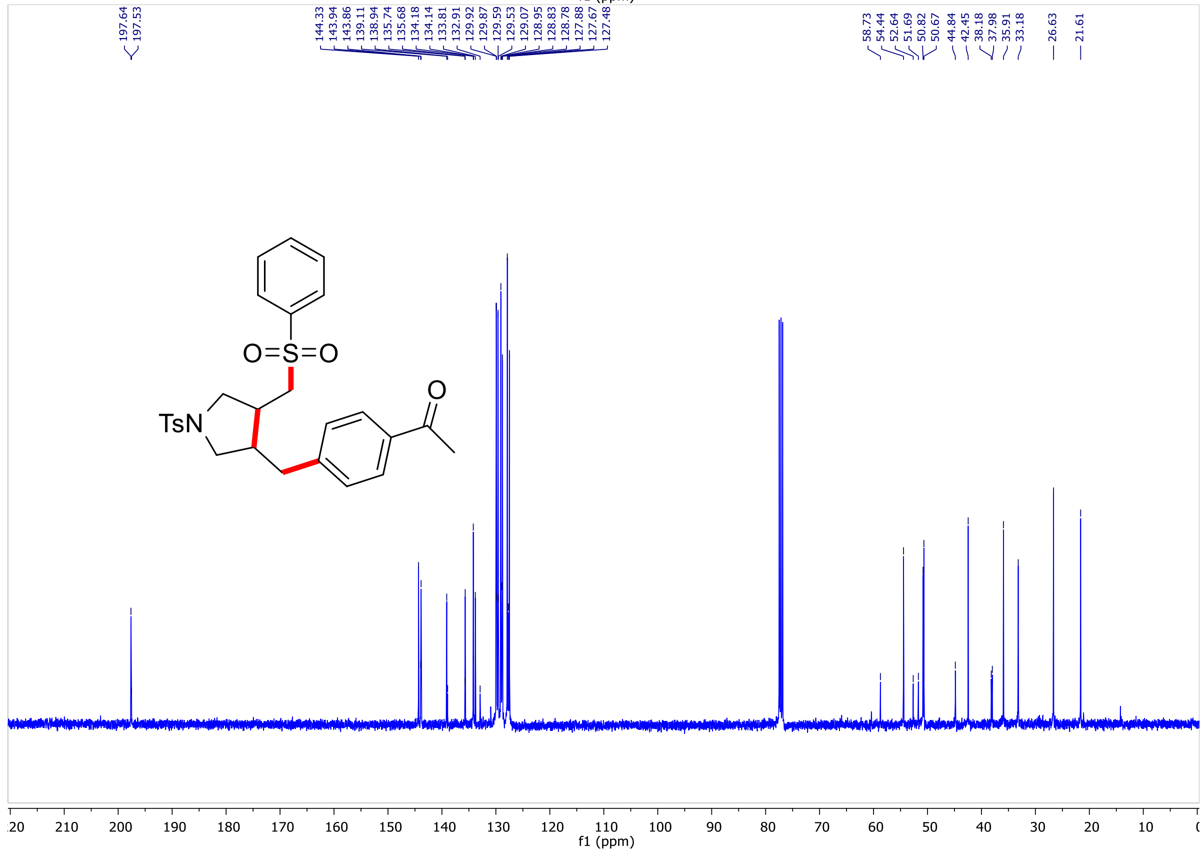
197.64 197.53
144.33 143.94 143.86 139.11 138.94 135.74 135.68 134.18 134.14 133.81 132.91 129.92 129.87 129.59 129.53 129.07 128.95 128.83 128.78 127.88 127.67 127.48
58.73 54.44 52.64 51.69 50.82 50.67 44.84 42.45 38.18 37.98 35.91 33.18 26.63 21.61
O
O=S=O
TsN
f1 (ppm)

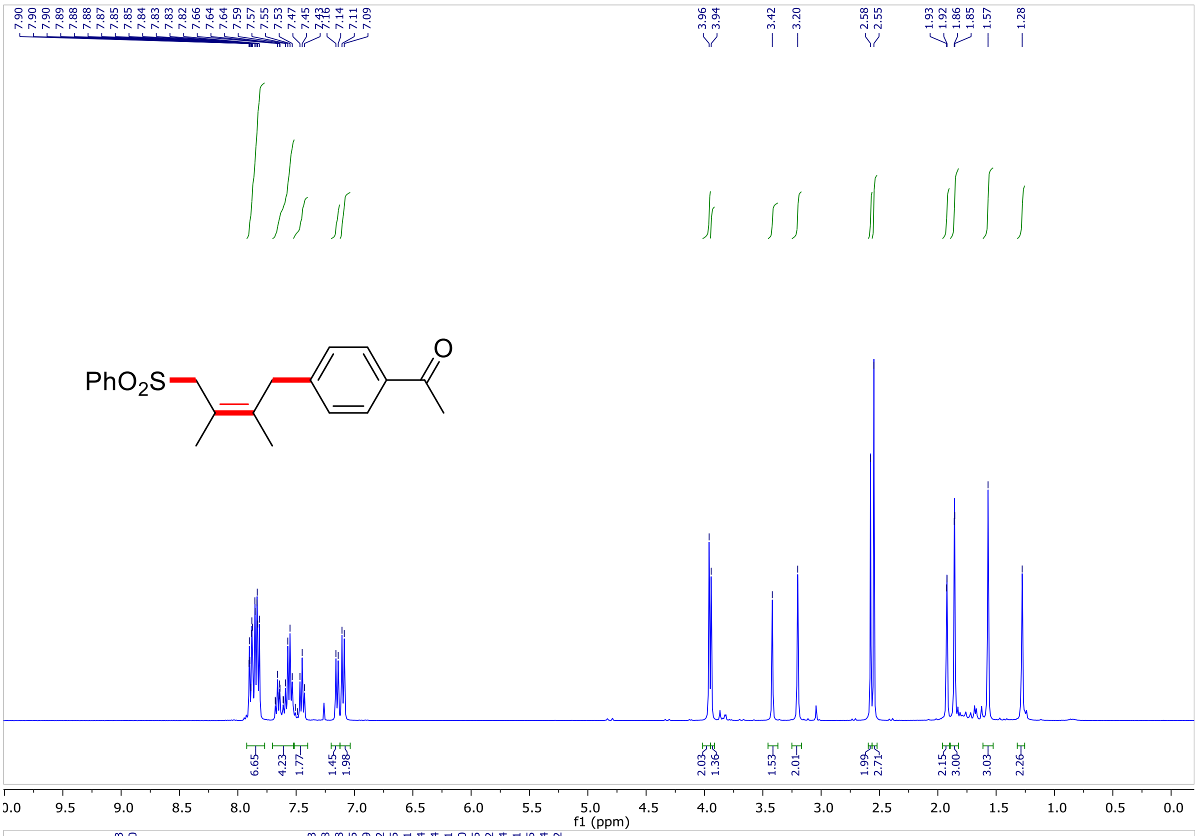
PhO2S
O
f1 (ppm)

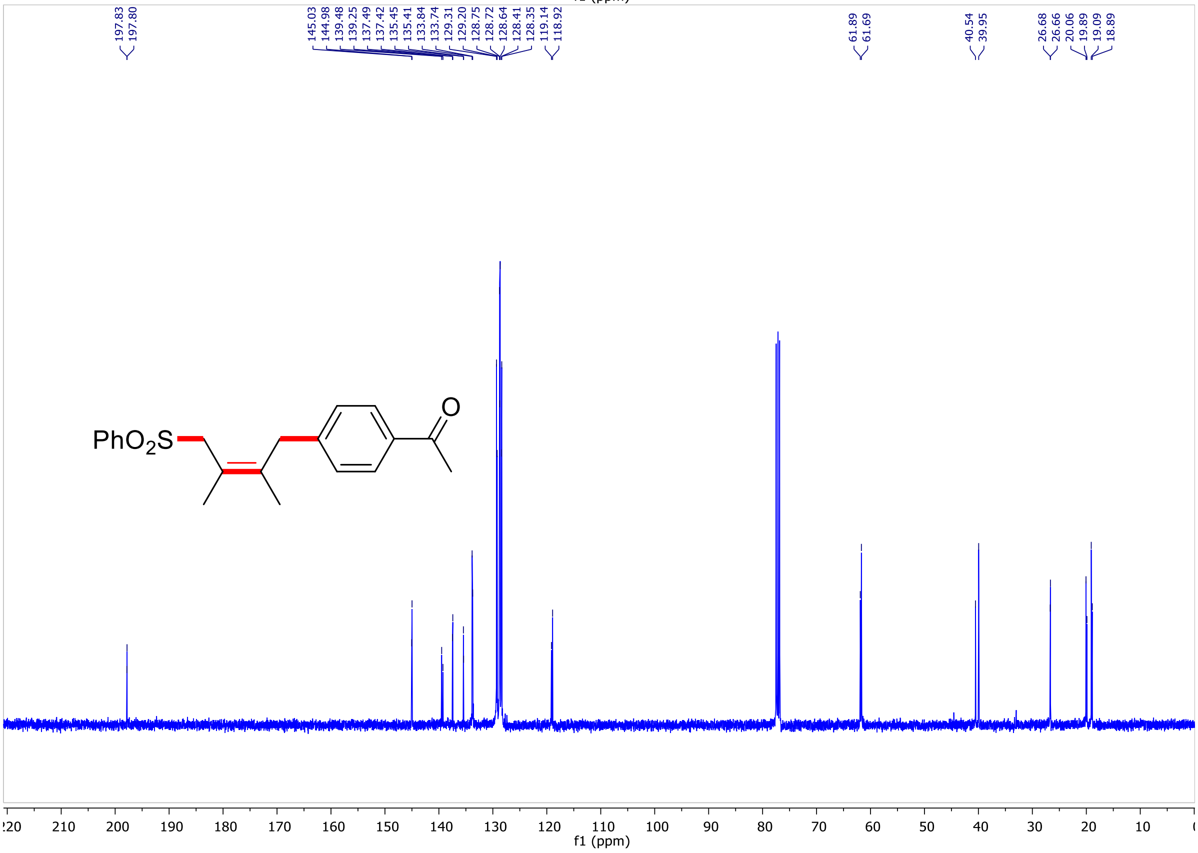
PhO2S
O
f1 (ppm)

NOESY

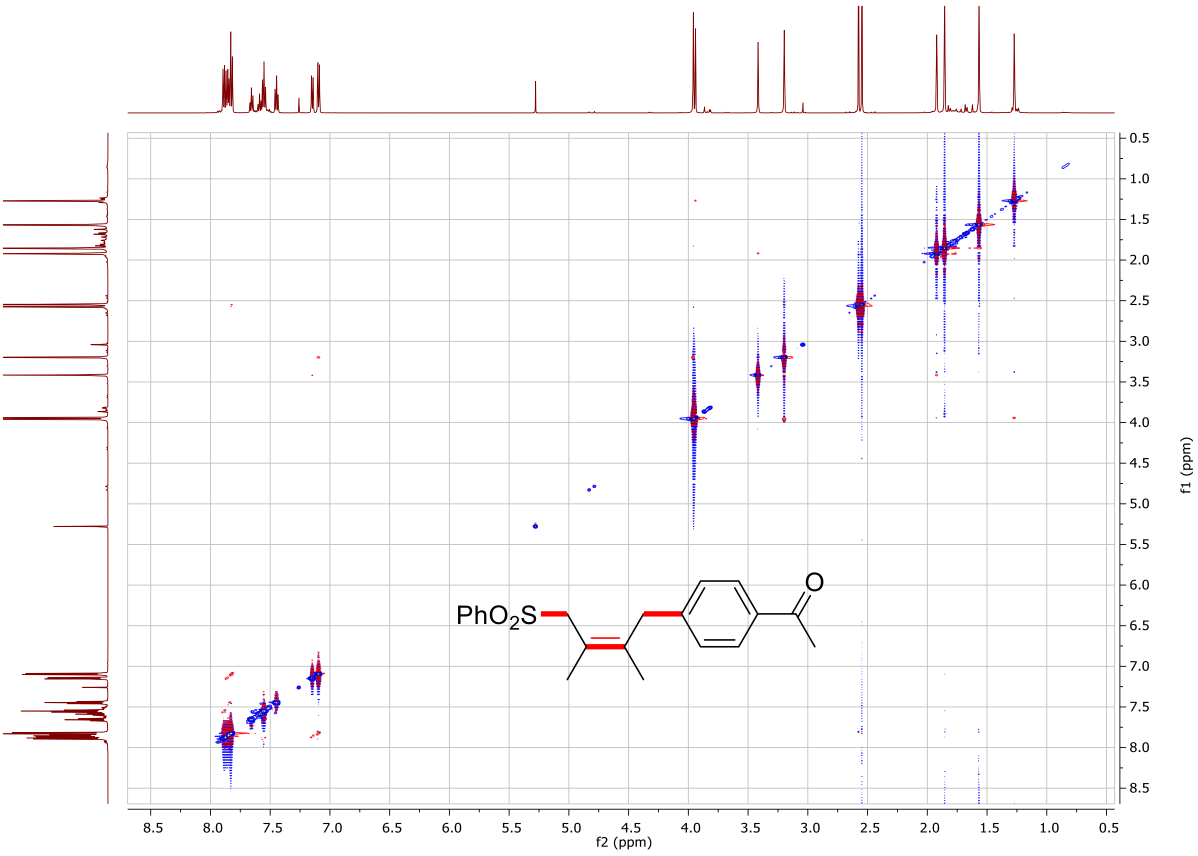

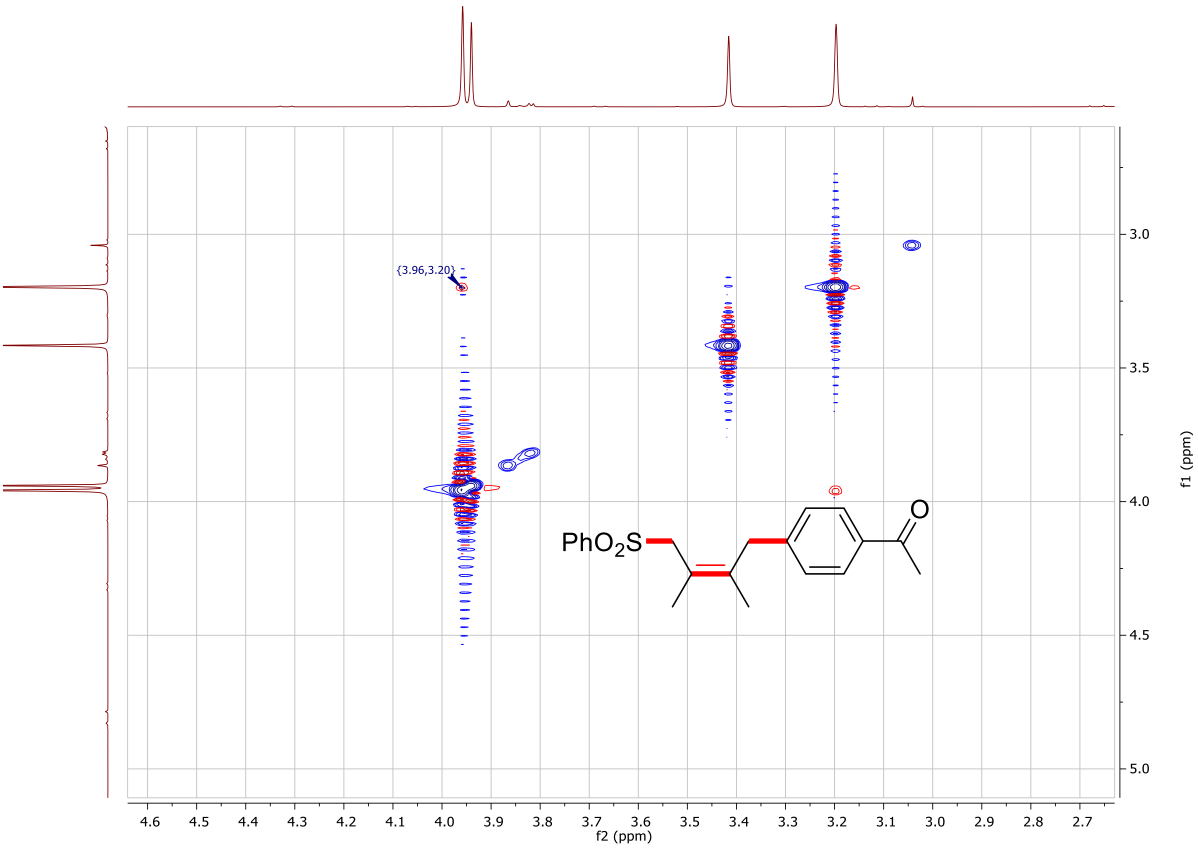

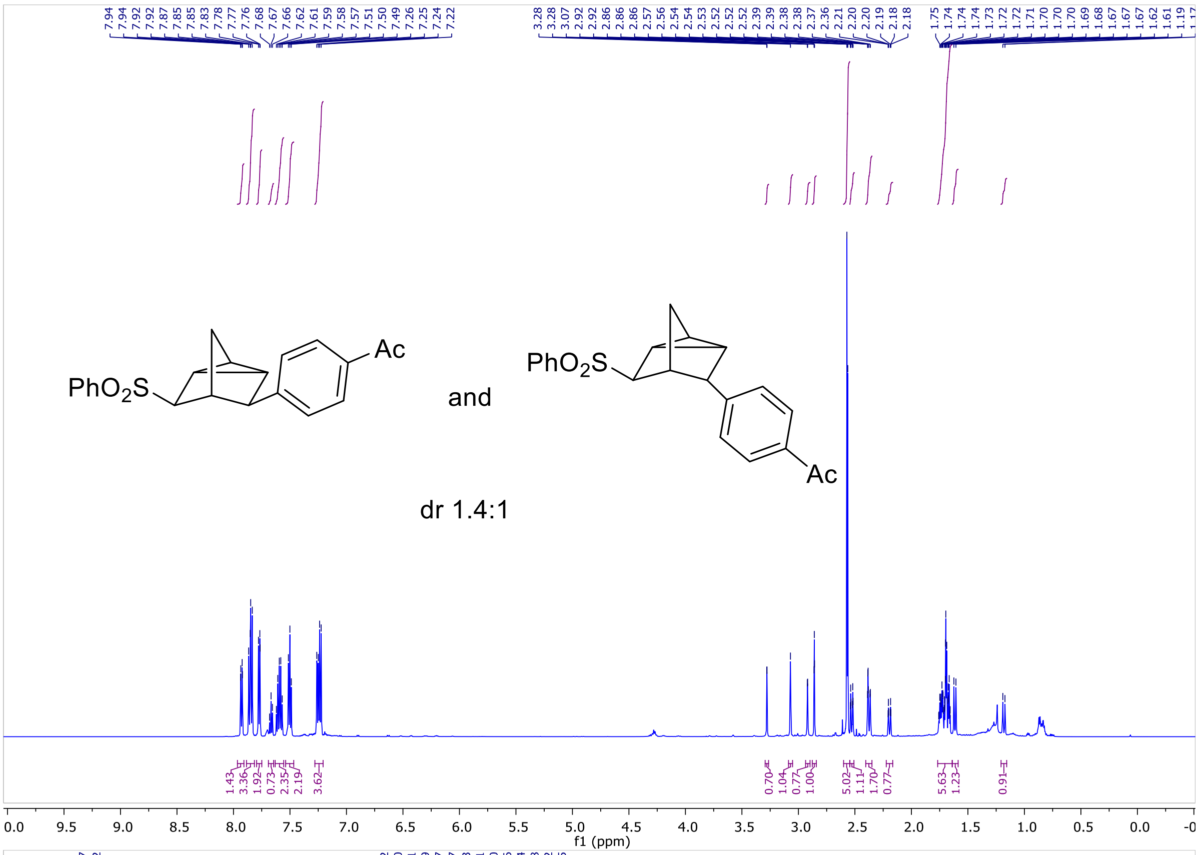
PhO2S
Ac
and
PhO2S
Ac
dr 1.4:1
f1 (ppm)

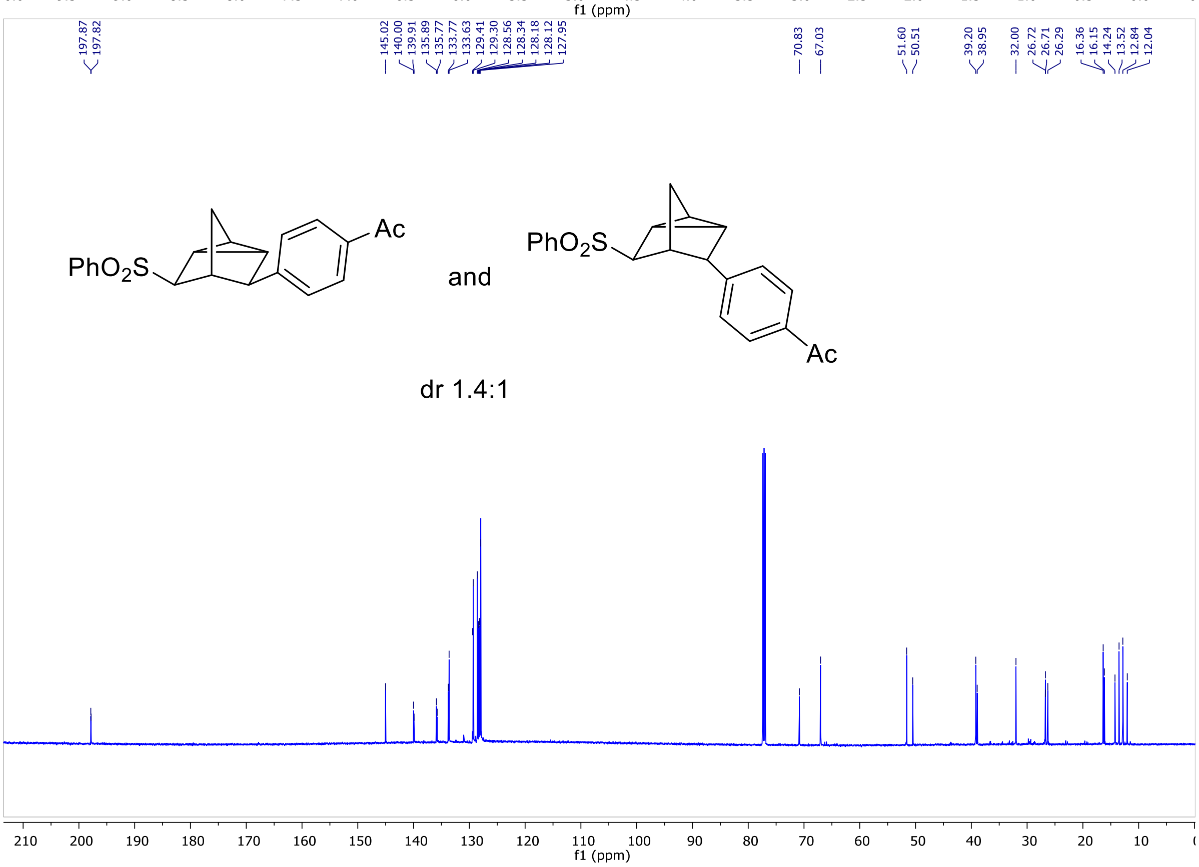
PhO2S
Ac
and
PhO2S
Ac
dr 1.4:1
f1 (ppm)

NOESY

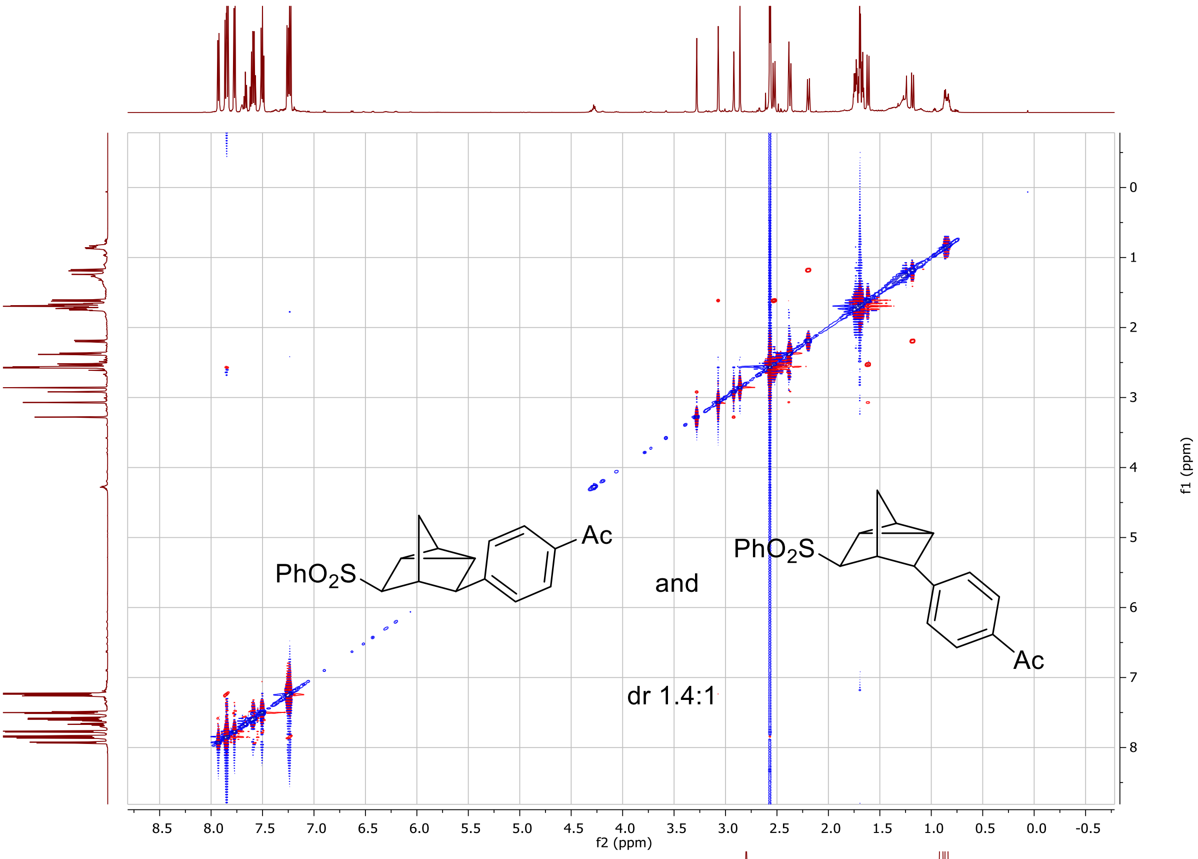

PhO2S
Ac
and
PhO2S
Ac
dr 1.4:1
f2 (ppm)
f1 (ppm)

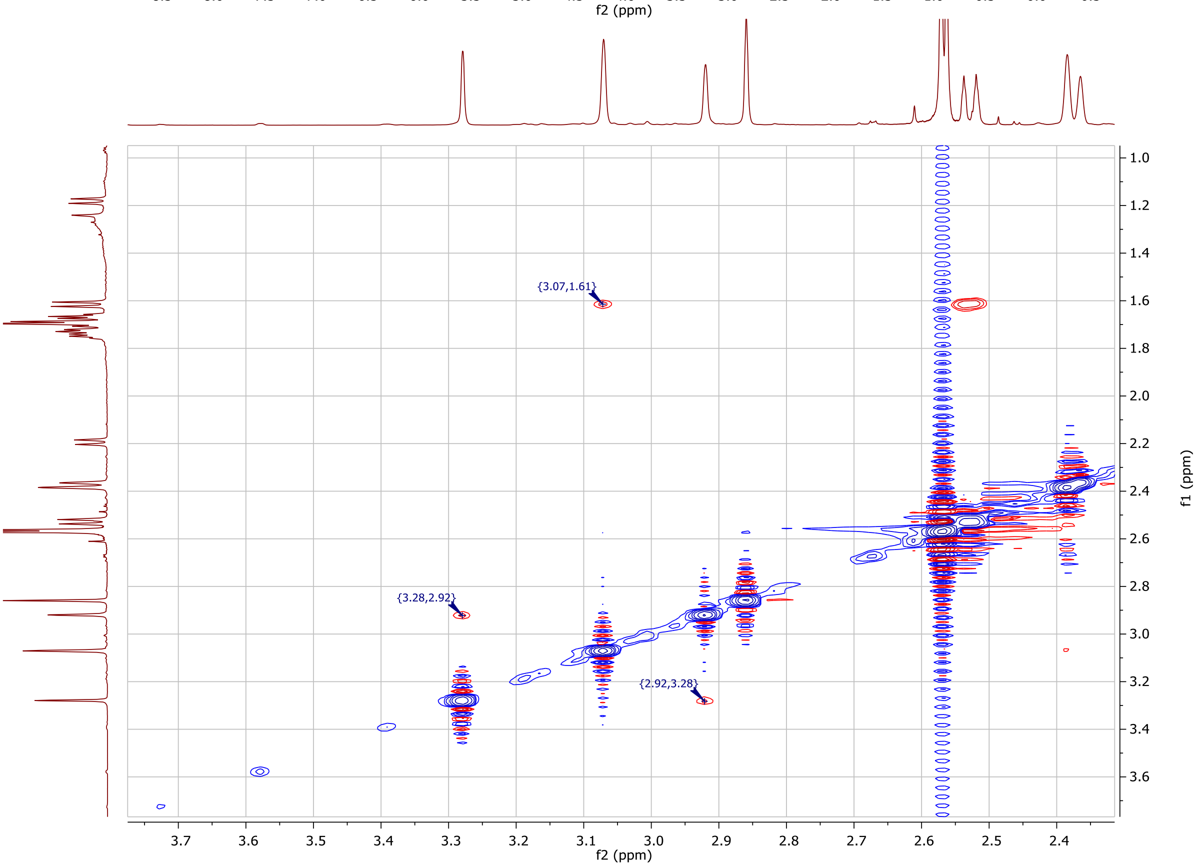

{3.07,1.61}
{3.28,2.92}
{2.92,3.28}
f2 (ppm)
f1 (ppm)

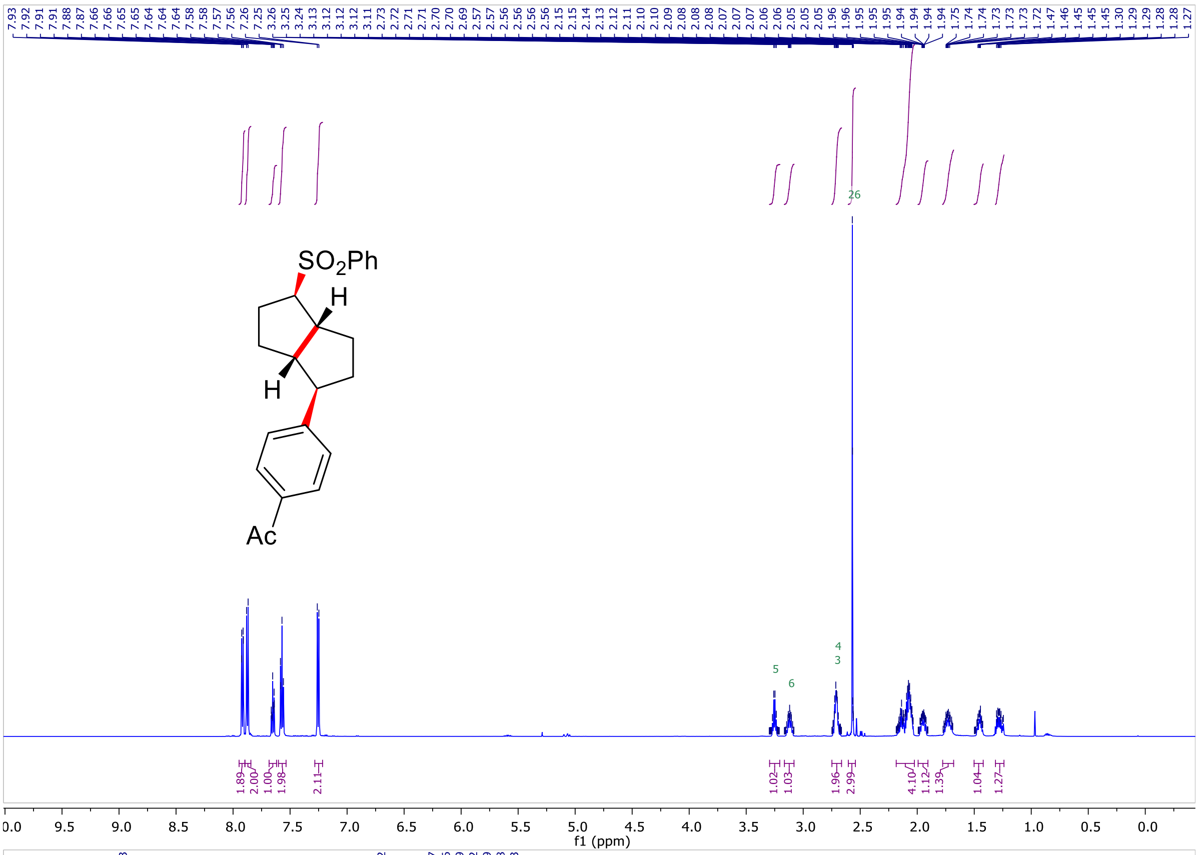
7.93 7.92 7.91 7.91 7.88 7.87 7.66 7.65 7.65 7.64 7.64 7.64 7.58 7.58 7.57 7.56 7.26 7.25 3.26 3.25 3.24 3.13 3.12 3.12 3.12 3.11 2.73 2.72 2.71 2.71 2.70 2.70 2.69 2.57 2.57 2.56 2.56 2.56 2.15 2.15 2.14 2.13 2.12 2.11 2.10 2.10 2.09 2.08 2.08 2.08 2.07 2.07 2.07 2.06 2.06 2.05 2.05 2.05 1.96 1.96 1.95 1.95 1.95 1.94 1.94 1.94 1.94 1.75 1.74 1.74 1.73 1.73 1.72 1.47 1.46 1.45 1.45 1.30 1.29 1.29 1.28 1.28 1.27
SO2Ph
H
H
Ac
26
5
6
4
3
1.89
2.00
1.00
1.98
2.11
1.02
1.03
1.96
2.99
4.10
1.12
1.39
1.04
1.27
f1 (ppm)

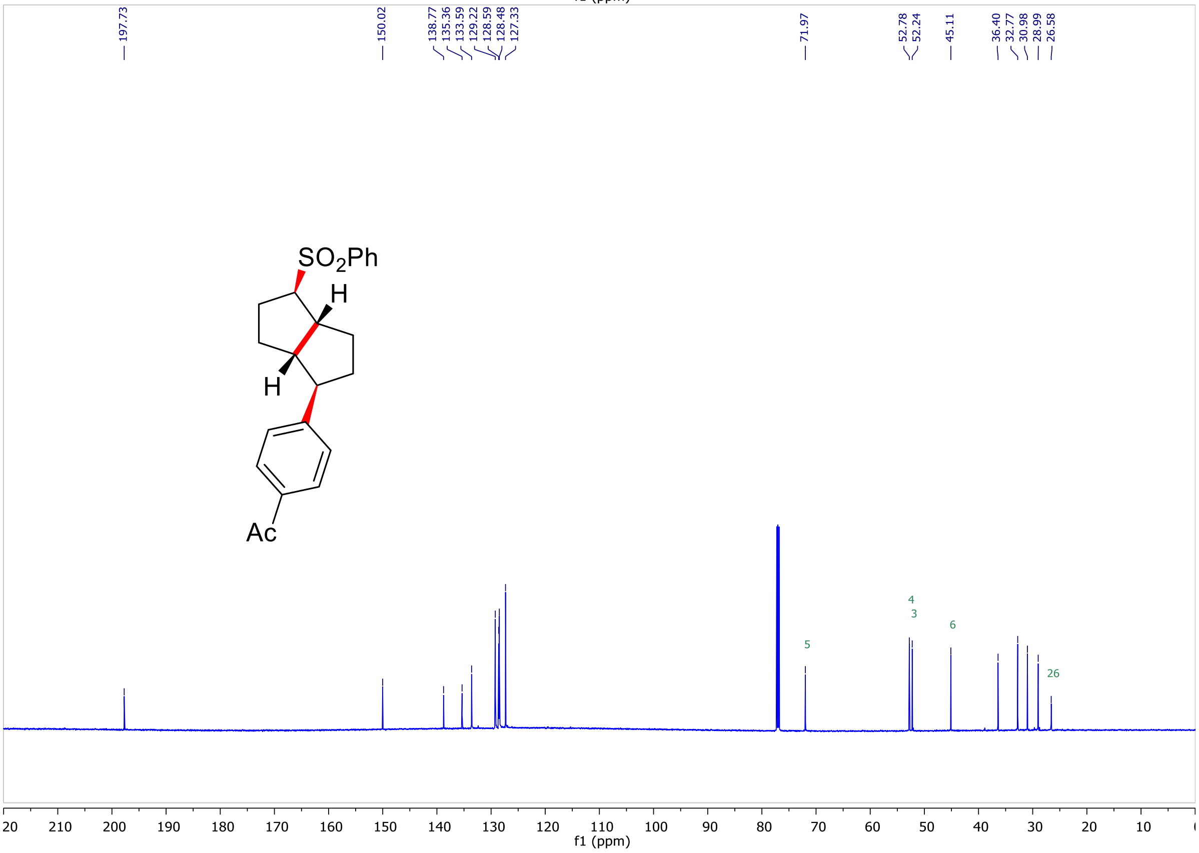
197.73
150.02
138.77 135.36 133.59 129.22 128.59 128.48 127.33
71.97
52.78 52.24
45.11
36.40 32.77 30.98 28.99 26.58
SO2Ph
H
H
Ac
4
3
5
6
26
f1 (ppm)

NOESY

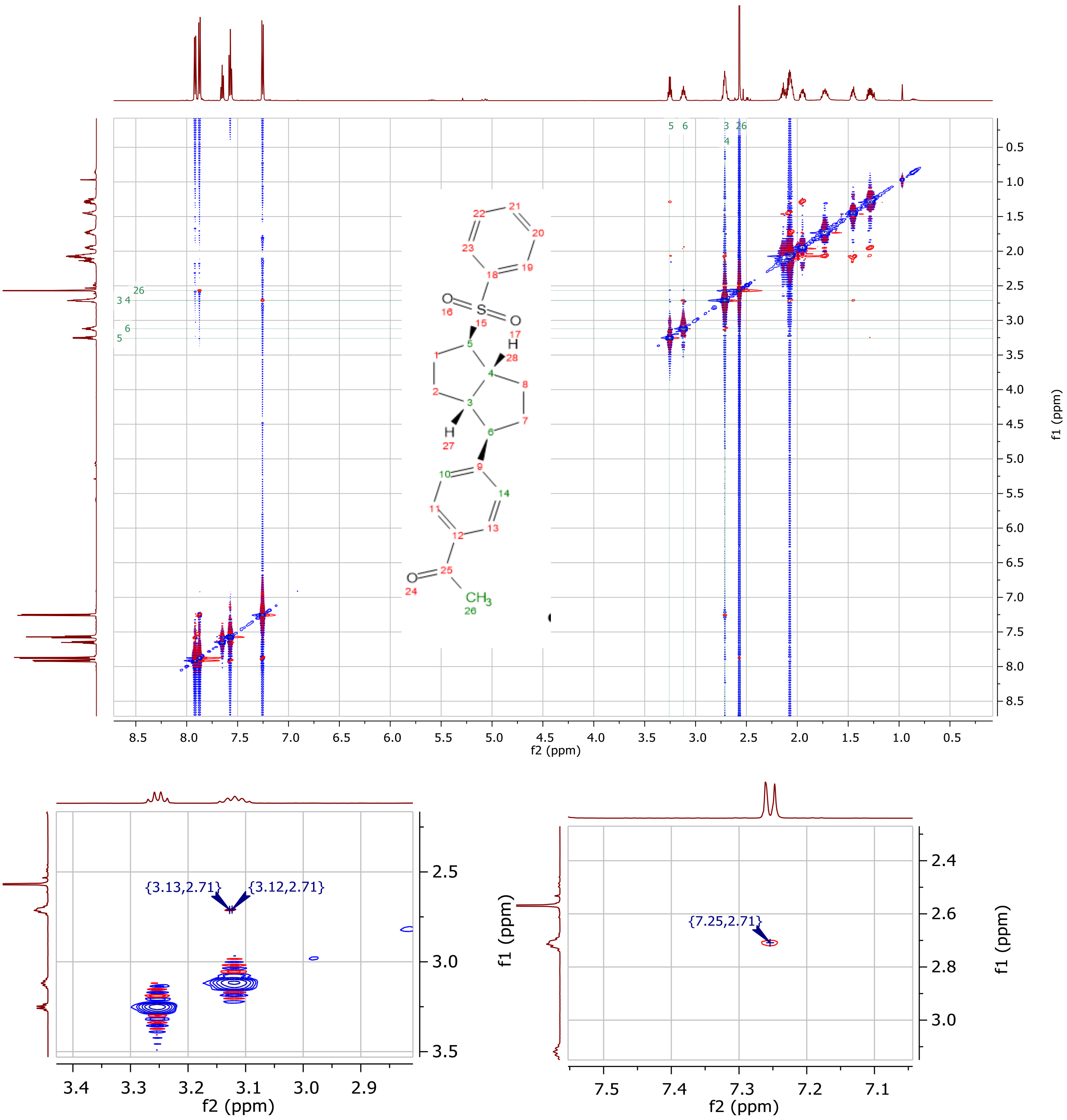

HSQC

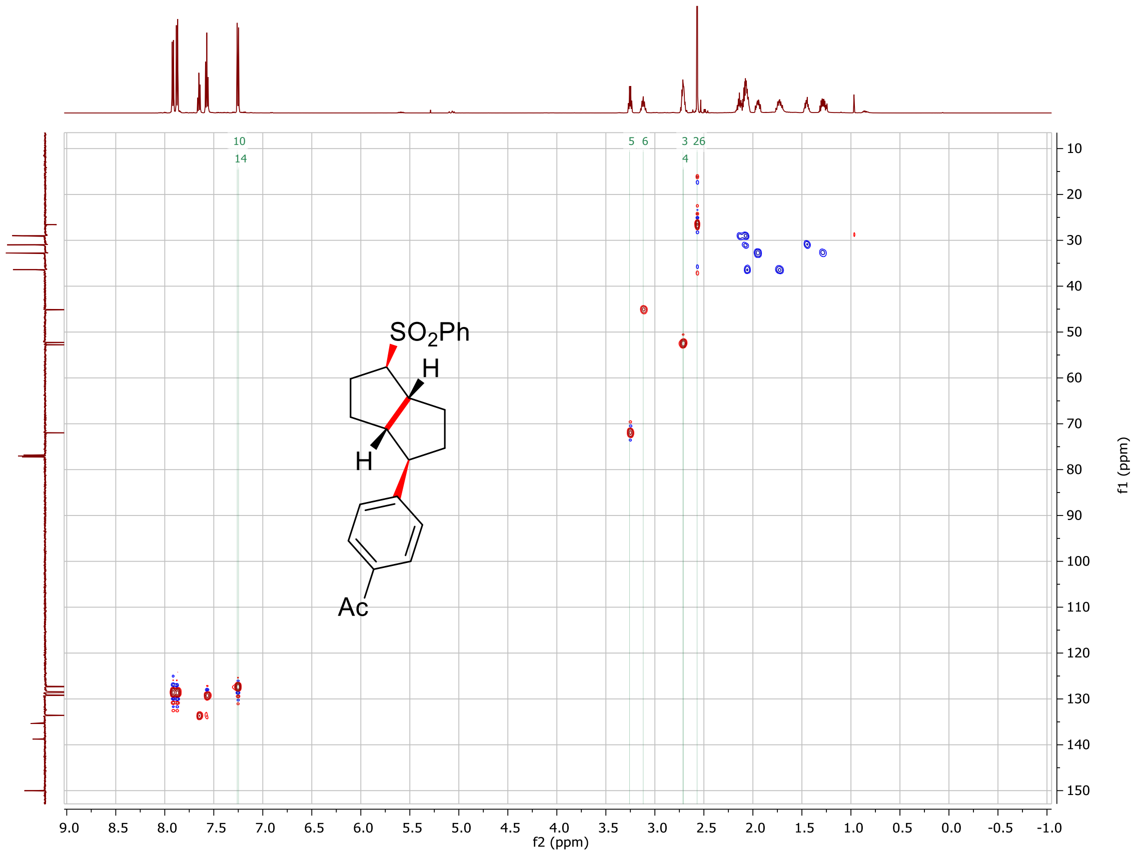

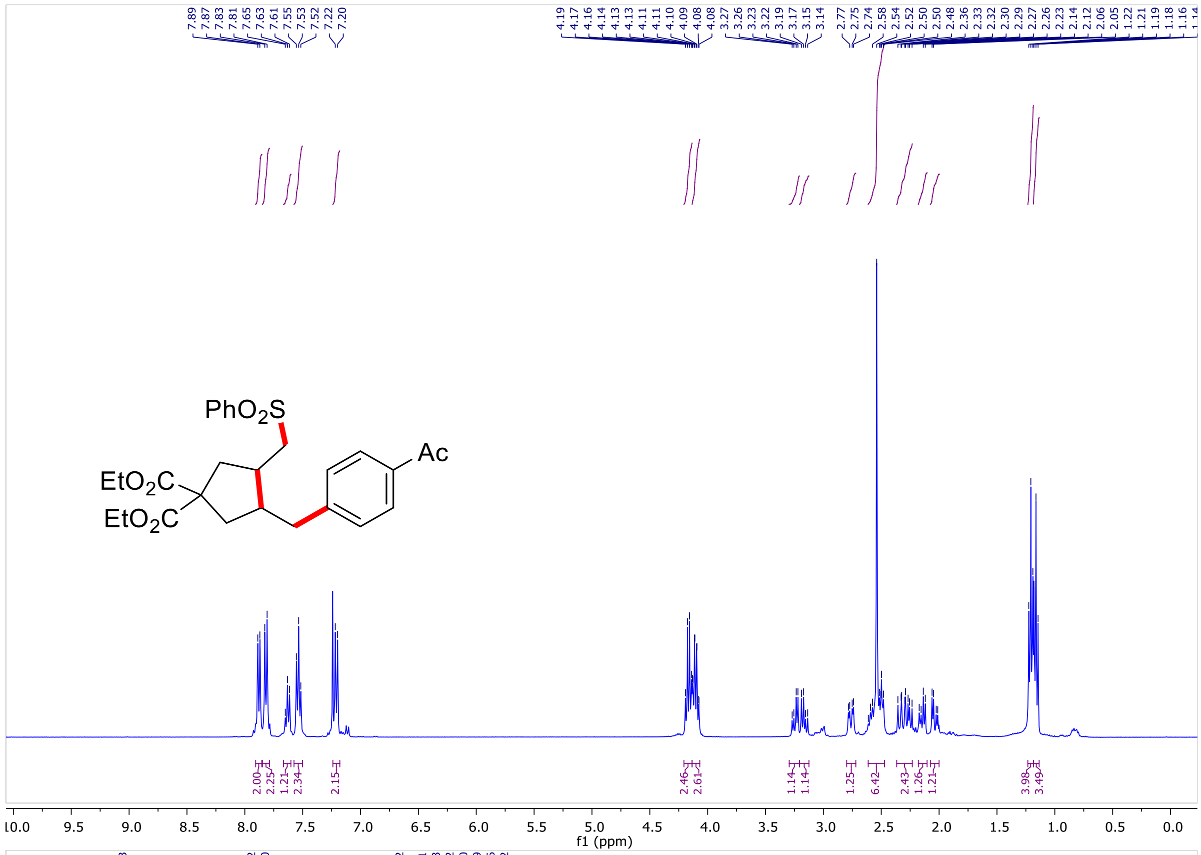
PhO2S
EtO2C
EtO2C
Ac
f1 (ppm)

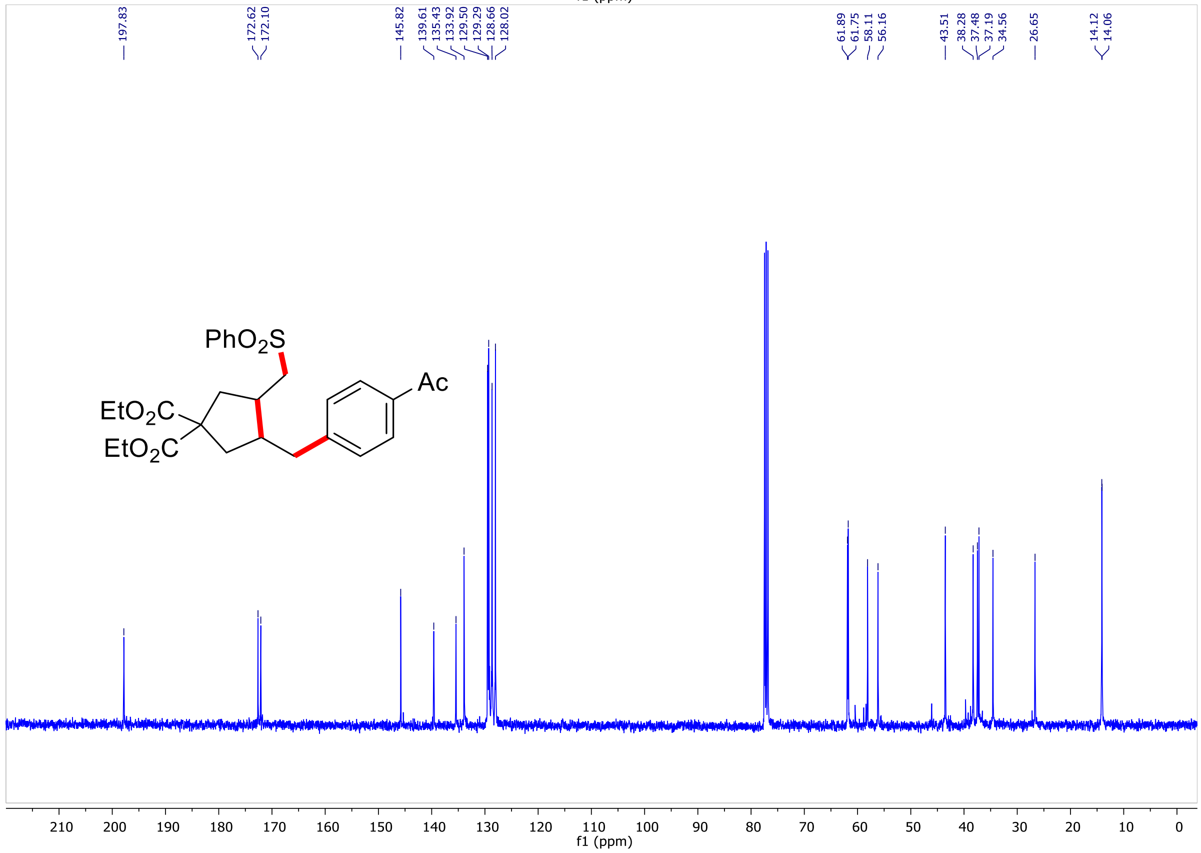
197.83
172.62
172.10
145.82
139.61
135.43
133.92
129.50
129.29
128.66
128.02
61.89
61.75
58.11
56.16
43.51
38.28
37.48
37.19
34.56
26.65
14.12
14.06
PhO2S
EtO2C
EtO2C
Ac
f1 (ppm)

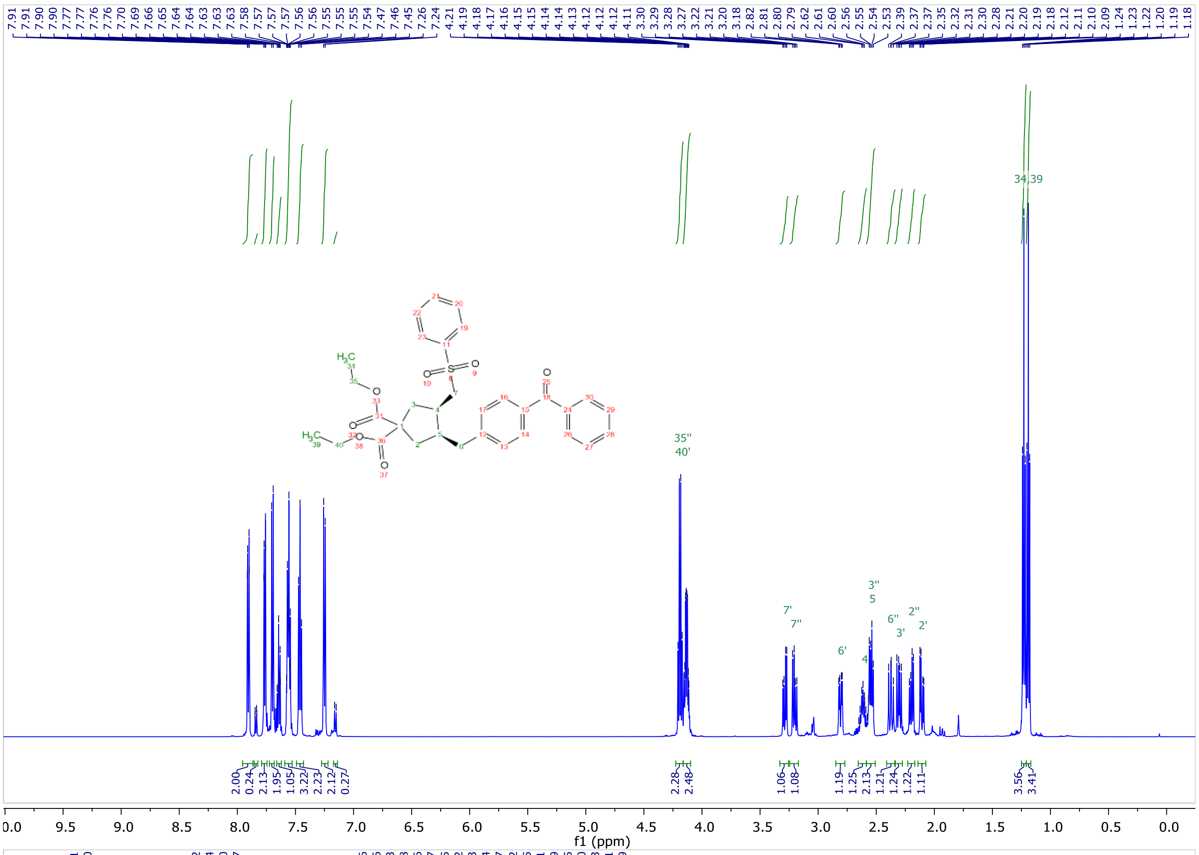

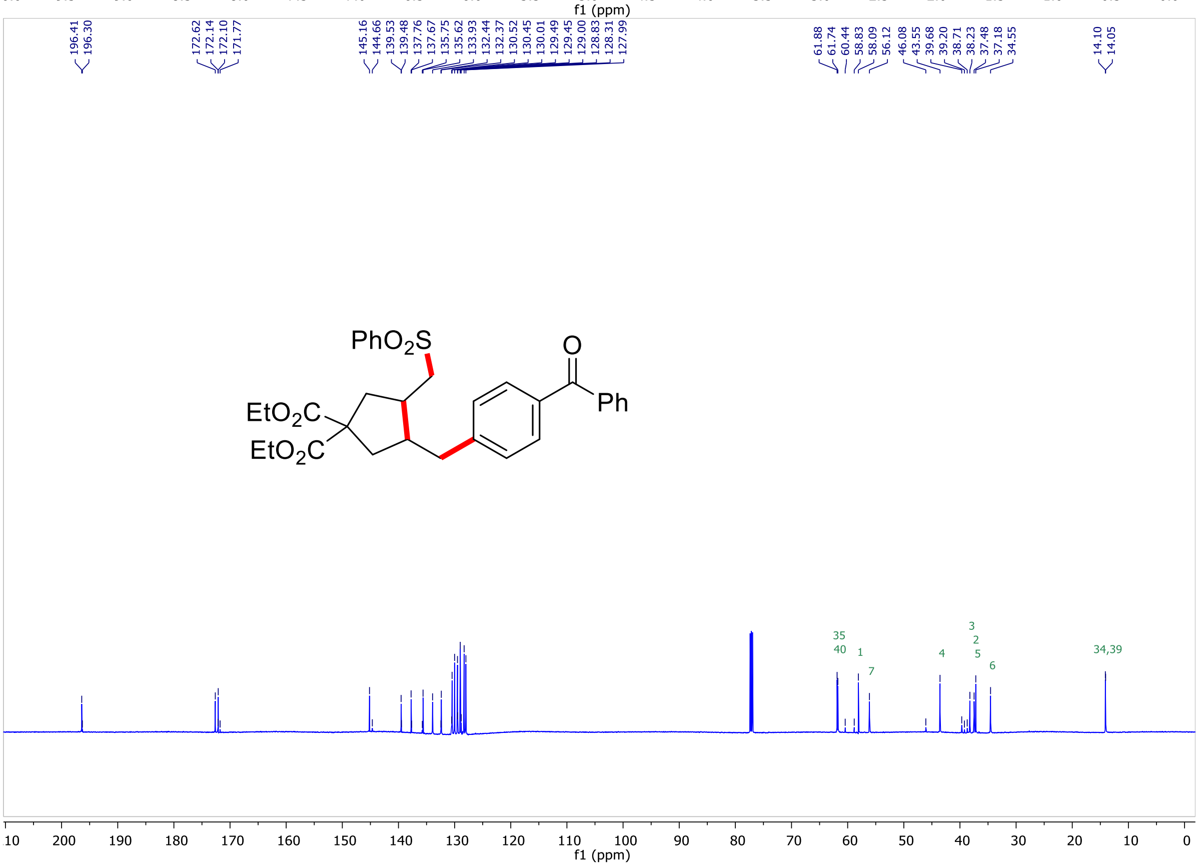

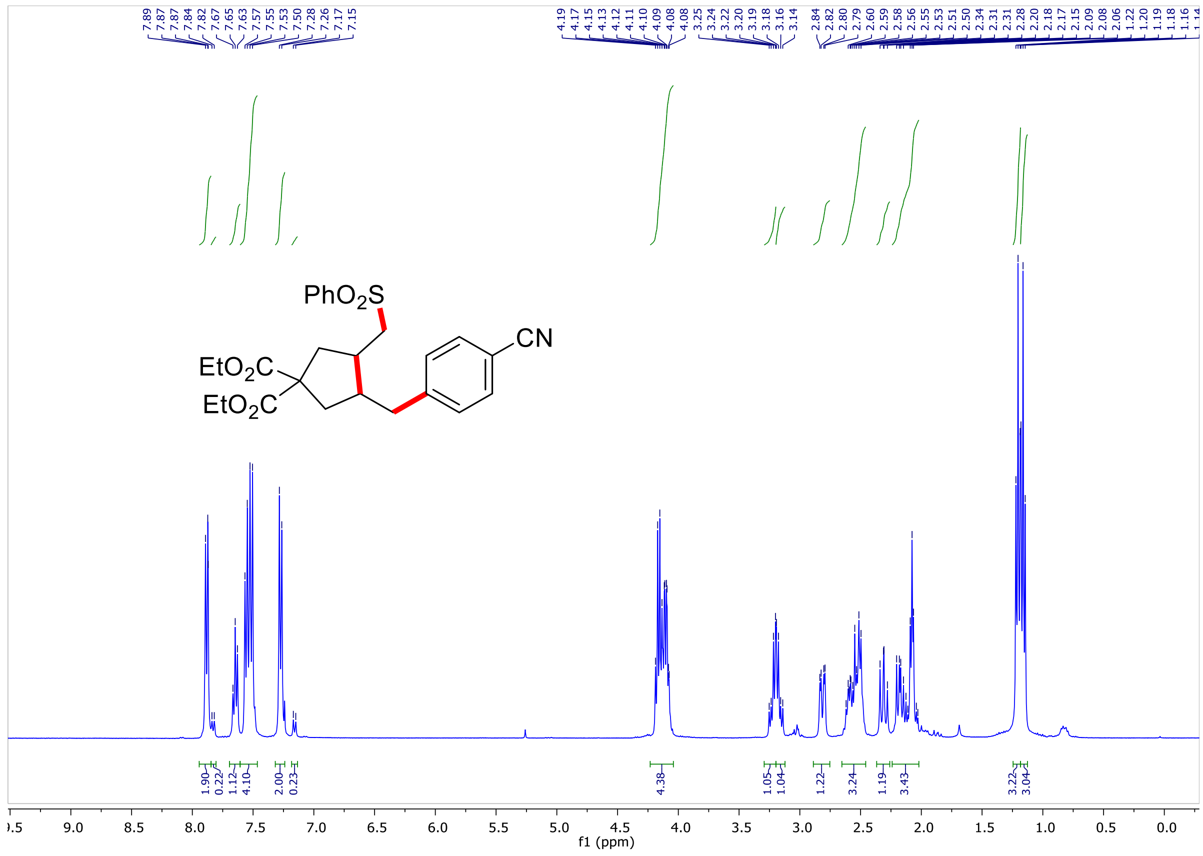
PhO2S
EtO2C
EtO2C
CN
f1 (ppm)

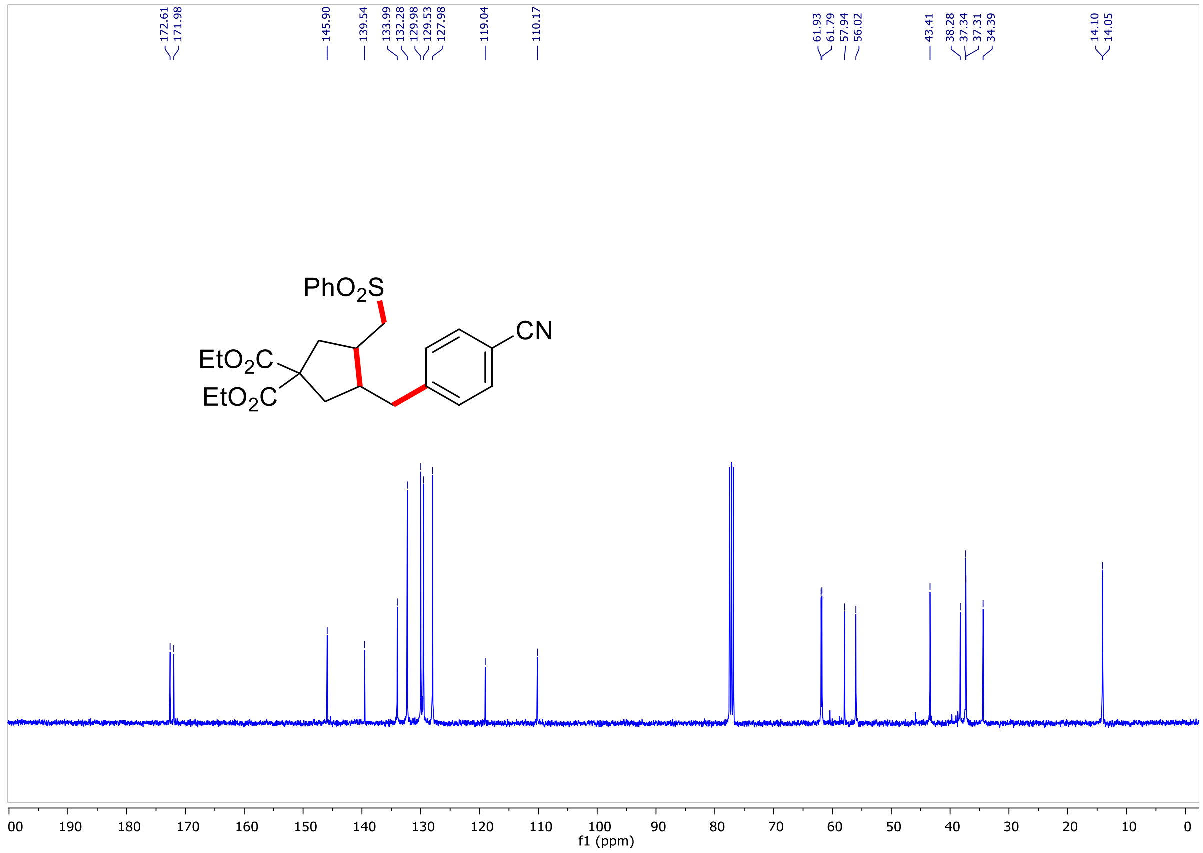
PhO2S
EtO2C
EtO2C
CN
f1 (ppm)

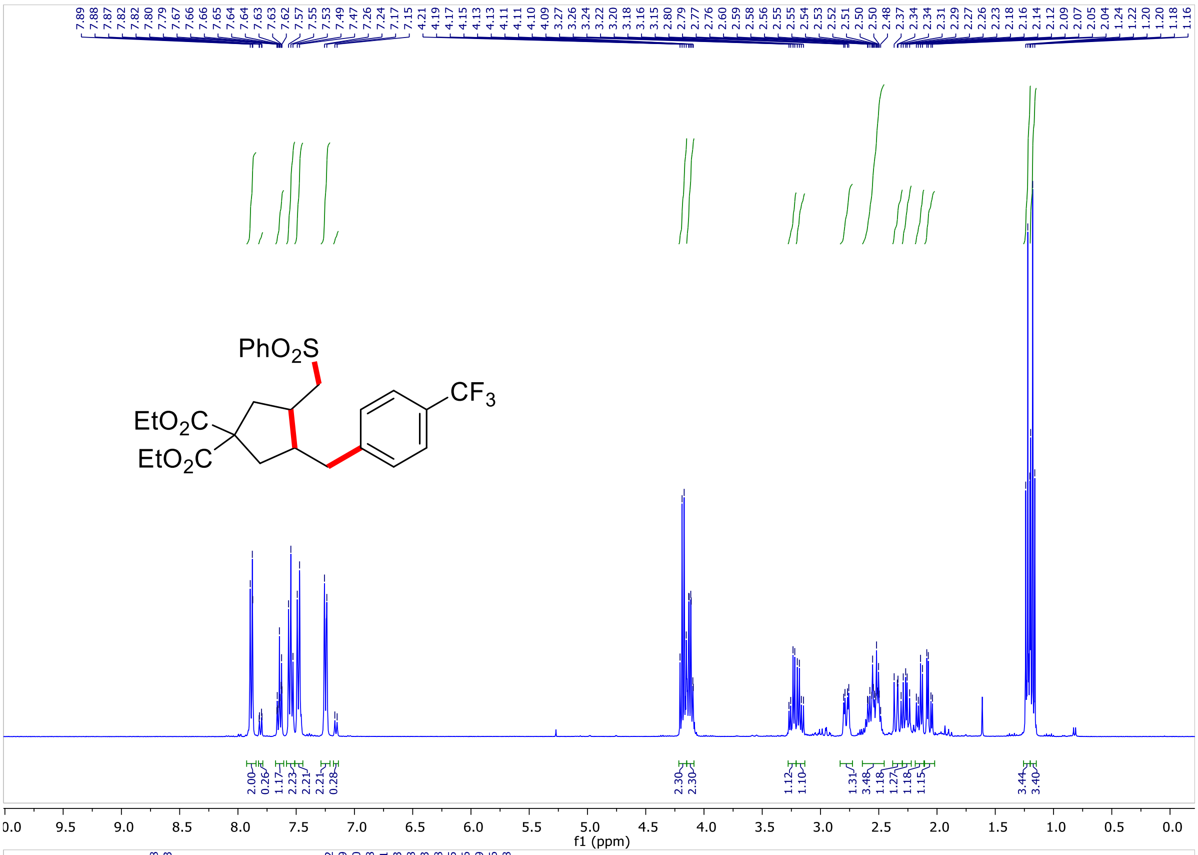
PhO2S
EtO2C
EtO2C
CF3
f1 (ppm)

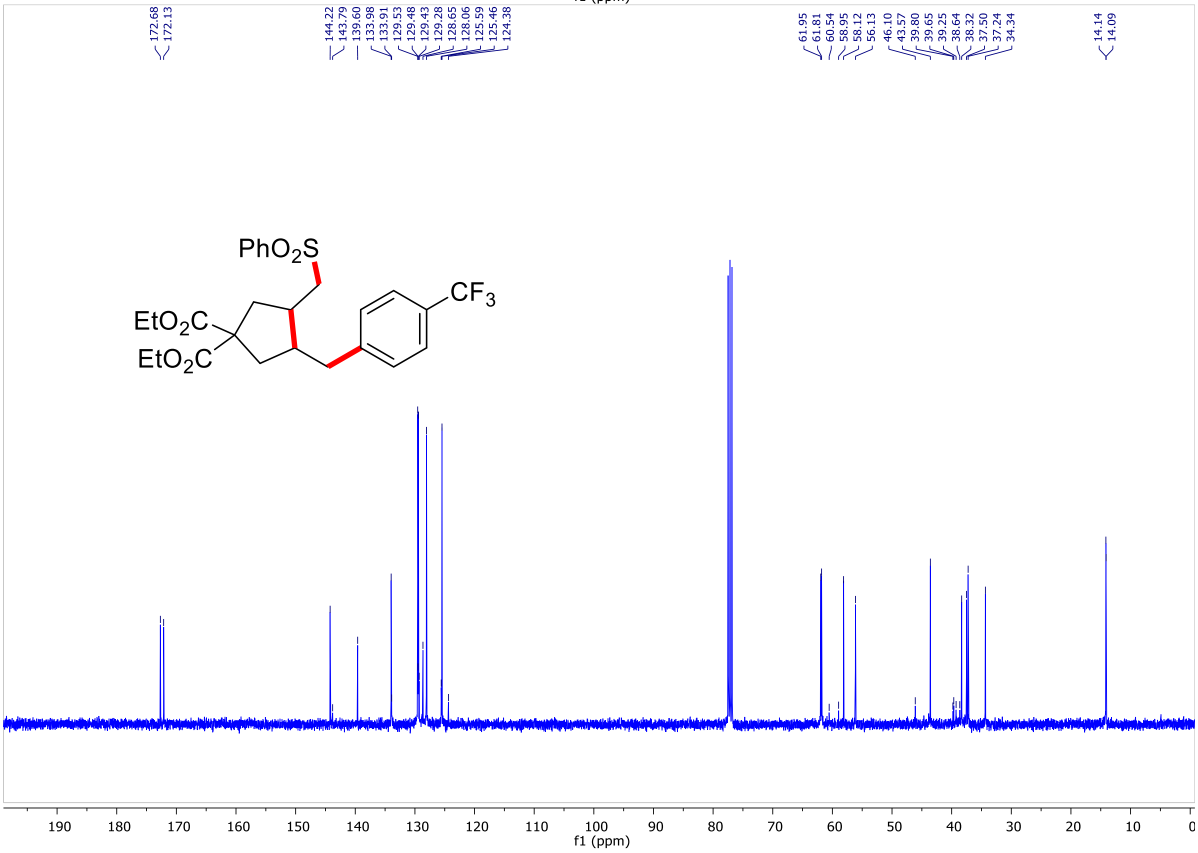
PhO2S
EtO2C
EtO2C
CF3
f1 (ppm)

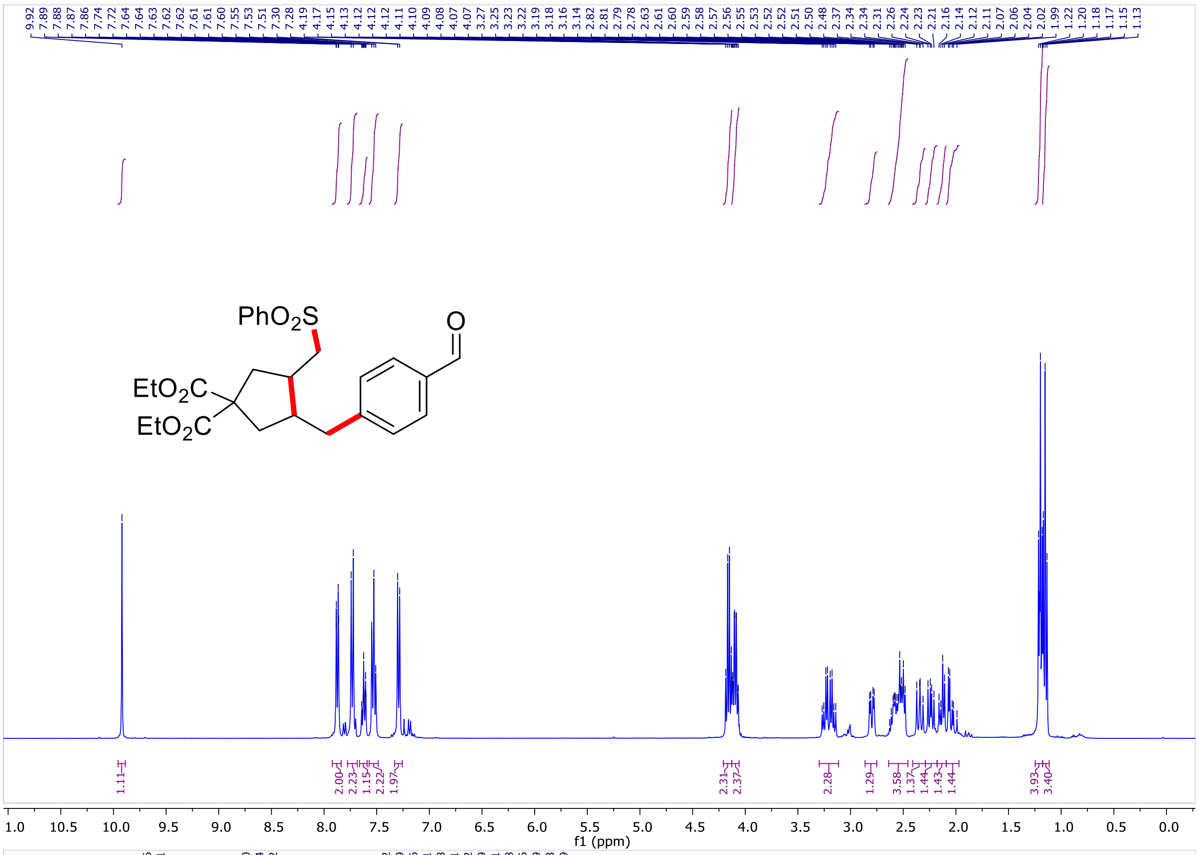
9.92 7.89 7.88 7.87 7.86 7.74 7.72 7.64 7.64 7.63 7.62 7.62 7.61 7.61 7.60 7.55 7.53 7.51 7.30 7.28 4.19 4.17 4.15 4.13 4.12 4.12 4.12 4.12 4.11 4.10 4.09 4.08 4.07 4.07 3.27 3.25 3.23 3.22 3.19 3.18 3.16 3.14 2.82 2.81 2.79 2.78 2.63 2.61 2.60 2.59 2.58 2.57 2.56 2.55 2.53 2.52 2.52 2.51 2.50 2.48 2.37 2.34 2.34 2.31 2.26 2.24 2.23 2.21 2.16 2.14 2.12 2.11 2.07 2.06 2.04 2.02 1.99 1.22 1.20 1.18 1.17 1.15 1.13
PhO2S
O
EtO2C
EtO2C
1.11 2.00 2.23 1.15 2.22 1.97 2.31 2.37 2.28 1.29 3.58 1.37 1.44 1.43 1.44 3.93 3.40
f1 (ppm)

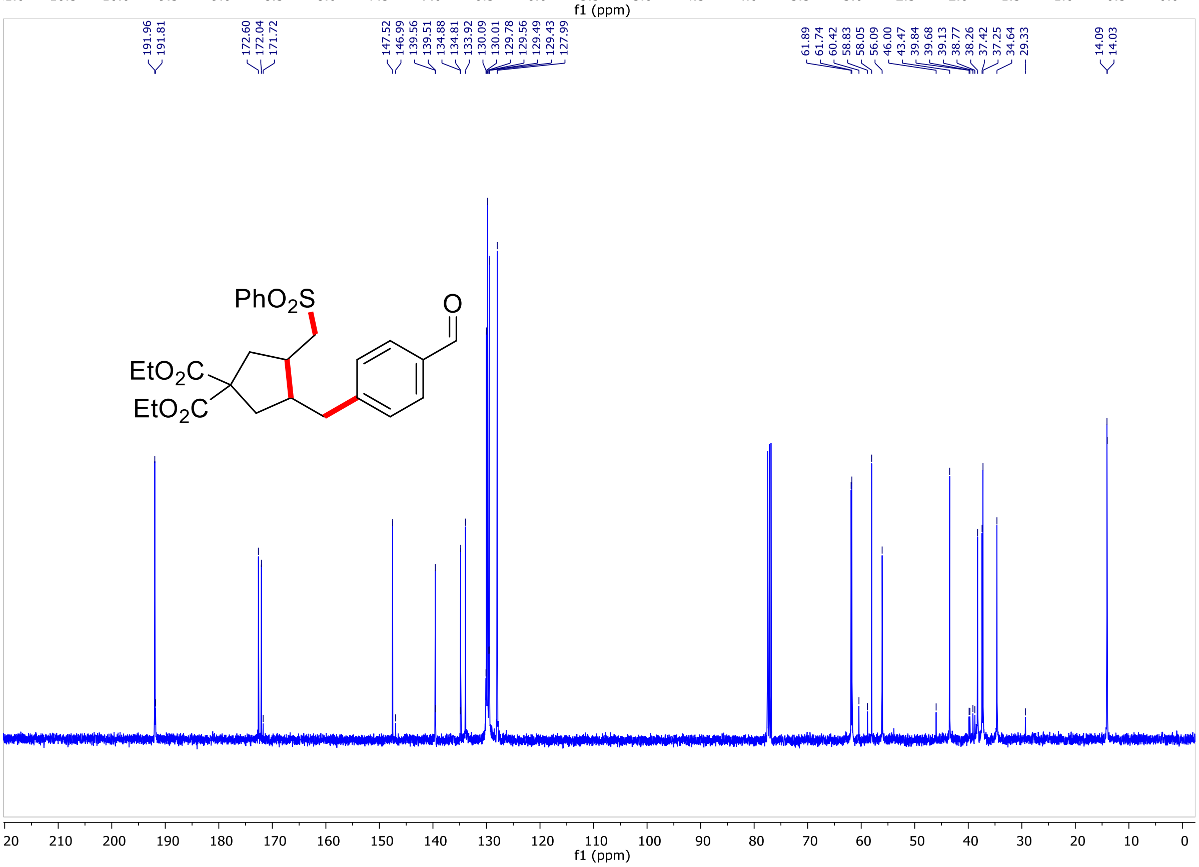
191.96 191.81 172.60 172.04 171.72 147.52 146.99 139.56 139.51 134.88 134.81 133.92 130.09 130.01 129.78 129.56 129.49 129.43 127.99 61.89 61.74 60.42 58.83 58.05 56.09 46.00 43.47 39.84 39.68 39.13 38.77 38.26 37.42 37.25 34.64 29.33 14.09 14.03
PhO2S
O
EtO2C
EtO2C
f1 (ppm)

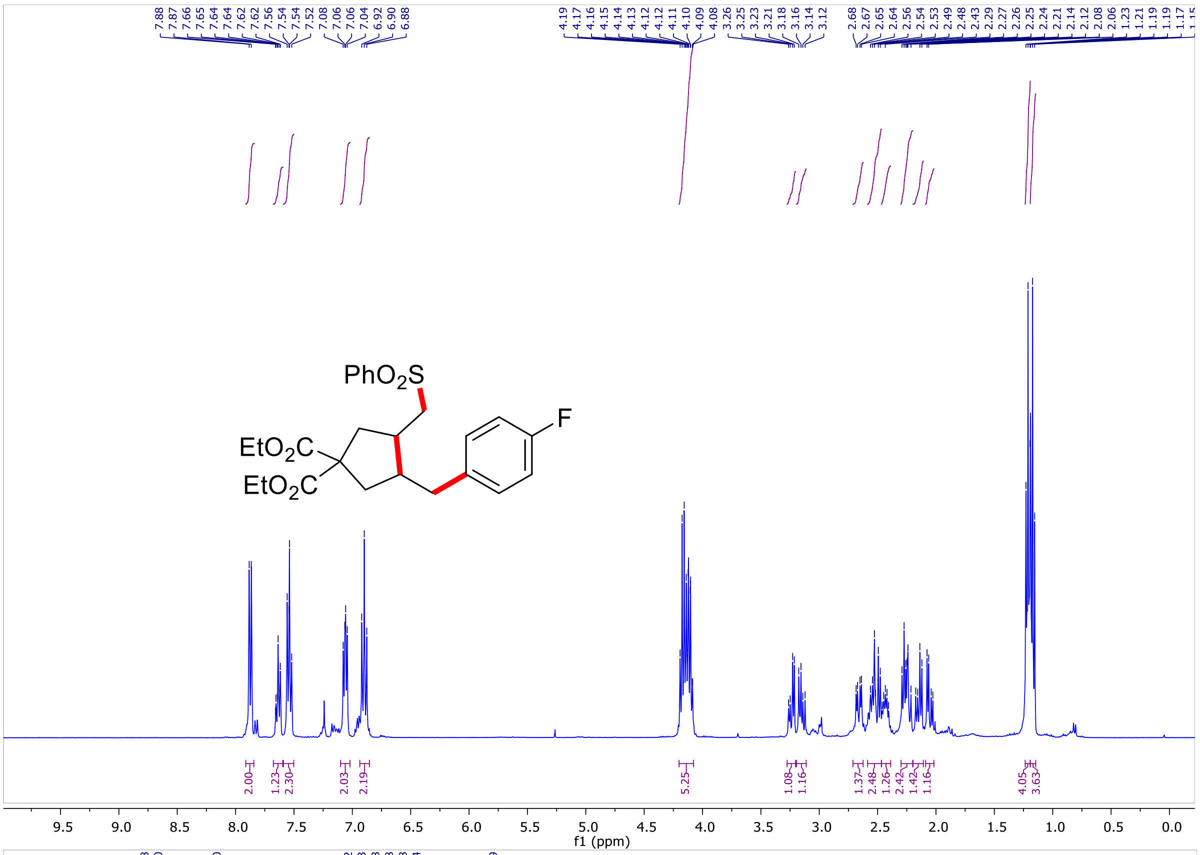
7.88 7.87 7.66 7.65 7.64 7.64 7.62 7.62 7.56 7.54 7.54 7.52 7.08 7.06 7.06 7.04 6.92 6.90 6.88
4.19 4.17 4.16 4.15 4.14 4.13 4.12 4.12 4.11 4.10 4.09 4.08 3.26 3.25 3.23 3.21 3.18 3.16 3.14 3.12
2.68 2.67 2.65 2.64 2.56 2.54 2.53 2.49 2.48 2.43 2.29 2.27 2.26 2.25 2.24 2.21 2.14 2.12 2.08 2.06 1.23 1.21 1.19 1.17
PhO2S
F
EtO2C
EtO2C
2.00 1.23 2.30 2.03 2.19 5.25 1.08 1.16 1.37 2.48 1.26 2.42 1.42 1.16 4.05 3.63
f1 (ppm)

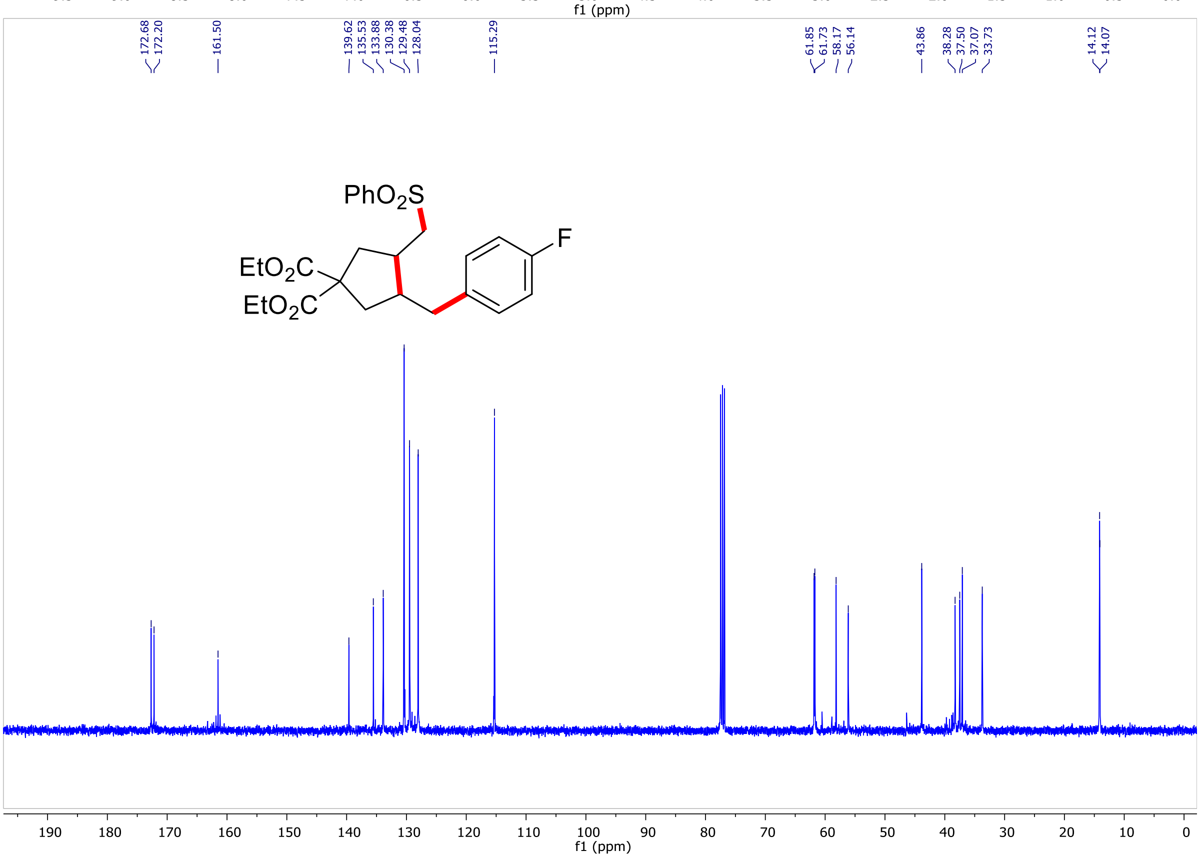
172.68 172.20 161.50 139.62 135.53 133.88 130.38 129.48 128.04 115.29 61.85 61.73 58.17 56.14 43.86 38.28 37.50 37.07 33.73 14.12 14.07
PhO2S
F
EtO2C
EtO2C
f1 (ppm)

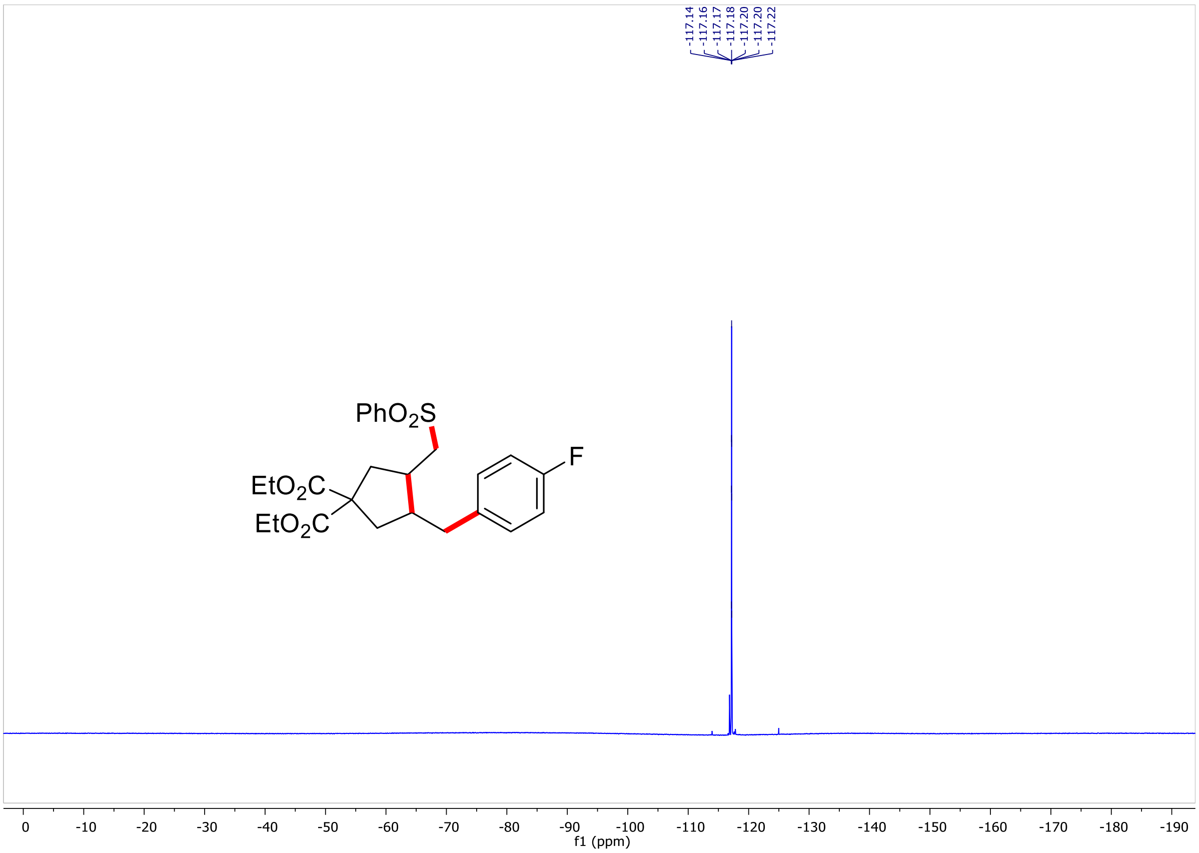

-117.14
-117.16
-117.17
-117.18
-117.20
-117.20
-117.22
PhO2S
EtO2C
EtO2C
F
0
-10
-20
-30
-40
-50
-60
-70
-80
-90
-100
-110
-120
-130
-140
-150
-160
-170
-180
-190
f1 (ppm)

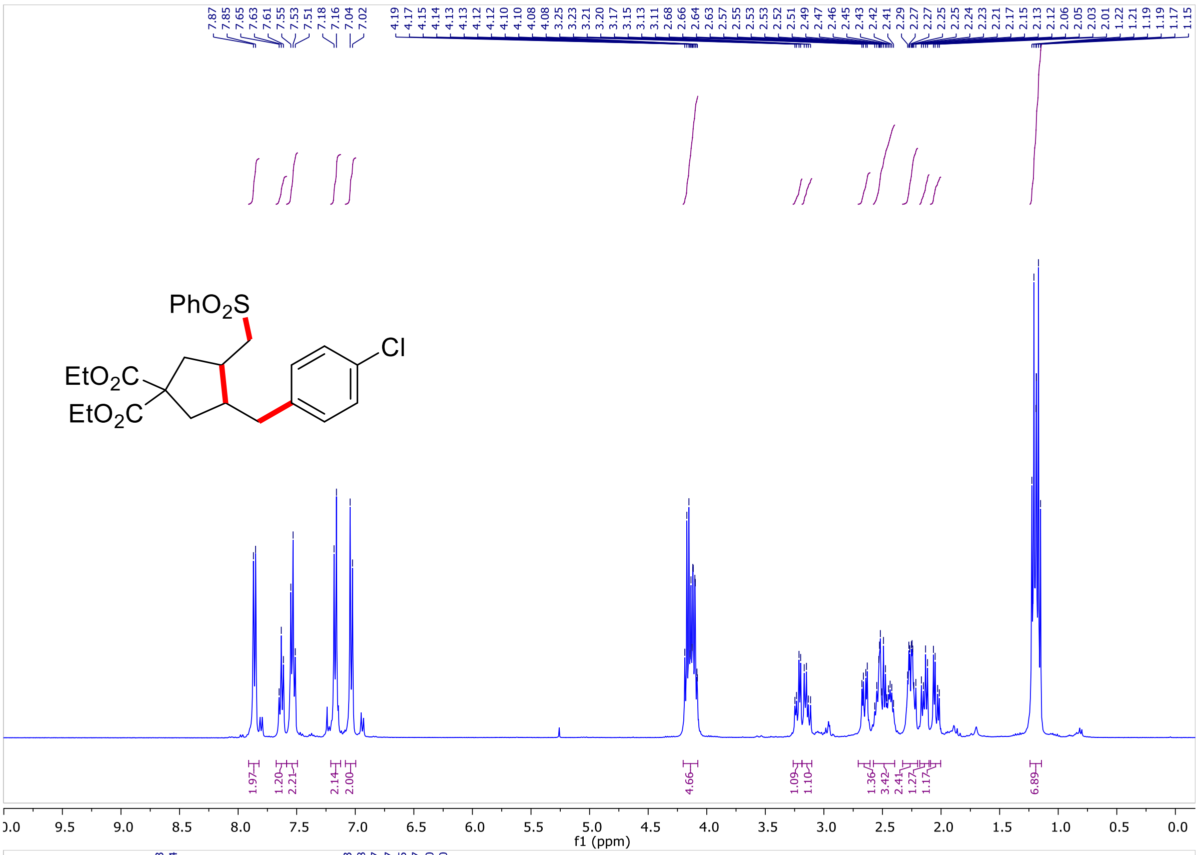
7.87 7.85 7.65 7.63 7.61 7.55 7.53 7.51 7.18 7.16 7.04 7.02
4.19 4.17 4.15 4.14 4.13 4.13 4.12 4.12 4.10 4.10 4.08 4.08 3.25 3.23 3.21 3.20 3.17 3.15 3.13 3.11 2.68 2.66 2.64 2.63 2.57 2.55 2.53 2.53 2.52 2.51 2.49 2.47 2.46 2.45 2.43 2.42 2.41 2.29 2.27 2.27 2.25 2.25 2.24 2.23 2.21 2.17 2.15 2.13 2.12 2.06 2.05 2.03 2.01 1.22 1.21 1.19 1.19 1.17 1.15
PhO2S
Cl
EtO2C
EtO2C
1.97 1.20 2.21 2.14 2.00 4.66 1.09 1.10 1.36 3.42 2.41 1.27 1.17 6.89
f1 (ppm)

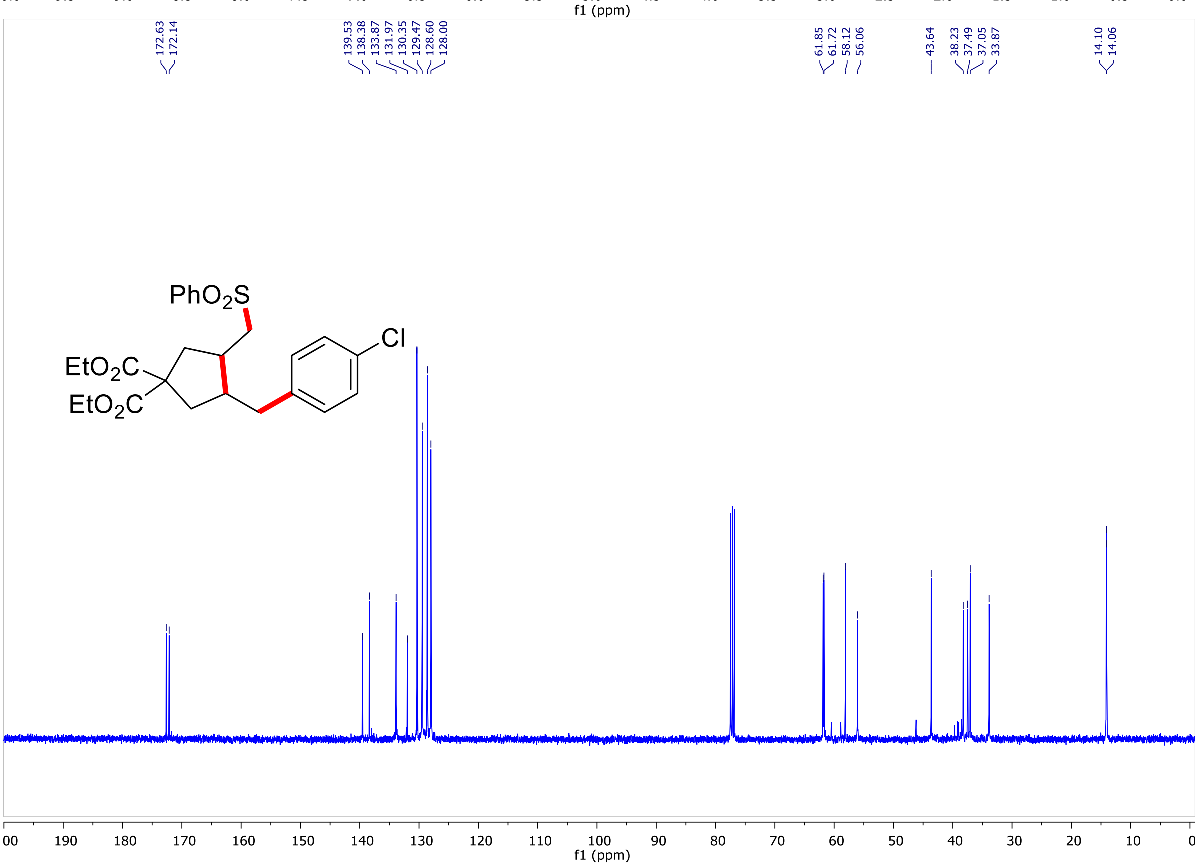
172.63 172.14
139.53 138.38 133.87 131.97 130.35 129.47 128.60 128.00
61.85 61.72 58.12 56.06
43.64
38.23 37.49 37.05 33.87
14.10 14.06
PhO2S
Cl
EtO2C
EtO2C
f1 (ppm)

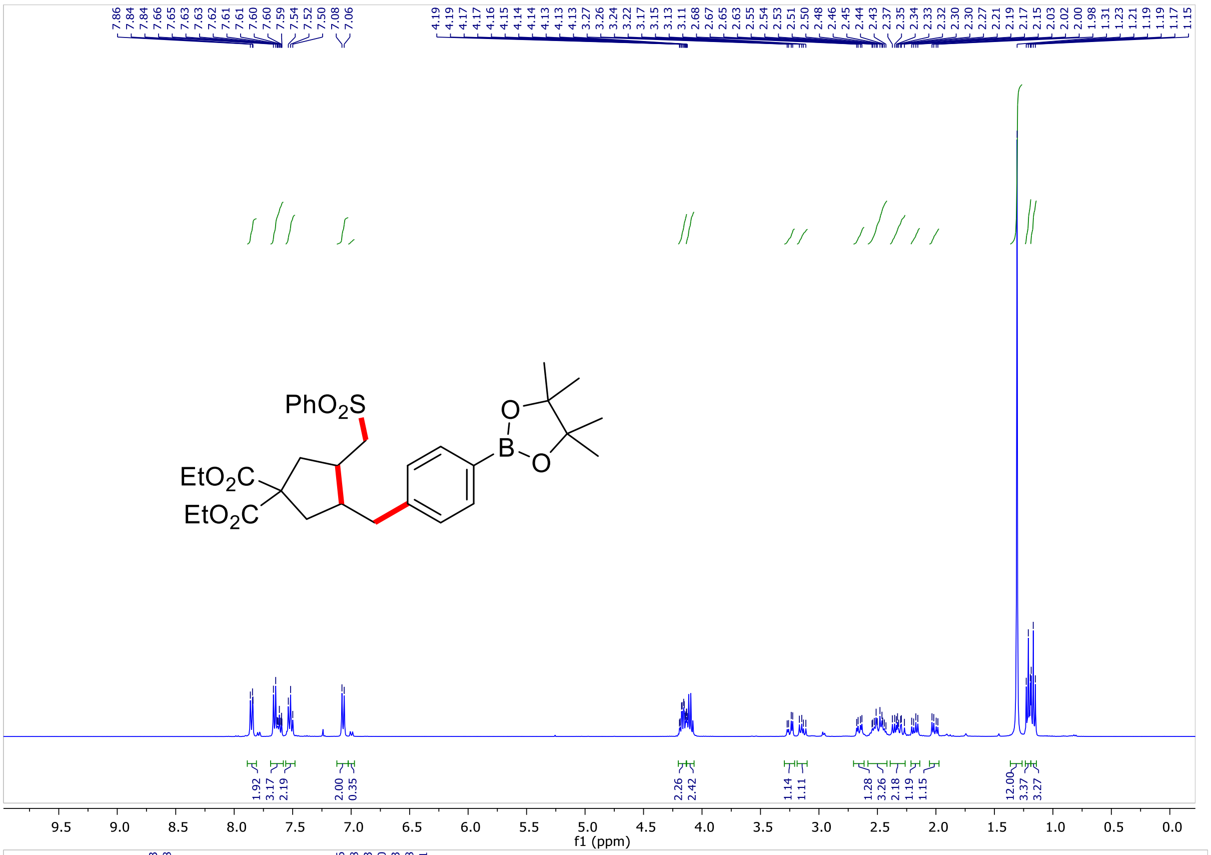
7.86 7.84 7.84 7.66 7.65 7.63 7.63 7.62 7.61 7.61 7.60 7.59 7.54 7.52 7.50 7.08 7.06
4.19 4.19 4.17 4.17 4.16 4.15 4.14 4.14 4.13 4.13 4.13 4.13 3.27 3.26 3.24 3.22 3.17 3.15 3.13 3.11 2.68 2.67 2.65 2.63 2.55 2.54 2.53 2.51 2.50 2.48 2.46 2.45 2.44 2.43 2.37 2.35 2.34 2.33 2.32 2.30 2.30 2.27 2.21 2.19 2.17 2.15 2.03 2.02 2.00 1.98 1.31 1.23 1.21 1.19 1.19 1.17 1.15
PhO2S
EtO2C
EtO2C
O
B
O
1.92 3.17 2.19 2.00 0.35 2.26 2.42 1.14 1.11 1.28 3.26 2.18 1.19 1.15 12.00 3.37 3.27
f1 (ppm)

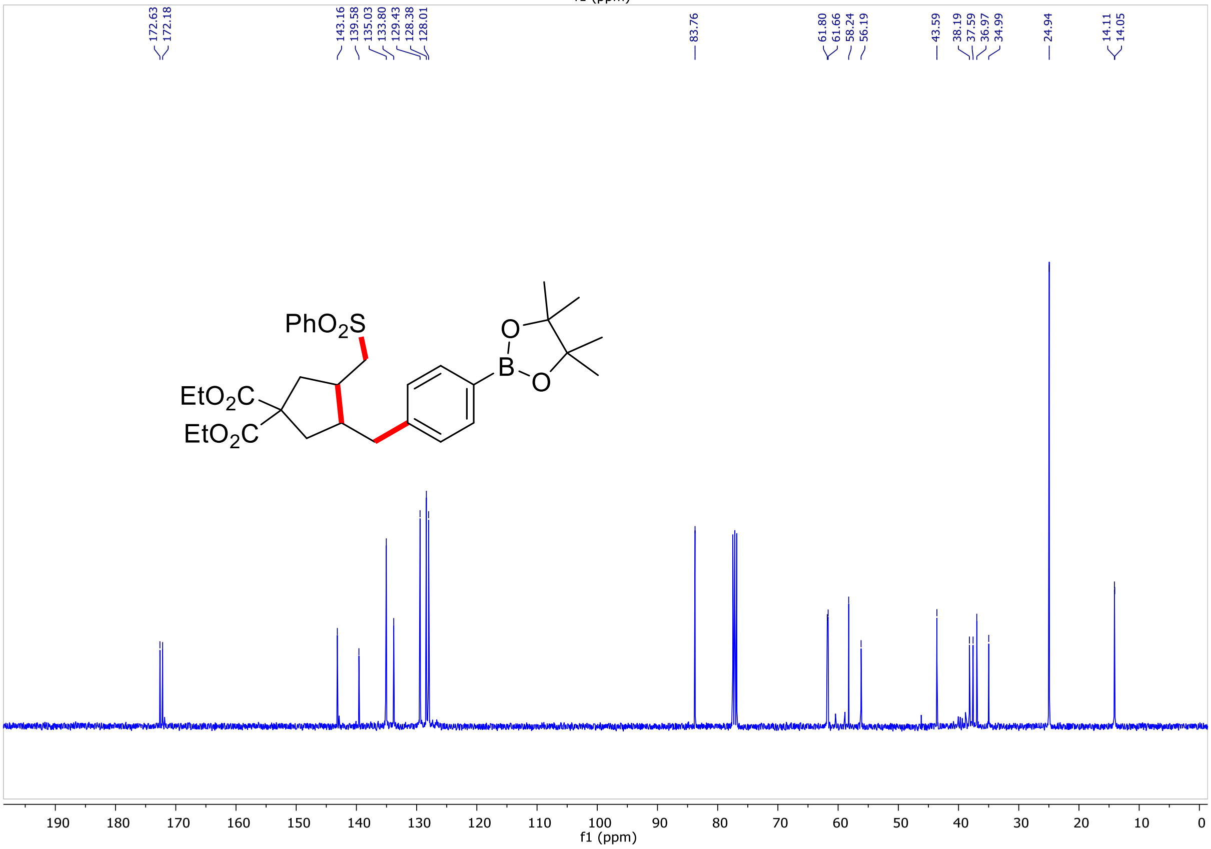
172.63 172.18 143.16 139.58 135.03 133.80 129.43 128.38 128.01 83.76 61.80 61.66 58.24 56.19 43.59 38.19 37.59 36.97 34.99 24.94 14.11 14.05
PhO2S
EtO2C
EtO2C
O
B
O
f1 (ppm)

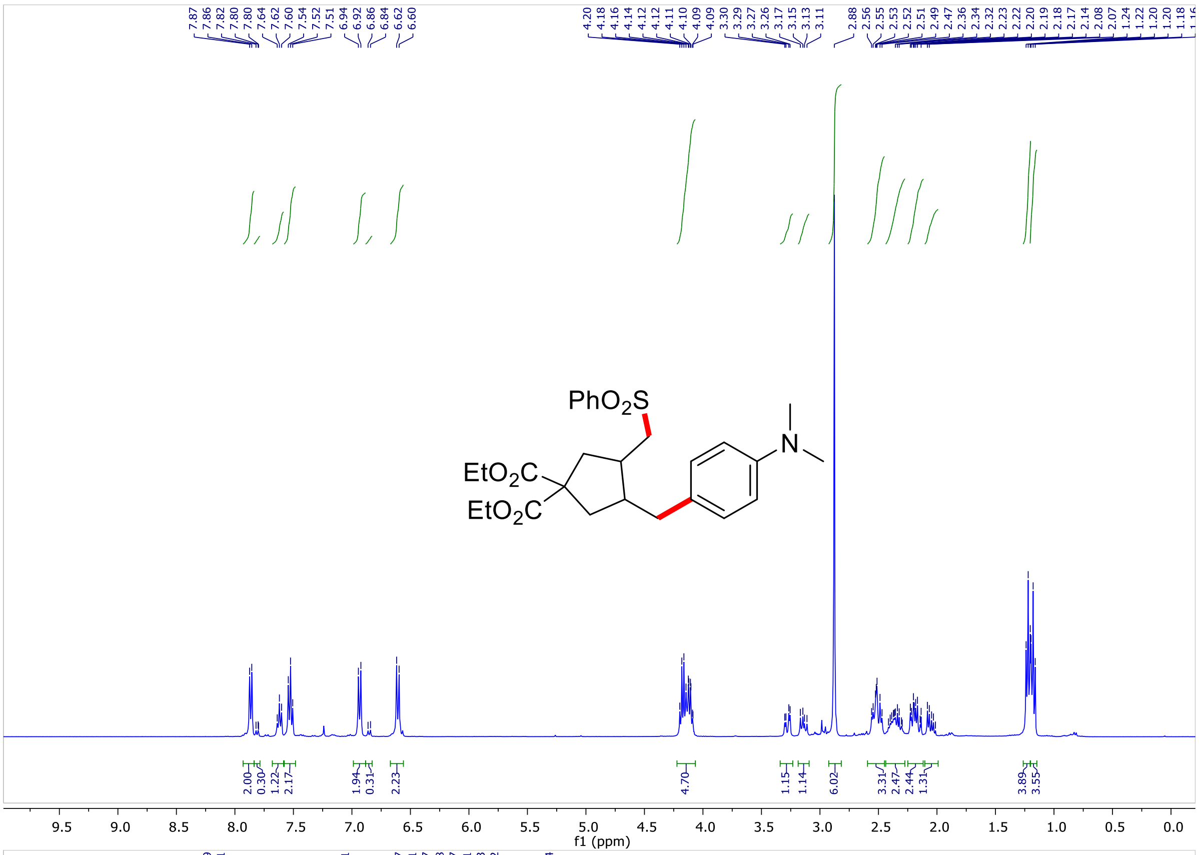
PhO2S
EtO2C
EtO2C
N
f1 (ppm)

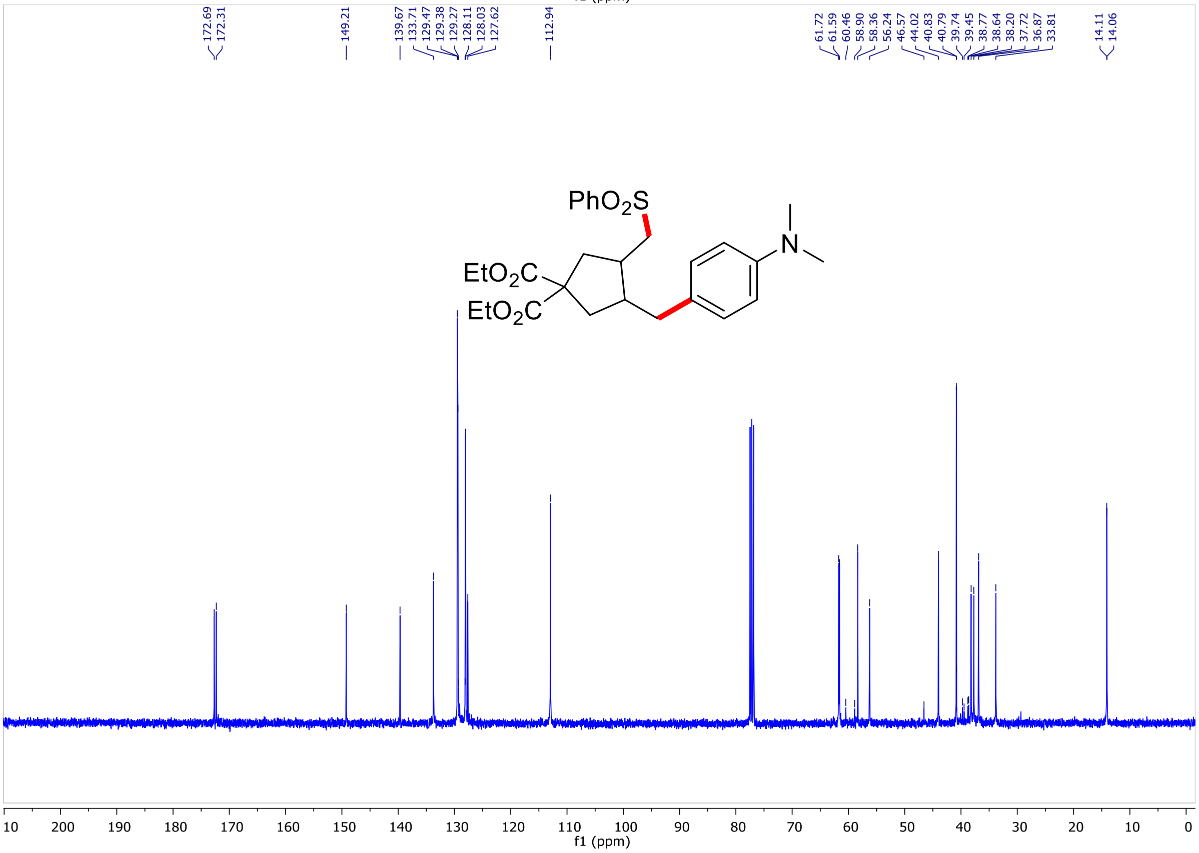
PhO2S
EtO2C
EtO2C
N
f1 (ppm)

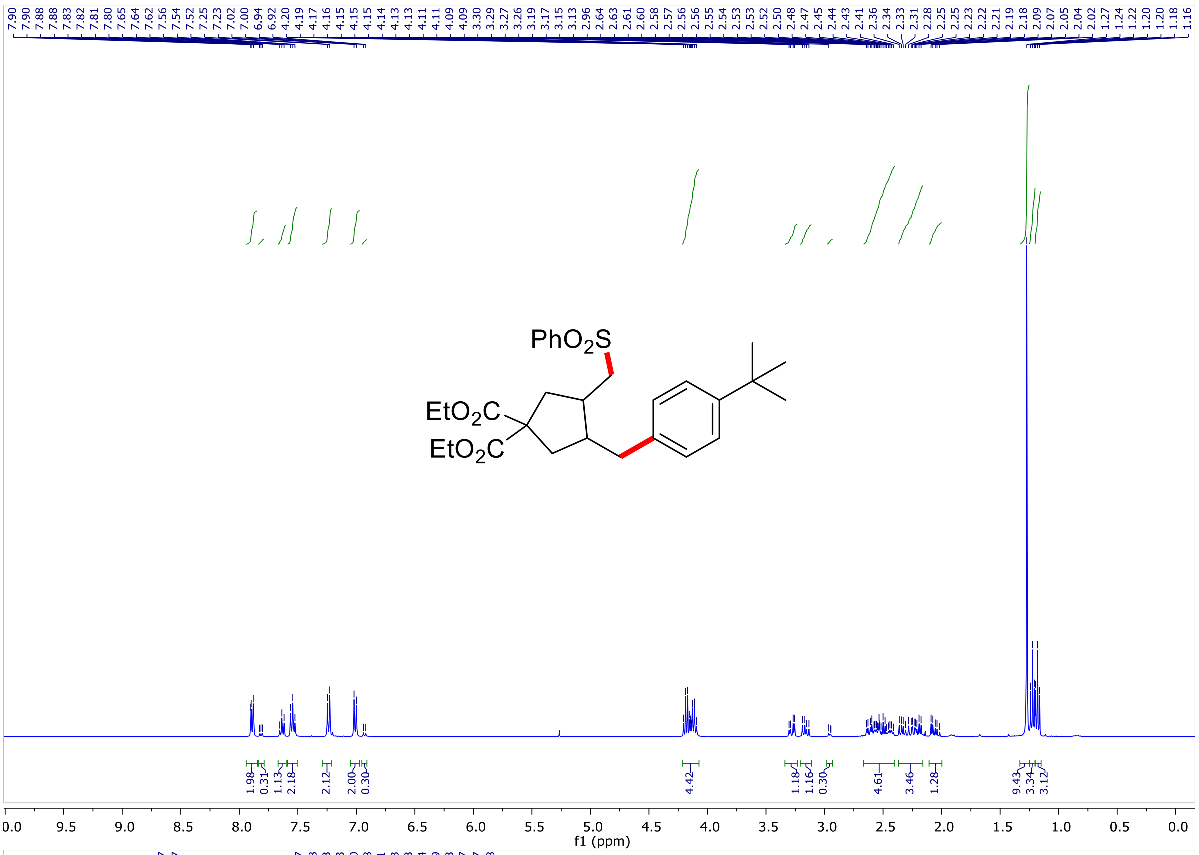
PhO2S
EtO2C
EtO2C
f1 (ppm)

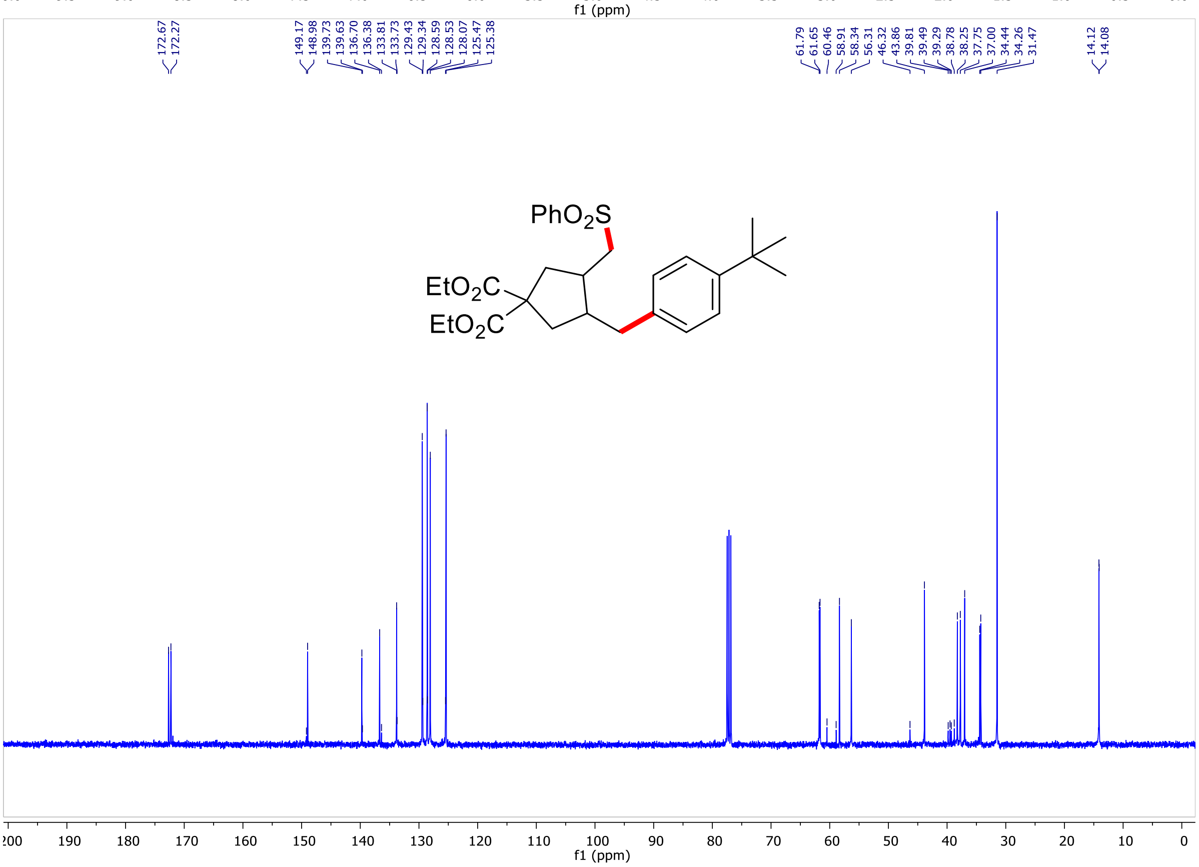
PhO2S
EtO2C
EtO2C
f1 (ppm)

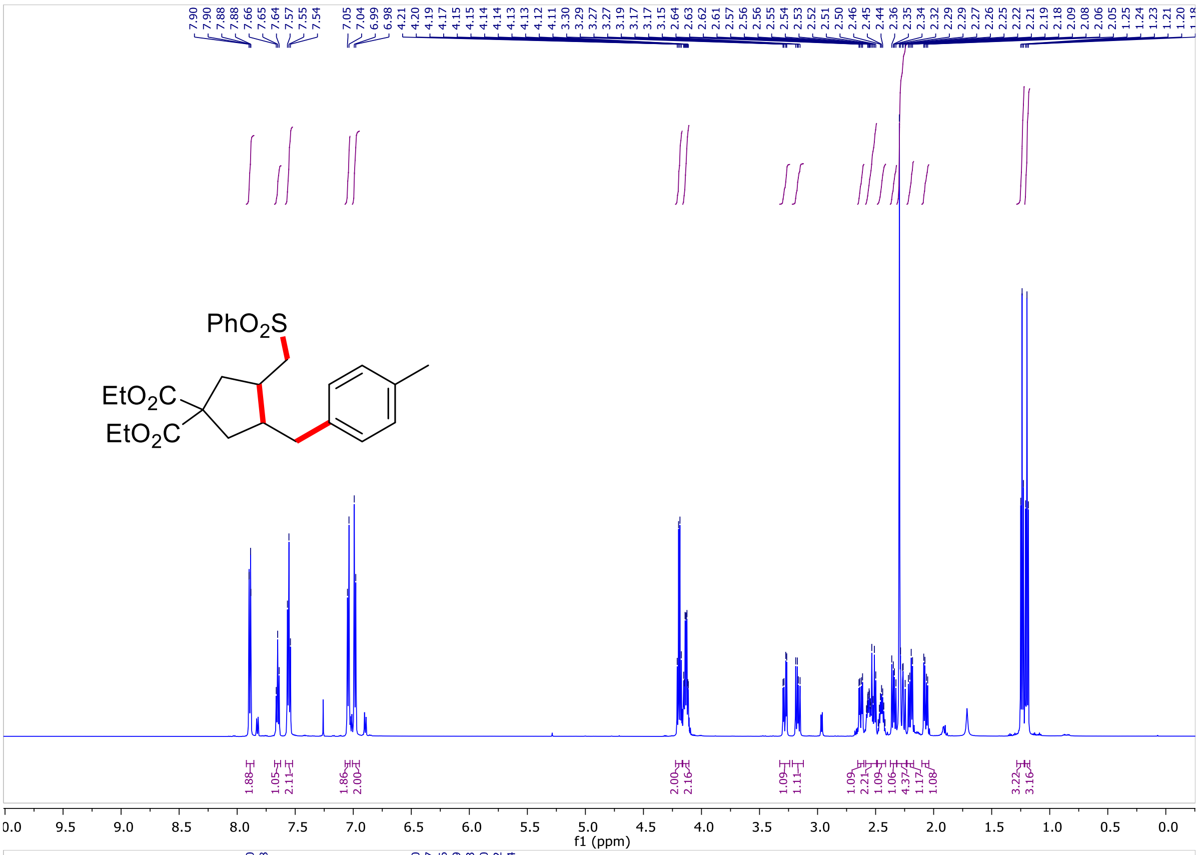
7.90 7.90 7.88 7.66 7.65 7.64 7.57 7.55 7.54 7.05 7.04 6.99 6.98 4.21 4.19 4.17 4.15 4.15 4.14 4.14 4.13 4.13 4.12 4.11 3.30 3.29 3.27 3.27 3.19 3.17 3.17 3.15 2.64 2.63 2.62 2.61 2.57 2.56 2.56 2.55 2.54 2.53 2.52 2.51 2.50 2.46 2.45 2.44 2.36 2.35 2.34 2.32 2.29 2.29 2.27 2.26 2.25 2.22 2.21 2.19 2.18 2.09 2.08 2.06 2.05 1.25 1.24 1.23 1.21 1.20
PhO2S
EtO2C
EtO2C
1.88 1.05 2.11 1.86 2.00 2.00 2.16 1.09 1.11 1.09 2.21 1.09 1.06 4.37 1.17 1.08 3.22 3.16
f1 (ppm)

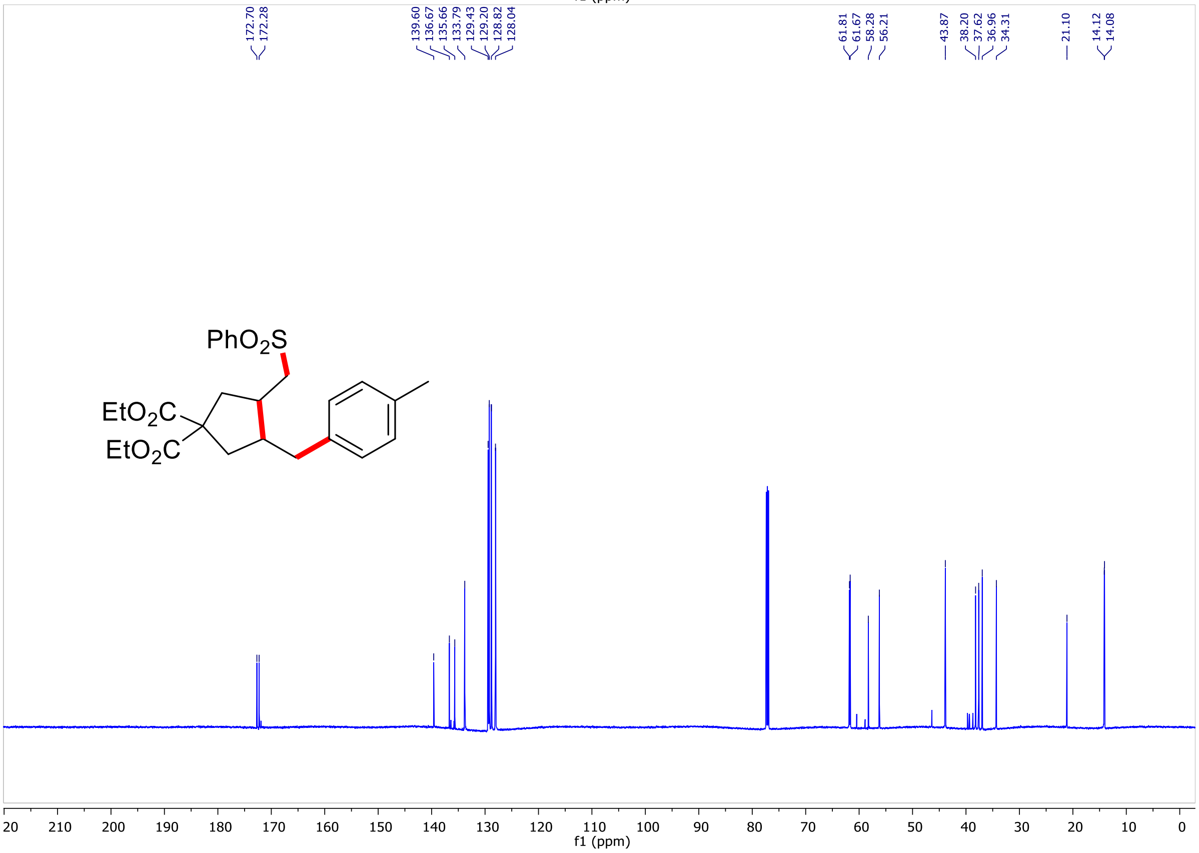
172.70 172.28 139.60 136.67 135.66 133.79 129.43 129.20 128.82 128.04 61.81 61.67 58.28 56.21 43.87 38.20 37.62 36.96 34.31 21.10 14.12 14.08
PhO2S
EtO2C
EtO2C
f1 (ppm)

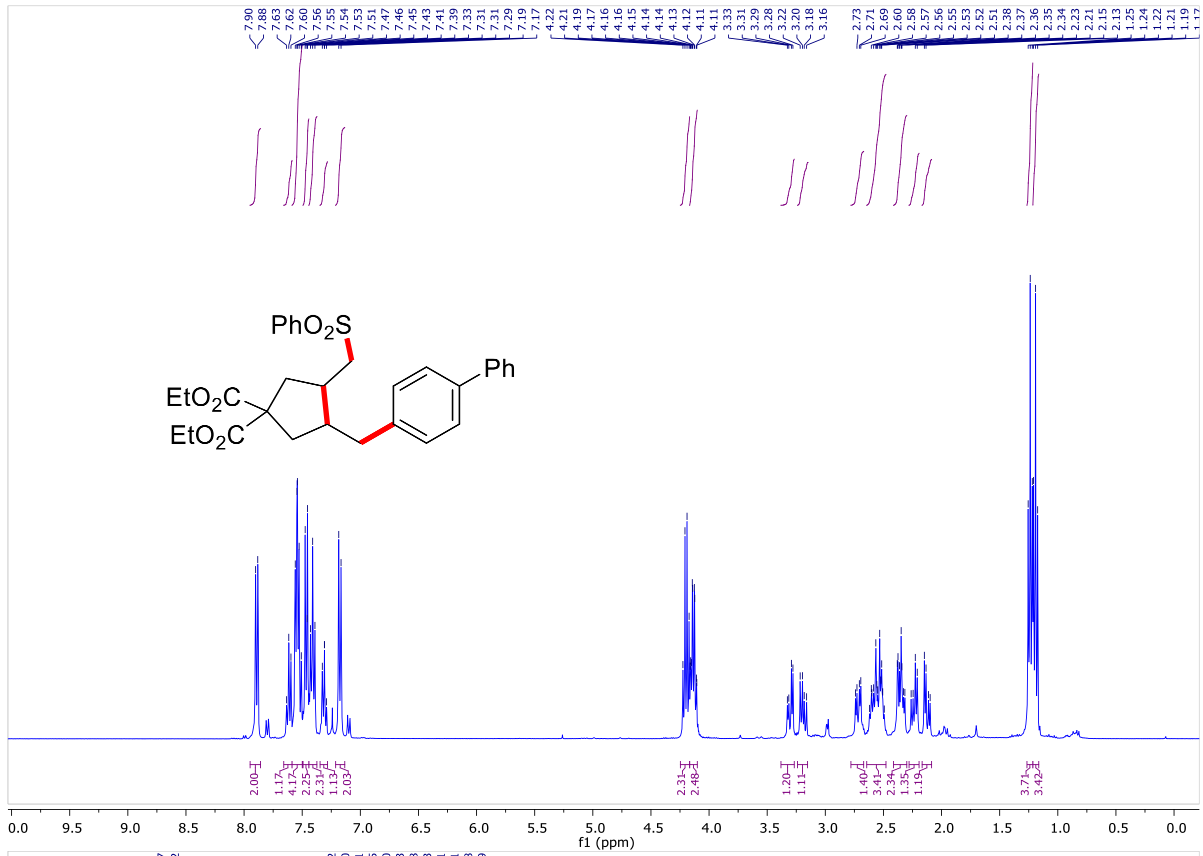

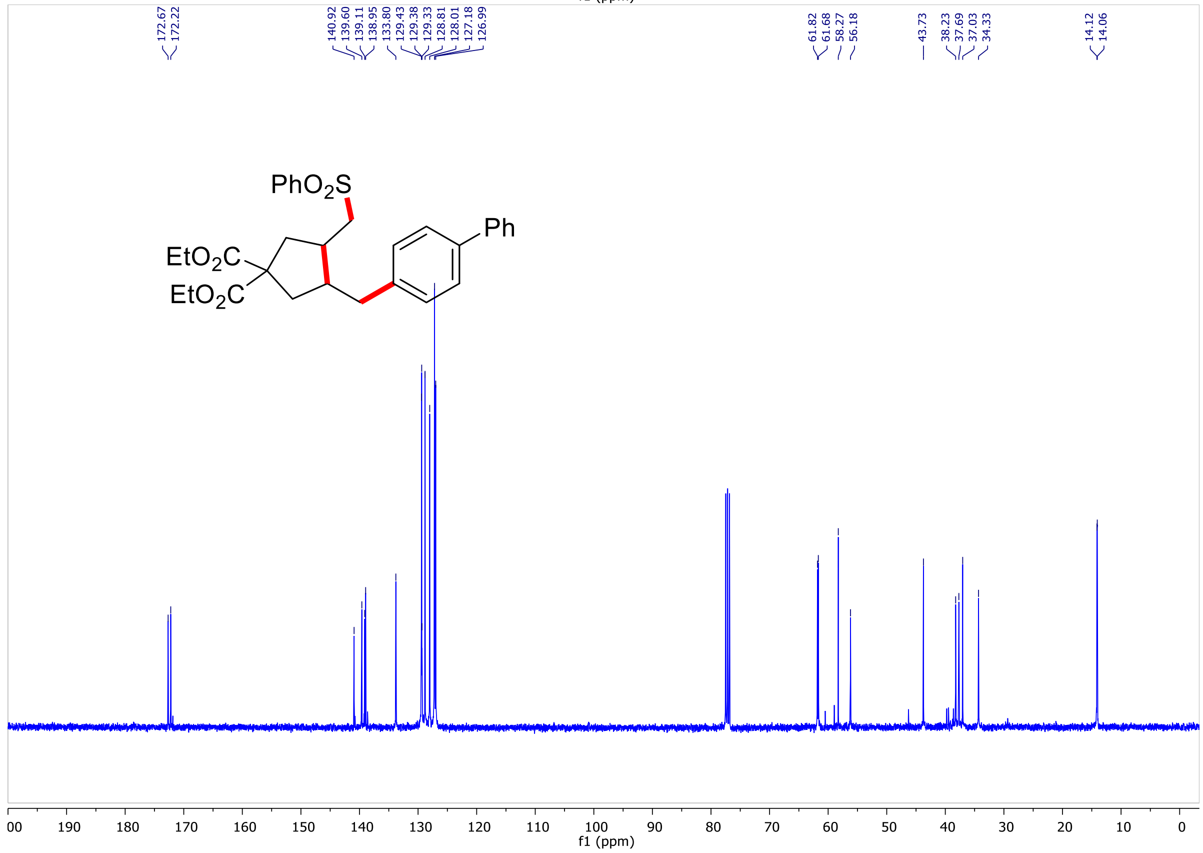

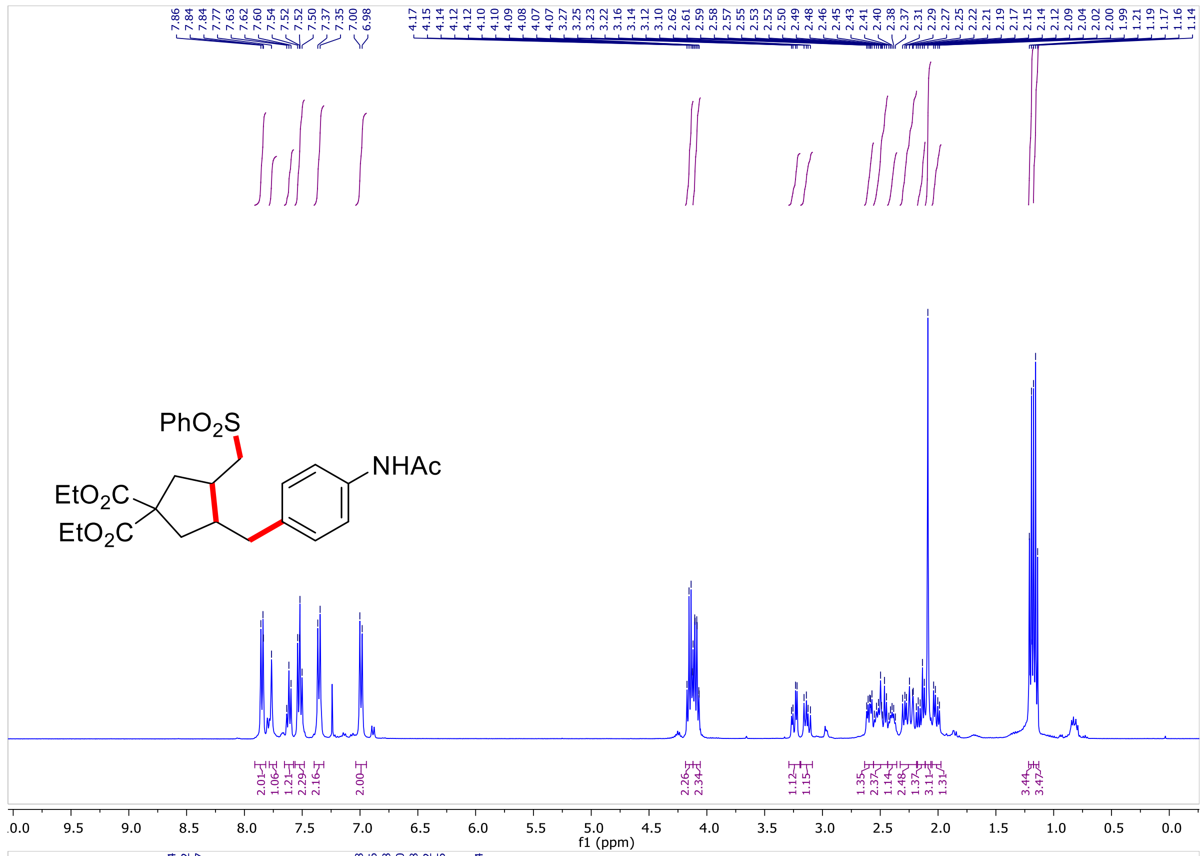

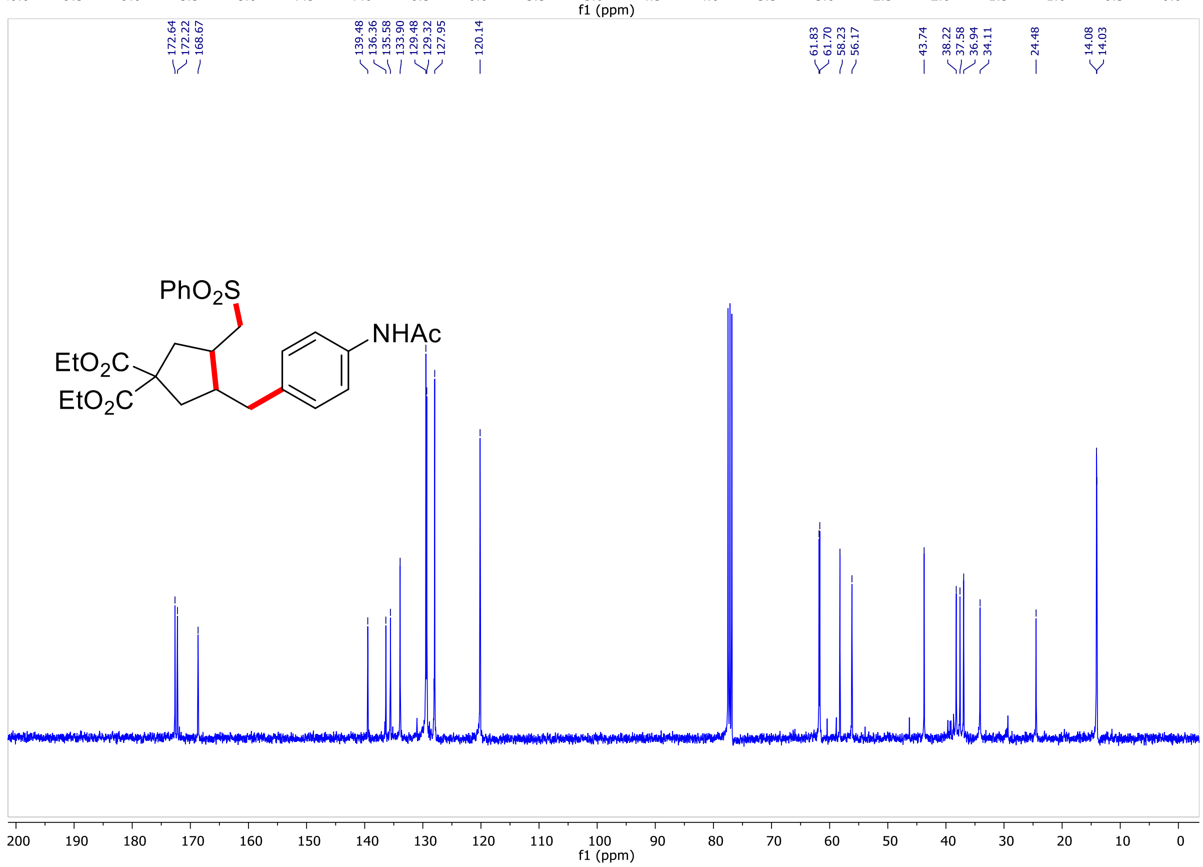

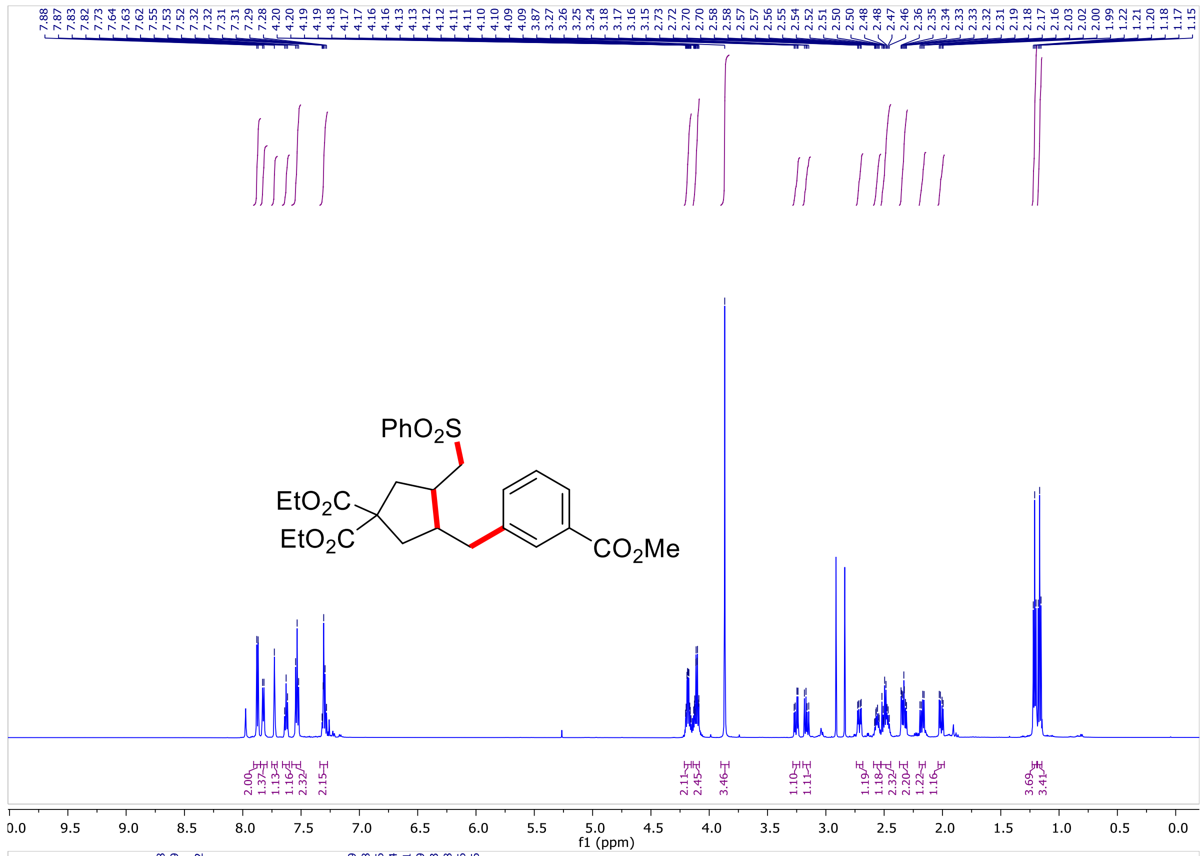

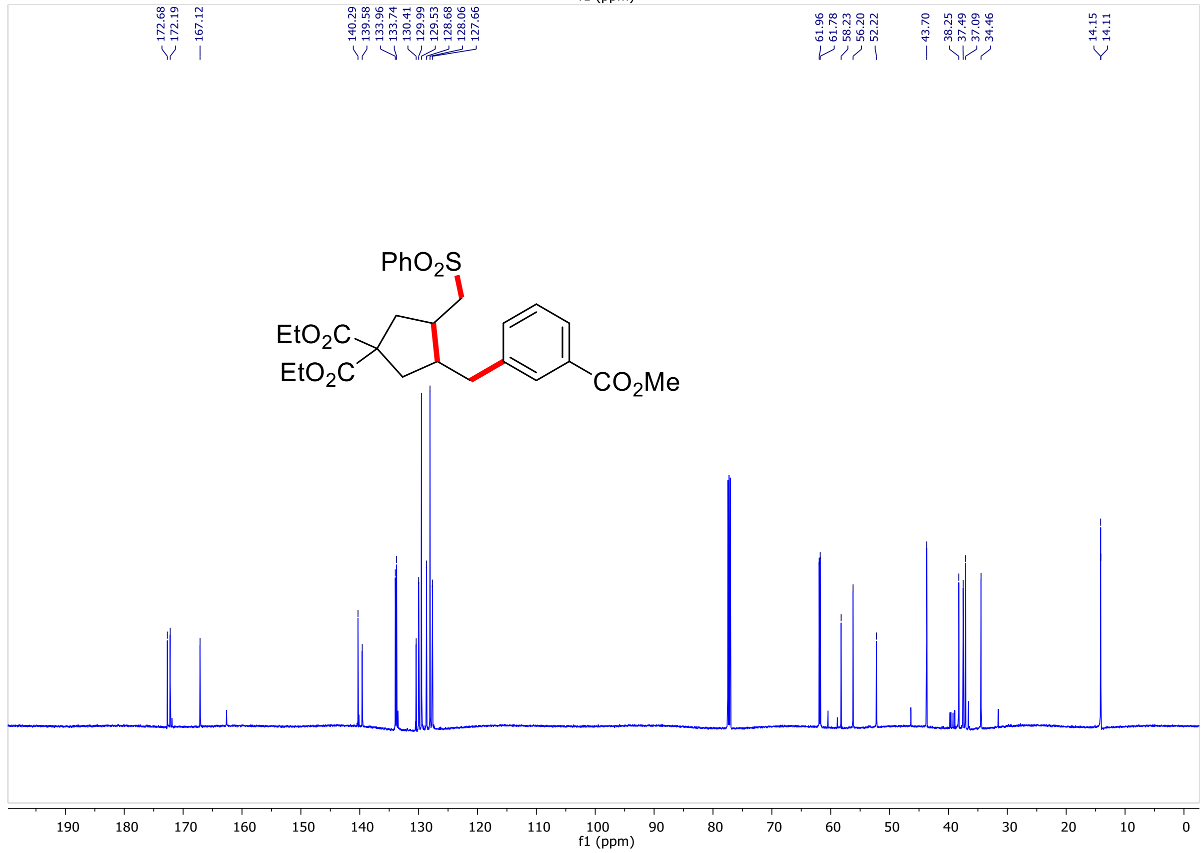

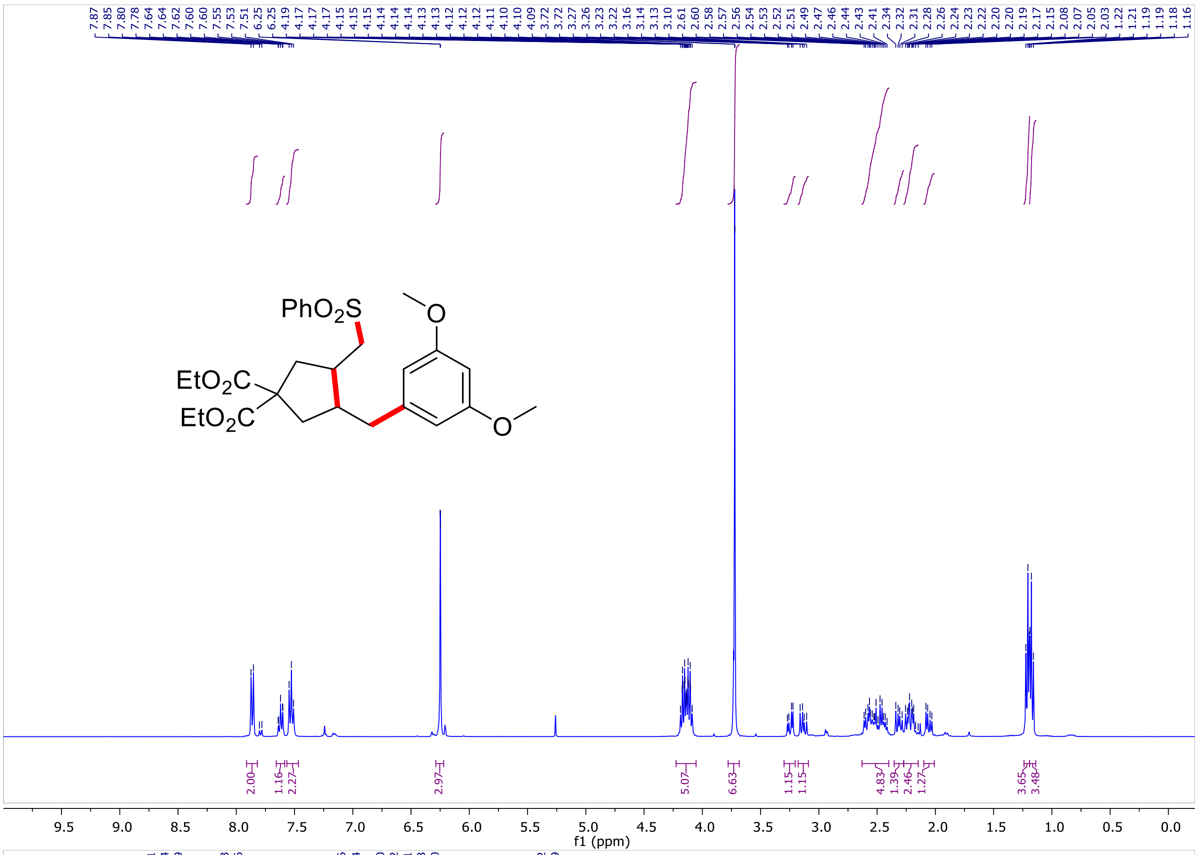
7.87 7.85 7.80 7.78 7.64 7.64 7.62 7.60 7.60 7.55 7.53 7.51 6.25 6.25 4.19 4.17 4.17 4.15 4.15 4.15 4.14 4.14 4.14 4.13 4.13 4.13 4.12 4.12 4.12 4.11 4.10 4.09 3.72 3.72 3.27 3.26 3.23 3.22 3.16 3.14 3.13 3.10 2.61 2.60 2.58 2.57 2.56 2.54 2.53 2.52 2.51 2.49 2.47 2.46 2.44 2.43 2.41 2.34 2.32 2.31 2.28 2.26 2.24 2.23 2.22 2.20 2.20 2.19 2.17 2.15 2.08 2.07 2.05 2.03 1.22 1.21 1.19 1.19 1.18 1.16
PhO2S
O
EtO2C
EtO2C
O
2.00 1.16 2.27 2.97 5.07 6.63 1.15 1.15 4.83 1.39 2.46 1.27 3.65 3.48
9.5 9.0 8.5 8.0 7.5 7.0 6.5 6.0 5.5 5.0 4.5 4.0 3.5 3.0 2.5 2.0 1.5 1.0 0.5 0.0
f1 (ppm)

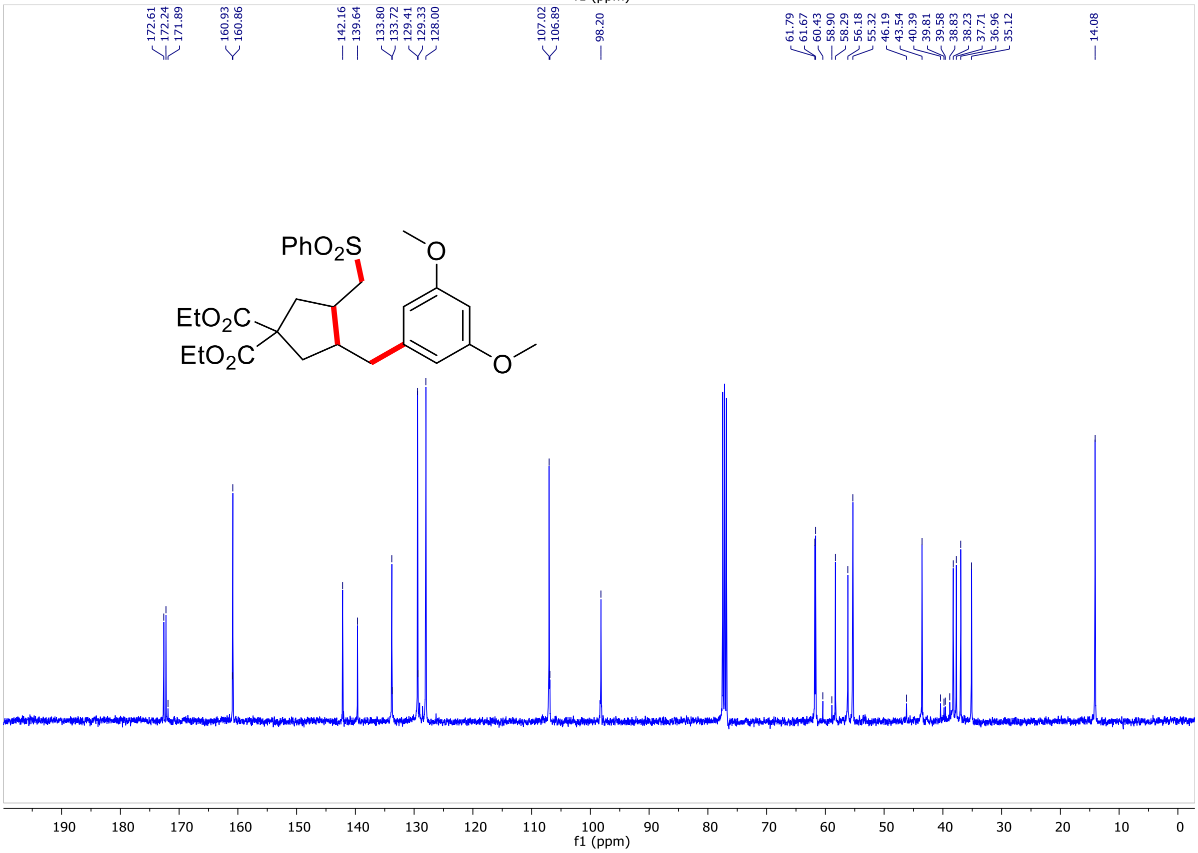
172.61 172.24 171.89 160.93 160.86 142.16 139.64 133.80 133.72 129.41 129.33 128.00 107.02 106.89 98.20 61.79 61.67 60.43 58.90 58.29 56.18 55.32 46.19 43.54 40.39 39.81 39.58 38.83 38.23 37.71 36.96 35.12 14.08
PhO2S
O
EtO2C
EtO2C
O
190 180 170 160 150 140 130 120 110 100 90 80 70 60 50 40 30 20 10 0
f1 (ppm)

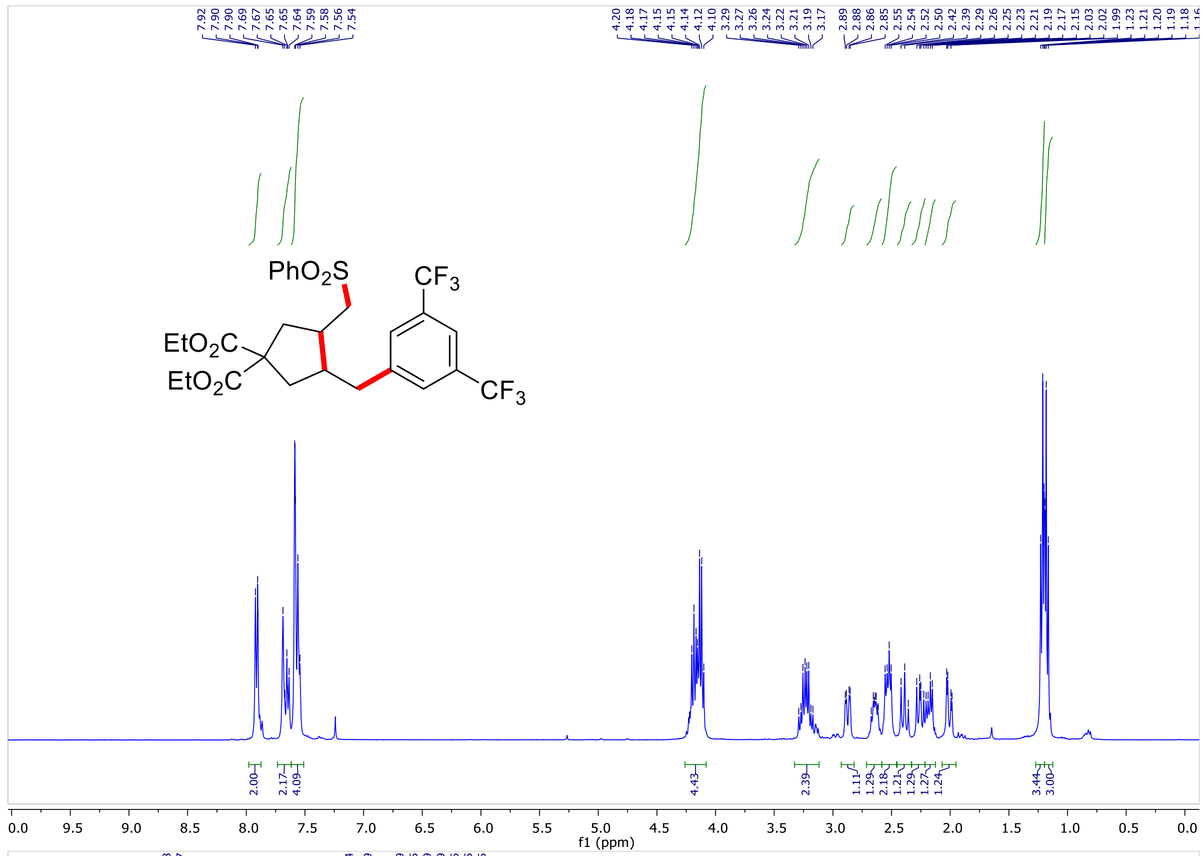

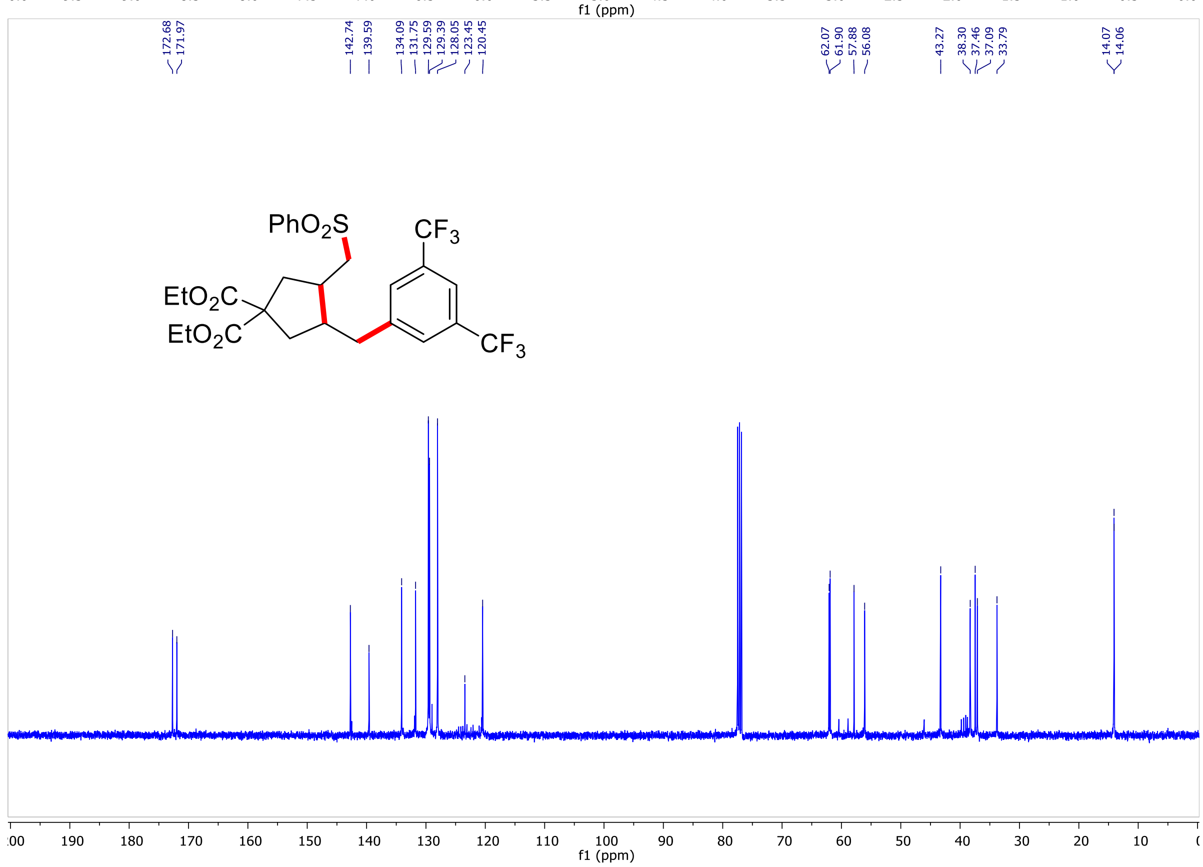

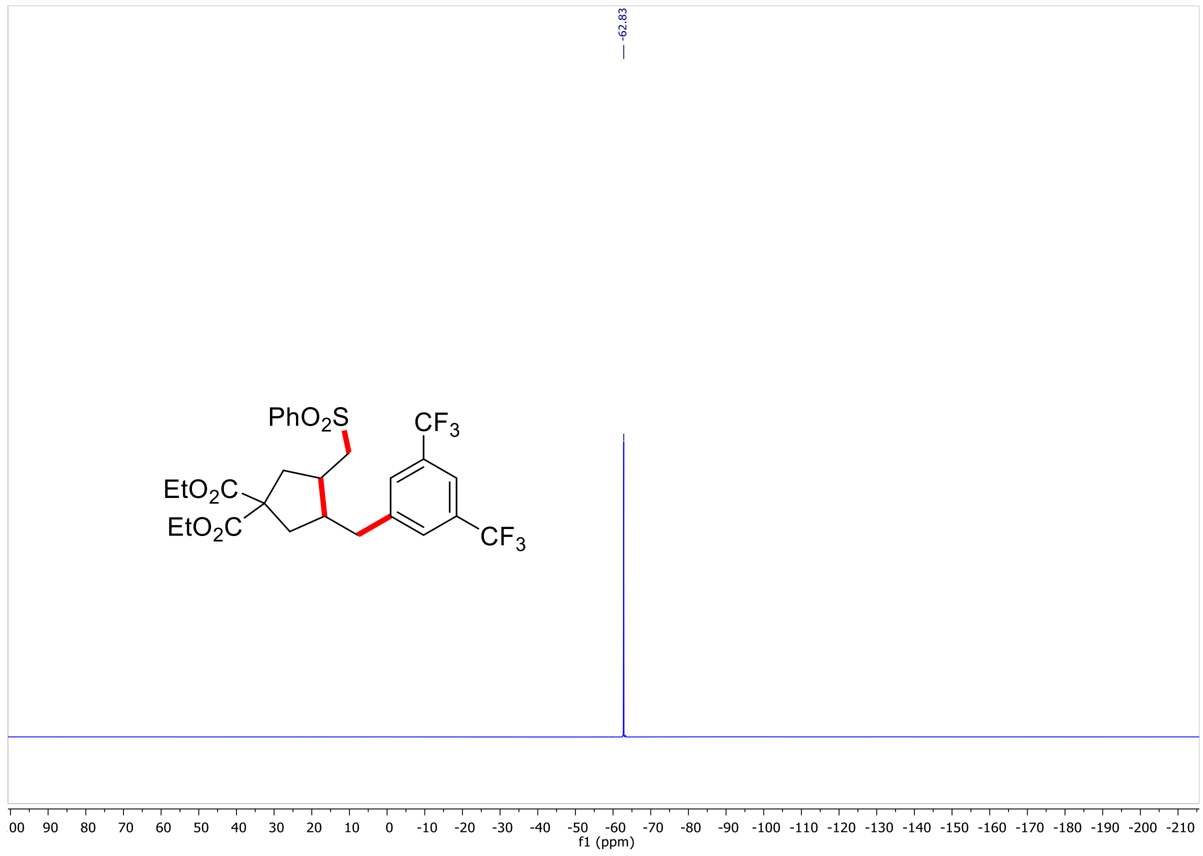
-62.83
PhO2S
CF3
EtO2C
EtO2C
CF3
00 90 80 70 60 50 40 30 20 10 0 -10 -20 -30 -40 -50 -60 -70 -80 -90 -100 -110 -120 -130 -140 -150 -160 -170 -180 -190 -200 -210
f1 (ppm)

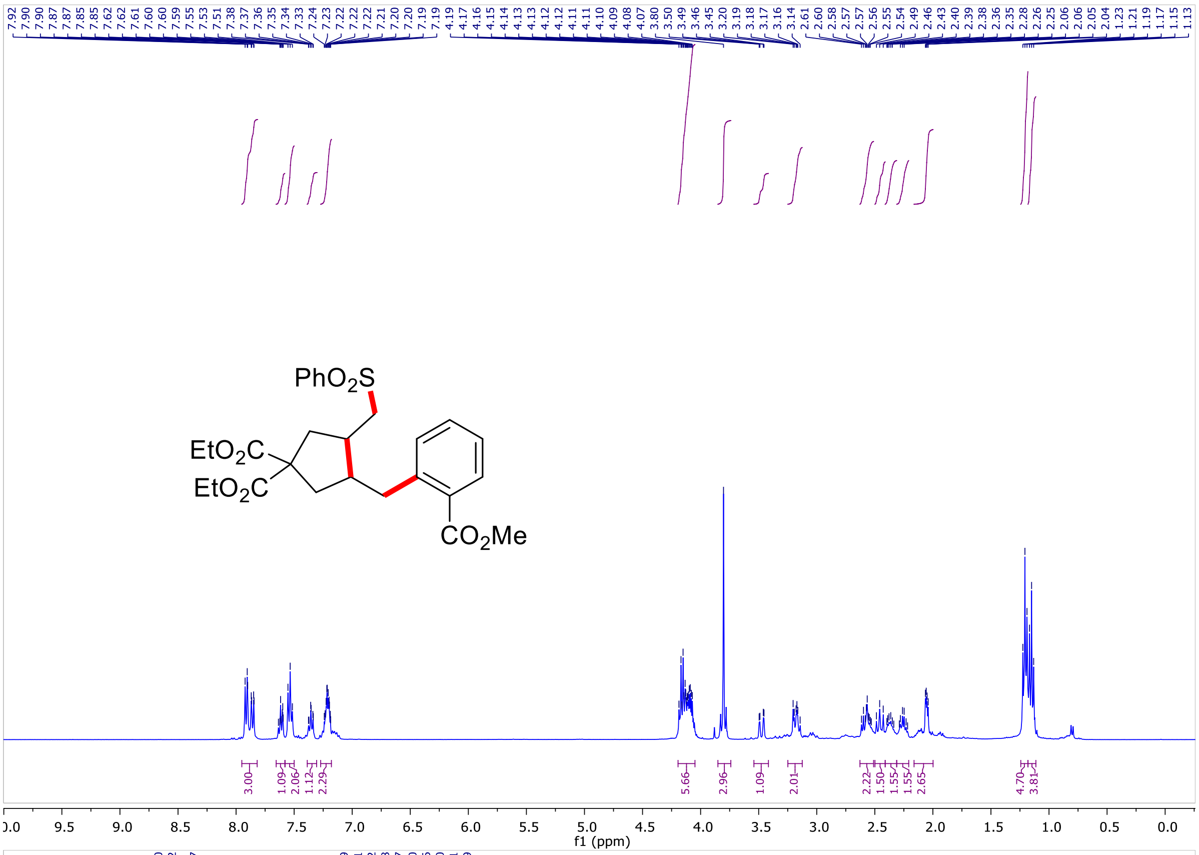
7.92 7.90 7.90 7.87 7.87 7.85 7.62 7.62 7.61 7.60 7.60 7.59 7.55 7.53 7.51 7.38 7.37 7.36 7.35 7.34 7.33 7.24 7.23 7.22 7.22 7.22 7.21 7.20 7.20 7.19 7.19 4.19 4.17 4.16 4.15 4.14 4.13 4.13 4.12 4.12 4.11 4.11 4.10 4.09 4.08 4.07 3.80 3.50 3.49 3.46 3.45 3.20 3.19 3.18 3.17 3.16 3.14 2.61 2.60 2.58 2.57 2.57 2.56 2.55 2.54 2.49 2.46 2.43 2.40 2.39 2.38 2.36 2.35 2.28 2.26 2.25 2.06 2.06 2.05 2.04 1.23 1.21 1.19 1.17 1.15 1.13
PhO2S
EtO2C
EtO2C
CO2Me
3.00 1.09 2.06 1.12 2.29 5.66 2.96 1.09 2.01 2.22 1.50 1.55 1.55 2.65 4.70 3.81
f1 (ppm)

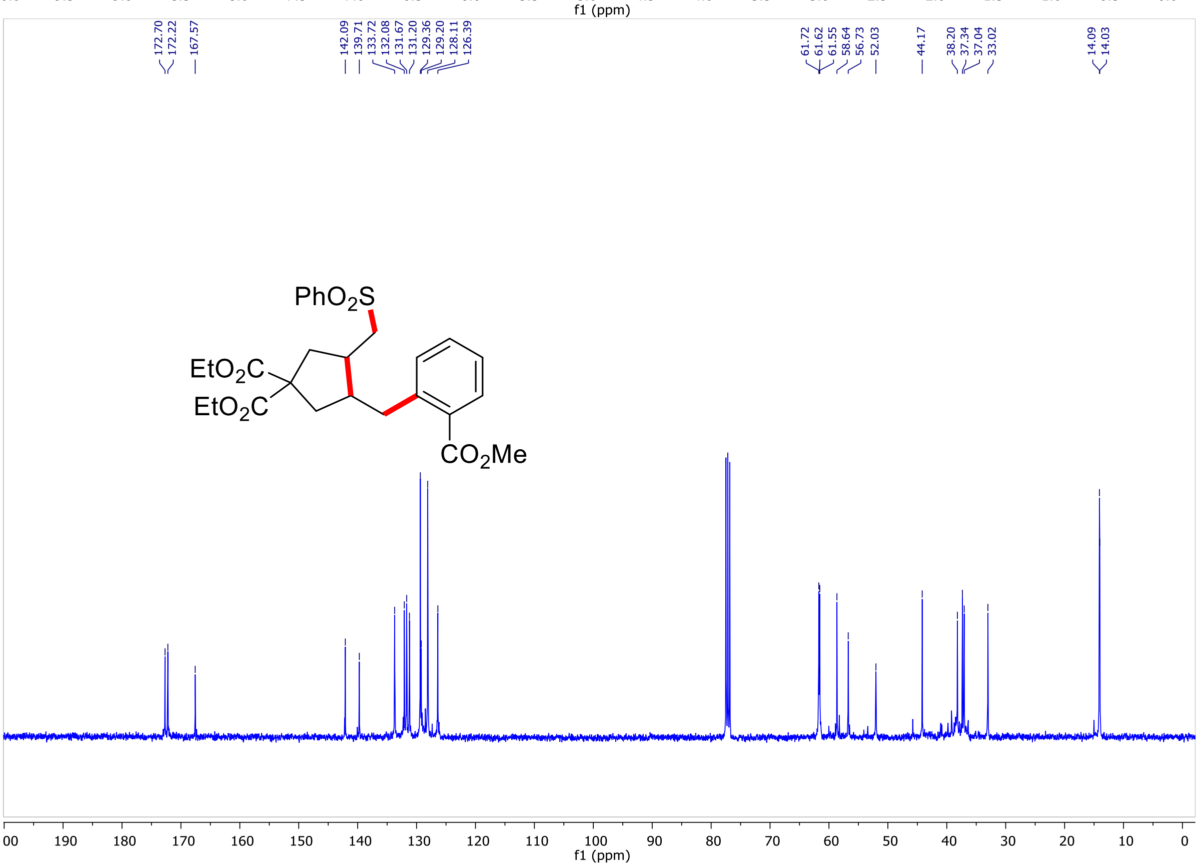
172.70 172.22 167.57 142.09 139.71 133.72 132.08 131.67 131.20 129.36 129.20 128.11 126.39 61.72 61.62 61.55 58.64 56.73 52.03 44.17 38.20 37.34 37.04 33.02 14.09 14.03
PhO2S
EtO2C
EtO2C
CO2Me
f1 (ppm)

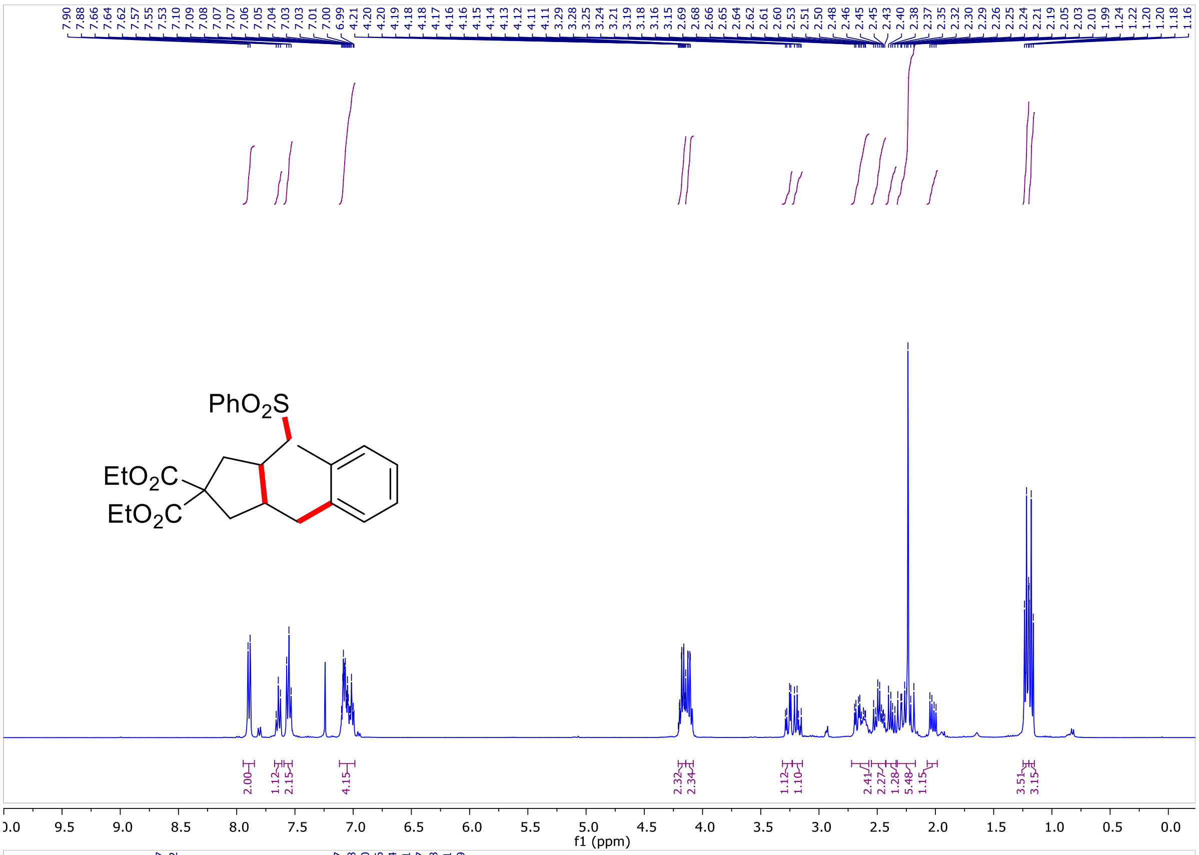
7.90 7.88 7.66 7.64 7.62 7.57 7.55 7.53 7.10 7.09 7.08 7.07 7.07 7.06 7.05 7.04 7.03 7.03 7.01 7.00 6.99 4.21 4.20 4.19 4.18 4.18 4.17 4.16 4.16 4.15 4.14 4.13 4.12 4.11 4.11 3.29 3.28 3.25 3.24 3.21 3.19 3.18 3.16 3.15 2.69 2.68 2.66 2.65 2.64 2.62 2.61 2.60 2.53 2.51 2.50 2.48 2.46 2.45 2.45 2.43 2.40 2.38 2.37 2.35 2.32 2.30 2.29 2.26 2.25 2.24 2.21 2.19 2.05 2.03 2.01 1.99 1.24 1.22 1.20 1.20 1.18 1.16
PhO2S
EtO2C
EtO2C
2.00 1.12 2.15 4.15 2.32 2.34 1.12 1.10 2.41 2.27 1.28 5.48 1.15 3.51 3.15
9.5 9.0 8.5 8.0 7.5 7.0 6.5 6.0 5.5 5.0 4.5 4.0 3.5 3.0 2.5 2.0 1.5 1.0 0.5 0.0
f1 (ppm)

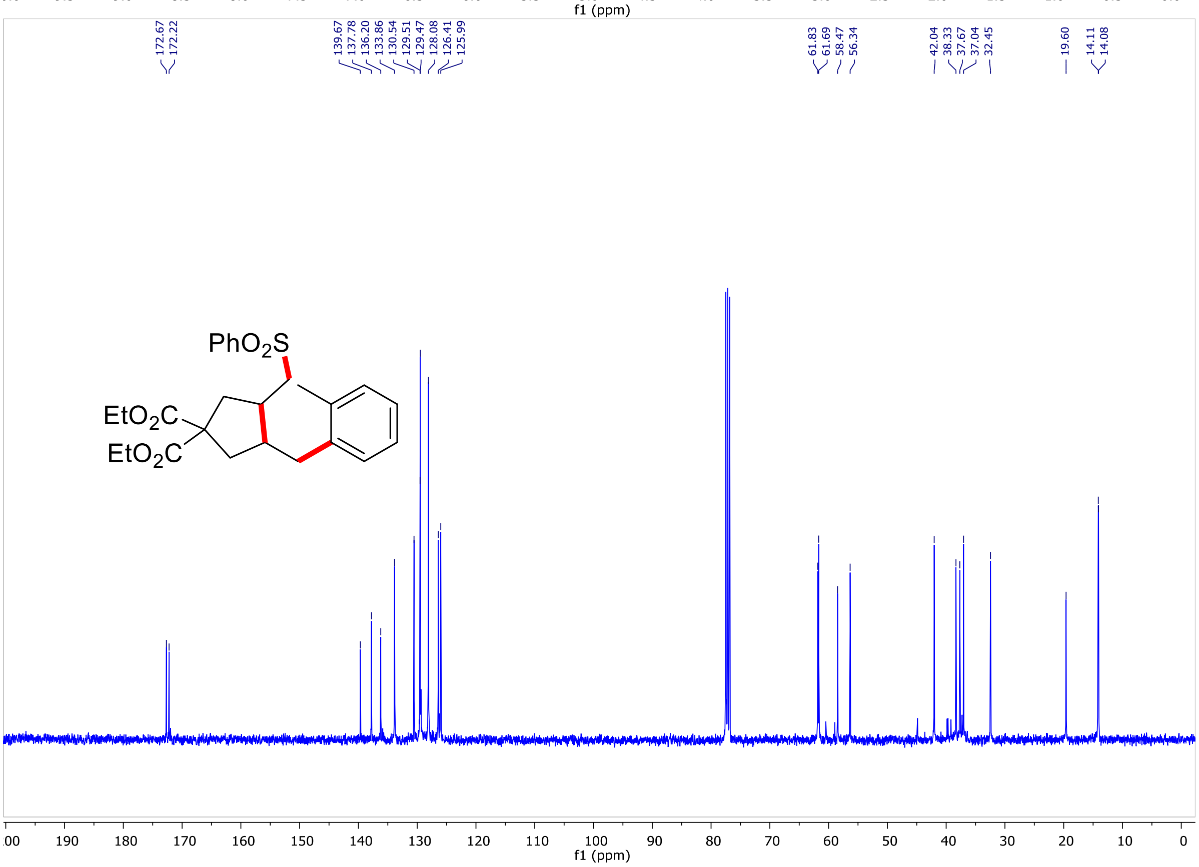
172.67 172.22
139.67 137.78 136.20 133.86 130.54 129.51 129.47 128.08 126.41 125.99
61.83 61.69 58.47 56.34
42.04 38.33 37.67 37.04 32.45
19.60 14.11 14.08
PhO2S
EtO2C
EtO2C
190 180 170 160 150 140 130 120 110 100 90 80 70 60 50 40 30 20 10 0
f1 (ppm)

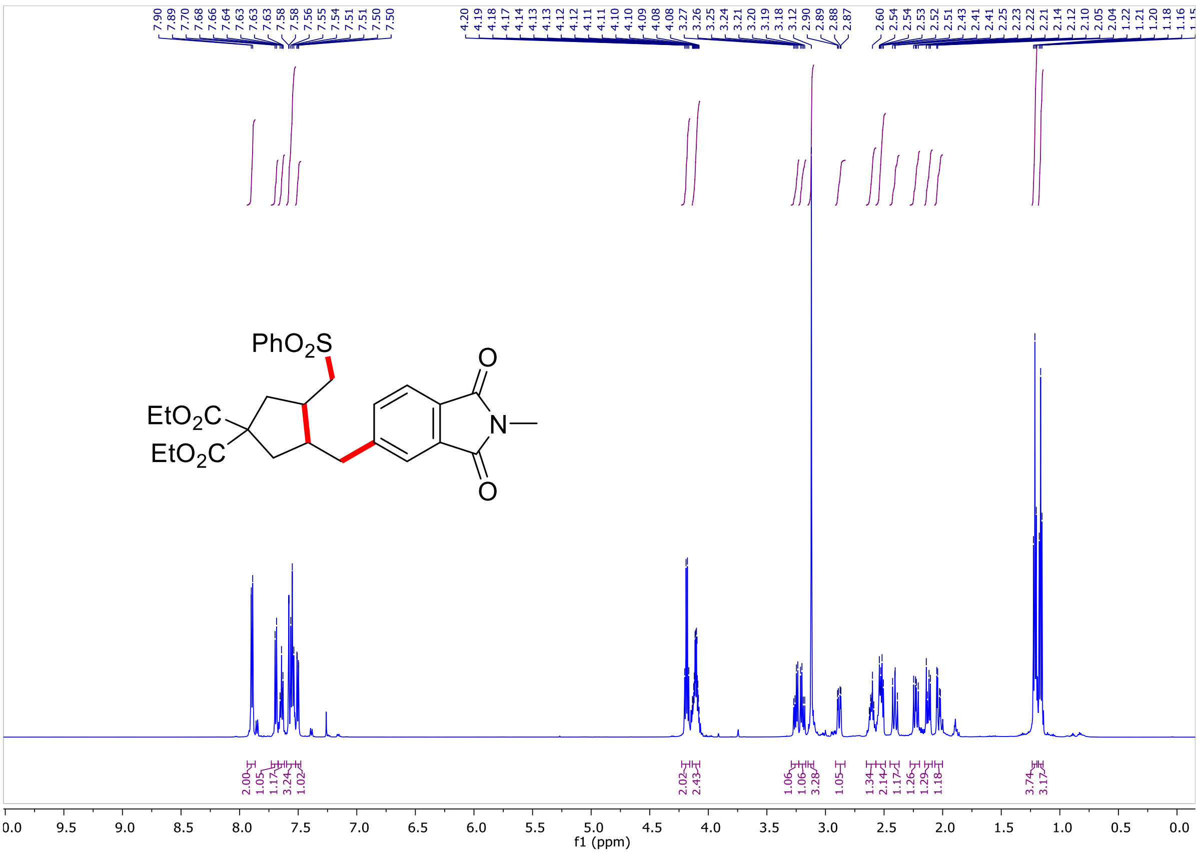
PhO2S
EtO2C
EtO2C
O
N
O
f1 (ppm)

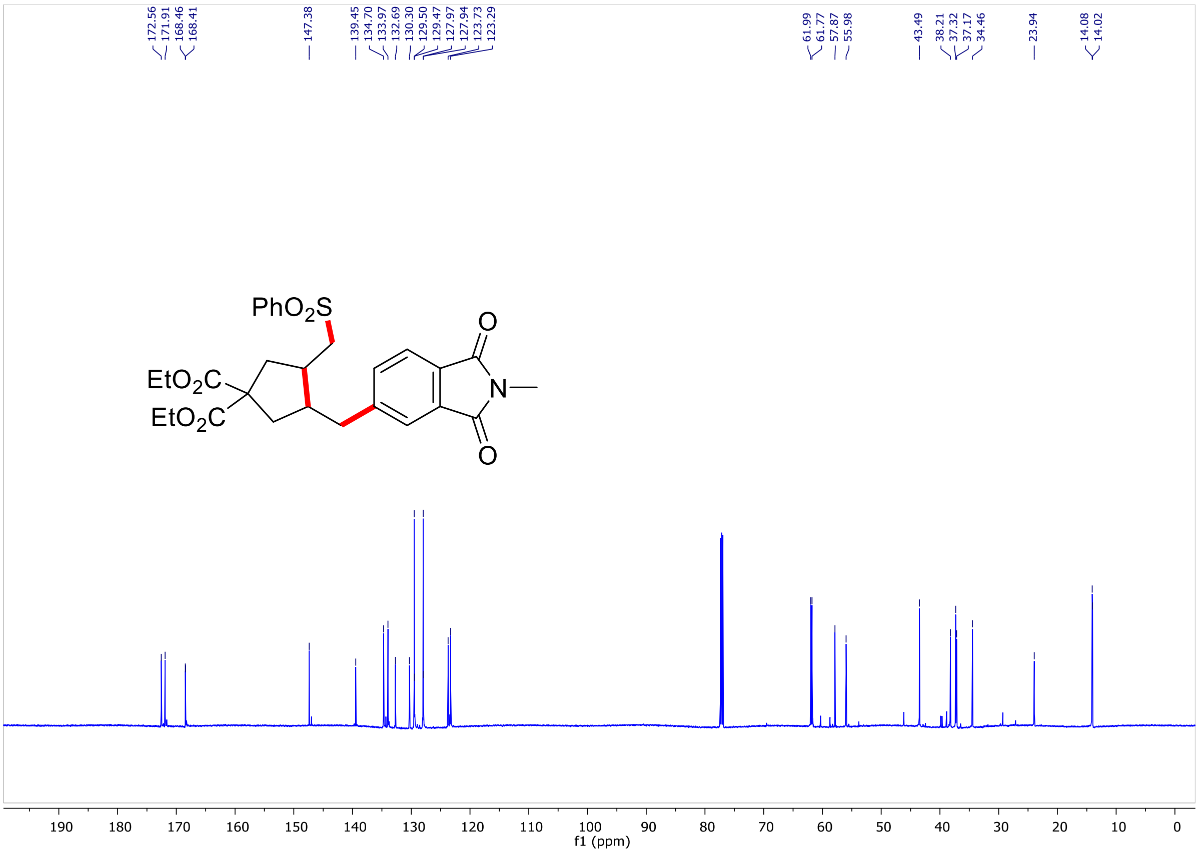
PhO2S
EtO2C
EtO2C
O
N
O
f1 (ppm)

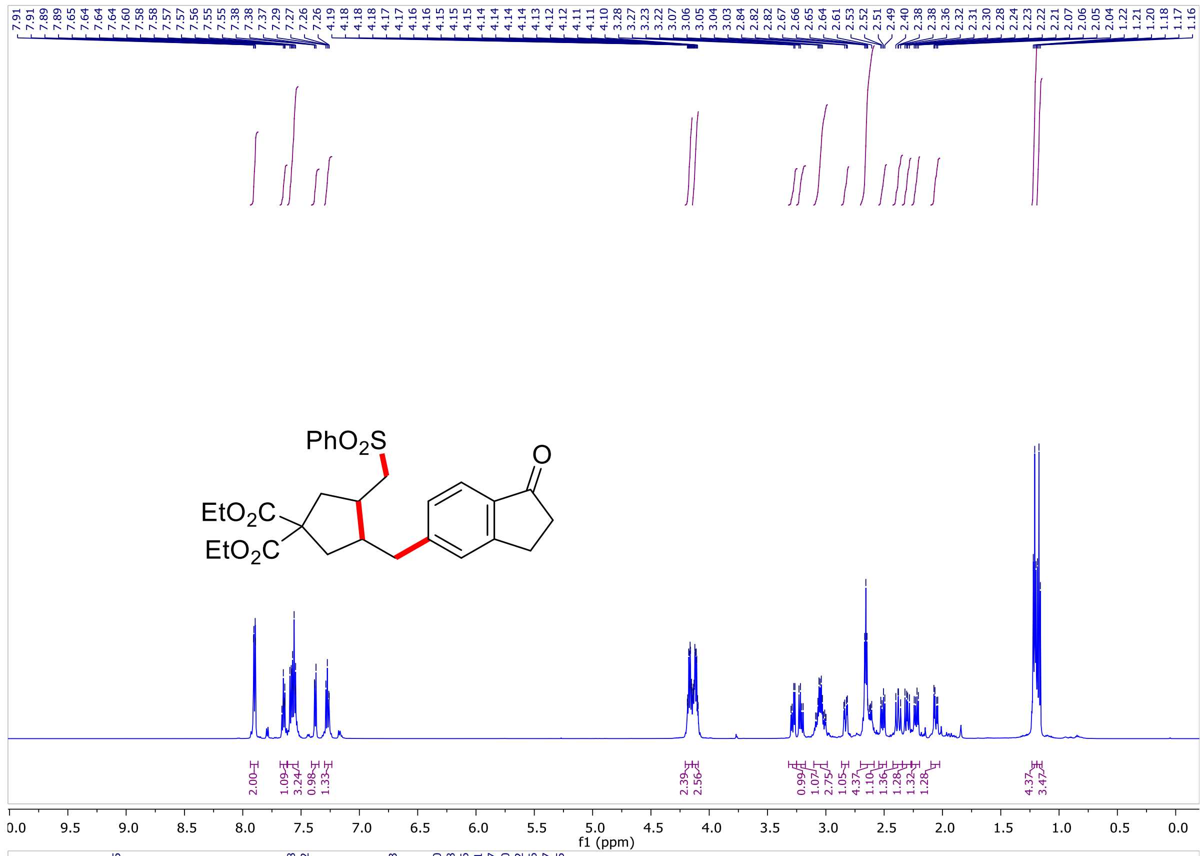

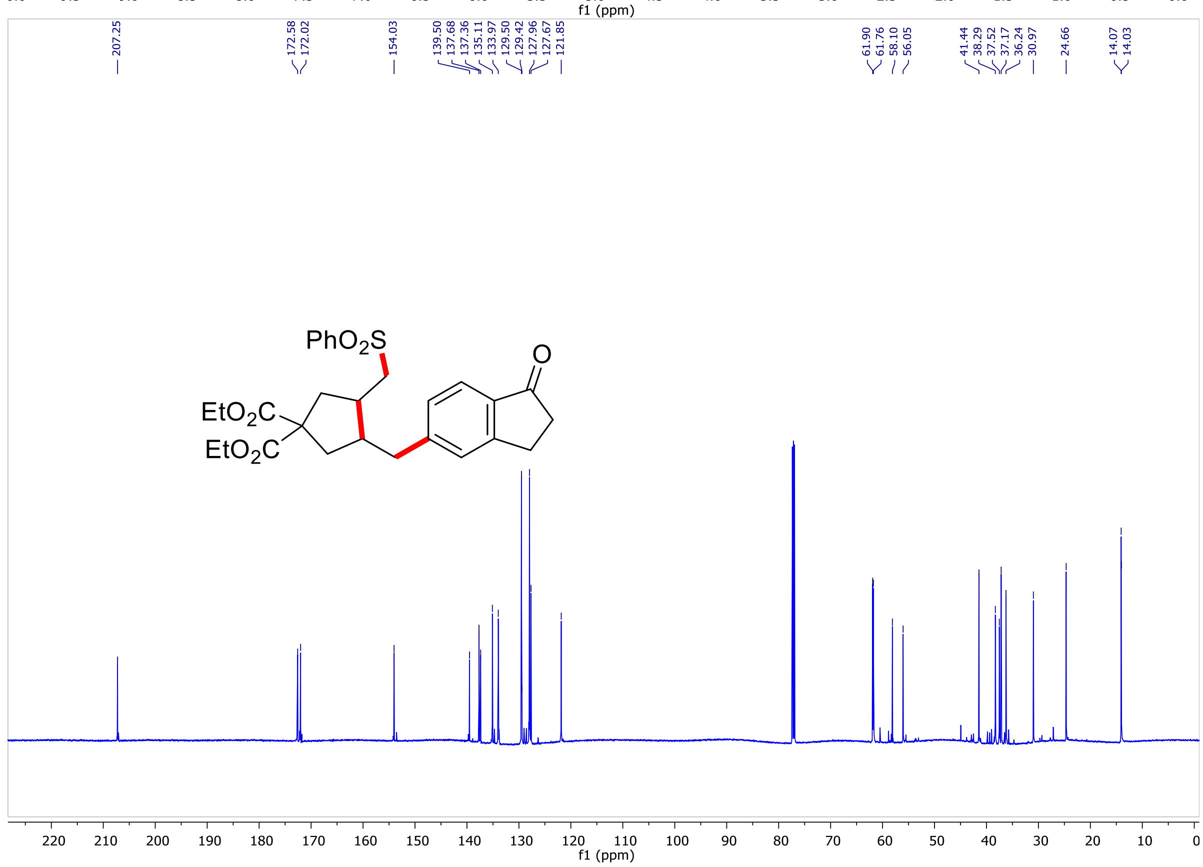

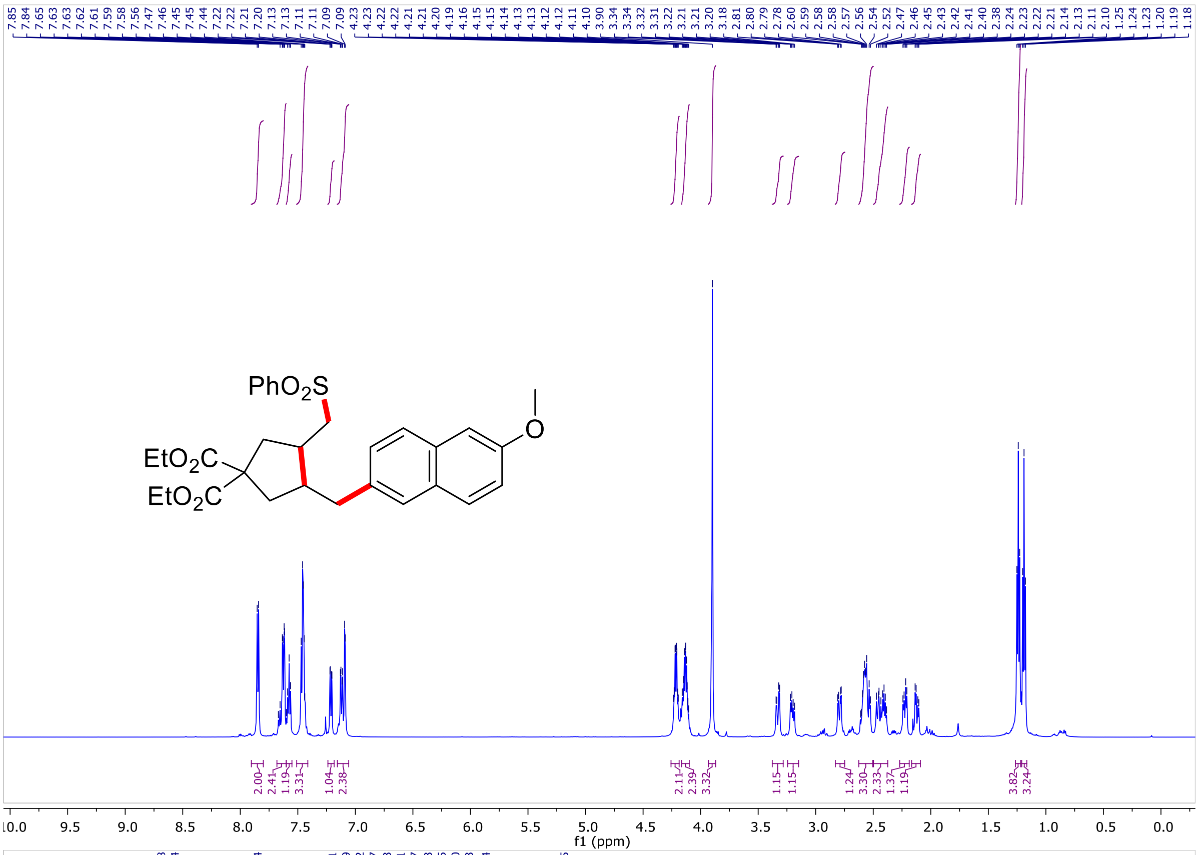
PhO2S
EtO2C
EtO2C
O
f1 (ppm)

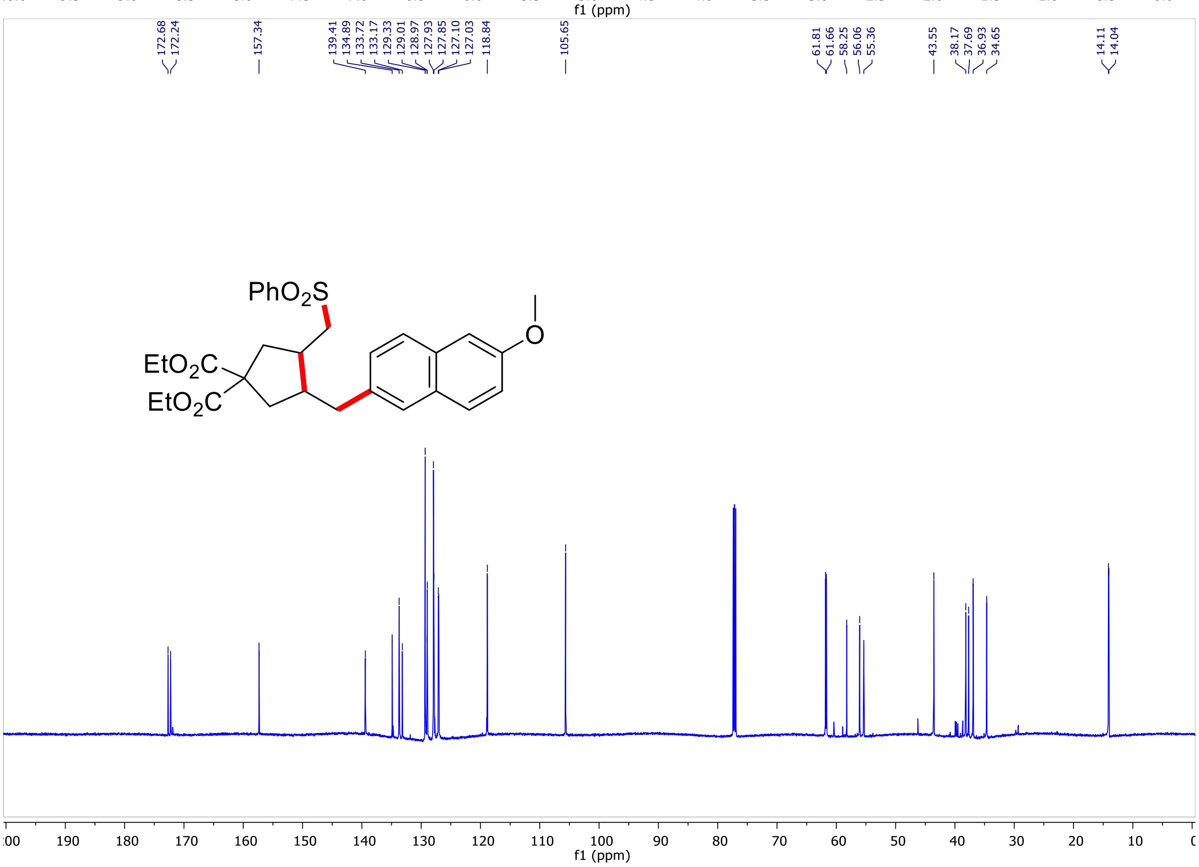
172.68
172.24
157.34
139.41
134.89
133.72
133.17
129.33
129.01
128.97
127.93
127.85
127.10
127.03
118.84
105.65
61.81
61.66
58.25
56.06
55.36
43.55
38.17
37.69
36.93
34.65
14.11
14.04
PhO2S
EtO2C
EtO2C
O
f1 (ppm)

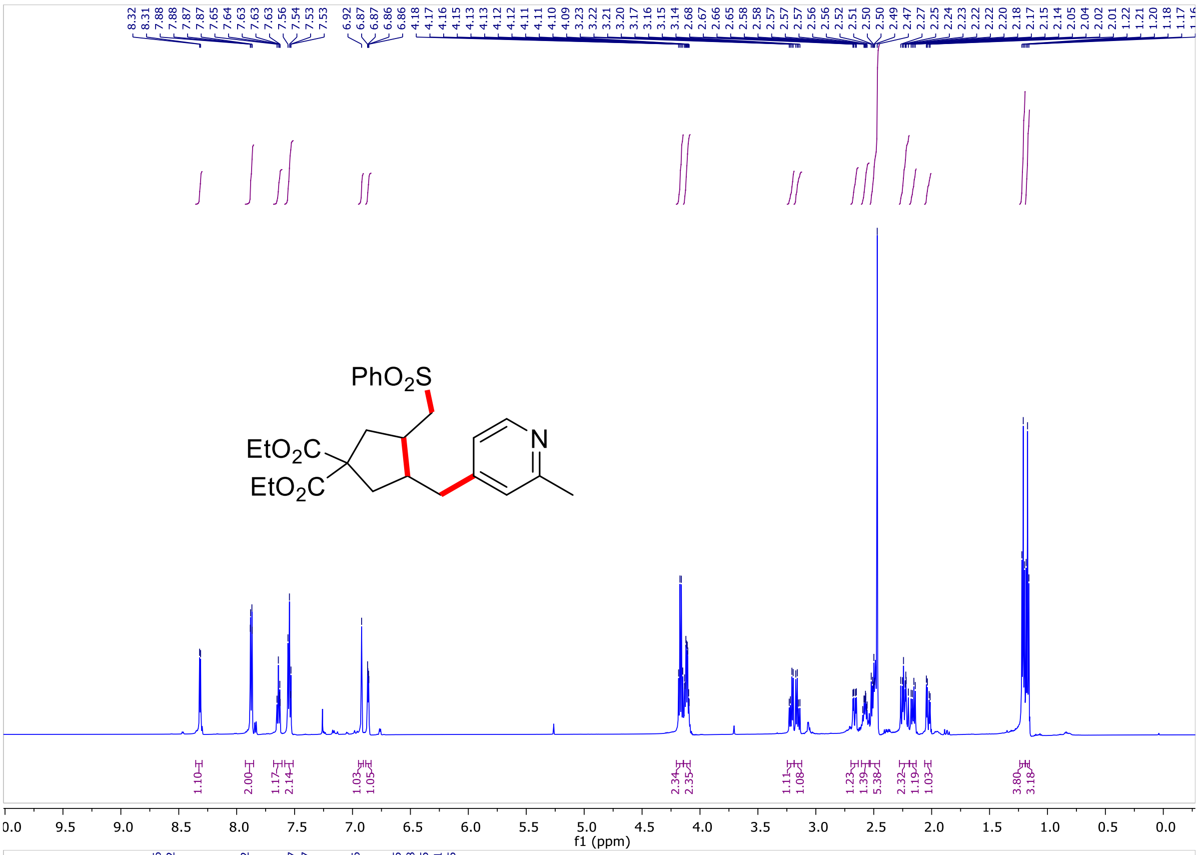
8.32 8.31 7.88 7.88 7.87 7.87 7.65 7.64 7.63 7.63 7.63 7.56 7.54 7.53 7.53 6.92 6.87 6.87 6.86 6.86 4.18 4.16 4.15 4.13 4.13 4.12 4.12 4.11 4.11 4.10 4.09 3.23 3.22 3.21 3.20 3.17 3.16 3.15 3.14 2.68 2.67 2.66 2.65 2.58 2.58 2.57 2.57 2.57 2.56 2.56 2.52 2.51 2.50 2.50 2.49 2.47 2.27 2.25 2.24 2.24 2.23 2.22 2.22 2.20 2.18 2.17 2.15 2.14 2.05 2.04 2.02 2.01 1.22 1.21 1.20 1.18 1.17
PhO2S
EtO2C
EtO2C
N
1.10
2.00
1.17
2.14
1.03
1.05
2.34
2.35
1.11
1.08
1.23
1.39
5.38
2.32
1.19
1.03
3.80
3.18
f1 (ppm)

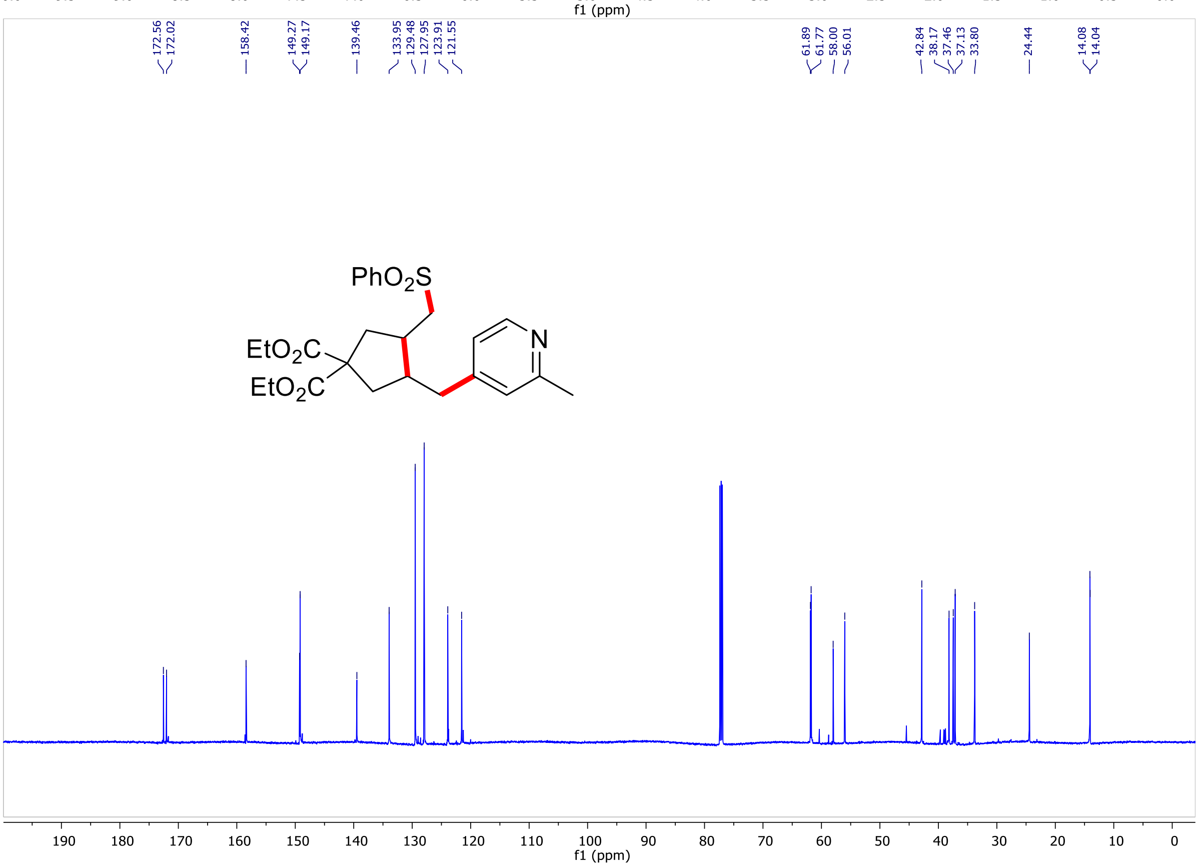
172.56 172.02 158.42 149.27 149.17 139.46 133.95 129.48 127.95 123.91 121.55 61.89 61.77 58.00 56.01 42.84 38.17 37.46 37.13 33.80 24.44 14.08 14.04
PhO2S
EtO2C
EtO2C
N
f1 (ppm)

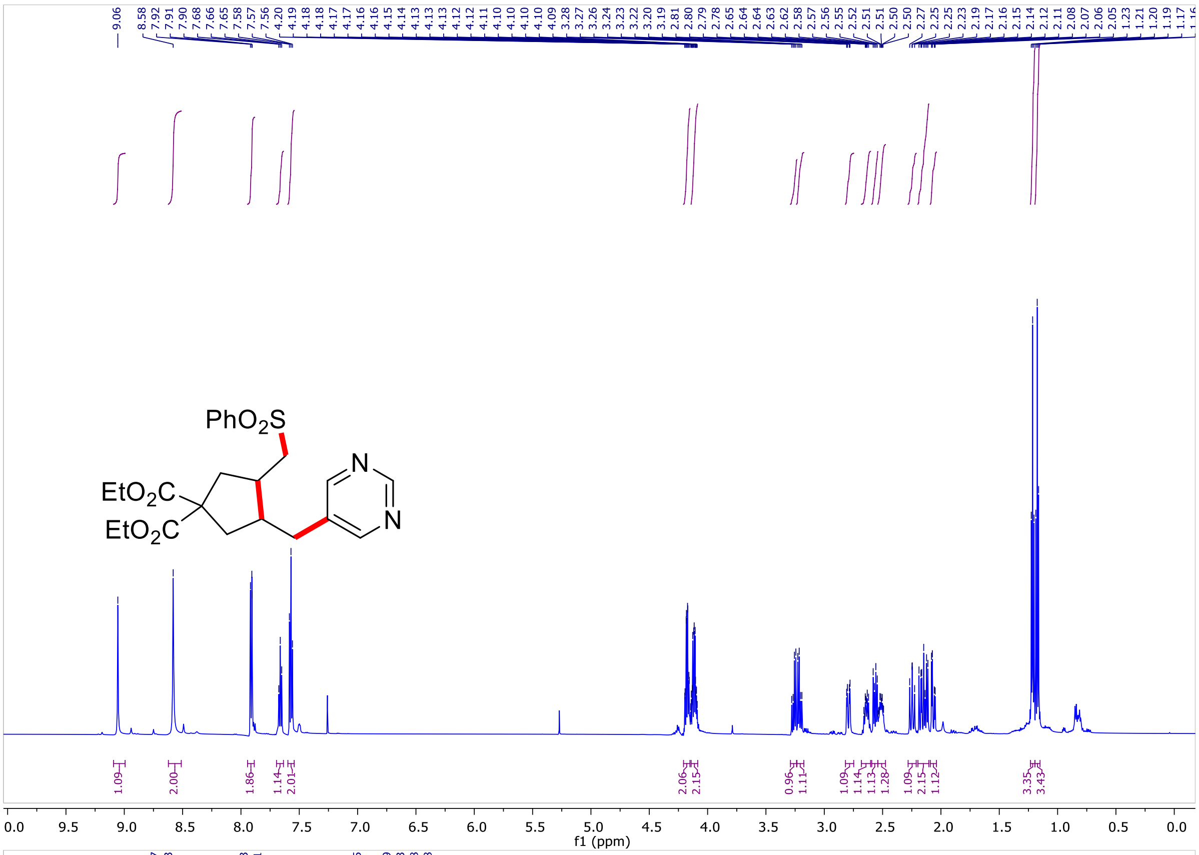
PhO2S
EtO2C
EtO2C
N
N
f1 (ppm)

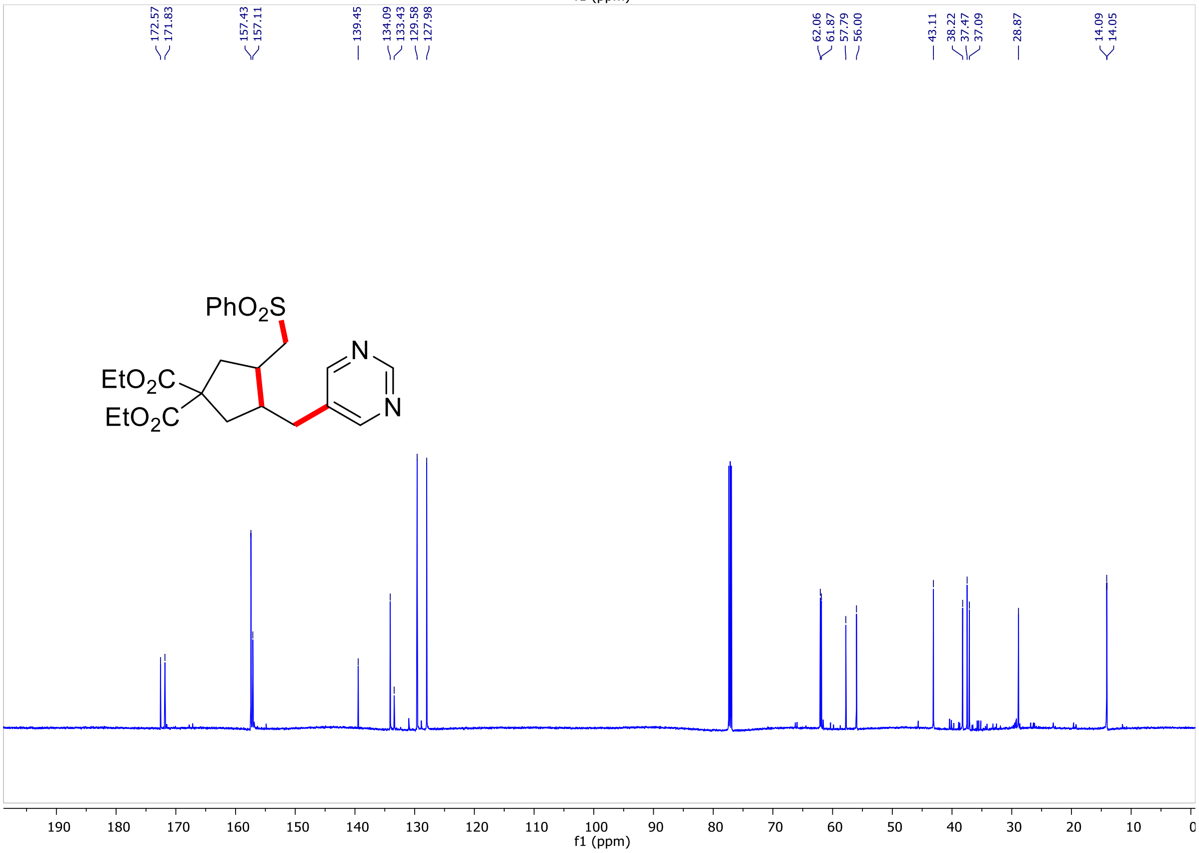
PhO2S
EtO2C
EtO2C
N
N
f1 (ppm)

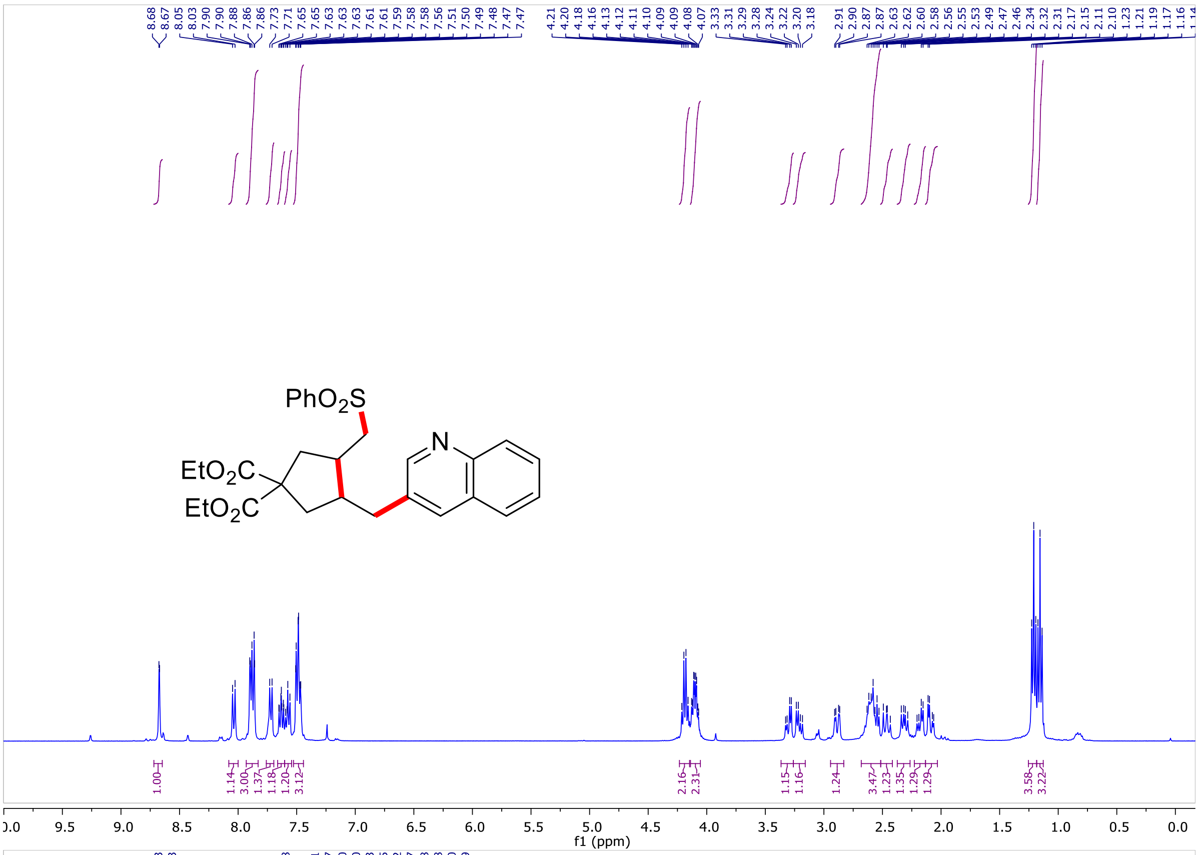
PhO2S
EtO2C
EtO2C
N
f1 (ppm)

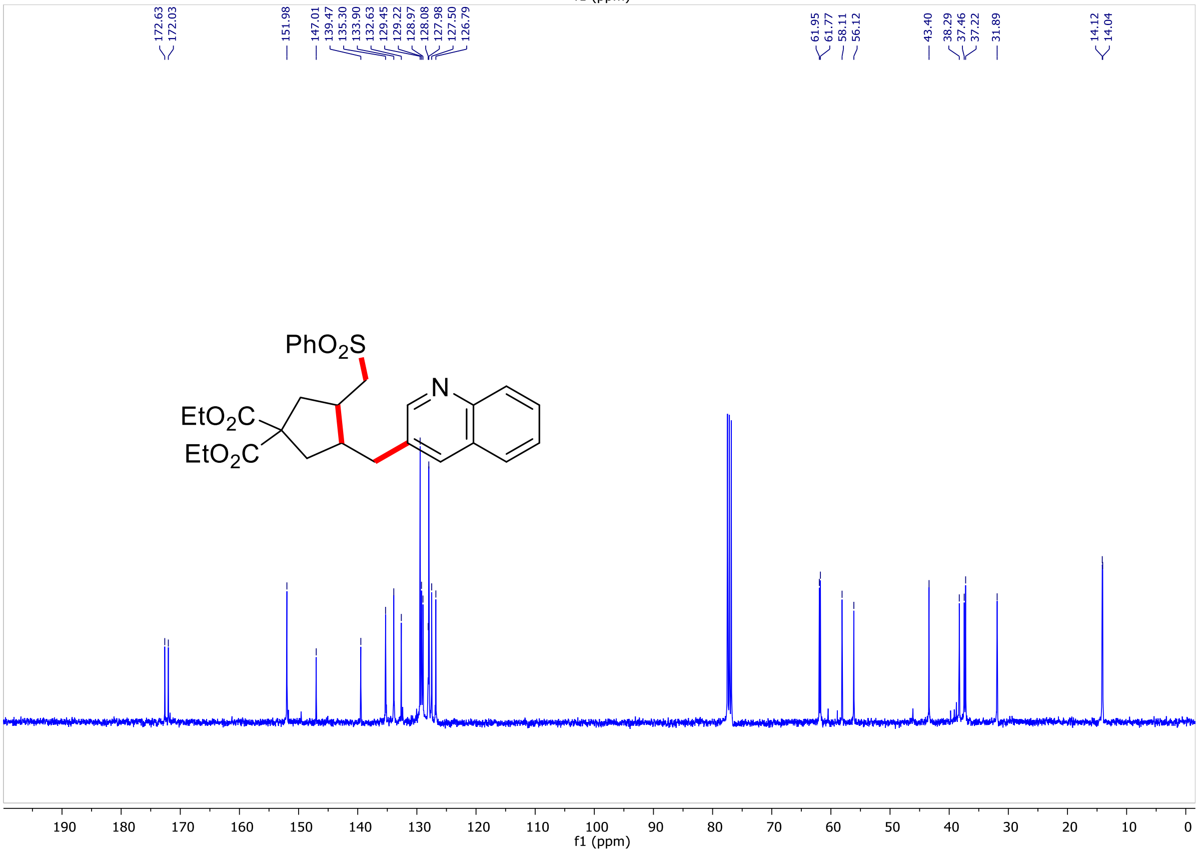
PhO2S
EtO2C
EtO2C
N
f1 (ppm)

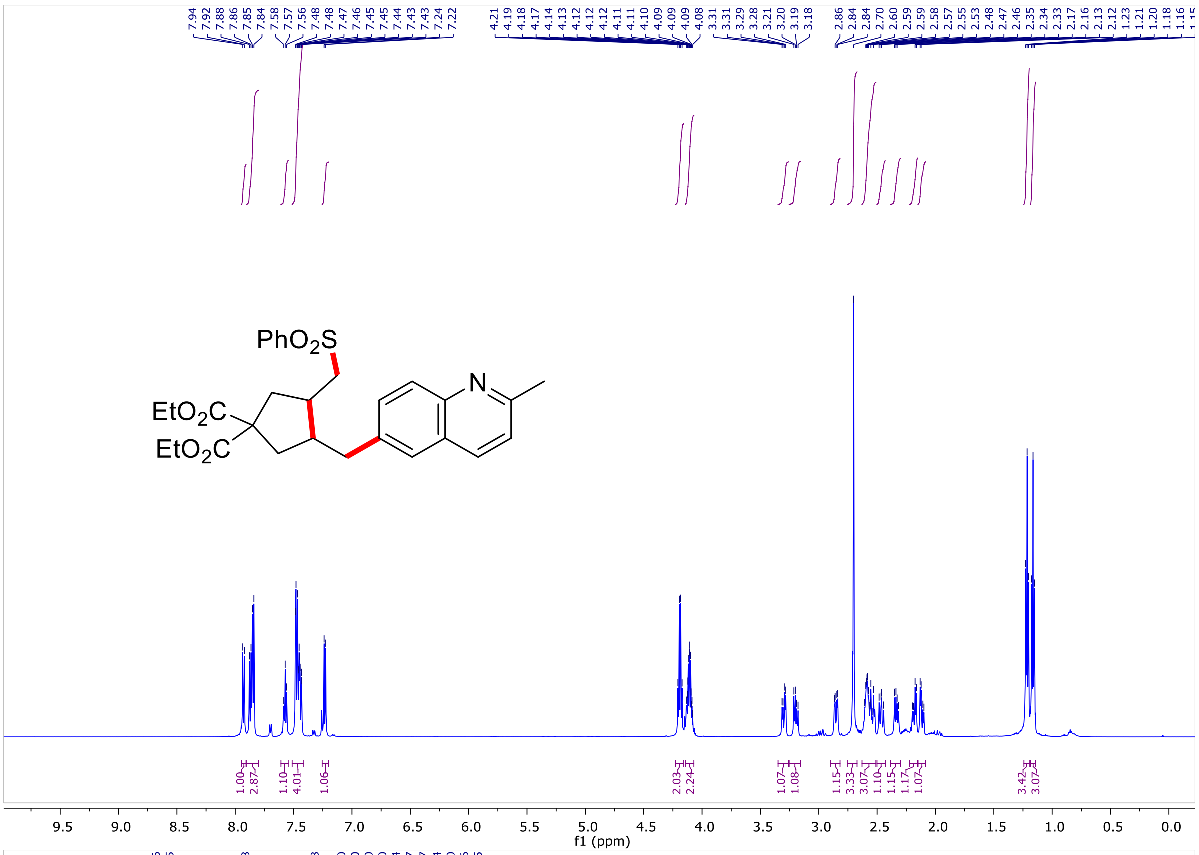
7.94
7.92
7.88
7.86
7.85
7.84
7.58
7.57
7.56
7.48
7.48
7.47
7.46
7.45
7.45
7.44
7.43
7.43
7.24
7.22
4.21
4.19
4.18
4.17
4.14
4.13
4.12
4.12
4.12
4.11
4.11
4.10
4.09
4.09
4.09
4.08
3.31
3.31
3.29
3.28
3.21
3.20
3.19
3.18
2.86
2.84
2.84
2.70
2.60
2.59
2.59
2.58
2.57
2.55
2.53
2.48
2.47
2.46
2.35
2.34
2.33
2.17
2.16
2.13
2.12
1.23
1.21
1.20
1.18
1.16
PhO2S
N
EtO2C
EtO2C
1.00
2.87
1.10
4.01
1.06
2.03
2.24
1.07
1.08
1.15
3.33
3.07
1.10
1.15
1.17
1.07
3.42
3.07
9.5
9.0
8.5
8.0
7.5
7.0
6.5
6.0
5.5
5.0
4.5
4.0
3.5
3.0
2.5
2.0
1.5
1.0
0.5
0.0
f1 (ppm)

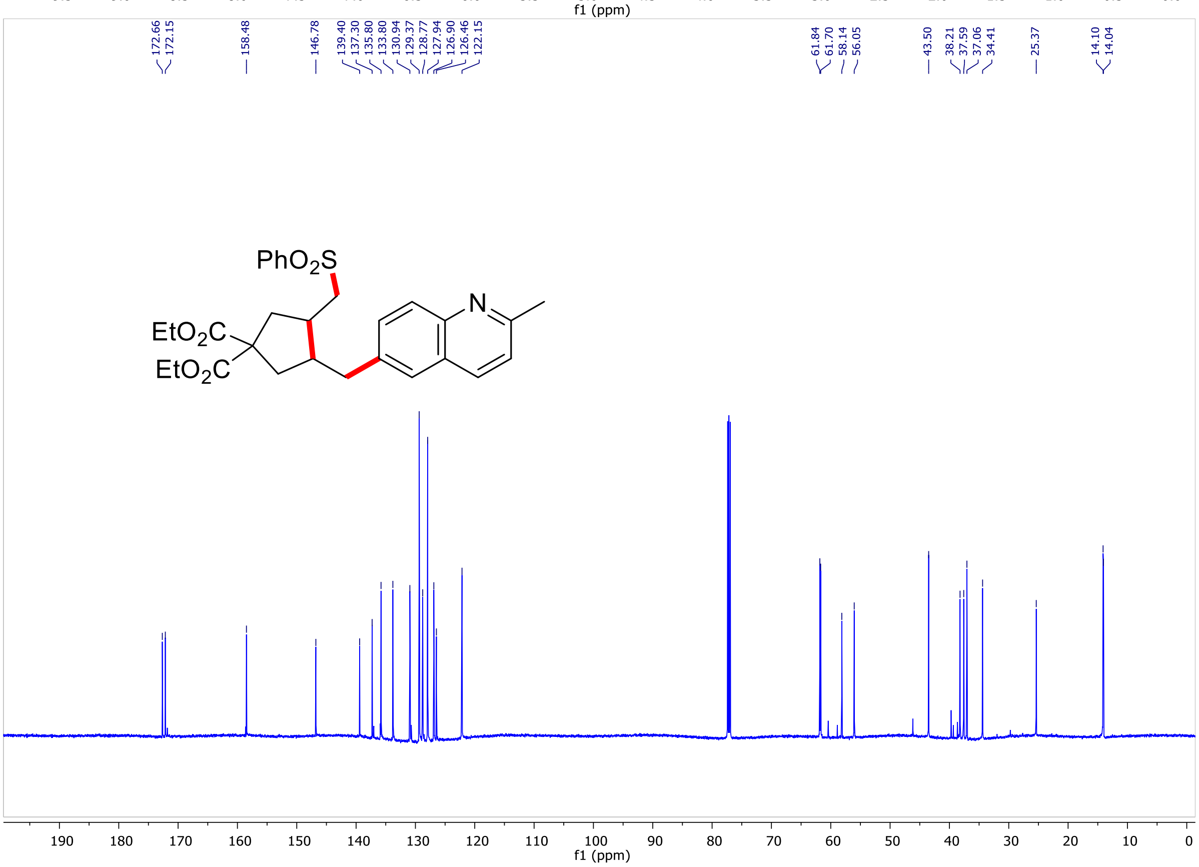
172.66
172.15
158.48
146.78
139.40
137.30
135.80
133.80
130.94
129.37
128.77
127.94
126.90
126.46
122.15
61.84
61.70
58.14
56.05
43.50
38.21
37.59
37.06
34.41
25.37
14.10
14.04
PhO2S
N
EtO2C
EtO2C
190
180
170
160
150
140
130
120
110
100
90
80
70
60
50
40
30
20
10
0
f1 (ppm)

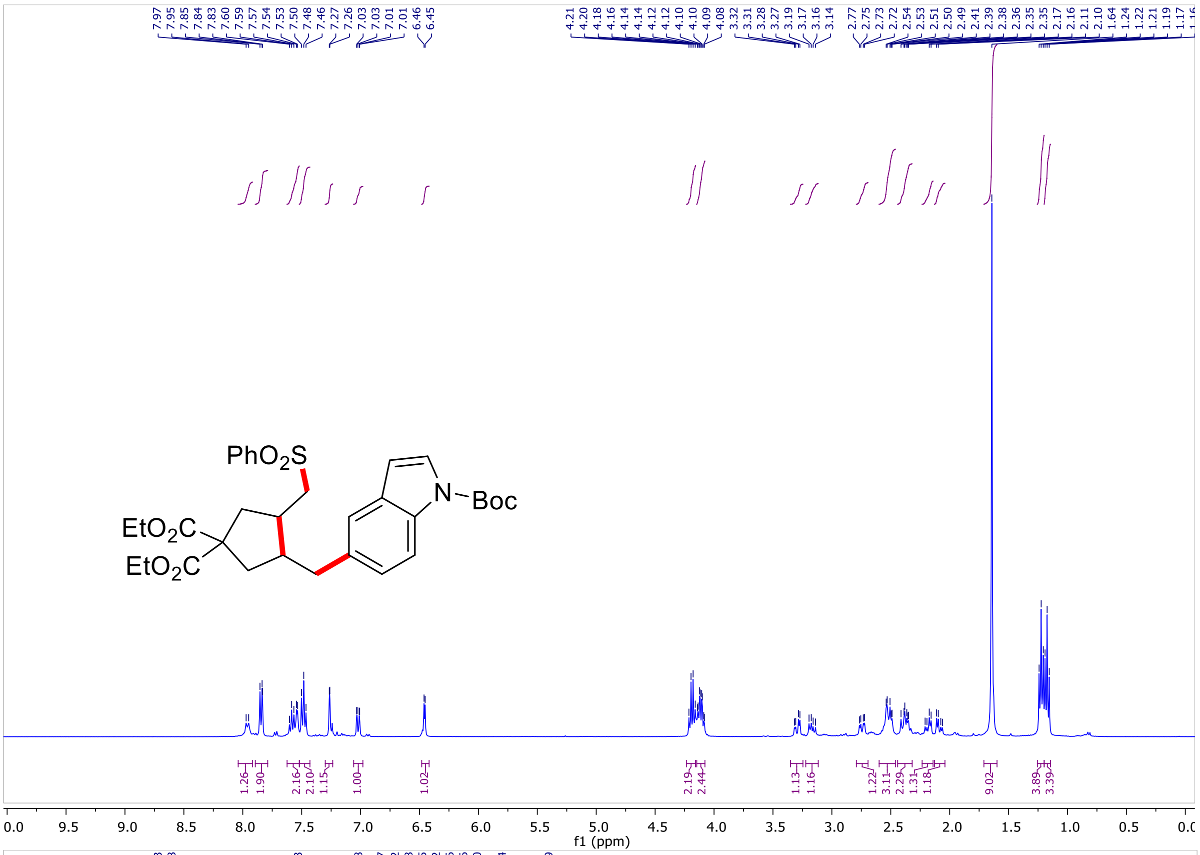
7.97 7.95 7.85 7.84 7.83 7.60 7.59 7.57 7.54 7.53 7.50 7.48 7.46 7.27 7.26 7.03 7.03 7.01 7.01 6.46 6.45
4.21 4.20 4.18 4.16 4.14 4.14 4.12 4.12 4.10 4.10 4.09 4.08 3.32 3.31 3.28 3.27 3.19 3.17 3.16 3.14
2.77 2.75 2.73 2.72 2.54 2.53 2.51 2.50 2.49 2.41 2.39 2.38 2.36 2.35 2.35 2.17 2.16 2.11 2.10 1.64 1.24 1.22 1.21 1.19 1.17
PhO2S
EtO2C
EtO2C
N
Boc
1.26 1.90 2.16 2.10 1.15 1.00 1.02 2.19 2.44 1.13 1.16 1.22 3.11 2.29 1.31 1.18 9.02 3.89 3.39
f1 (ppm)

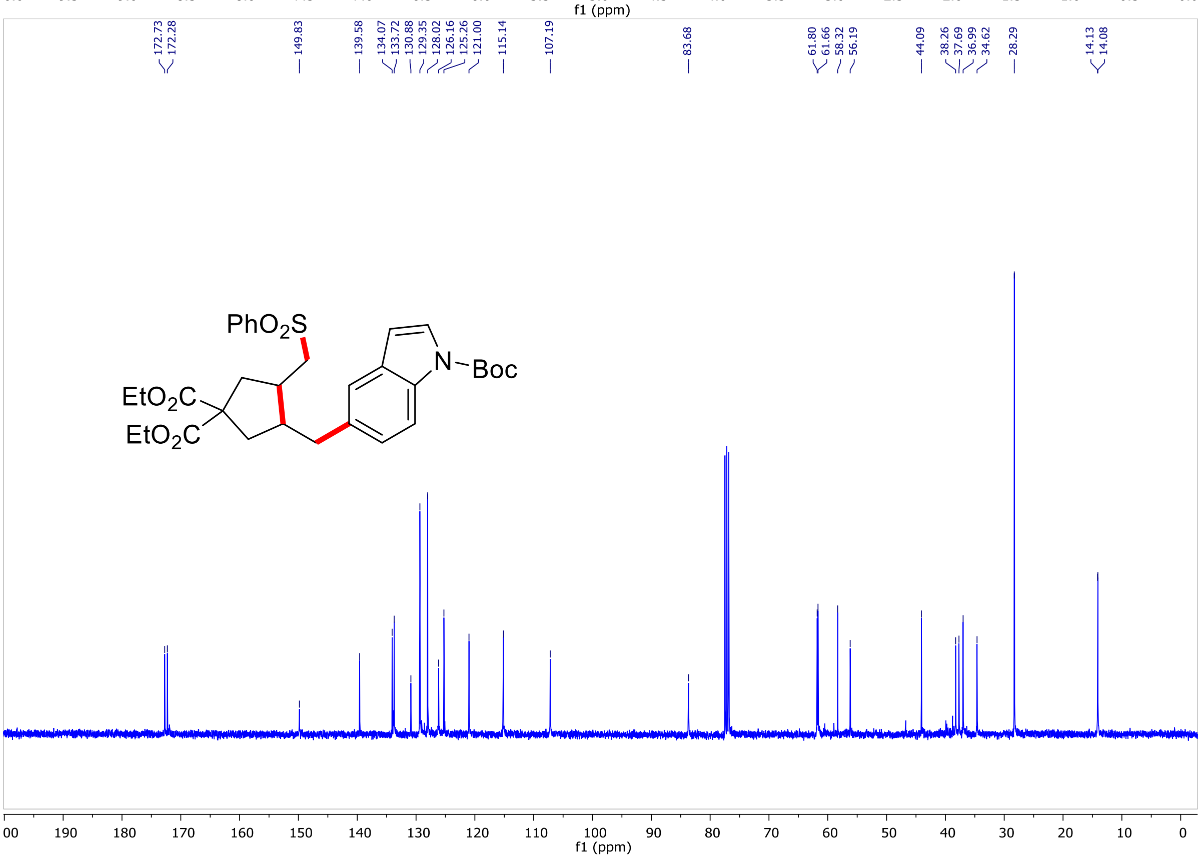
172.73 172.28 149.83 139.58 134.07 133.72 130.88 129.35 128.02 126.16 125.26 121.00 115.14 107.19 83.68 61.80 61.66 58.32 56.19 44.09 38.26 37.69 36.99 34.62 28.29 14.13 14.08
PhO2S
EtO2C
EtO2C
N
Boc
f1 (ppm)

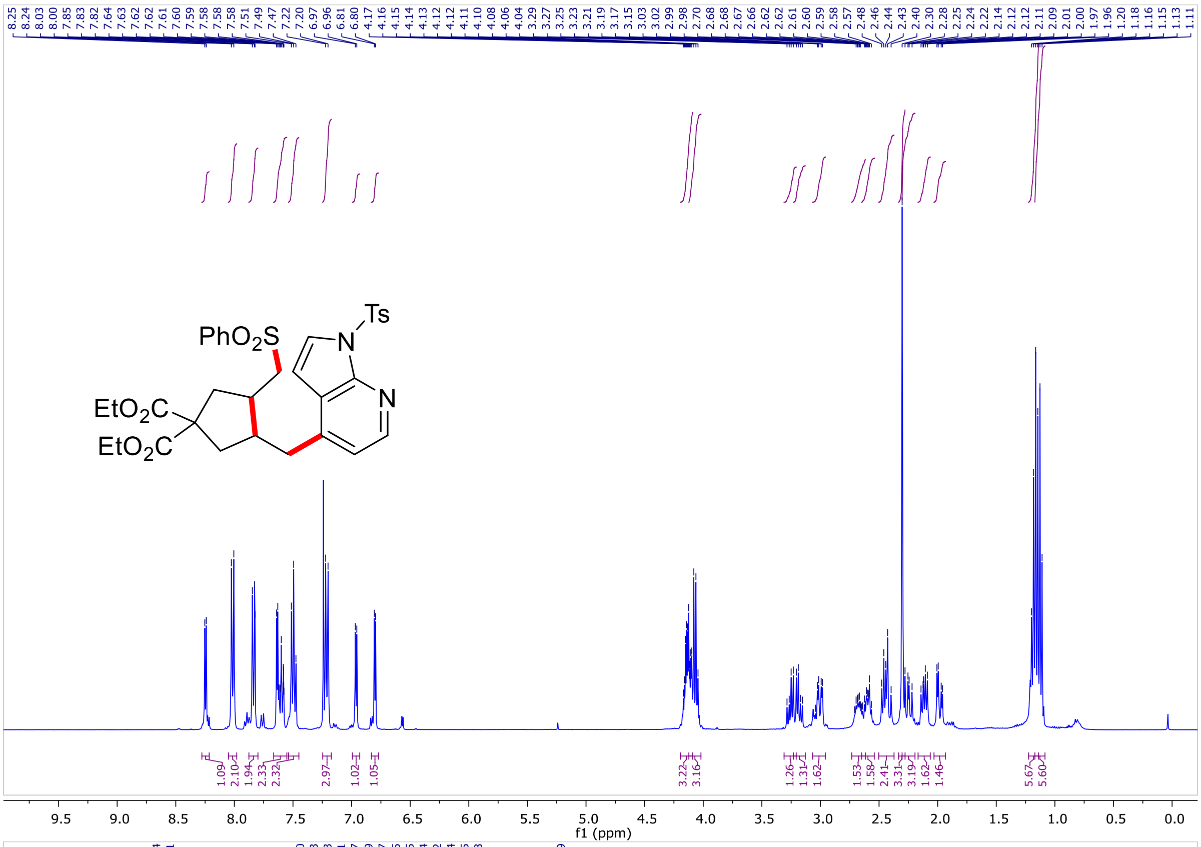

8.25 8.24 8.03 8.00 7.85 7.83 7.82 7.64 7.63 7.62 7.62 7.61 7.60 7.59 7.58 7.58 7.58 7.51 7.49 7.47 7.22 7.20 6.97 6.96 6.81 6.80 4.17 4.16 4.15 4.14 4.13 4.12 4.12 4.11 4.10 4.08 4.06 4.04 3.29 3.27 3.25 3.23 3.21 3.19 3.17 3.15 3.03 3.02 2.99 2.98 2.70 2.68 2.68 2.67 2.66 2.62 2.62 2.61 2.60 2.59 2.58 2.57 2.48 2.46 2.44 2.43 2.40 2.30 2.28 2.25 2.24 2.22 2.14 2.12 2.12 2.11 2.09 2.01 2.00 1.97 1.96 1.20 1.18 1.16 1.15 1.13 1.11
PhO2S
Ts
N
N
EtO2C
EtO2C
1.09 2.10 1.94 2.33 2.32 2.97 1.02 1.05 3.22 3.16 1.26 1.31 1.62 1.53 1.58 2.41 3.31 3.19 1.62 1.46 5.67 5.60
9.5 9.0 8.5 8.0 7.5 7.0 6.5 6.0 5.5 5.0 4.5 4.0 3.5 3.0 2.5 2.0 1.5 1.0 0.5 0.0
f1 (ppm)

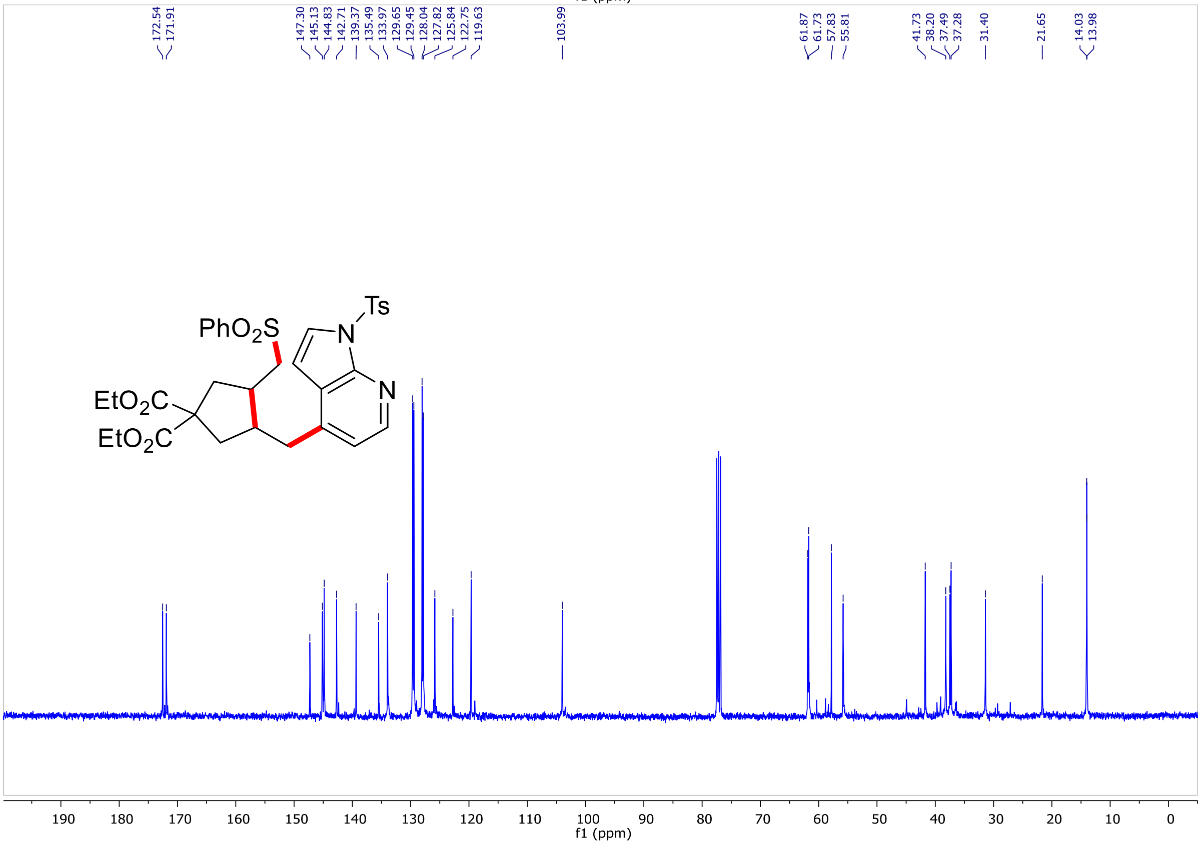

172.54 171.91 147.30 145.13 144.83 142.71 139.37 135.49 133.97 129.65 129.45 128.04 127.82 125.84 122.75 119.63 103.99 61.87 61.73 57.83 55.81 41.73 38.20 37.49 37.28 31.40 21.65 14.03 13.98
PhO2S
Ts
N
N
EtO2C
EtO2C
190 180 170 160 150 140 130 120 110 100 90 80 70 60 50 40 30 20 10 0
f1 (ppm)

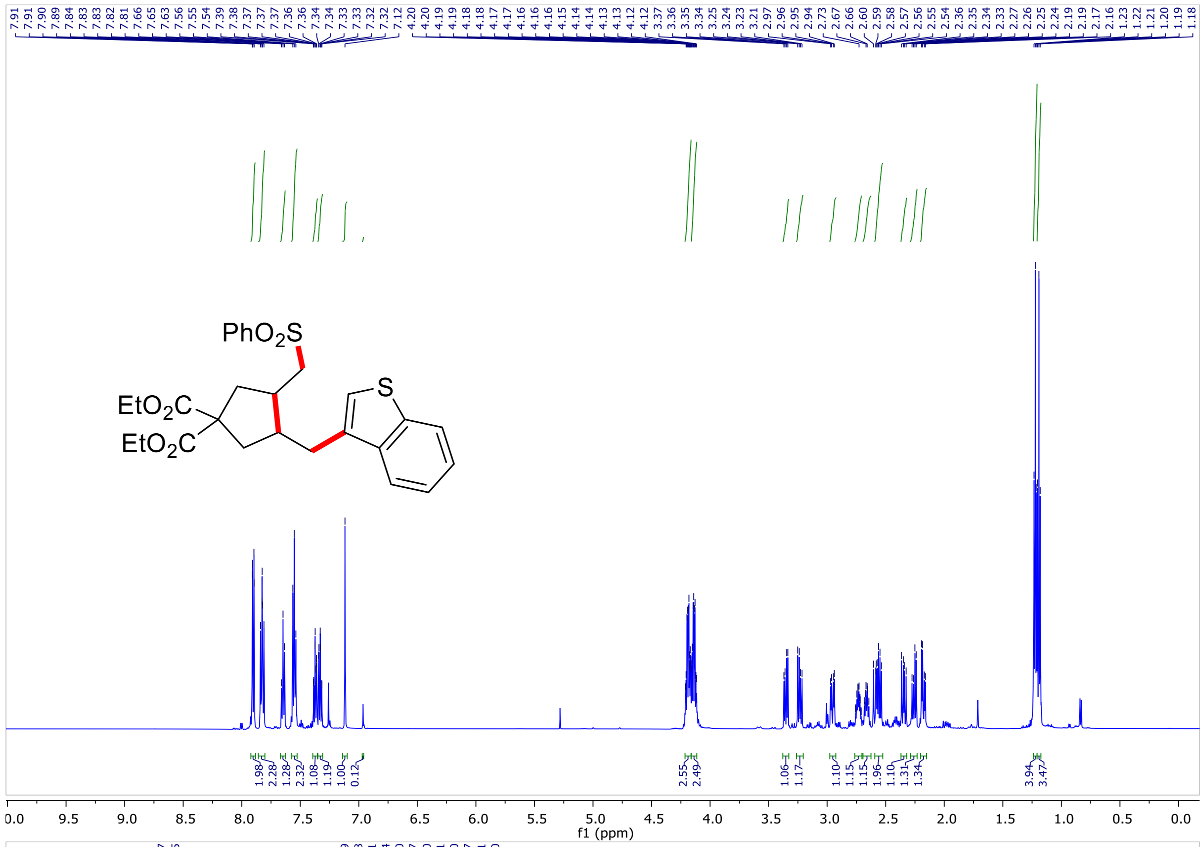
PhO2S
EtO2C
EtO2C
S
f1 (ppm)

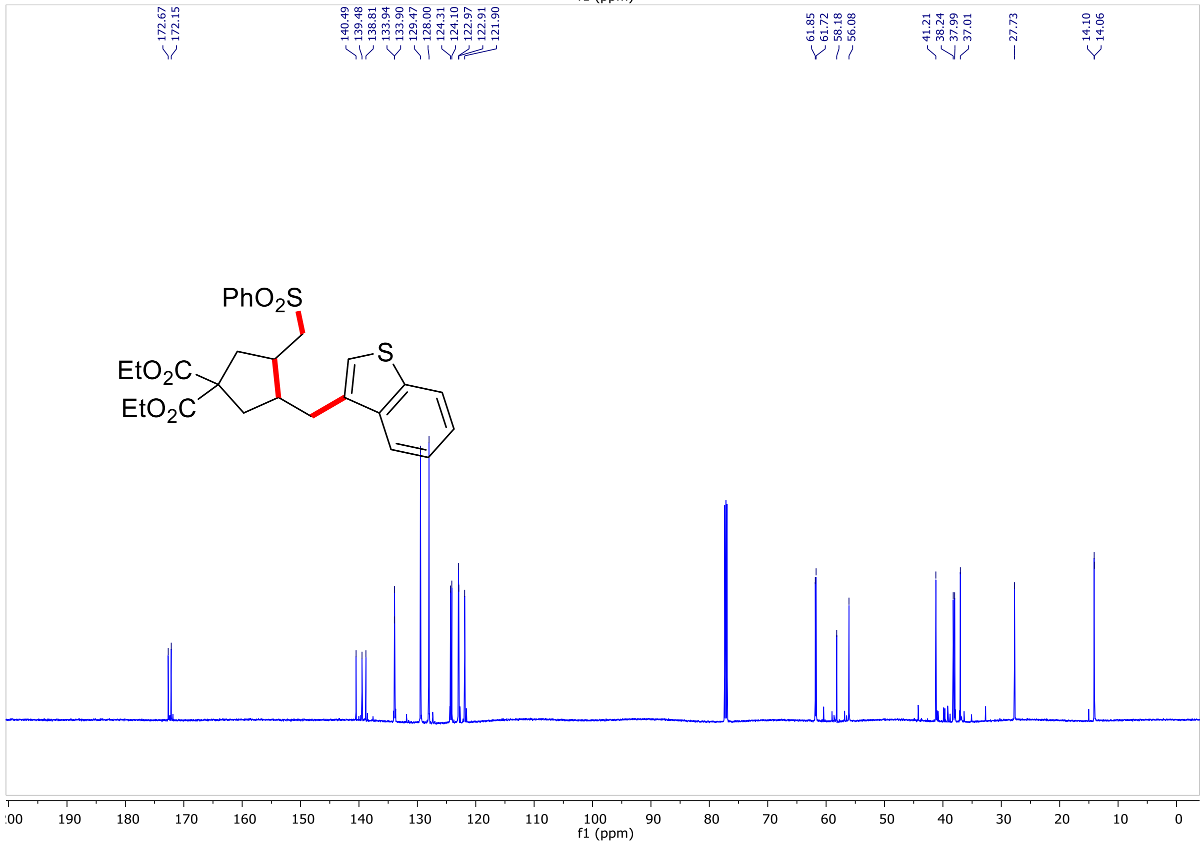
PhO2S
EtO2C
EtO2C
S
f1 (ppm)

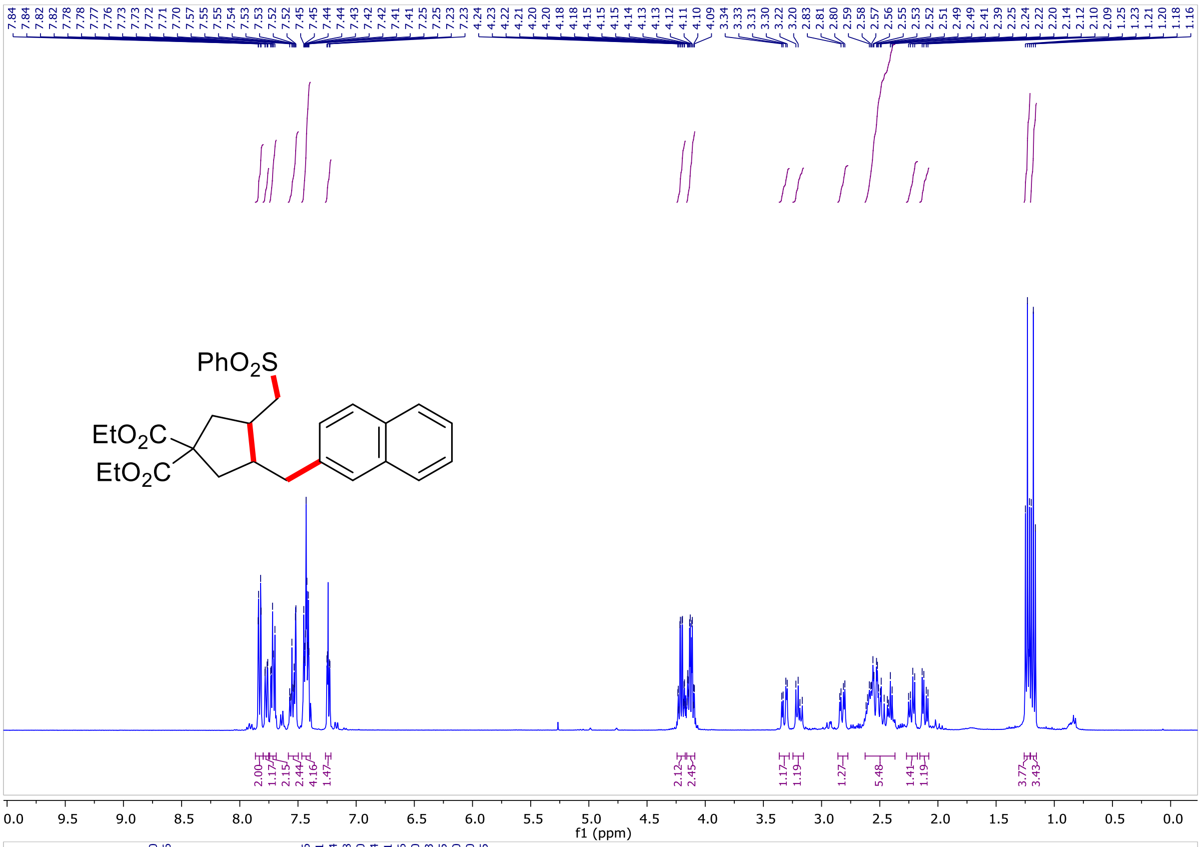
PhO2S
EtO2C
EtO2C
f1 (ppm)

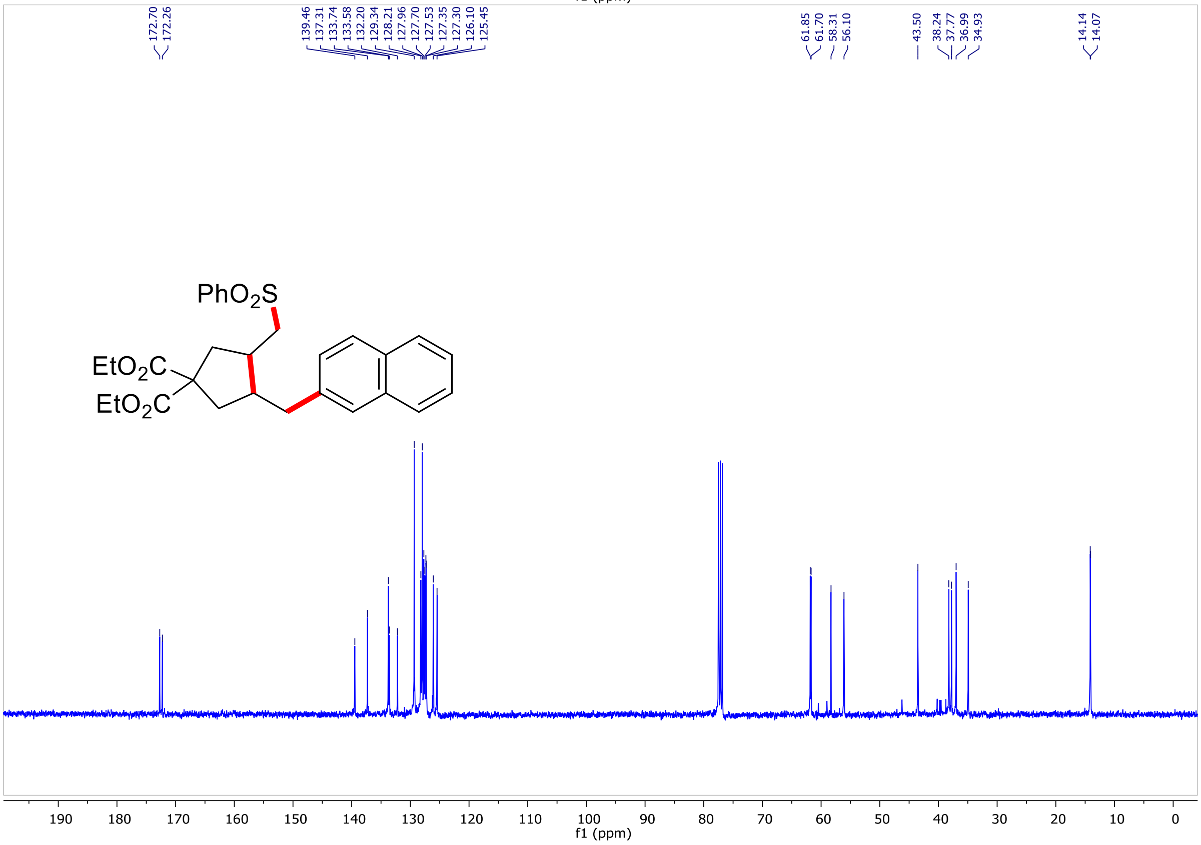
PhO2S
EtO2C
EtO2C
f1 (ppm)

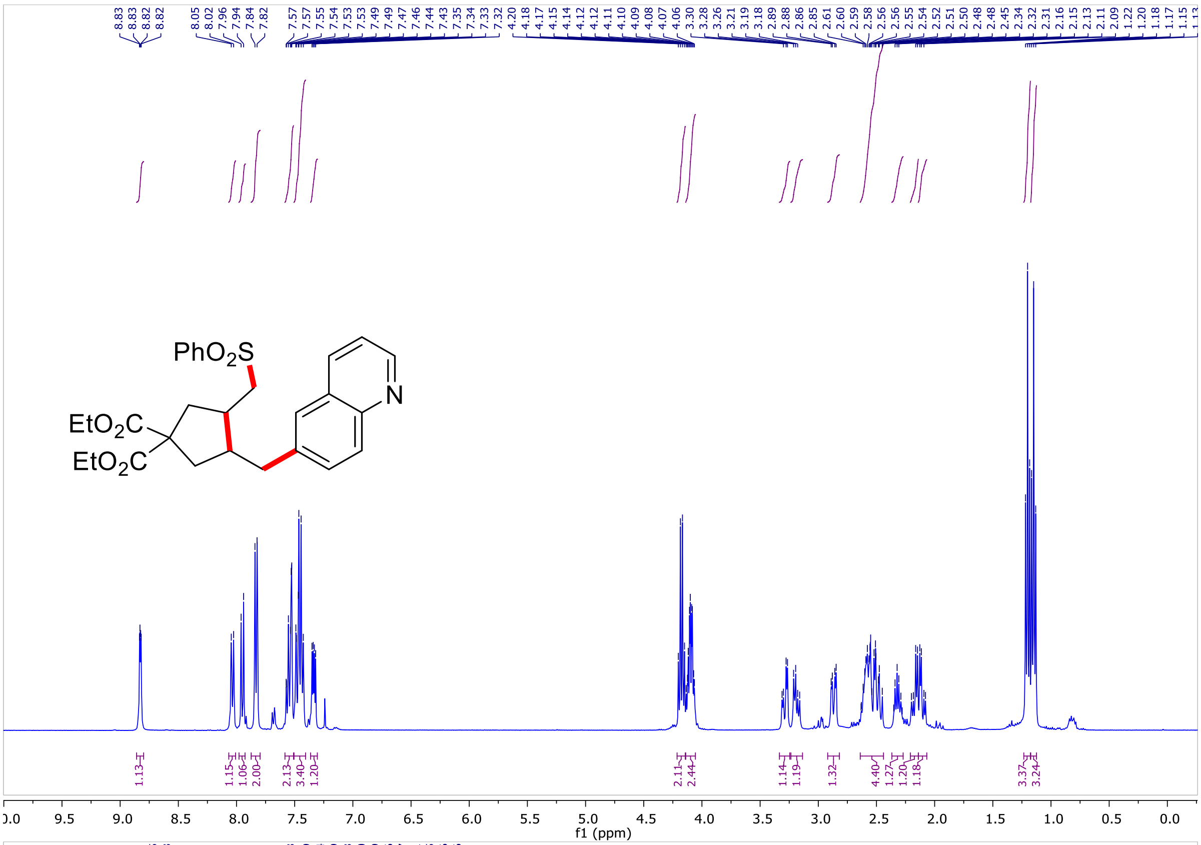
8.83 8.83 8.82 8.82 8.05 8.02 7.96 7.94 7.84 7.82 7.57 7.57 7.55 7.54 7.53 7.53 7.49 7.49 7.47 7.46 7.44 7.43 7.35 7.34 7.33 7.32 4.20 4.18 4.17 4.15 4.14 4.12 4.11 4.10 4.09 4.08 4.07 4.06 3.30 3.28 3.26 3.21 3.19 3.18 2.89 2.88 2.86 2.85 2.61 2.60 2.59 2.58 2.56 2.56 2.55 2.54 2.52 2.51 2.50 2.48 2.45 2.34 2.32 2.31 2.16 2.15 2.13 2.11 2.09 1.22 1.20 1.18 1.17 1.15 1.13
PhO2S
EtO2C
EtO2C
N
1.13 1.15 1.06 2.00 2.13 3.40 1.20 2.11 2.44 1.14 1.19 1.32 4.40 1.27 1.20 1.18 3.37 3.24
f1 (ppm)

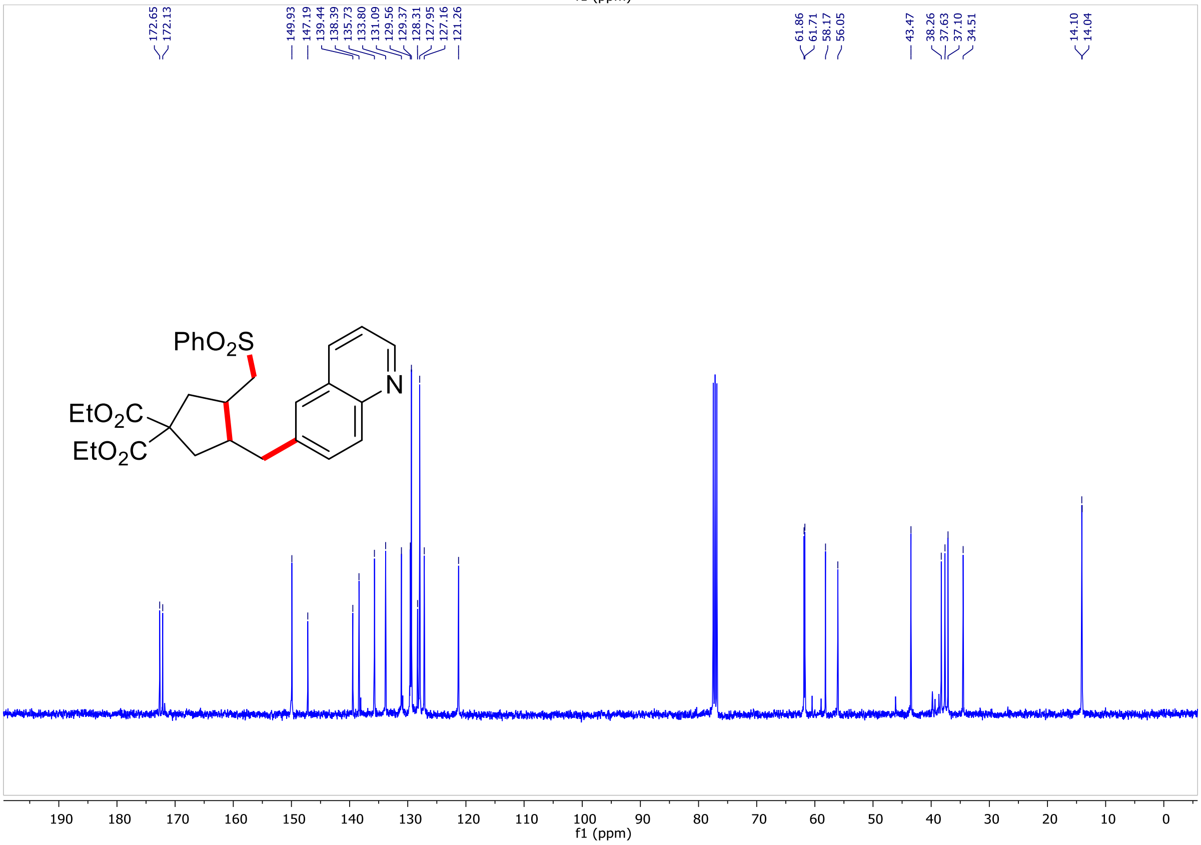
172.65 172.13 149.93 147.19 139.44 138.39 135.73 133.80 131.09 129.56 129.37 128.31 127.95 127.16 121.26 61.86 61.71 58.17 56.05 43.47 38.26 37.63 37.10 34.51 14.10 14.04
PhO2S
EtO2C
EtO2C
N
f1 (ppm)

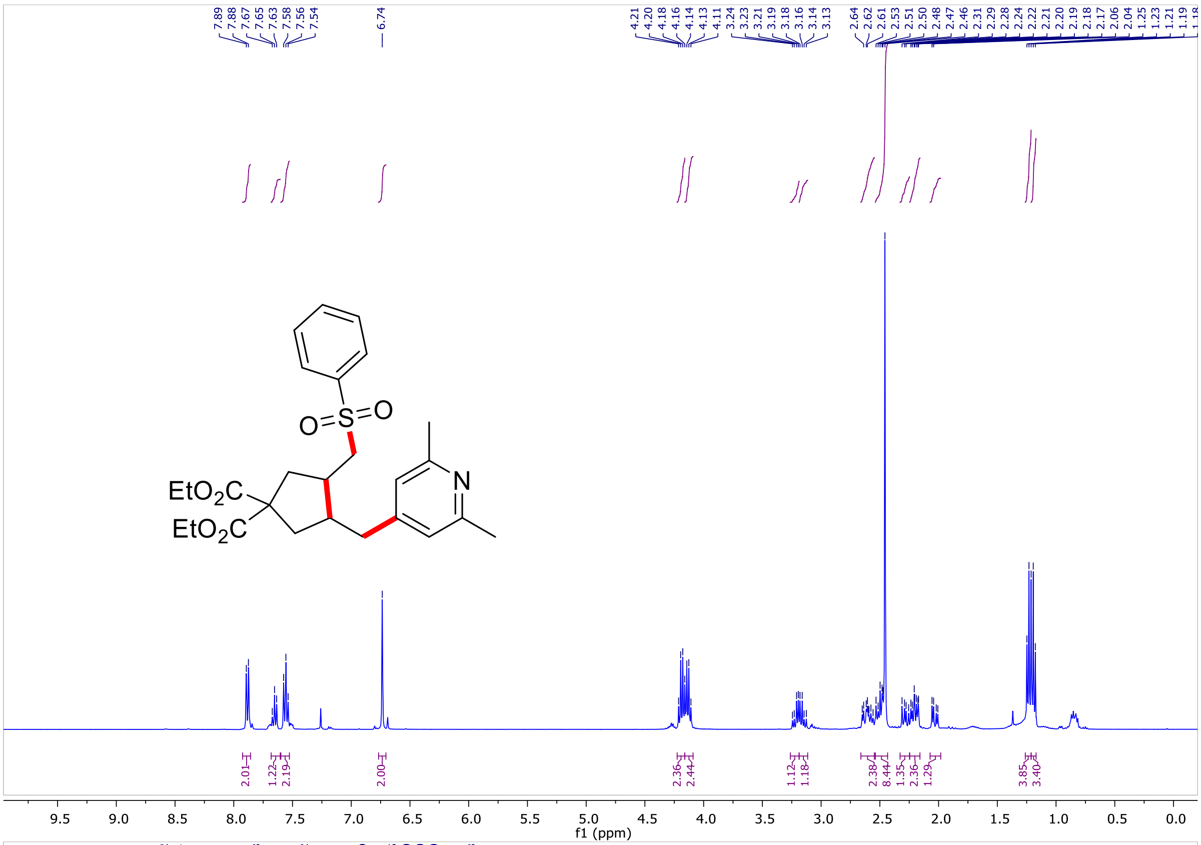
EtO2C
EtO2C
O=S=O
N
f1 (ppm)

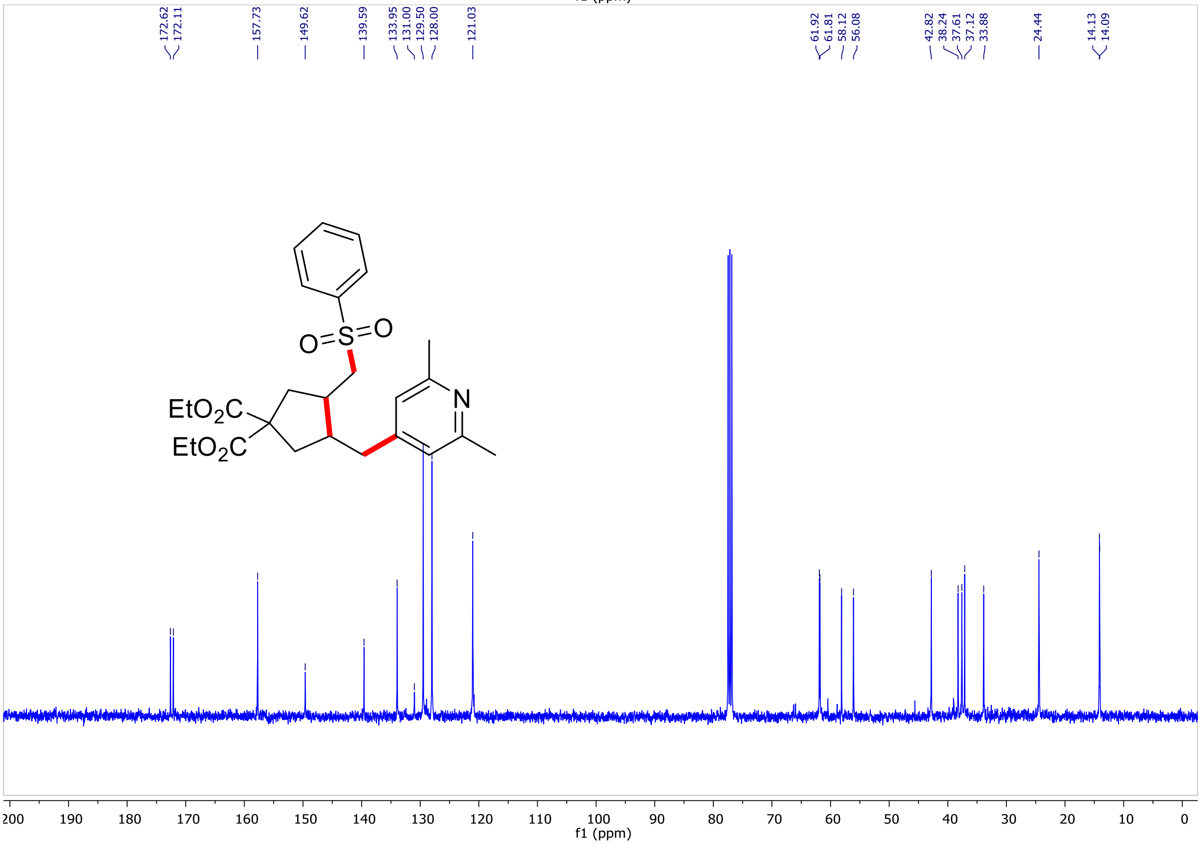
EtO2C
EtO2C
O=S=O
N
f1 (ppm)

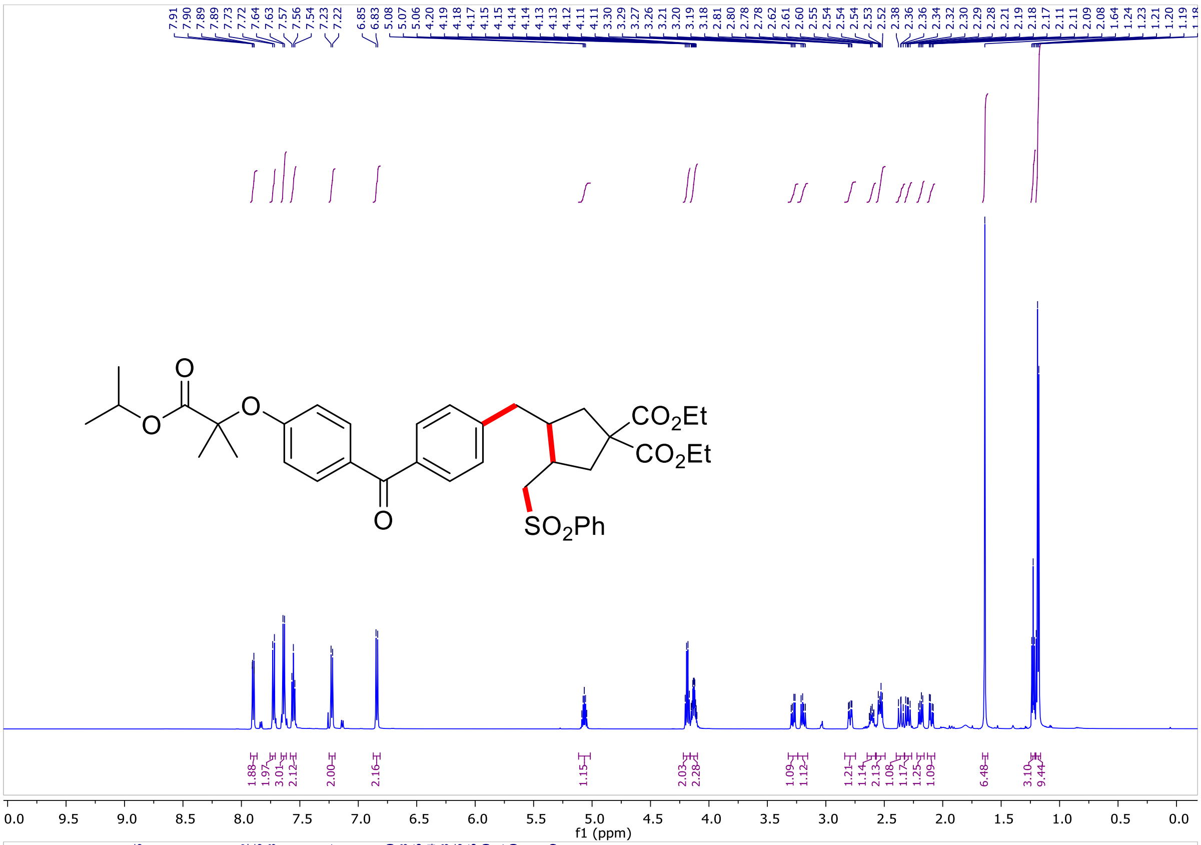
7.91 7.90 7.89 7.89 7.73 7.72 7.64 7.63 7.57 7.56 7.54 7.23 7.22 6.85 6.83 5.08 5.07 5.06 4.20 4.19 4.18 4.17 4.15 4.15 4.14 4.14 4.13 4.13 4.12 4.11 4.11 3.30 3.29 3.27 3.26 3.21 3.20 3.19 3.18 2.81 2.80 2.78 2.78 2.62 2.61 2.60 2.55 2.54 2.54 2.54 2.53 2.52 2.38 2.36 2.36 2.34 2.32 2.30 2.29 2.28 2.21 2.19 2.18 2.17 2.11 2.11 2.09 2.08 1.64 1.24 1.23 1.21 1.20 1.19
O
O
O
O
CO_2Et
CO_2Et
SO_2Ph
1.88 1.97 3.01 2.12 2.00 2.16 1.15 2.03 2.28 1.09 1.12 1.21 1.14 2.13 1.08 1.17 1.25 1.09 6.48 3.10 9.44
0.0 9.5 9.0 8.5 8.0 7.5 7.0 6.5 6.0 5.5 5.0 4.5 4.0 3.5 3.0 2.5 2.0 1.5 1.0 0.5 0.0
f1 (ppm)

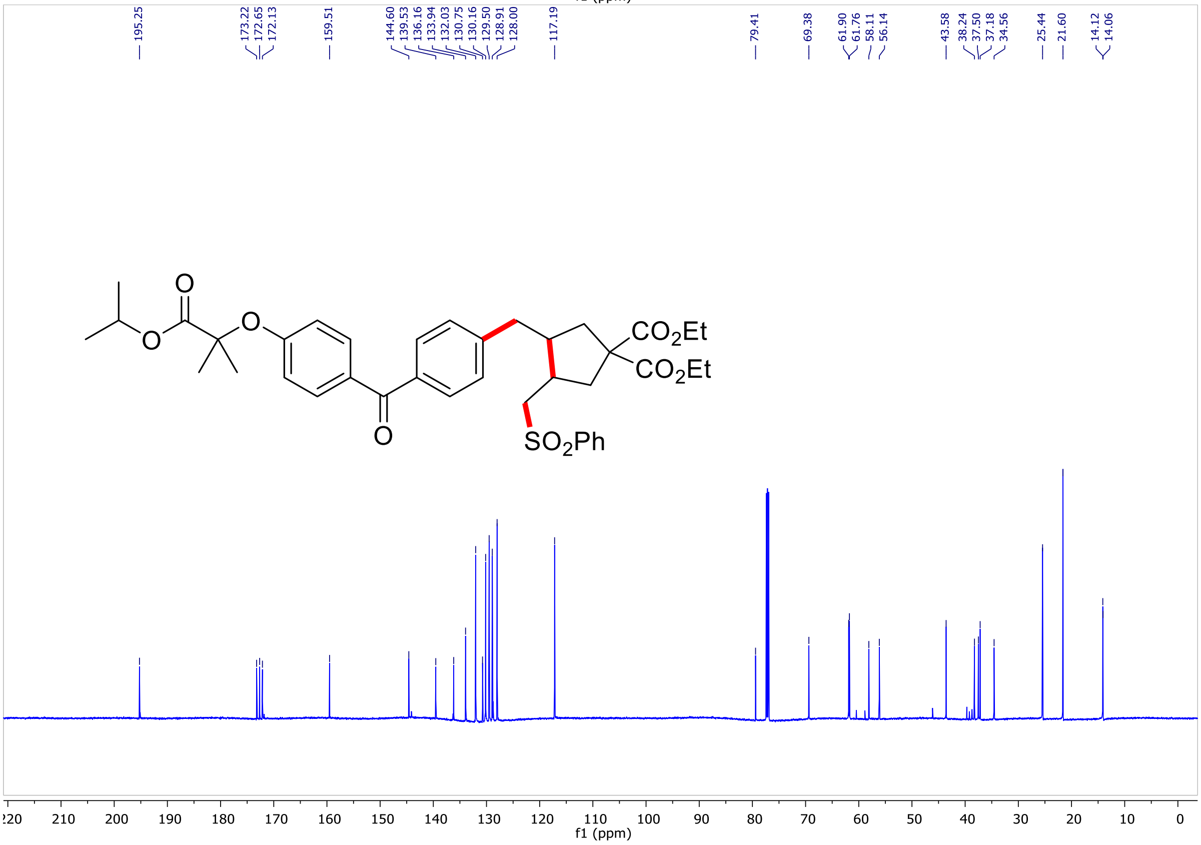
195.25 173.22 172.65 172.13 159.51 144.60 139.53 136.16 133.94 132.03 130.75 130.16 129.50 128.91 128.00 117.19 79.41 69.38 61.90 61.76 58.11 56.14 43.58 38.24 37.50 37.18 34.56 25.44 21.60 14.12 14.06
O
O
O
O
CO_2Et
CO_2Et
SO_2Ph
210 200 190 180 170 160 150 140 130 120 110 100 90 80 70 60 50 40 30 20 10 0
f1 (ppm)

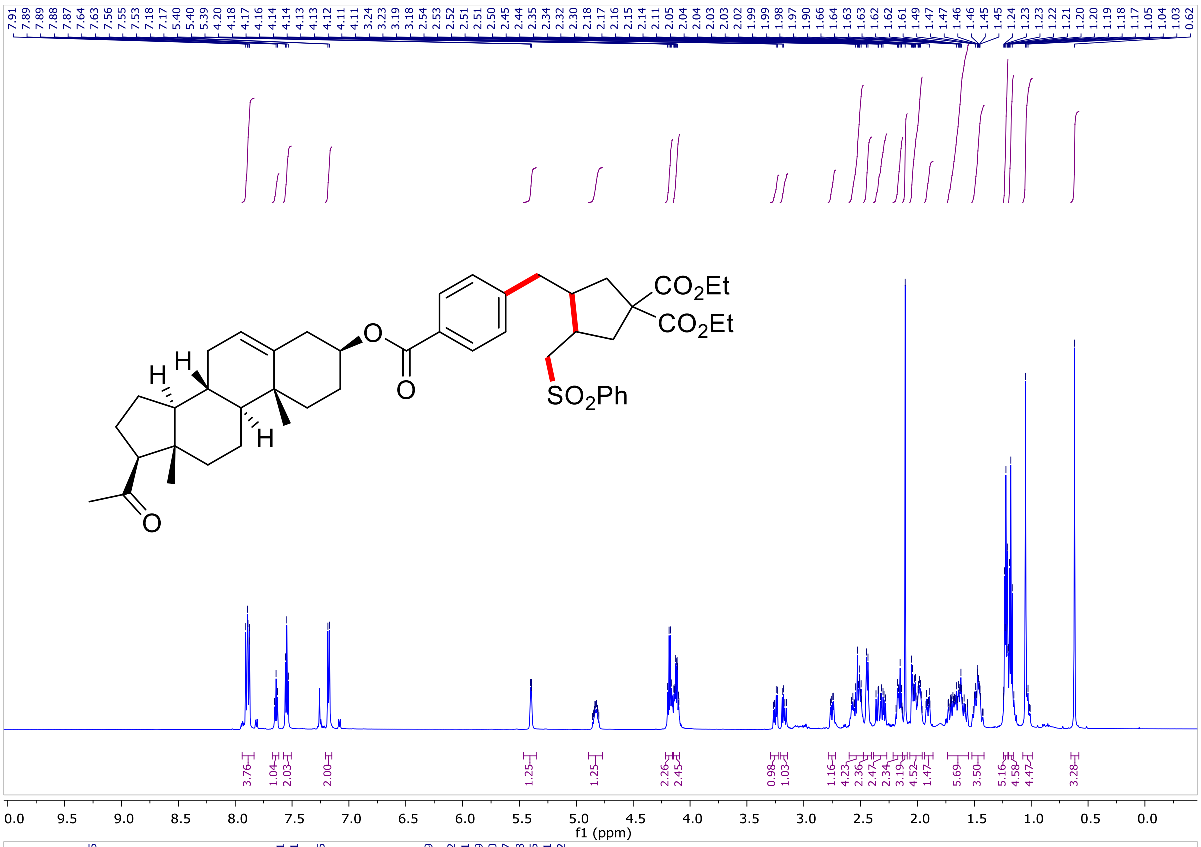

CO2Et
CO2Et
SO2Ph
O
H
f1 (ppm)

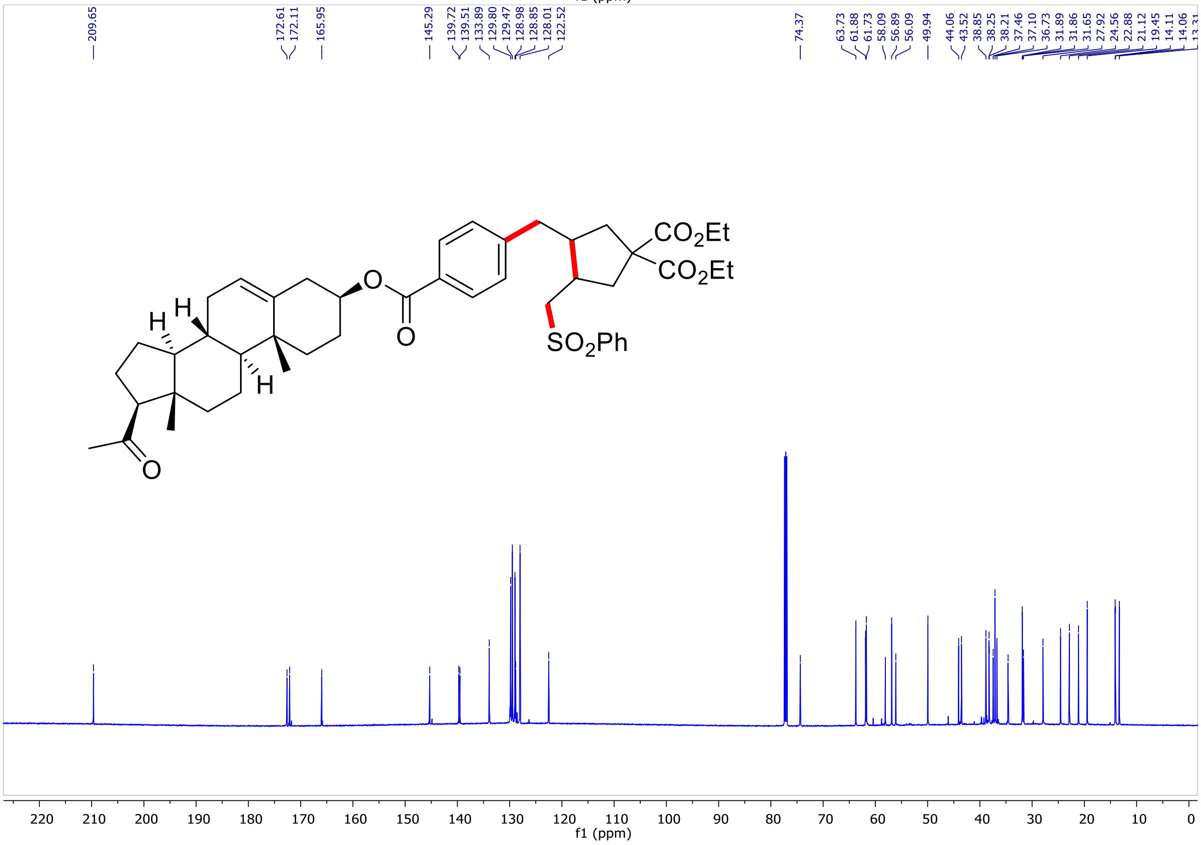

CO2Et
CO2Et
SO2Ph
O
H
f1 (ppm)

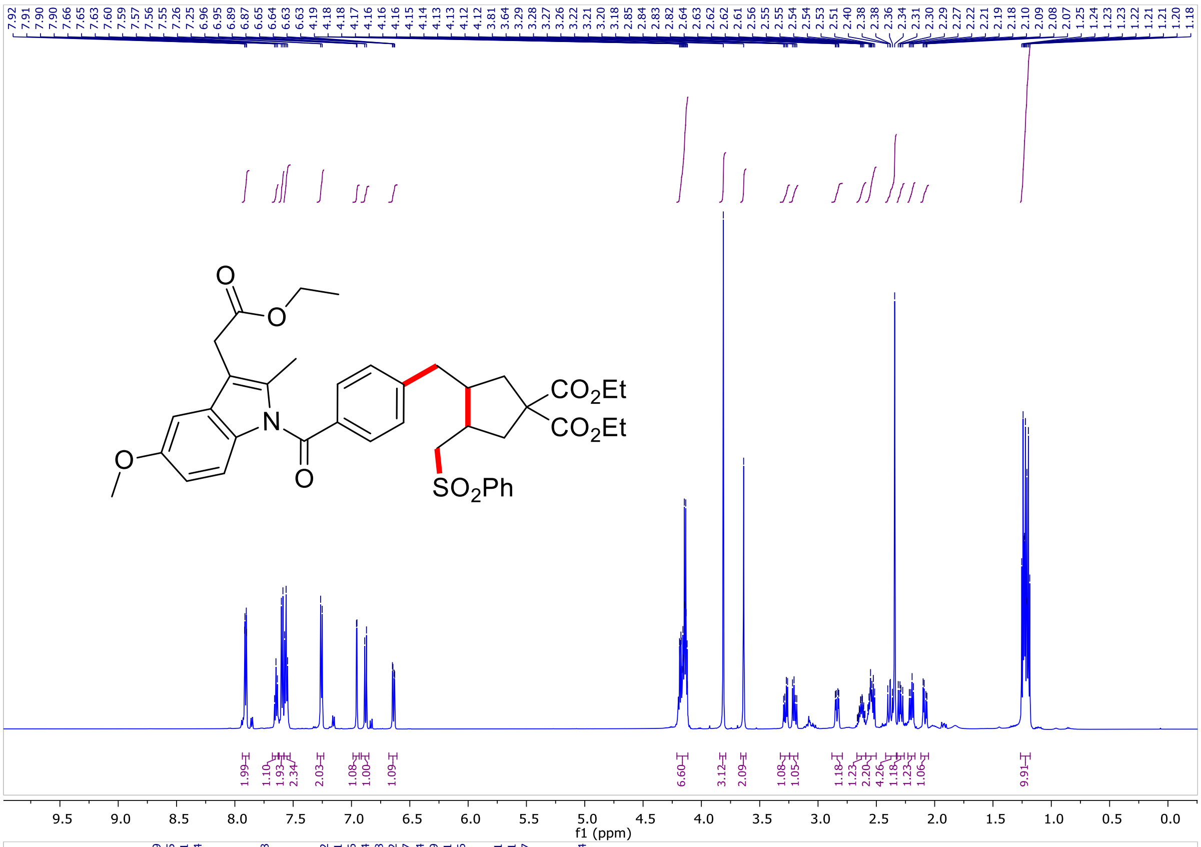
7.92 7.91 7.90 7.66 7.65 7.63 7.60 7.59 7.57 7.56 7.55 7.26 7.25 6.96 6.95 6.89 6.87 6.65 6.64 6.63 6.63 4.19 4.18 4.18 4.17 4.16 4.16 4.15 4.14 4.13 4.13 4.12 4.12 3.81 3.64 3.29 3.28 3.27 3.26 3.22 3.21 3.20 3.18 2.85 2.84 2.83 2.82 2.64 2.63 2.62 2.62 2.61 2.56 2.55 2.55 2.54 2.54 2.53 2.51 2.40 2.38 2.38 2.36 2.34 2.31 2.30 2.29 2.27 2.22 2.21 2.19 2.18 2.10 2.09 2.08 2.07 1.25 1.24 1.23 1.23 1.22 1.21 1.21 1.20 1.18
O
O
N
O
O
CO2Et
CO2Et
SO2Ph
1.99 1.10 1.93 2.34 2.03 1.08 1.00 1.09 6.60 3.12 2.09 1.08 1.05 1.18 1.23 2.20 4.26 1.18 1.23 1.06 9.91
9.5 9.0 8.5 8.0 7.5 7.0 6.5 6.0 5.5 5.0 4.5 4.0 3.5 3.0 2.5 2.0 1.5 1.0 0.5 0.0
f1 (ppm)

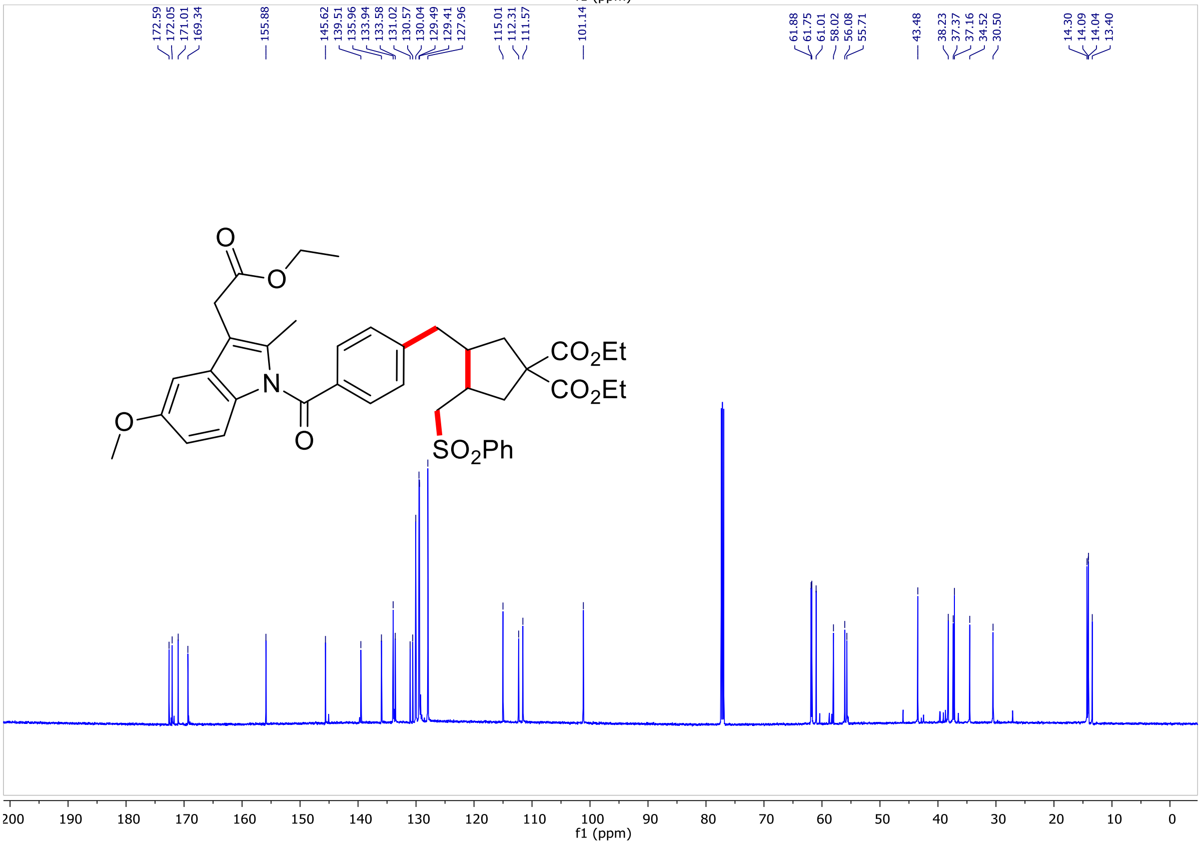
172.59 172.05 171.01 169.34 155.88 145.62 139.51 135.96 133.94 133.58 131.02 130.57 130.04 129.49 129.41 127.96 115.01 112.31 111.57 101.14 61.88 61.75 61.01 58.02 56.08 55.71 43.48 38.23 37.37 37.16 34.52 30.50 14.30 14.09 14.04 13.40
O
O
N
O
O
CO2Et
CO2Et
SO2Ph
200 190 180 170 160 150 140 130 120 110 100 90 80 70 60 50 40 30 20 10 0
f1 (ppm)

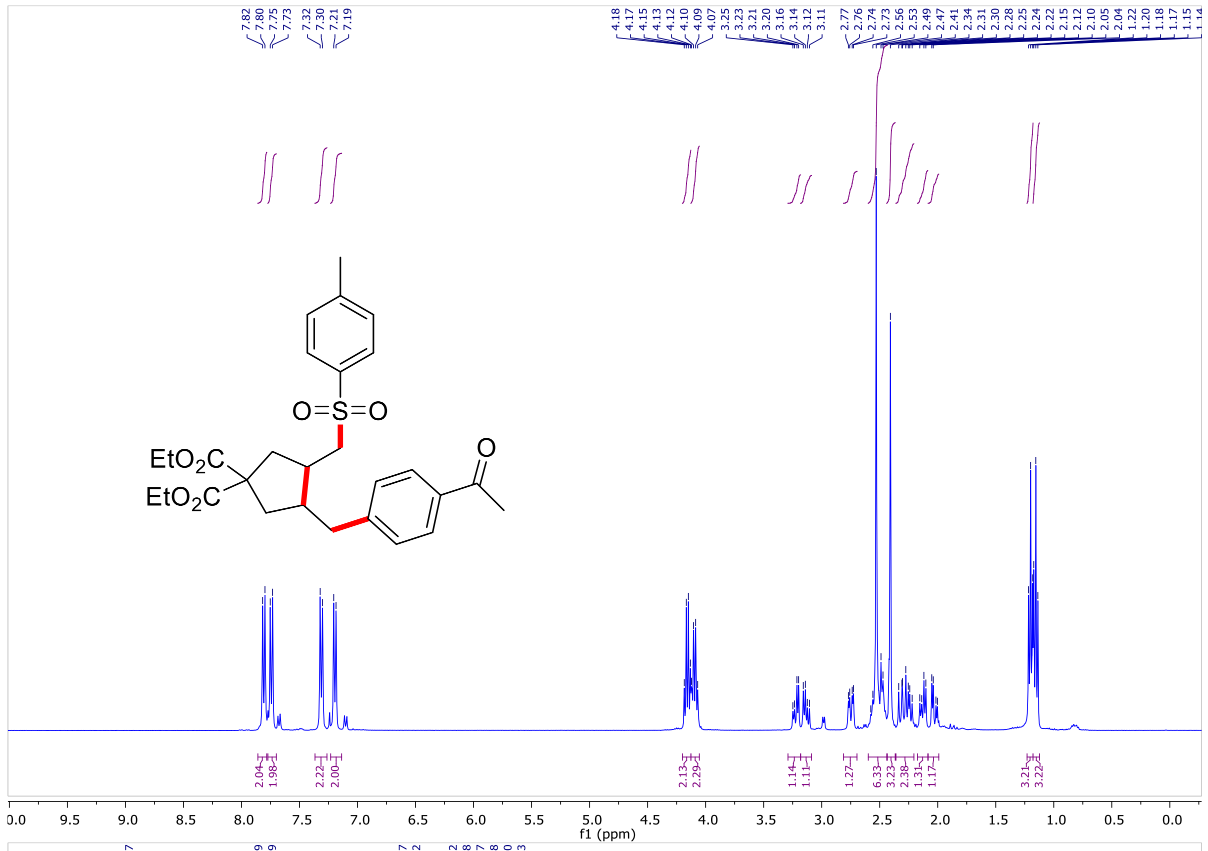
7.82
7.80
7.75
7.73
7.32
7.30
7.21
7.19
4.18
4.17
4.15
4.13
4.12
4.10
4.09
4.07
3.25
3.23
3.21
3.20
3.16
3.14
3.12
3.11
2.77
2.76
2.74
2.73
2.56
2.53
2.49
2.47
2.41
2.34
2.31
2.30
2.28
2.25
2.24
2.22
2.15
2.12
2.10
2.05
2.04
1.22
1.20
1.18
1.17
1.15
2.04
1.98
2.22
2.00
2.13
2.29
1.14
1.11
1.27
6.33
3.23
2.38
1.31
1.17
3.21
3.22
EtO2C
EtO2C
O=S=O
O
f1 (ppm)

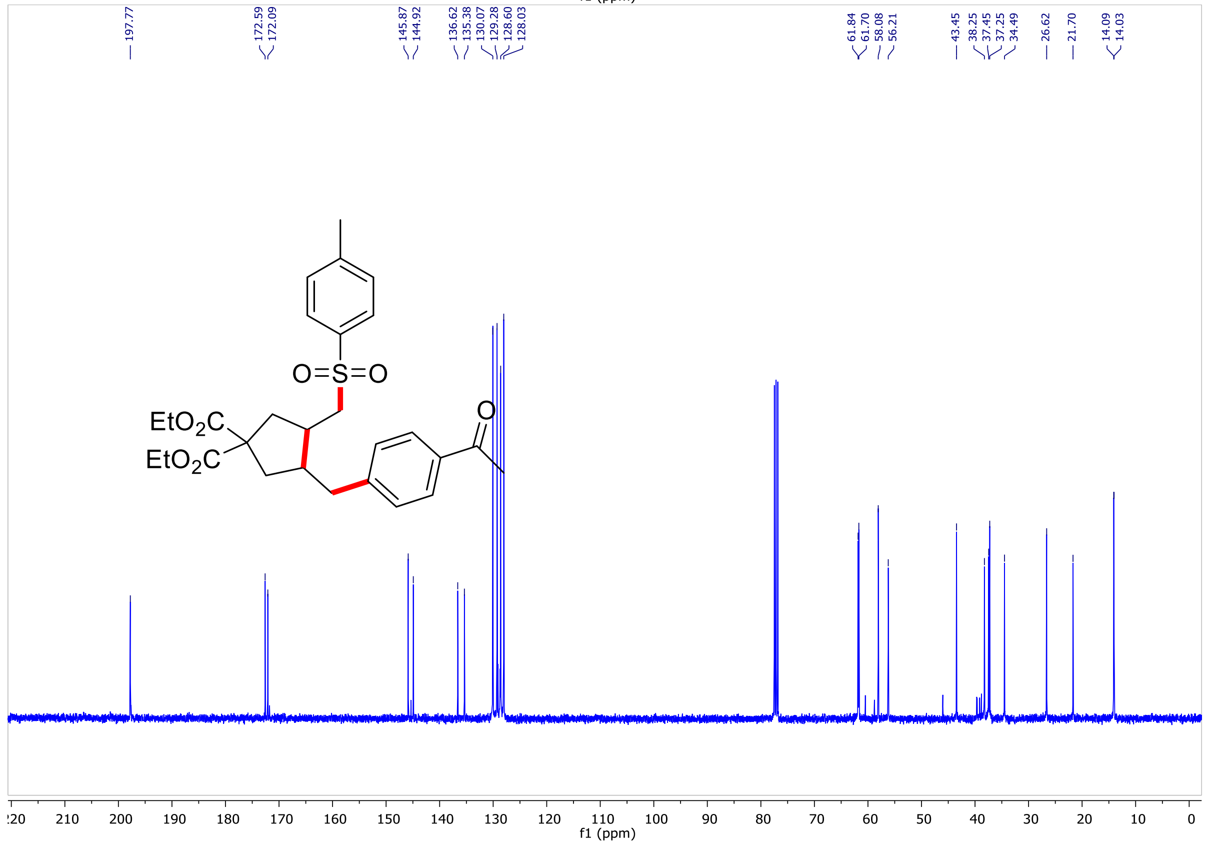
197.77
172.59
172.09
145.87
144.92
136.62
135.38
130.07
129.28
128.60
128.03
61.84
61.70
58.08
56.21
43.45
38.25
37.45
37.25
34.49
26.62
21.70
14.09
14.03
EtO2C
EtO2C
O=S=O
O
f1 (ppm)

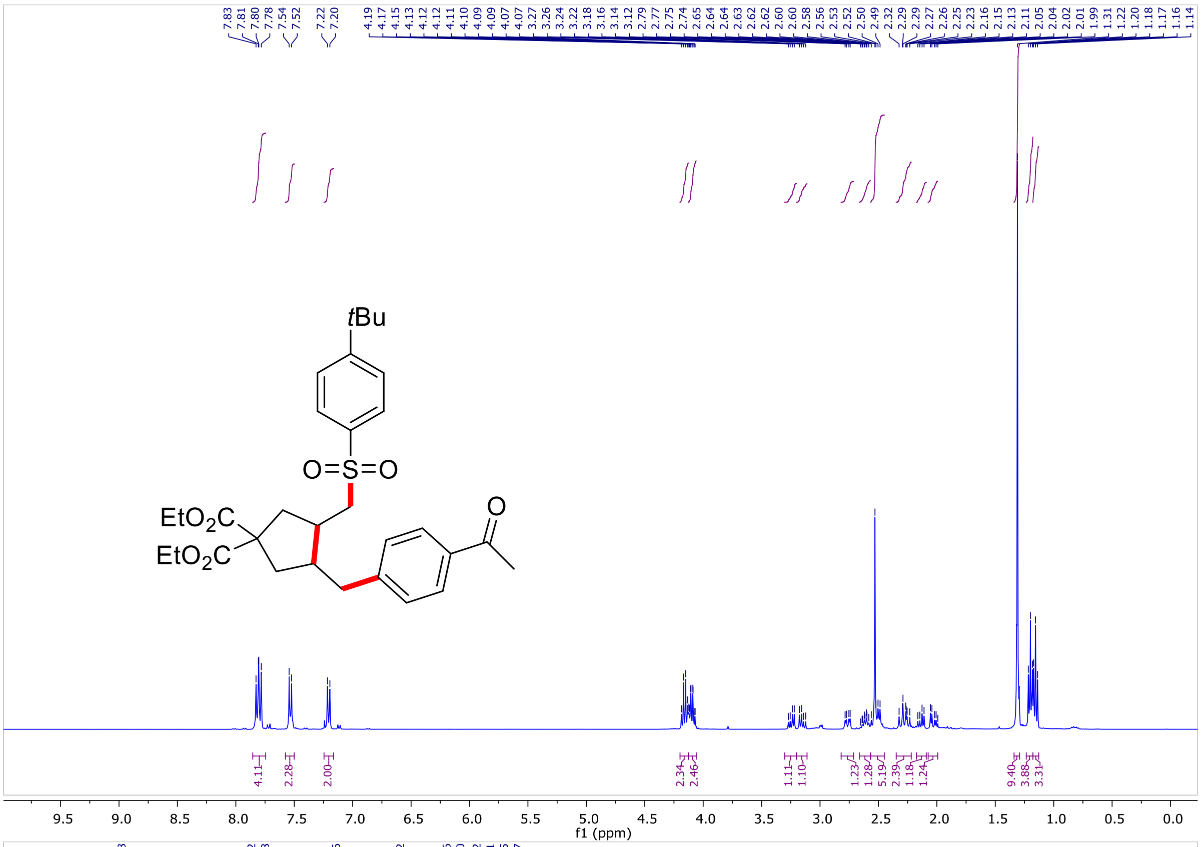

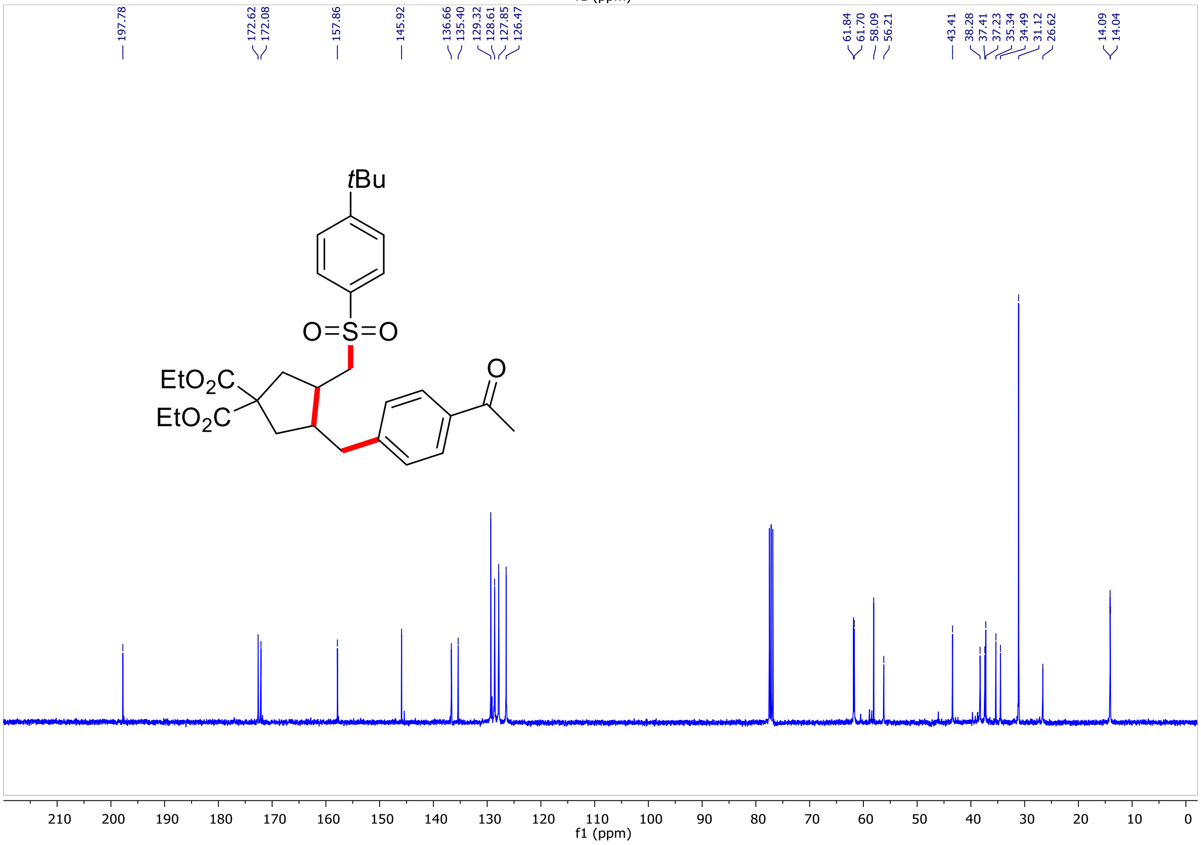

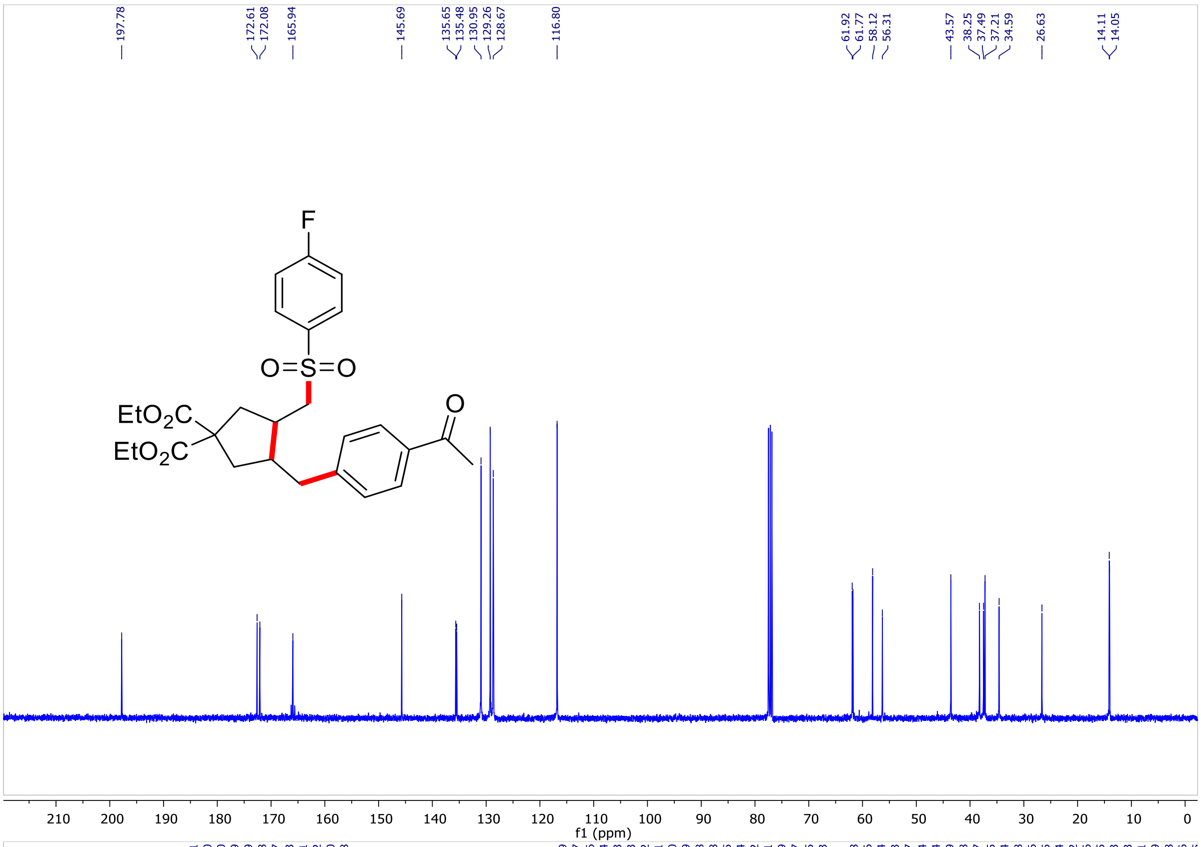
197.78
172.61
172.08
165.94
145.69
135.65
135.48
130.95
129.26
128.67
116.80
61.92
61.77
58.12
56.31
43.57
38.25
37.49
37.21
34.59
26.63
14.11
14.05
F
O=S=O
EtO_2C
EtO_2C
O
f1 (ppm)

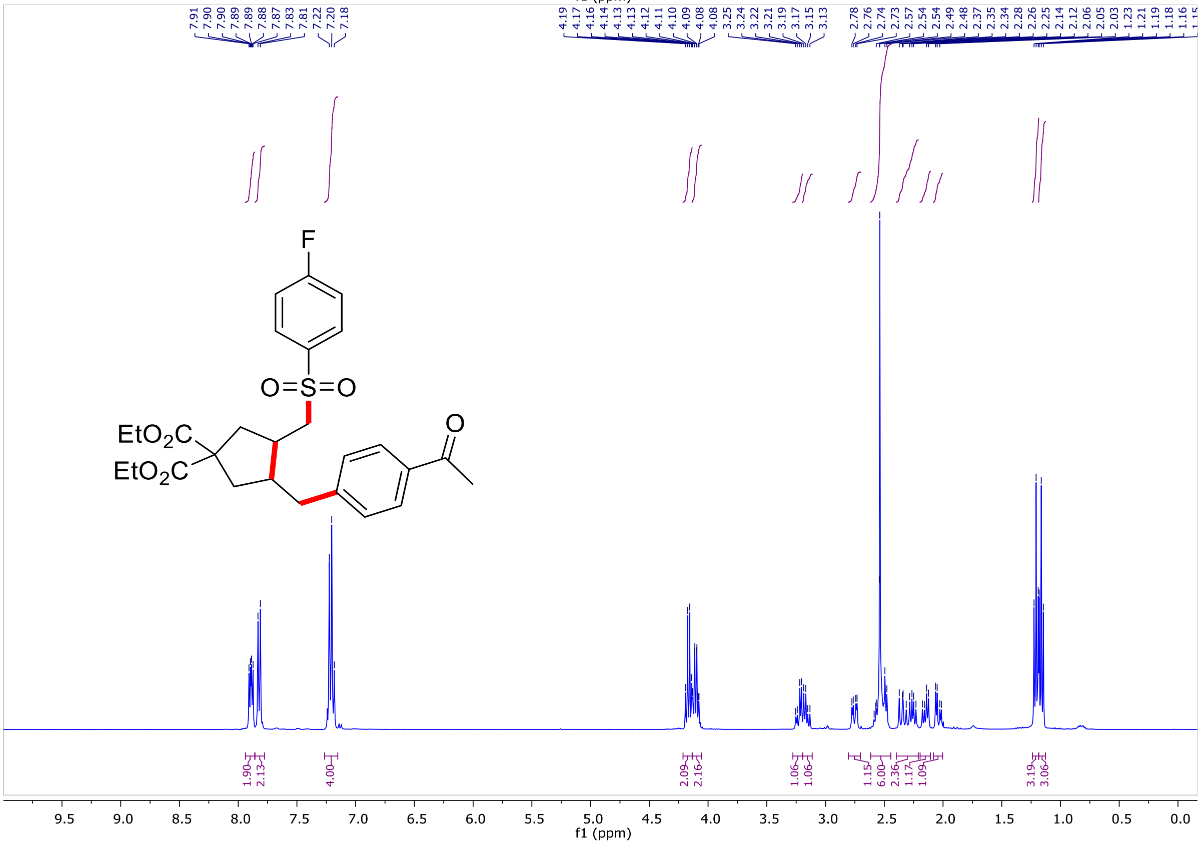
7.91
7.90
7.90
7.89
7.88
7.87
7.83
7.81
7.22
7.20
7.18
4.19
4.17
4.16
4.14
4.13
4.13
4.12
4.11
4.10
4.09
4.08
4.08
3.25
3.24
3.22
3.21
3.19
3.17
3.15
3.13
2.78
2.76
2.74
2.73
2.57
2.54
2.54
2.49
2.48
2.37
2.35
2.34
2.28
2.26
2.25
2.14
2.12
2.06
2.05
2.03
1.23
1.21
1.19
1.18
1.16
F
O=S=O
EtO_2C
EtO_2C
O
1.90
2.13
4.00
2.09
2.16
1.06
1.06
1.15
6.00
2.36
1.17
1.09
3.19
3.06
f1 (ppm)

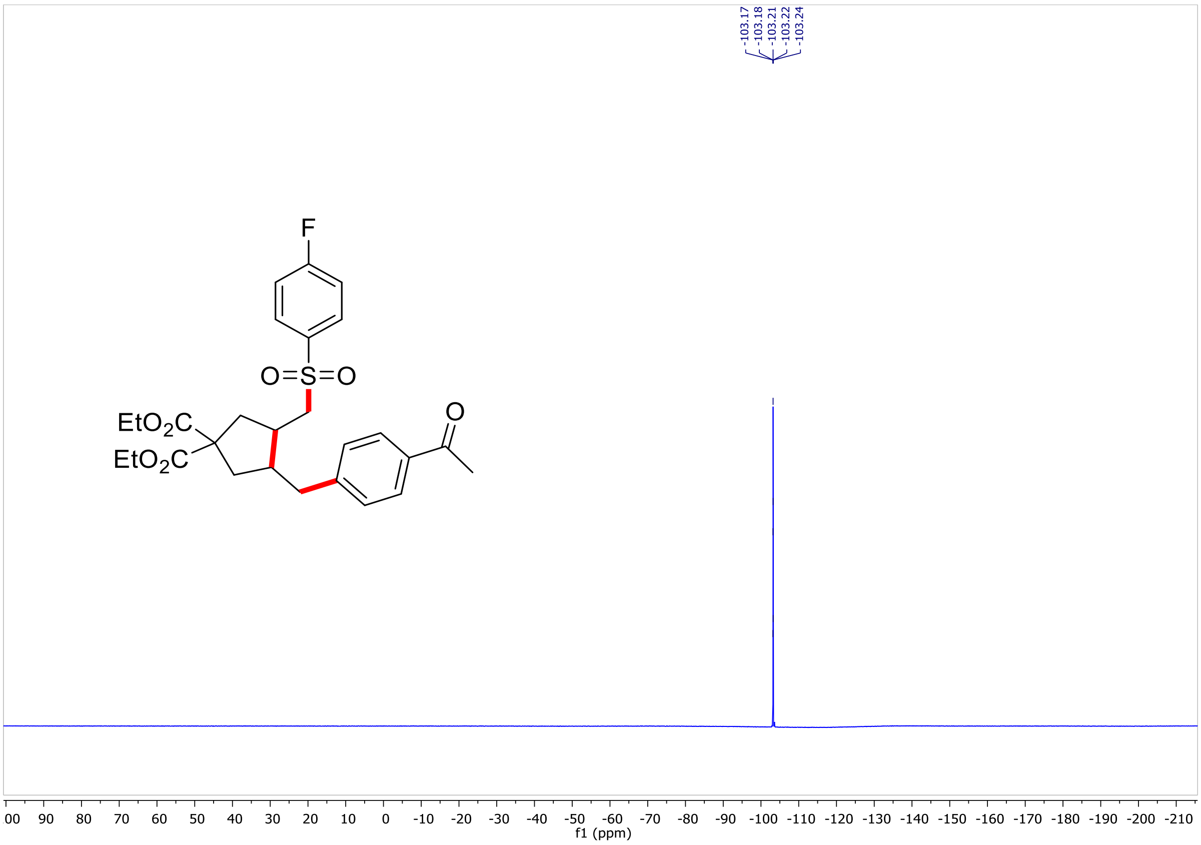
-103.17
-103.18
-103.21
-103.22
-103.24
F
O=S=O
EtO2C
EtO2C
O
00 90 80 70 60 50 40 30 20 10 0 -10 -20 -30 -40 -50 -60 -70 -80 -90 -100 -110 -120 -130 -140 -150 -160 -170 -180 -190 -200 -210
f1 (ppm)

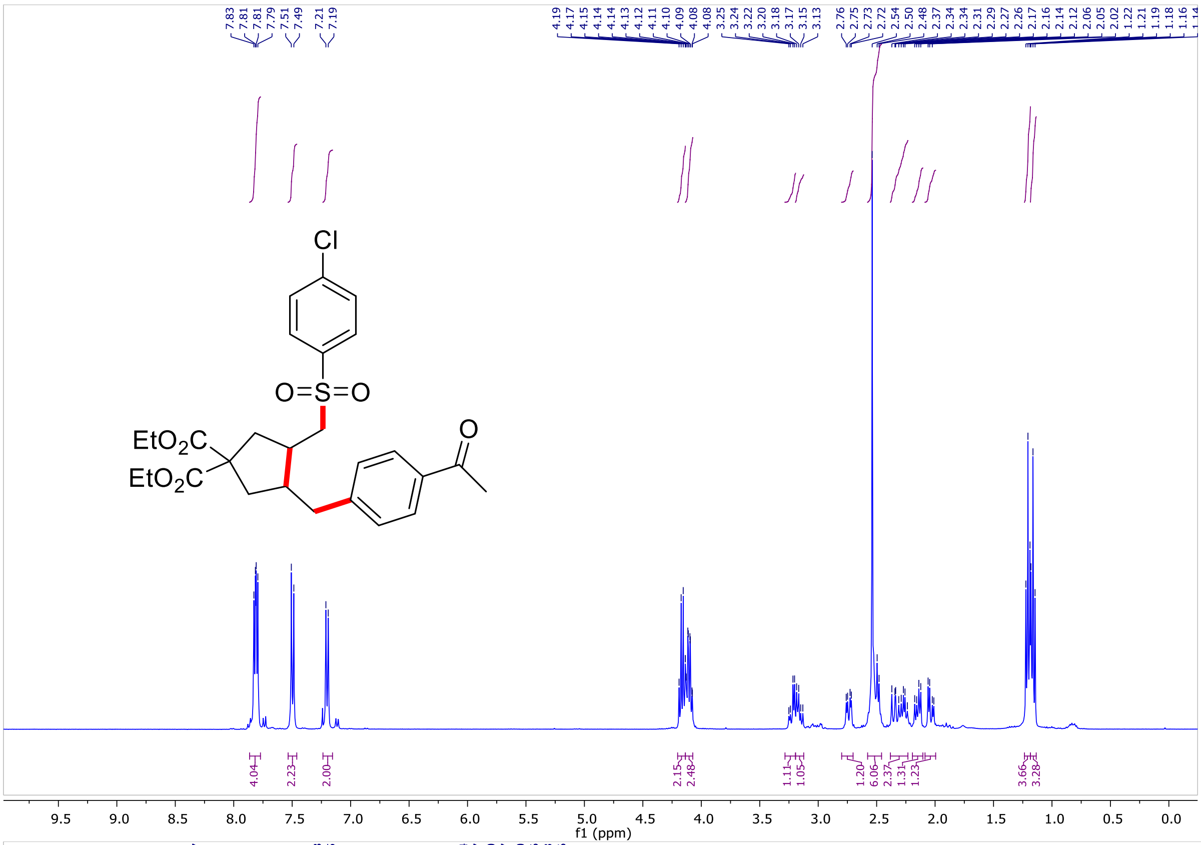

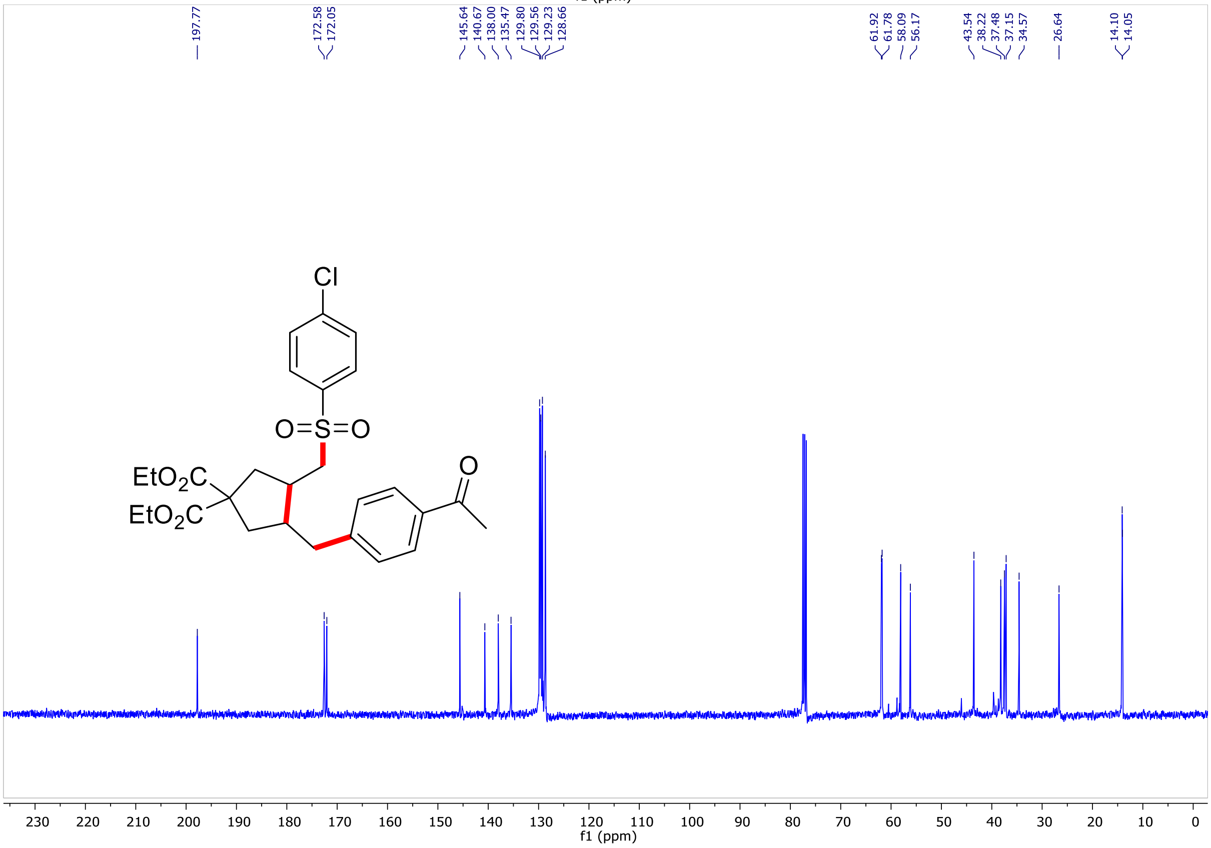

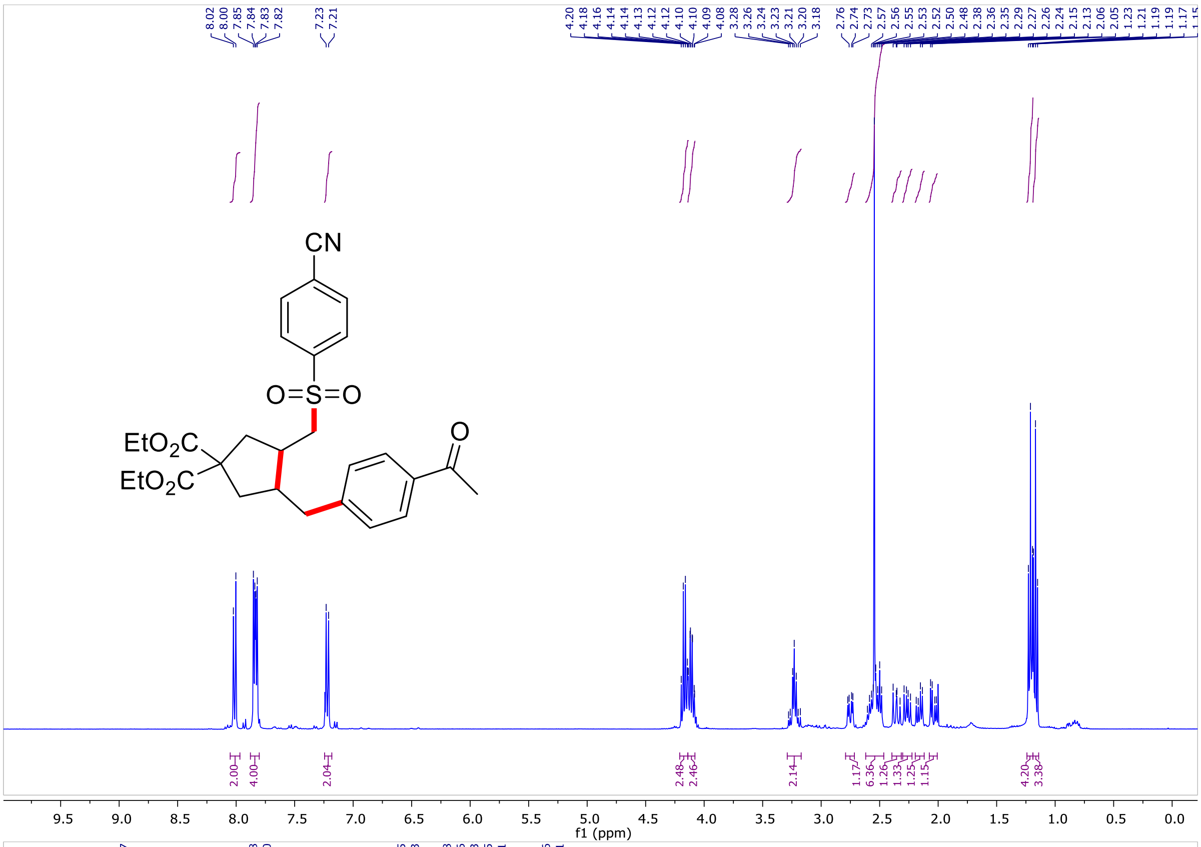
8.02
8.00
7.85
7.84
7.83
7.82
7.23
7.21
CN
O=S=O
EtO2C
EtO2C
O
2.00
4.00
2.04
2.48
2.46
2.14
4.20
3.38
f1 (ppm)

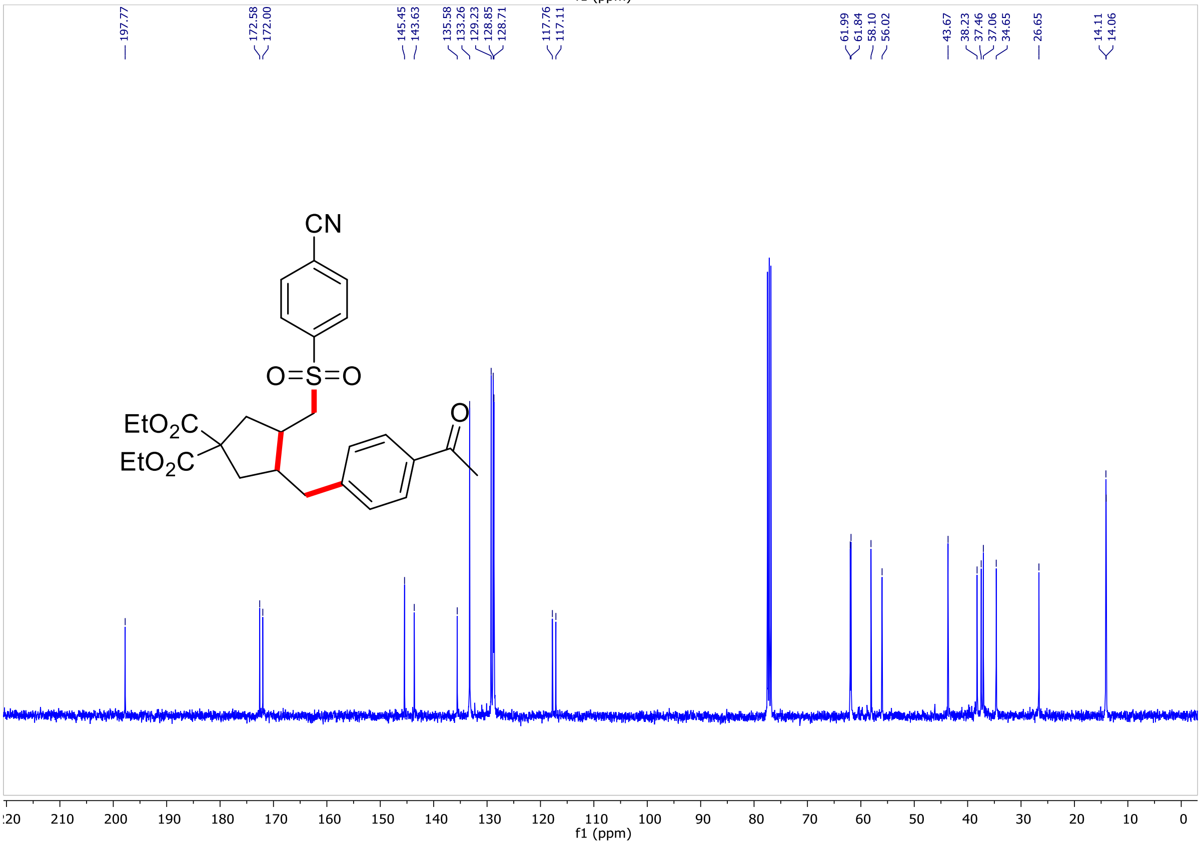
197.77
172.58
172.00
145.45
143.63
135.58
133.26
129.23
128.85
128.71
117.76
117.11
61.99
61.84
58.10
56.02
43.67
38.23
37.46
37.06
34.65
26.65
14.11
14.06
CN
O=S=O
EtO2C
EtO2C
O
f1 (ppm)

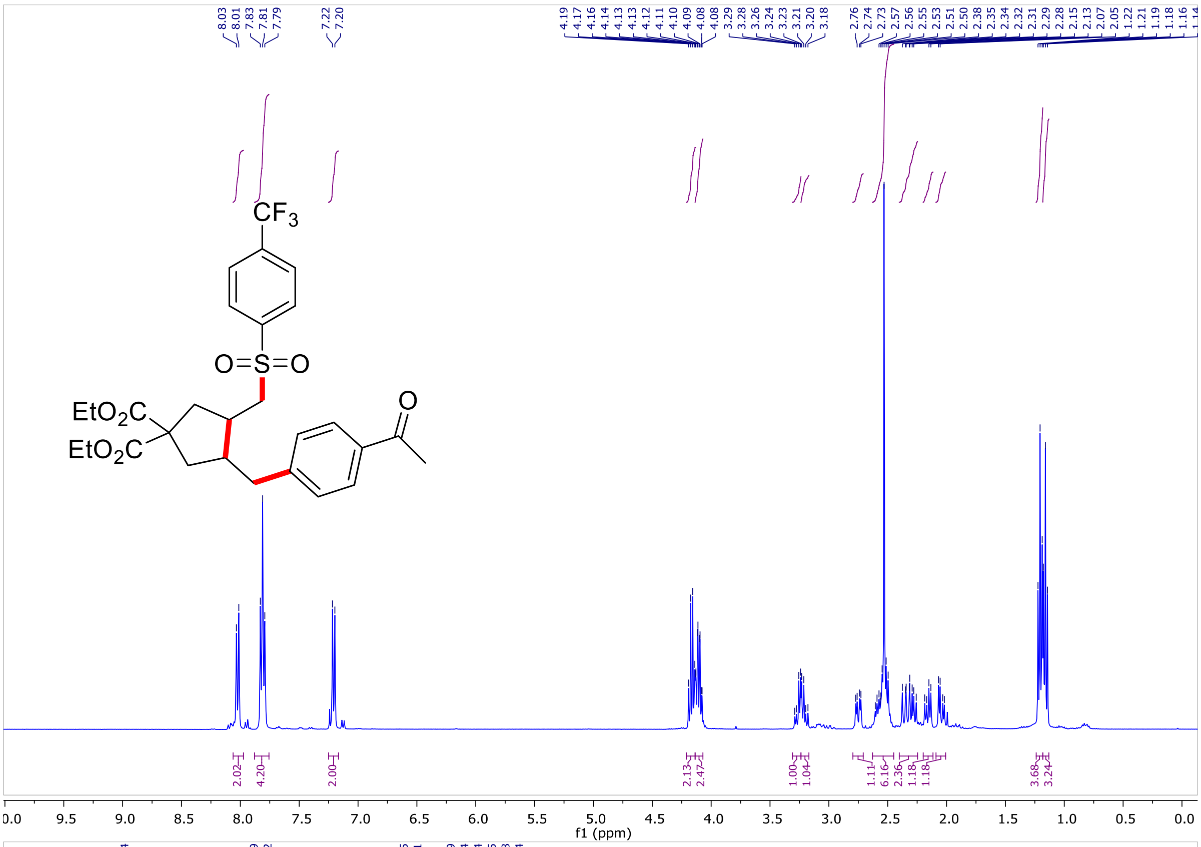

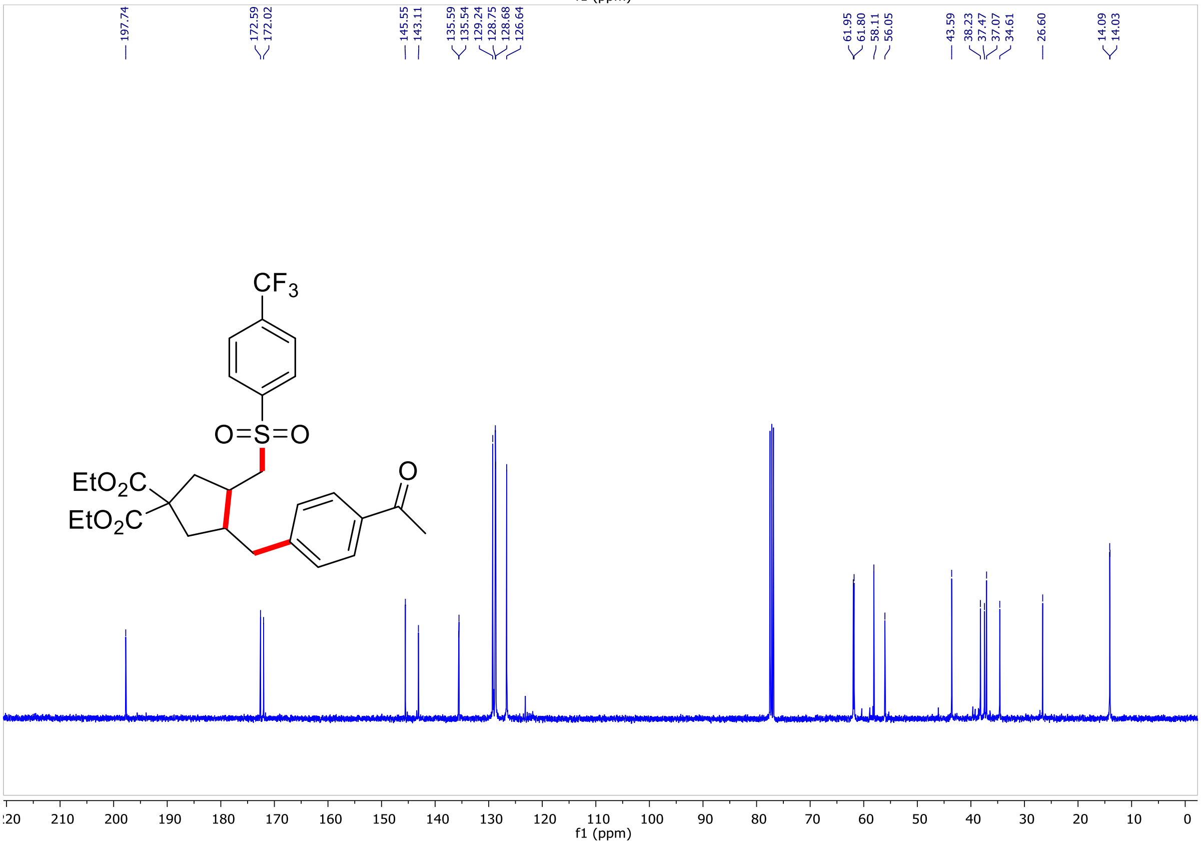

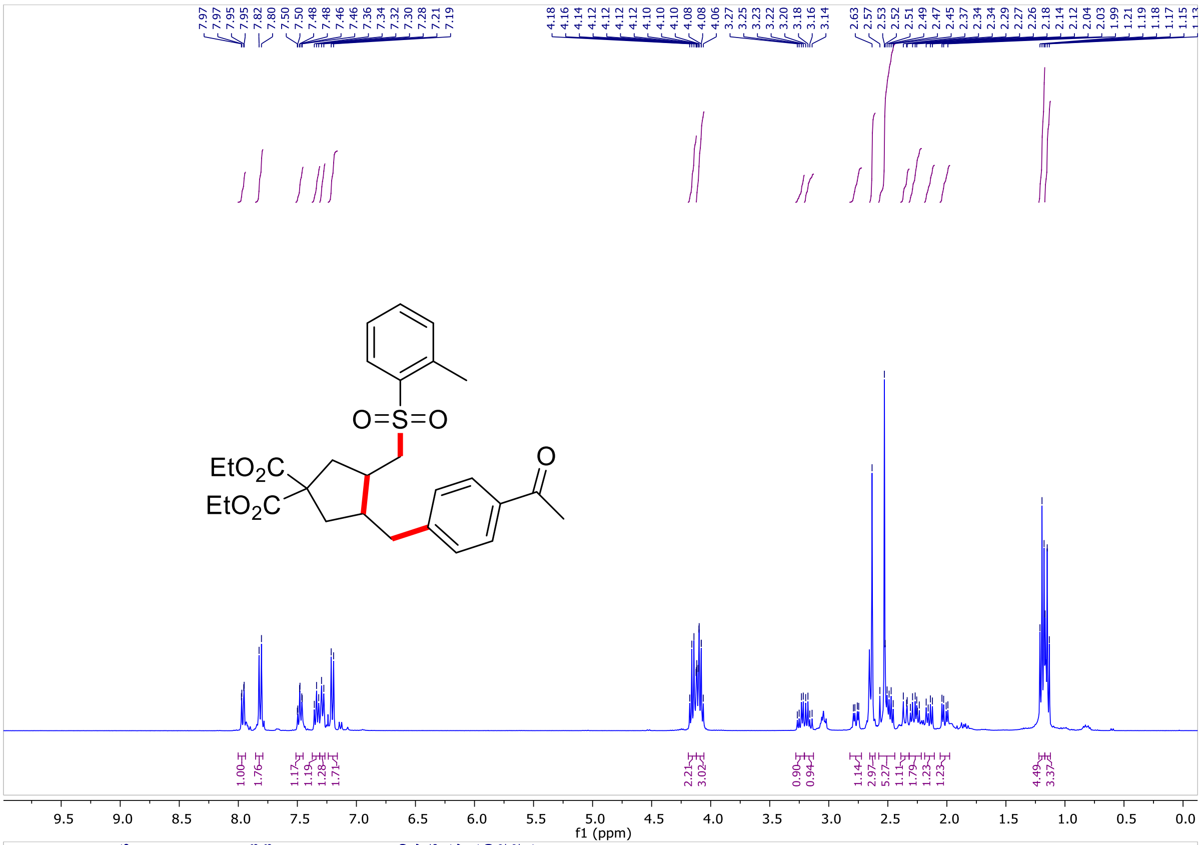

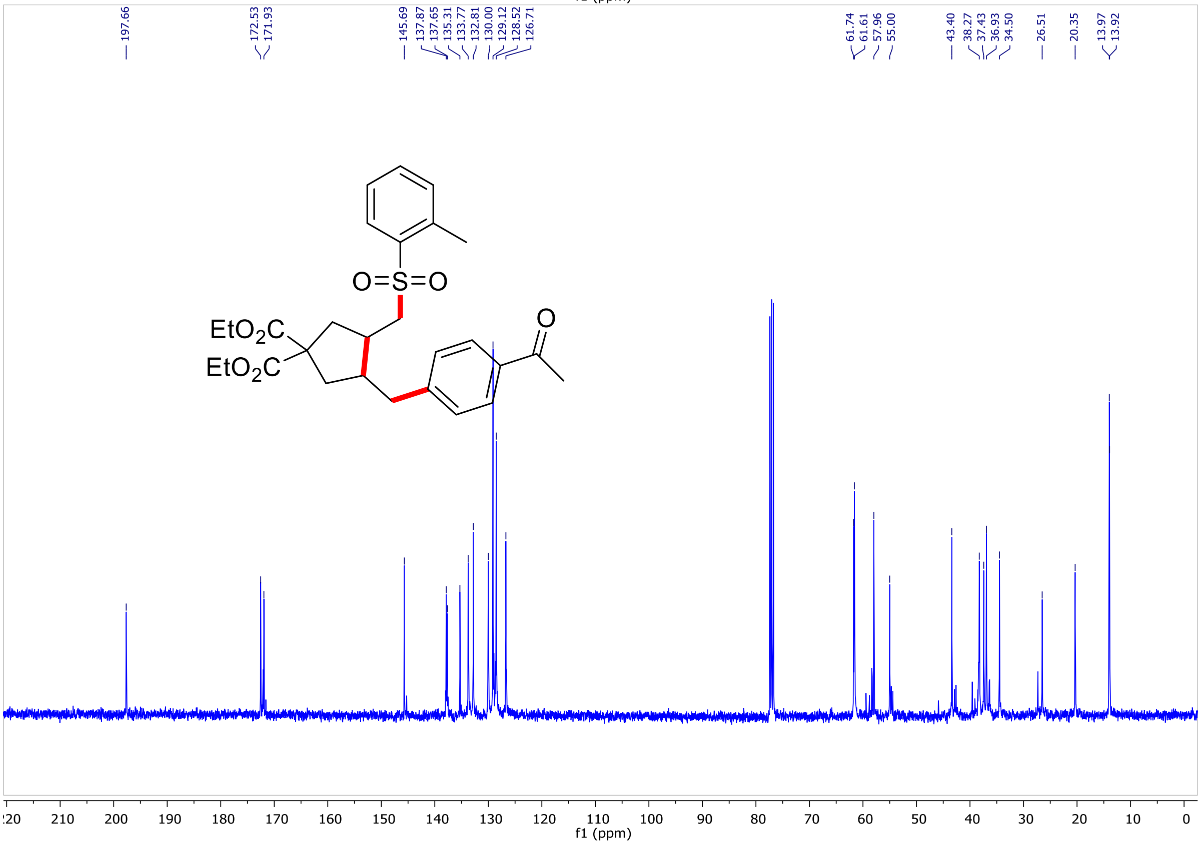

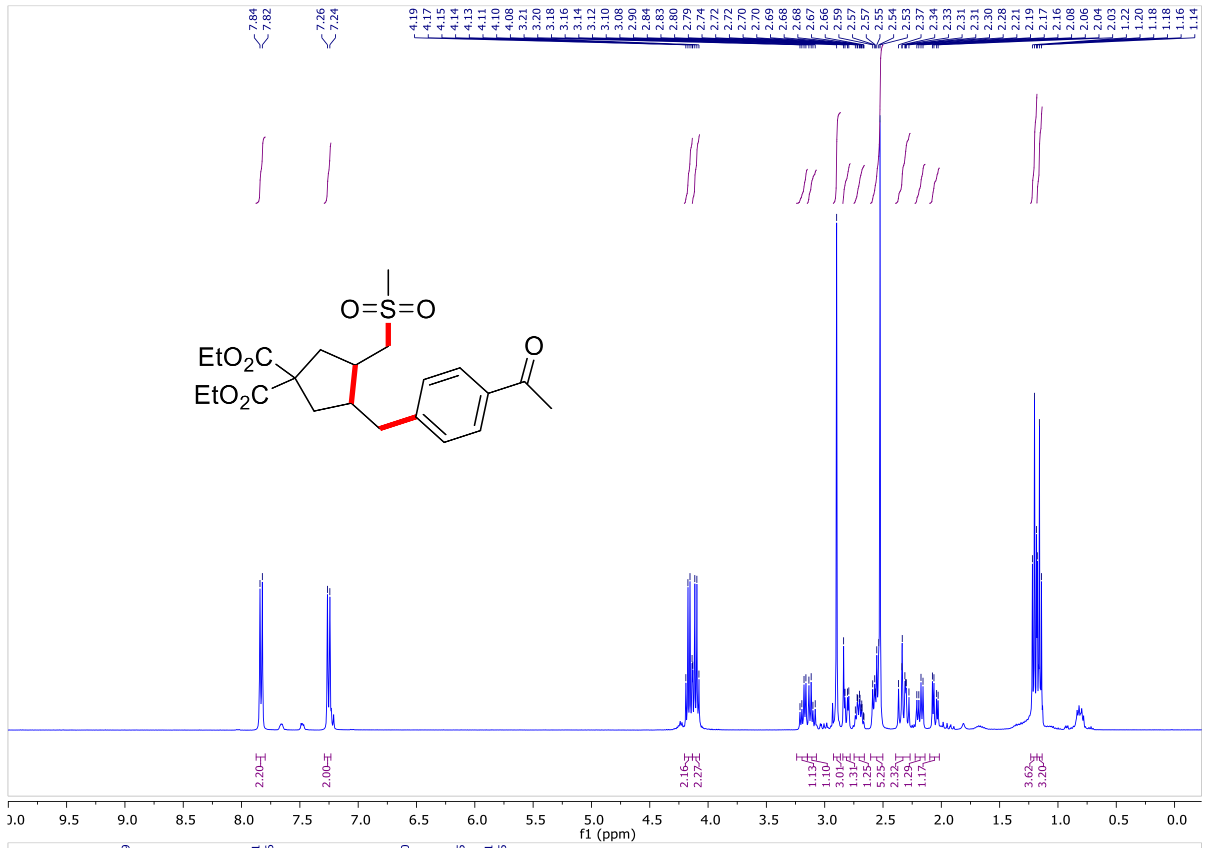

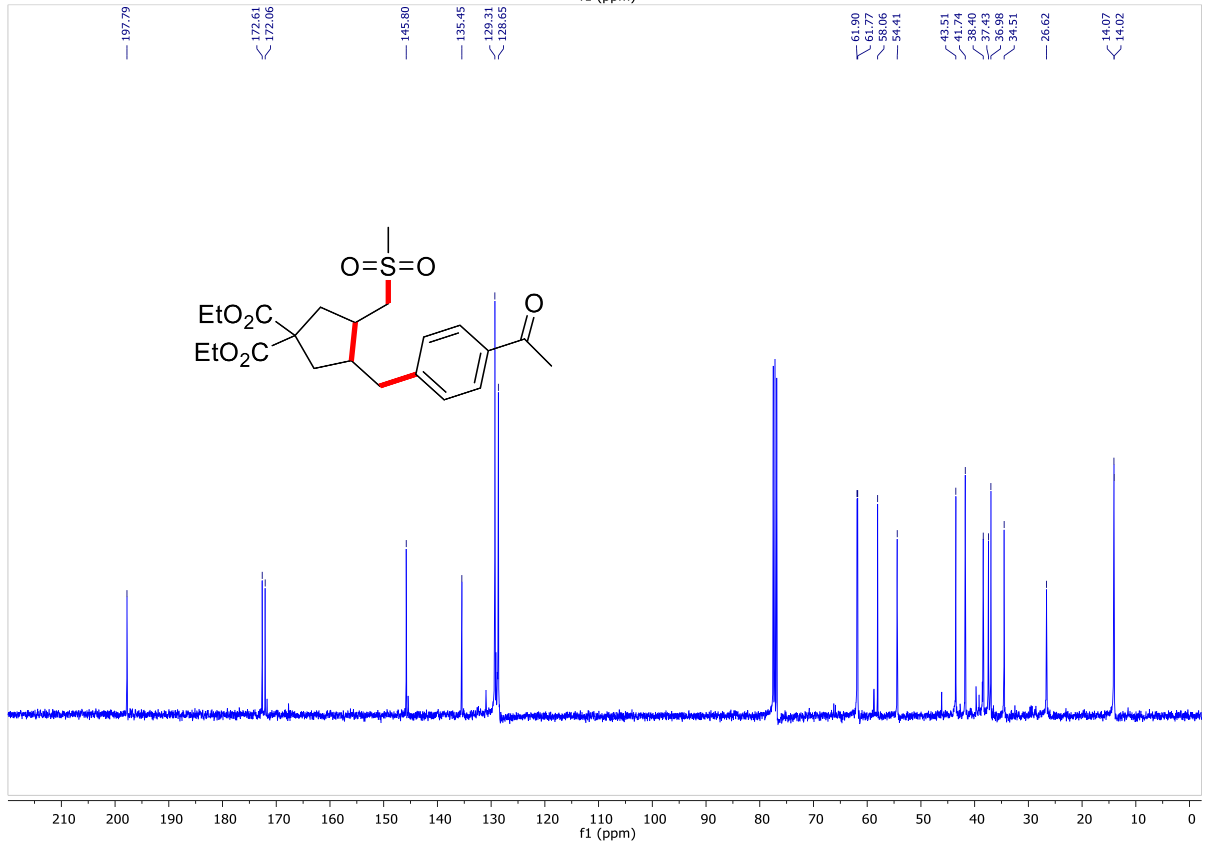

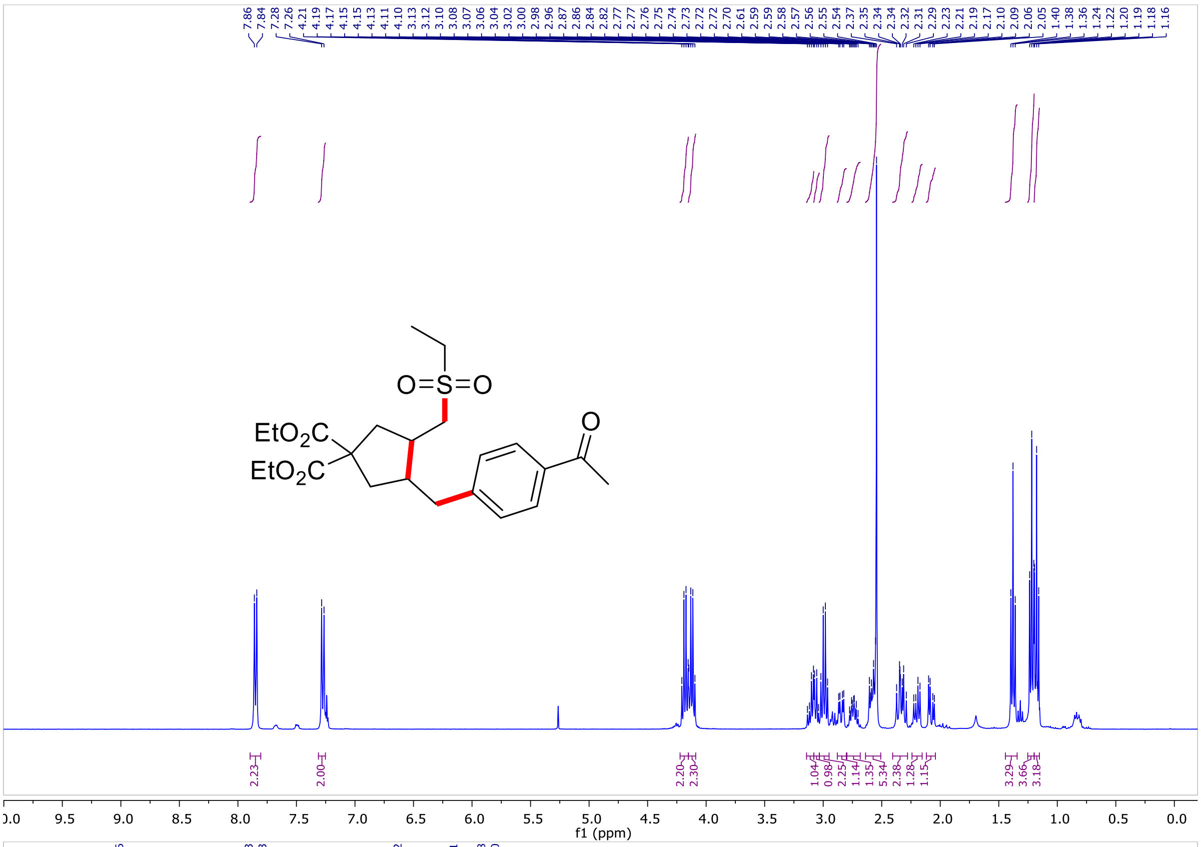
7.86
7.84
7.28
7.26
4.21
4.19
4.17
4.15
4.15
4.13
4.11
4.10
3.13
3.12
3.10
3.08
3.07
3.06
3.04
3.02
3.00
2.98
2.96
2.87
2.86
2.84
2.82
2.77
2.77
2.76
2.75
2.74
2.73
2.72
2.72
2.70
2.61
2.59
2.59
2.58
2.57
2.56
2.55
2.54
2.37
2.35
2.34
2.34
2.32
2.31
2.29
2.23
2.21
2.19
2.17
2.10
2.09
2.06
2.05
1.40
1.38
1.36
1.24
1.22
1.20
1.19
1.18
1.16
EtO2C
EtO2C
O=S=O
O
2.23
2.00
2.20
2.30
1.04
0.98
2.25
1.14
1.35
5.34
2.38
1.28
1.15
3.29
3.66
3.18
f1 (ppm)

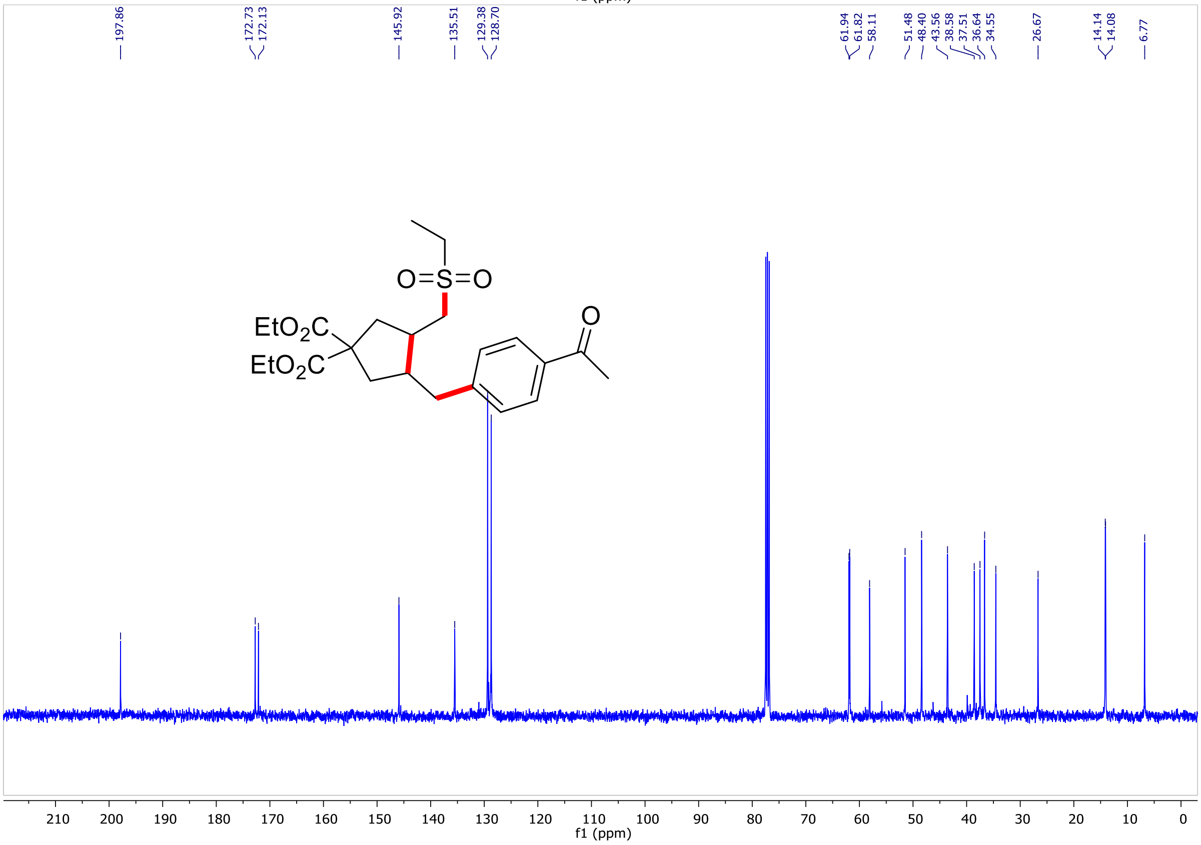
197.86
172.73
172.13
145.92
135.51
129.38
128.70
61.94
61.82
58.11
51.48
48.40
43.56
38.58
37.51
36.64
34.55
26.67
14.14
14.08
6.77
EtO2C
EtO2C
O=S=O
O
f1 (ppm)

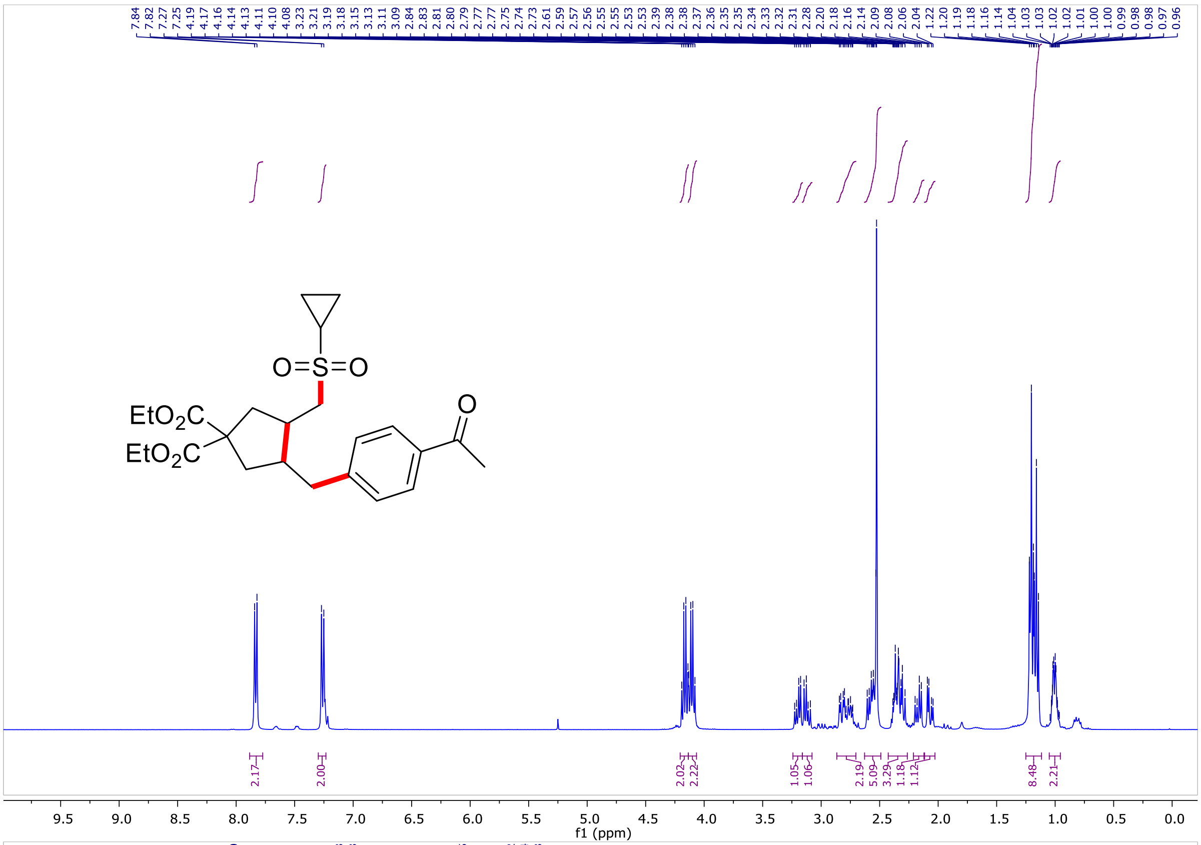
7.84 7.82 7.27 7.25 4.19 4.17 4.16 4.14 4.13 4.11 4.10 4.08 3.23 3.21 3.19 3.18 3.15 3.13 3.11 3.09 2.84 2.83 2.81 2.80 2.79 2.77 2.77 2.75 2.74 2.73 2.61 2.59 2.57 2.56 2.55 2.55 2.53 2.53 2.39 2.38 2.38 2.37 2.36 2.35 2.35 2.34 2.33 2.32 2.31 2.28 2.20 2.18 2.16 2.14 2.09 2.08 2.06 2.04 1.22 1.20 1.19 1.18 1.16 1.14 1.04 1.03 1.03 1.02 1.02 1.01 1.00 1.00 0.99 0.98 0.98 0.97 0.96
O=S=O
EtO2C
EtO2C
O
2.17 2.00 2.02 2.22 1.05 1.06 2.19 5.09 3.29 1.18 1.12 8.48 2.21
9.5 9.0 8.5 8.0 7.5 7.0 6.5 6.0 5.5 5.0 4.5 4.0 3.5 3.0 2.5 2.0 1.5 1.0 0.5 0.0
f1 (ppm)

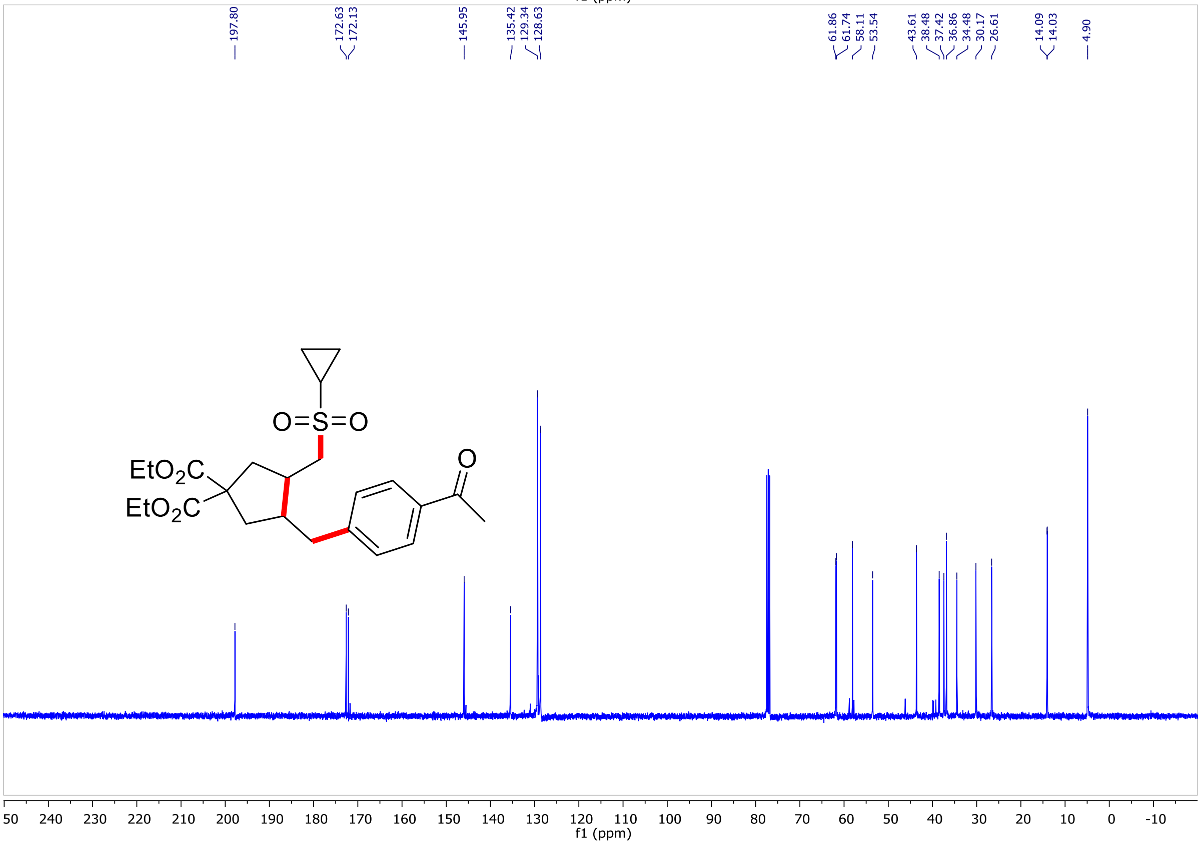
197.80 172.63 172.13 145.95 135.42 129.34 128.63 61.86 61.74 58.11 53.54 43.61 38.48 37.42 36.86 34.48 30.17 26.61 14.09 14.03 4.90
O=S=O
EtO2C
EtO2C
O
50 240 230 220 210 200 190 180 170 160 150 140 130 120 110 100 90 80 70 60 50 40 30 20 10 0 -10
f1 (ppm)

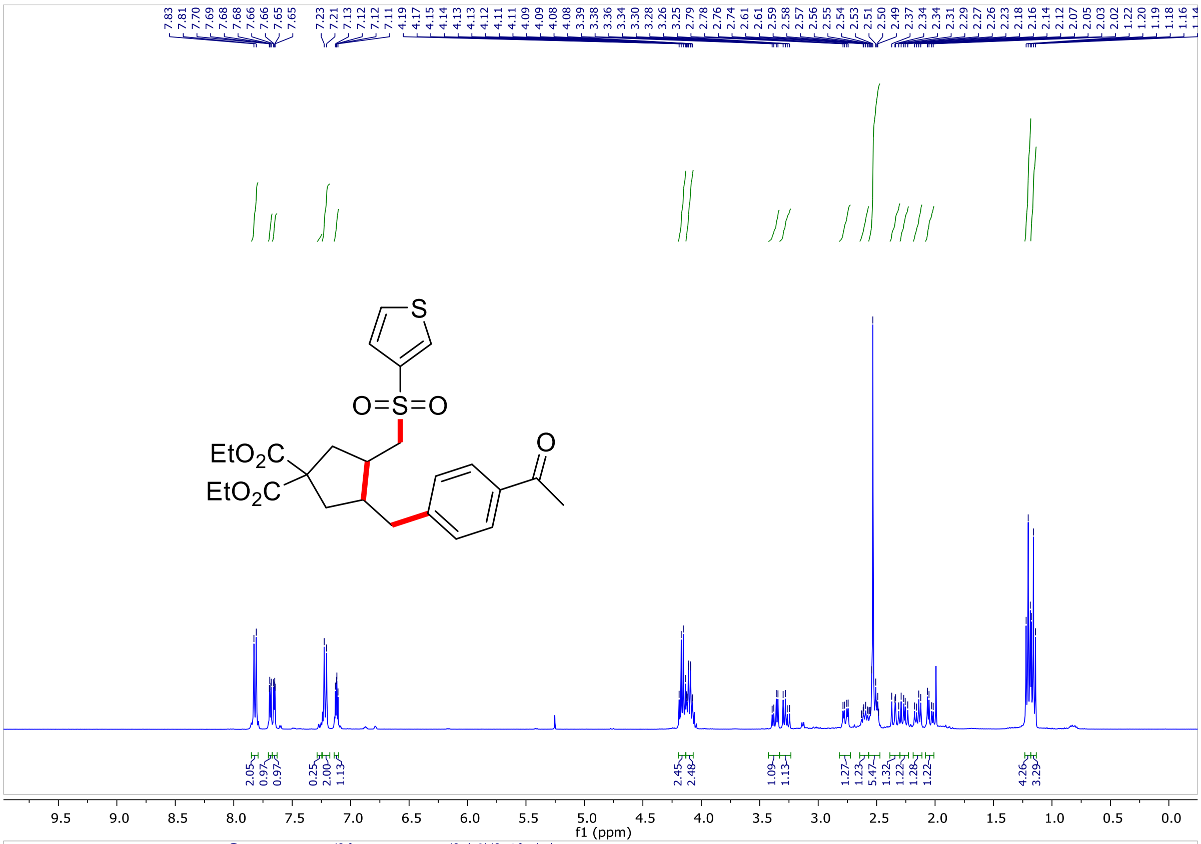
7.83
7.81
7.70
7.69
7.68
7.66
7.66
7.65
7.65
7.23
7.21
7.13
7.12
7.12
7.11
4.19
4.17
4.15
4.14
4.13
4.13
4.12
4.11
4.11
4.09
4.09
4.08
4.08
3.39
3.38
3.36
3.34
3.30
3.28
3.26
3.25
2.79
2.78
2.76
2.74
2.61
2.61
2.59
2.58
2.57
2.56
2.55
2.54
2.53
2.51
2.50
2.49
2.37
2.34
2.34
2.31
2.29
2.27
2.26
2.23
2.18
2.16
2.14
2.12
2.07
2.05
2.03
2.02
1.22
1.20
1.19
1.18
1.16
S
O=S=O
O
EtO2C
EtO2C
2.05
0.97
0.97
0.25
2.00
1.13
2.45
2.48
1.09
1.13
1.27
1.23
5.47
1.32
1.22
1.28
1.22
4.26
3.29
9.5
9.0
8.5
8.0
7.5
7.0
6.5
6.0
5.5
5.0
4.5
4.0
3.5
3.0
2.5
2.0
1.5
1.0
0.5
0.0
f1 (ppm)

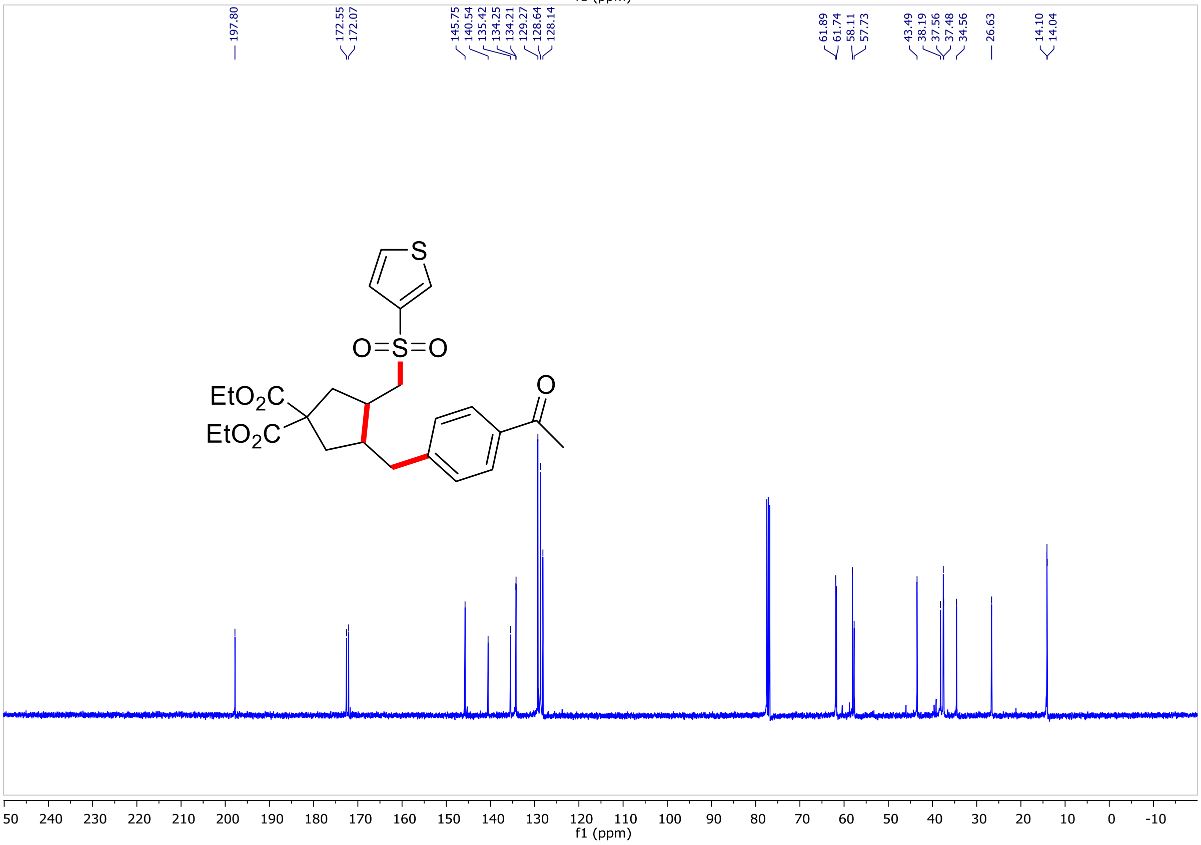
197.80
172.55
172.07
145.75
140.54
135.42
134.25
134.21
129.27
128.64
128.14
61.89
61.74
58.11
57.73
43.49
38.19
37.56
37.48
34.56
26.63
14.10
14.04
S
O=S=O
O
EtO2C
EtO2C
250
240
230
220
210
200
190
180
170
160
150
140
130
120
110
100
90
80
70
60
50
40
30
20
10
0
-10
f1 (ppm)

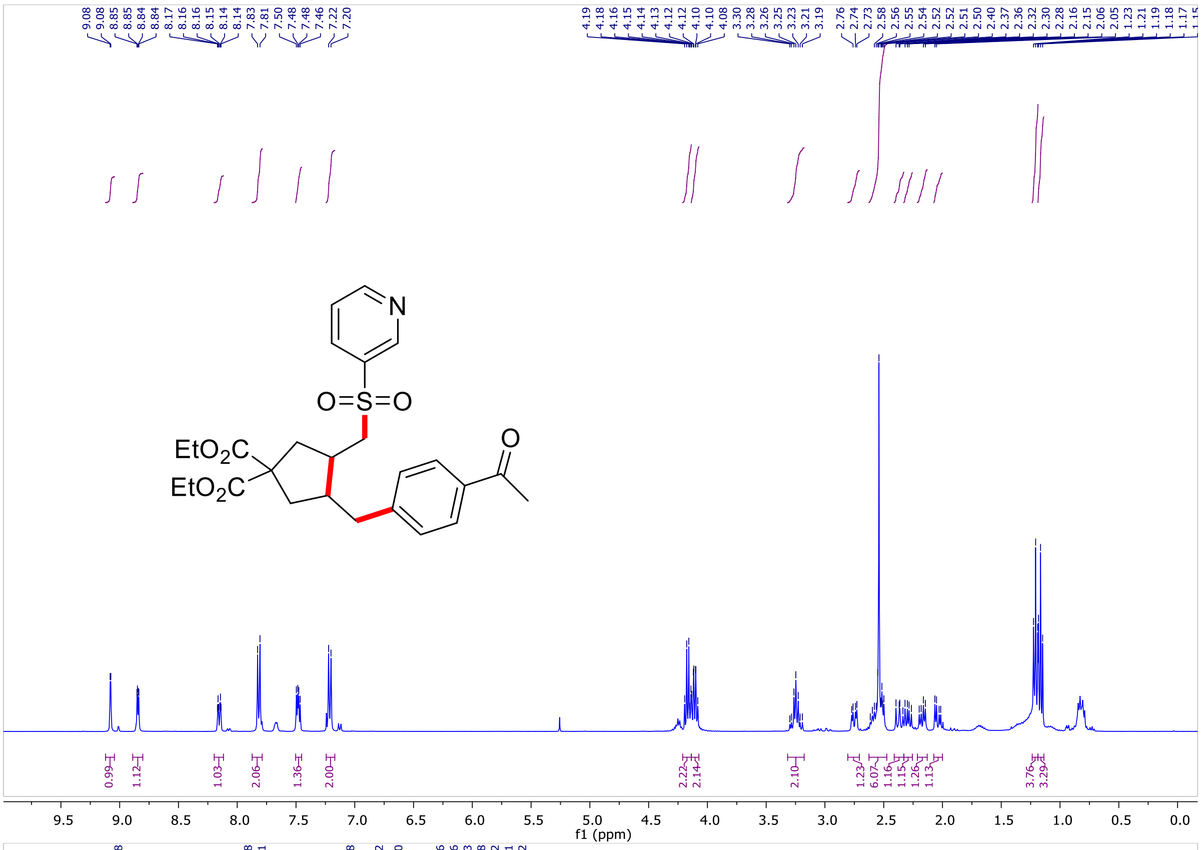
9.08 9.08 8.85 8.85 8.84 8.84 8.17 8.16 8.16 8.15 8.14 8.14 7.83 7.81 7.50 7.48 7.48 7.46 7.22 7.20
4.19 4.18 4.16 4.15 4.14 4.13 4.12 4.12 4.10 4.10 4.08 3.30 3.28 3.26 3.25 3.23 3.21 3.19 2.76 2.74 2.73 2.58 2.56 2.55 2.55 2.54 2.52 2.51 2.50 2.40 2.37 2.36 2.32 2.30 2.28 2.16 2.15 2.06 2.05 1.23 1.21 1.19 1.18 1.17
N
O=S=O
EtO2C
EtO2C
O
0.99 1.12 1.03 2.06 1.36 2.00 2.22 2.14 2.10 1.23 6.07 1.16 1.15 1.26 1.13 3.76 3.29
9.5 9.0 8.5 8.0 7.5 7.0 6.5 6.0 5.5 5.0 4.5 4.0 3.5 3.0 2.5 2.0 1.5 1.0 0.5 0.0
f1 (ppm)

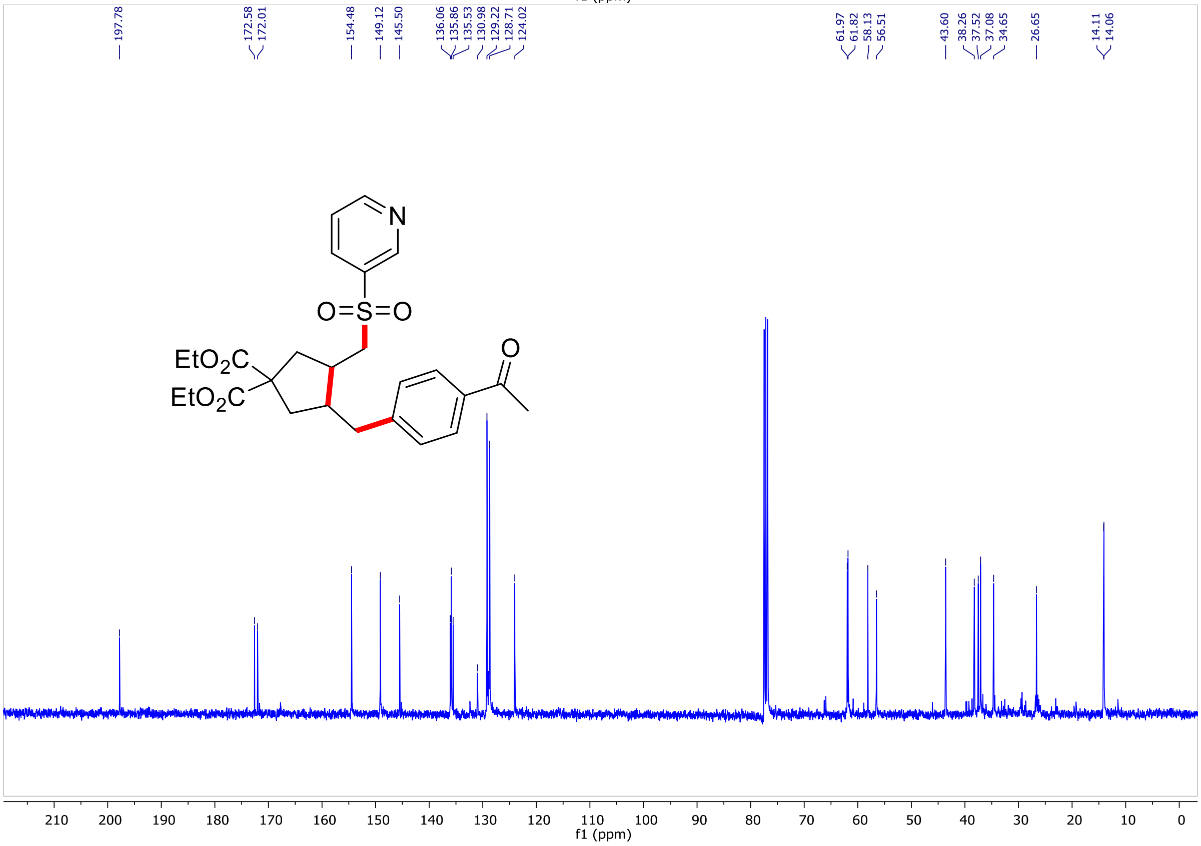
197.78 172.58 172.01 154.48 149.12 145.50 136.06 135.86 135.53 130.98 129.22 128.71 124.02
61.97 61.82 58.13 56.51 43.60 38.26 37.52 37.08 34.65 26.65 14.11 14.06
N
O=S=O
EtO2C
EtO2C
O
210 200 190 180 170 160 150 140 130 120 110 100 90 80 70 60 50 40 30 20 10 0
f1 (ppm)

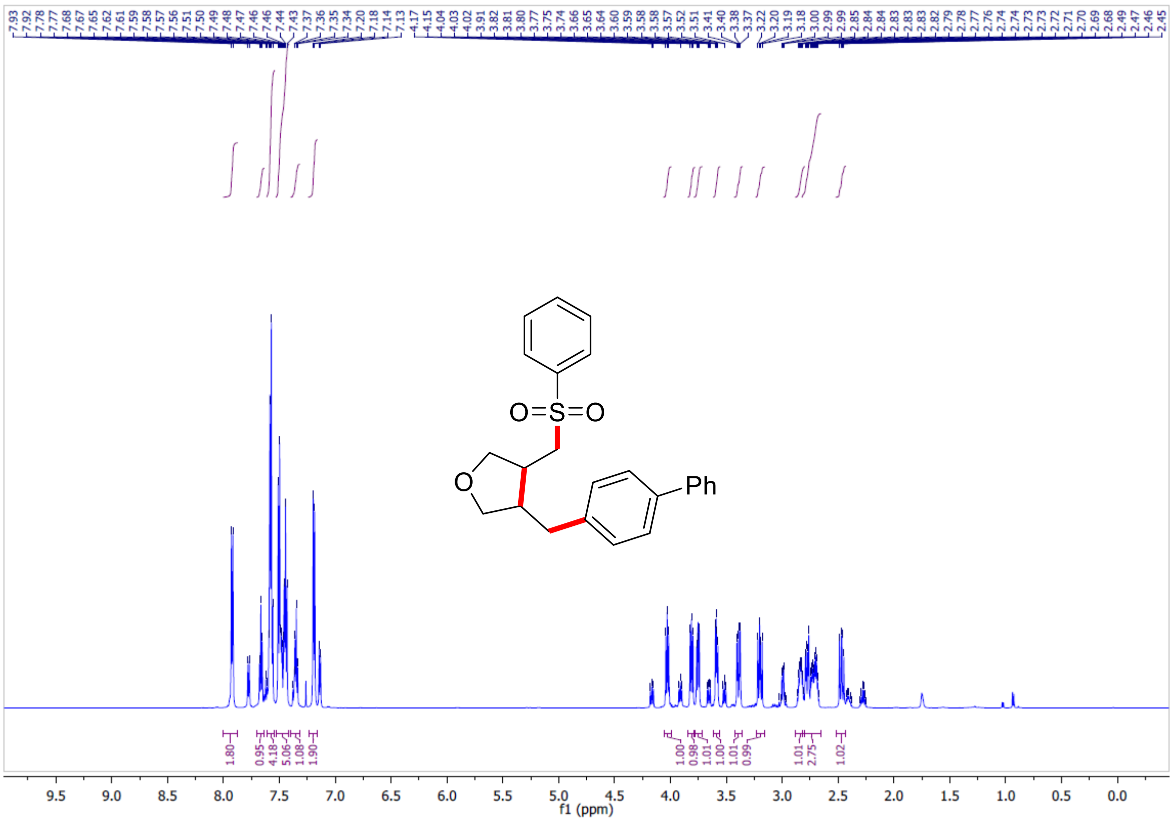

O=S=O
O
Ph
f1 (ppm)

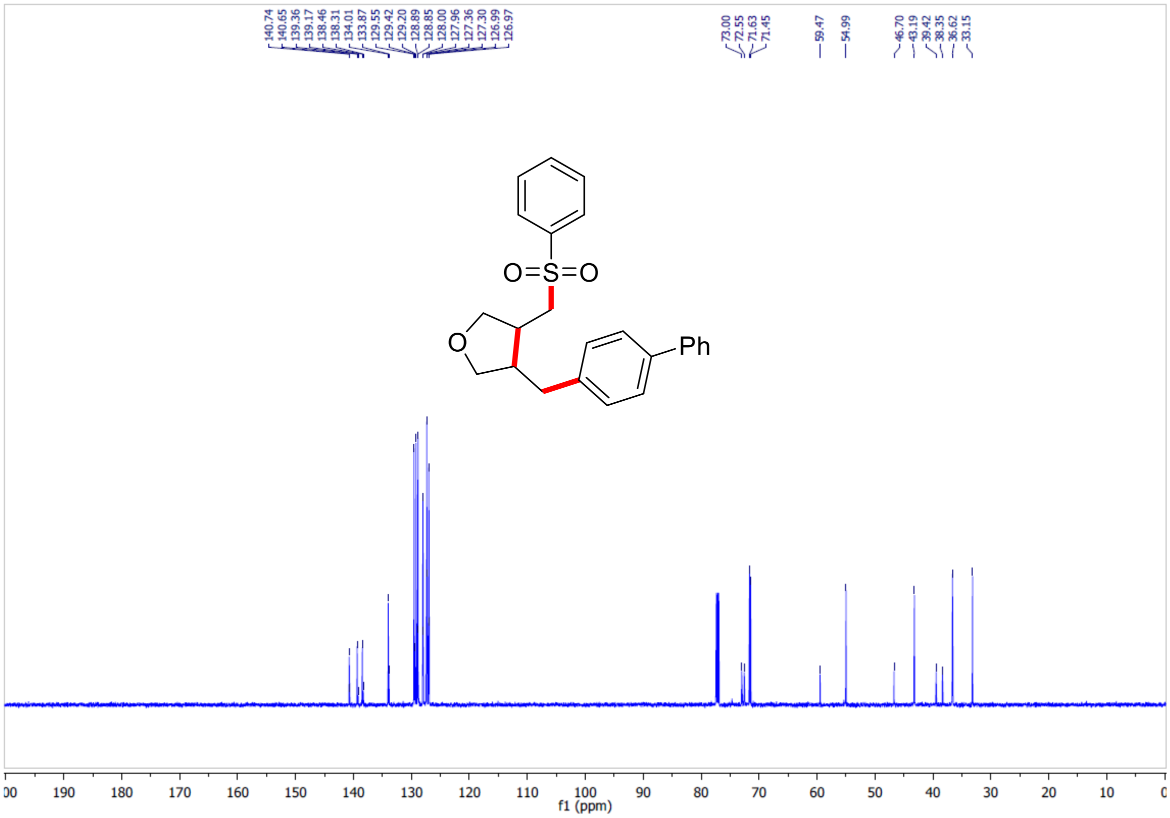

140.74
140.65
139.36
139.17
138.46
138.31
134.01
133.87
129.55
129.42
129.20
128.89
128.85
128.00
127.96
127.36
127.30
126.99
126.97
73.00
72.55
71.63
71.45
59.47
54.99
46.70
43.19
39.42
38.35
36.62
33.15
O=S=O
O
Ph
f1 (ppm)

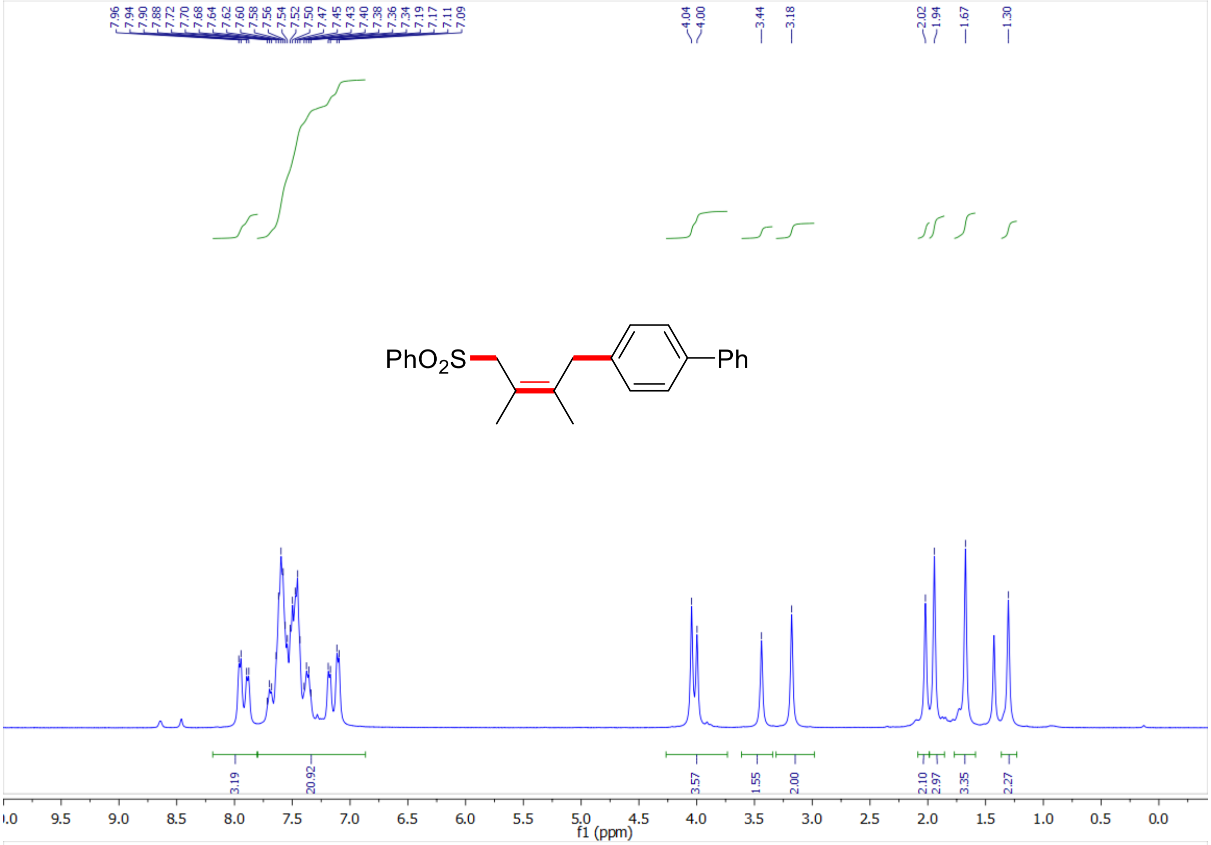
PhO2S
Ph
7.96 7.94 7.90 7.88 7.72 7.70 7.68 7.64 7.62 7.60 7.58 7.56 7.54 7.52 7.50 7.47 7.45 7.43 7.40 7.38 7.36 7.34 7.19 7.17 7.11 7.09
4.04 4.00
3.44
3.18
2.02 1.94
1.67
1.30
3.19 20.92 3.57 1.55 2.00 2.10 2.97 3.35 2.27
f1 (ppm)

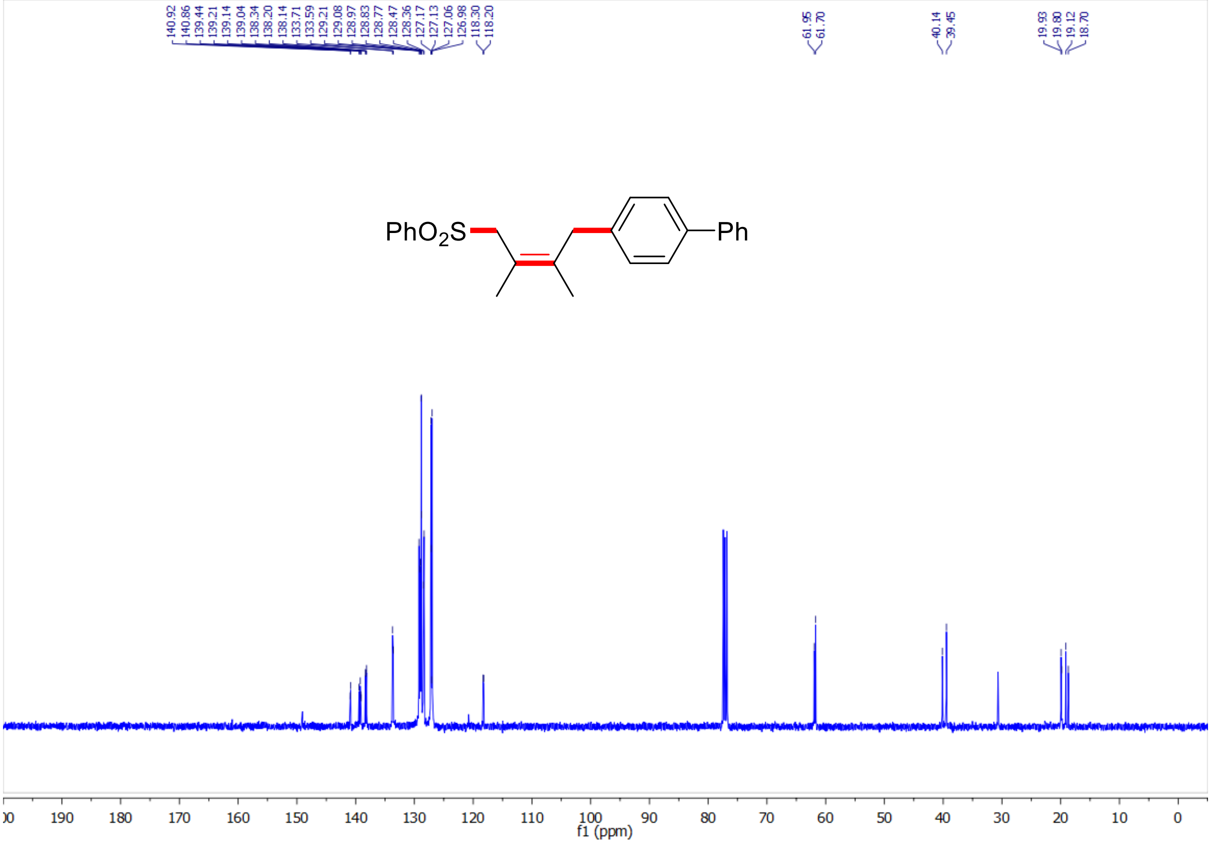
PhO2S
Ph
140.92 140.86 139.44 139.21 139.14 139.04 138.34 138.20 138.14 133.71 133.59 129.21 129.08 128.97 128.83 128.77 128.47 128.36 127.17 127.13 127.06 126.98 118.30 118.20
61.95 61.70
40.14 39.45
19.93 19.80 19.12 18.70
f1 (ppm)

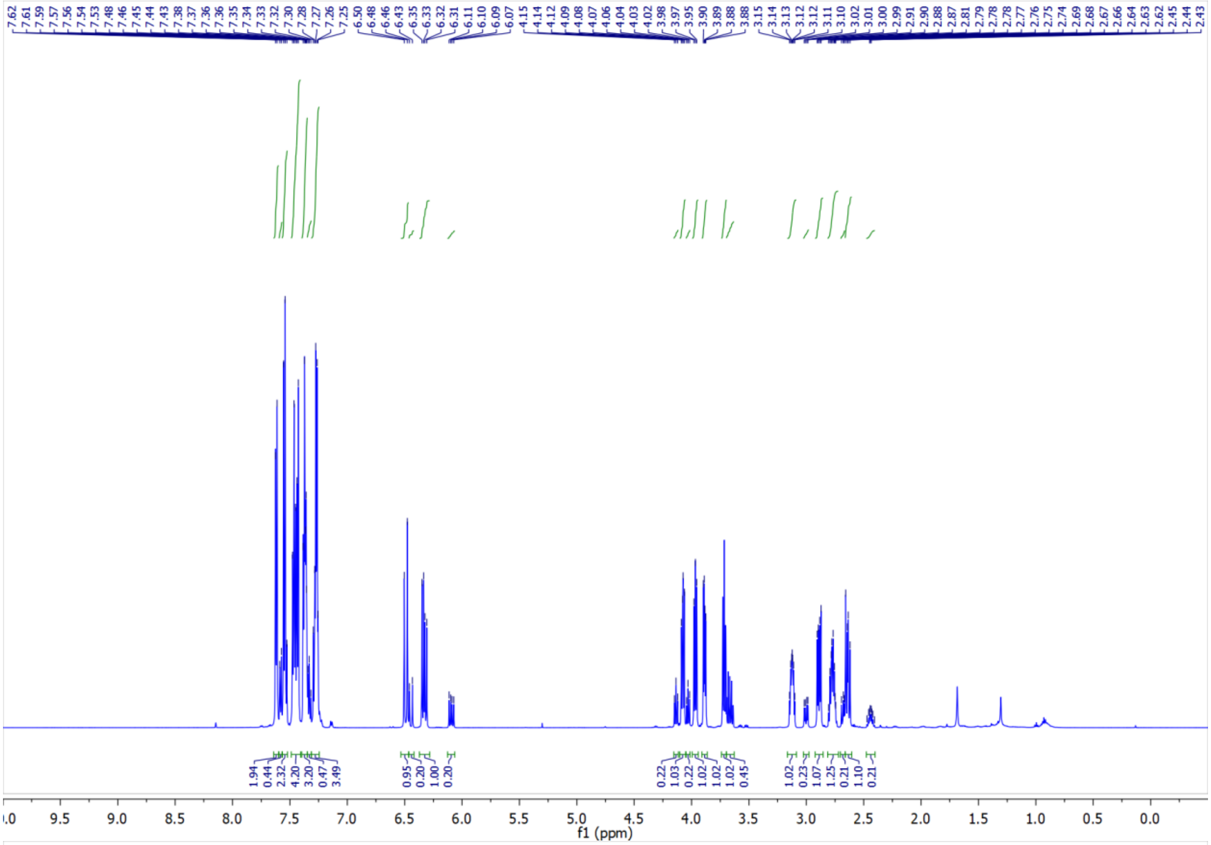
f1 (ppm)

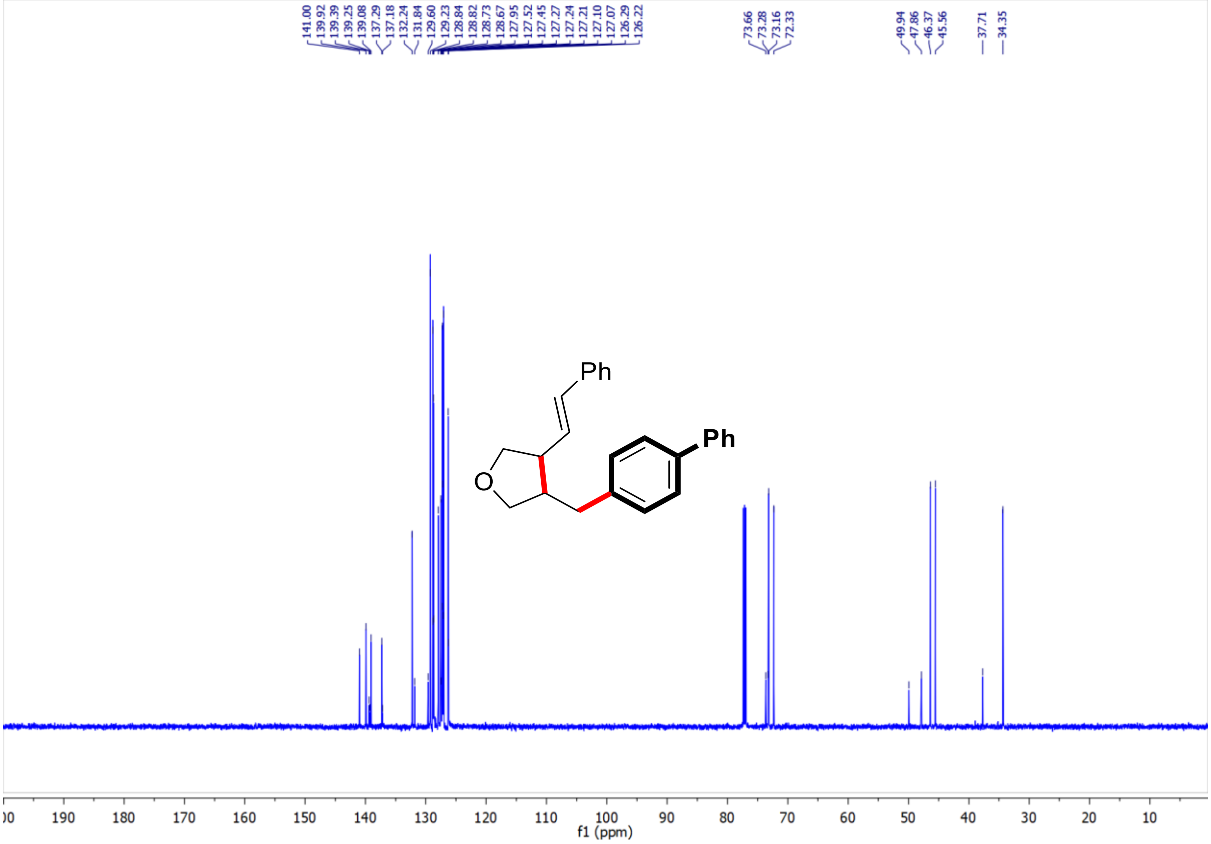
Ph
Ph
O
f1 (ppm)

HSQC

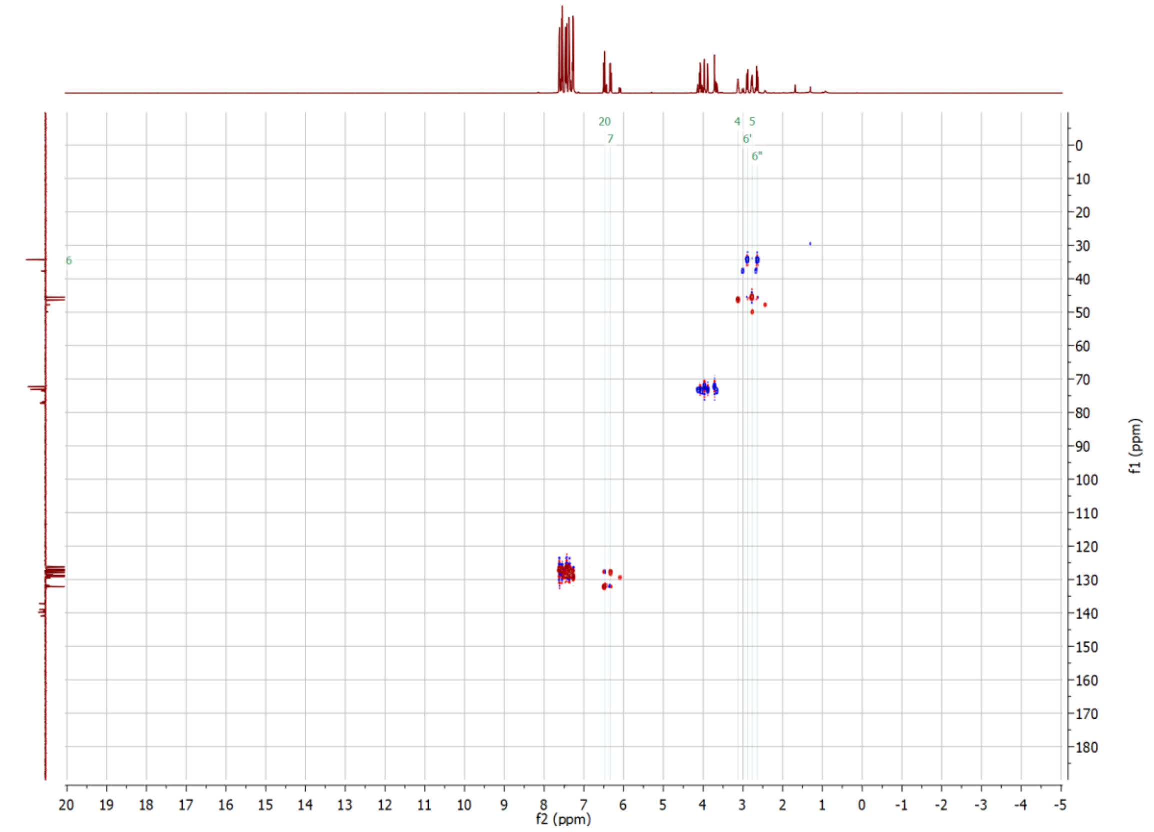
f2 (ppm)
f1 (ppm)
20
7
4 5
6'
6''
6

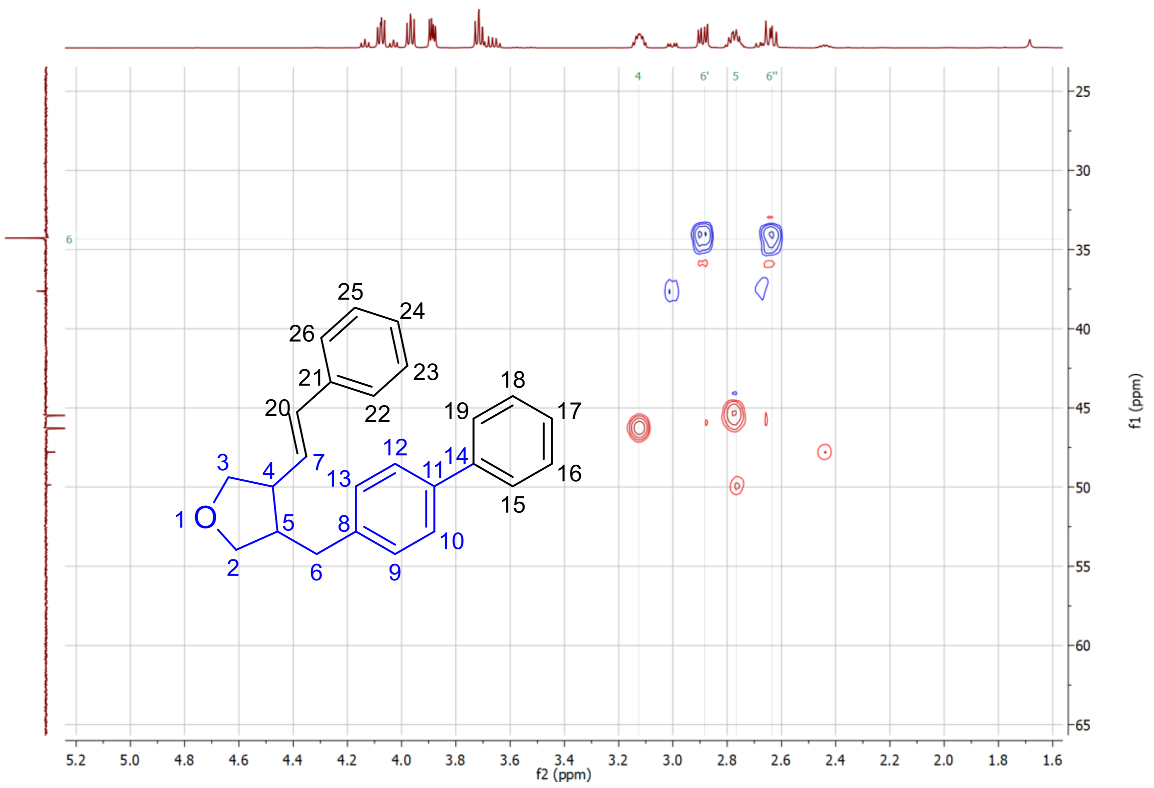
f2 (ppm)
f1 (ppm)
4
6'
5
6''
6
1
2
3
4
5
6
7
8
9
10
11
12
13
14
15
16
17
18
19
20
21
22
23
24
25
26

NOESY

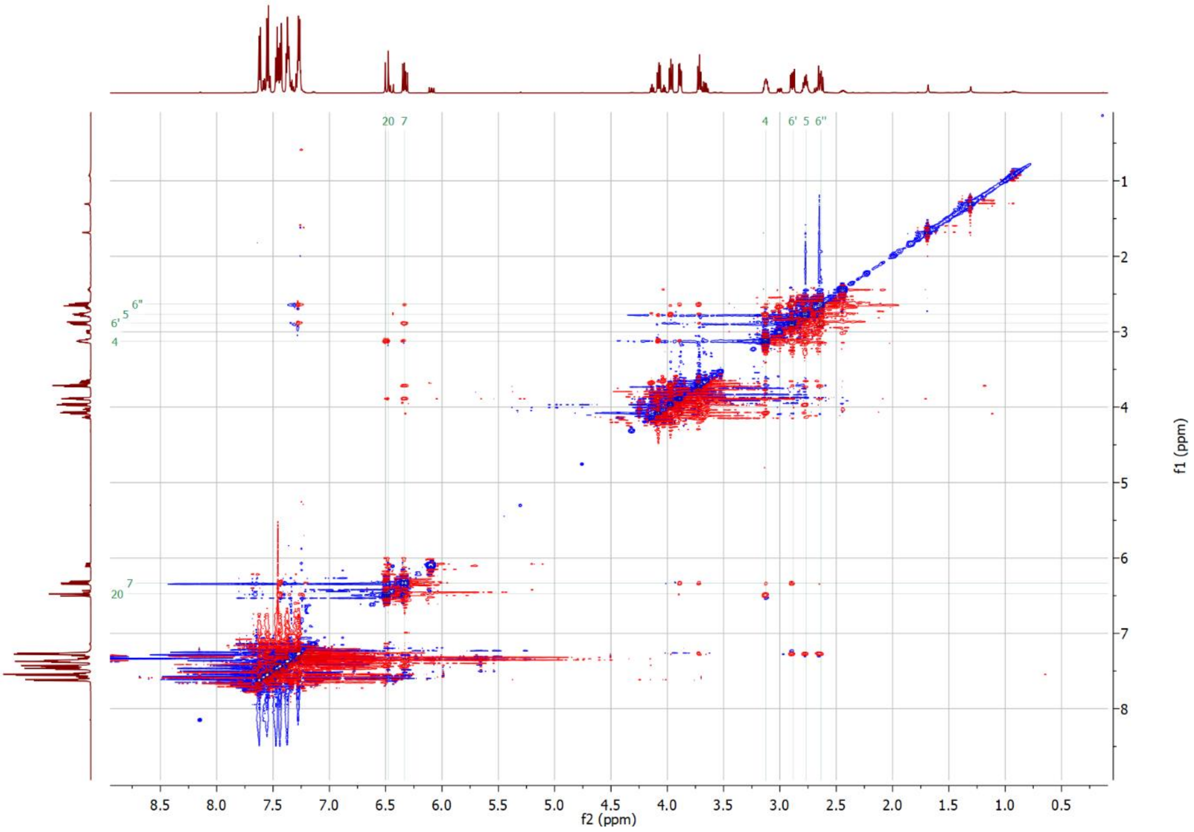

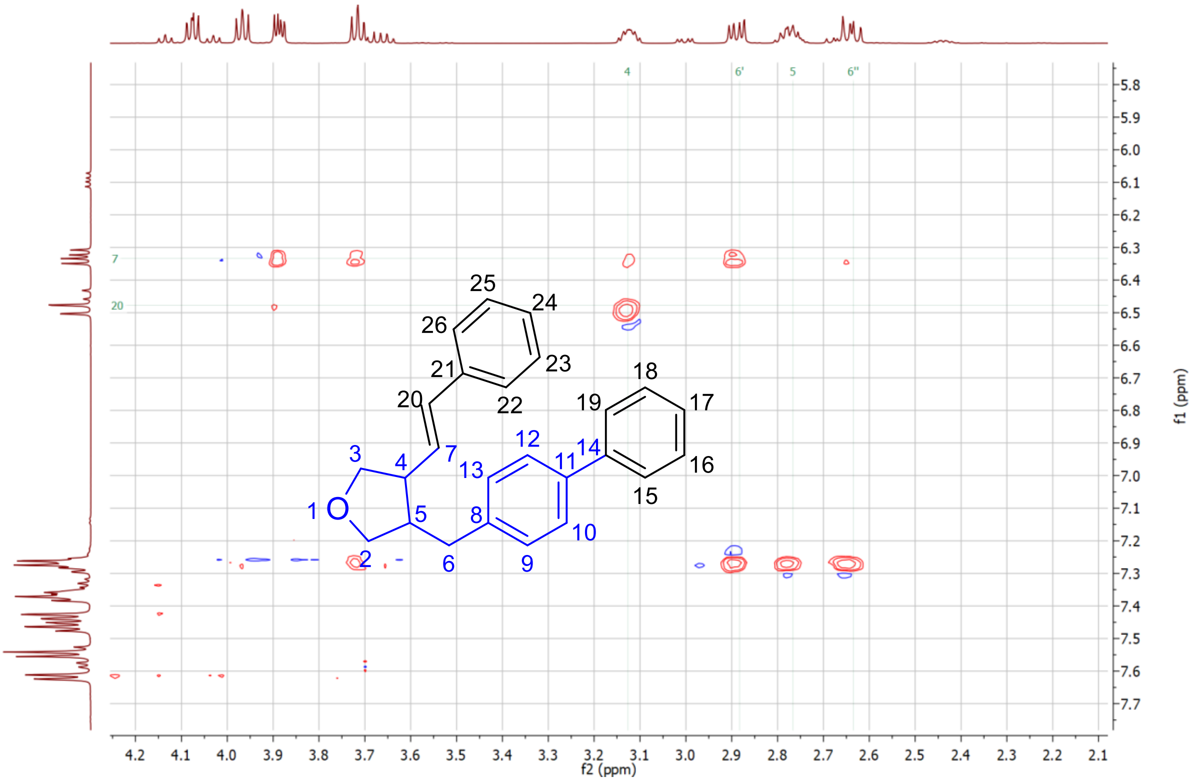

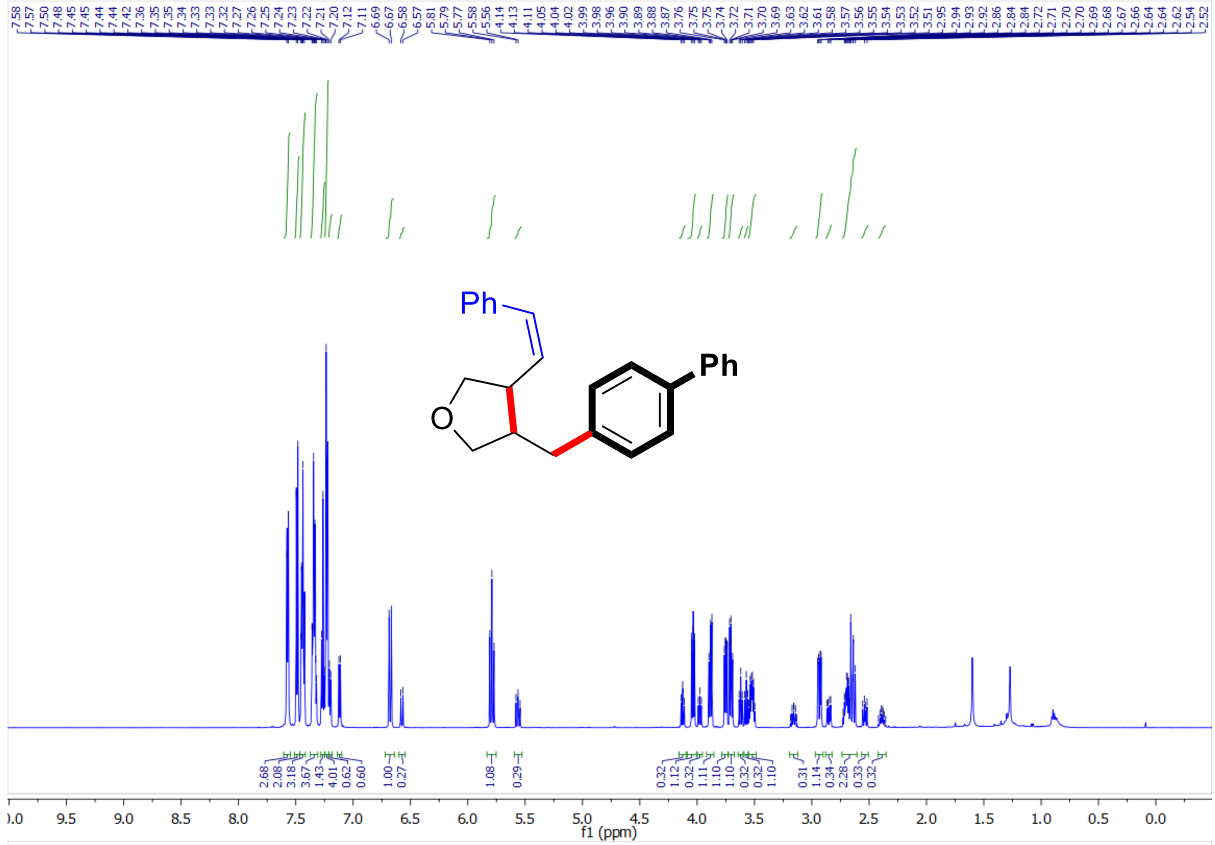

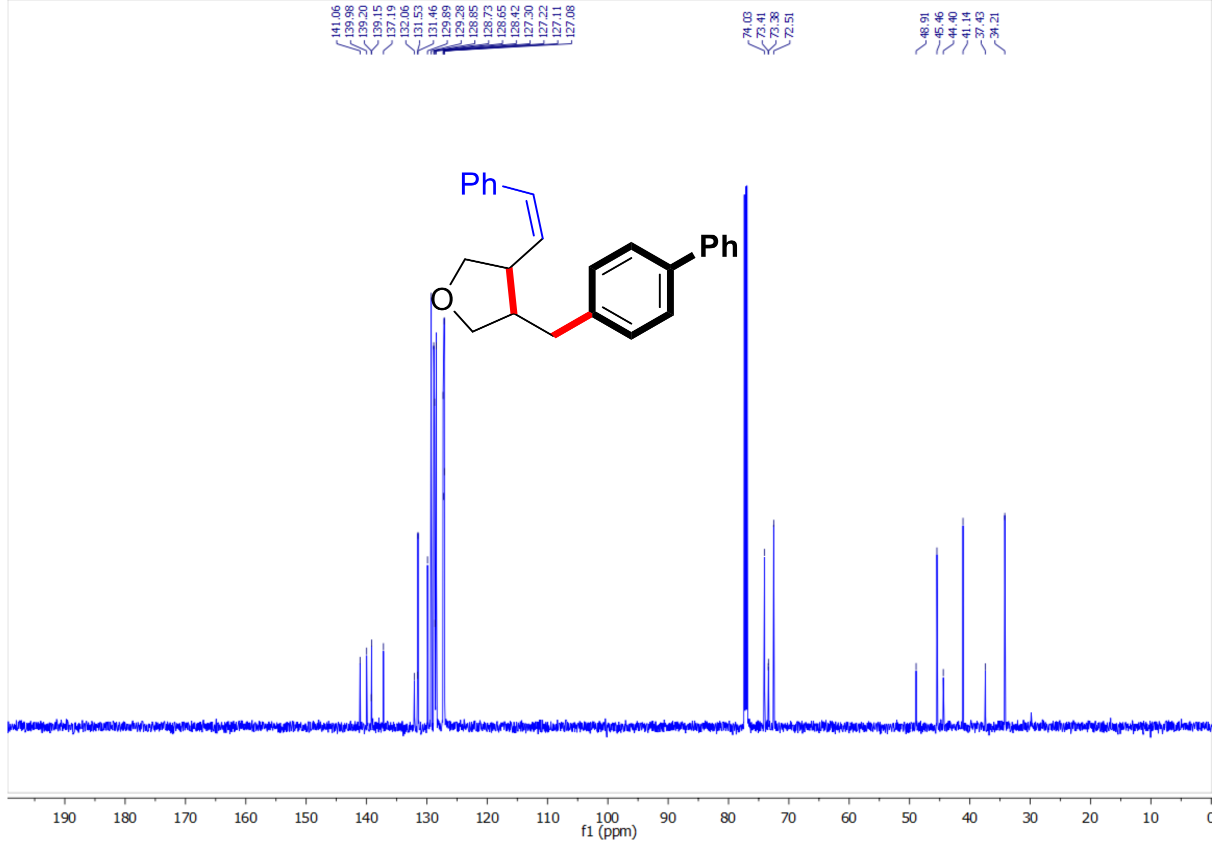

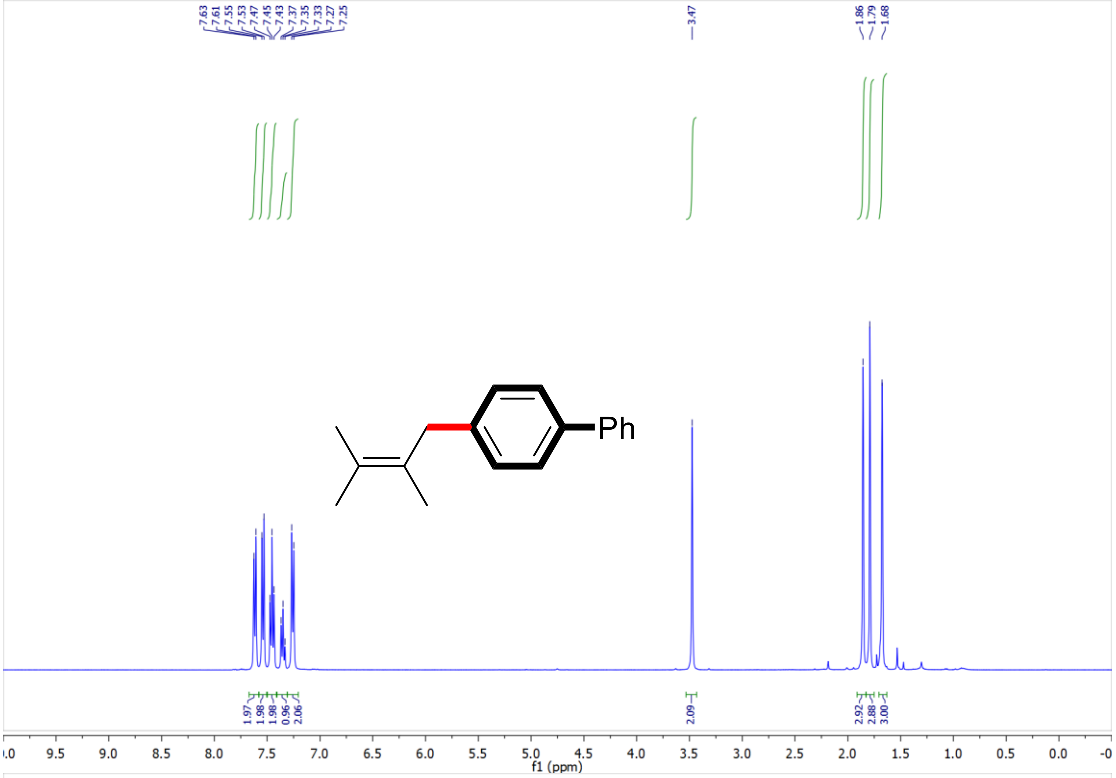
7.63
7.61
7.55
7.53
7.47
7.45
7.43
7.37
7.35
7.33
7.27
7.25
3.47
1.86
1.79
1.68
Ph
1.97
1.98
1.98
0.96
2.06
2.09
2.92
2.88
3.00
f1 (ppm)

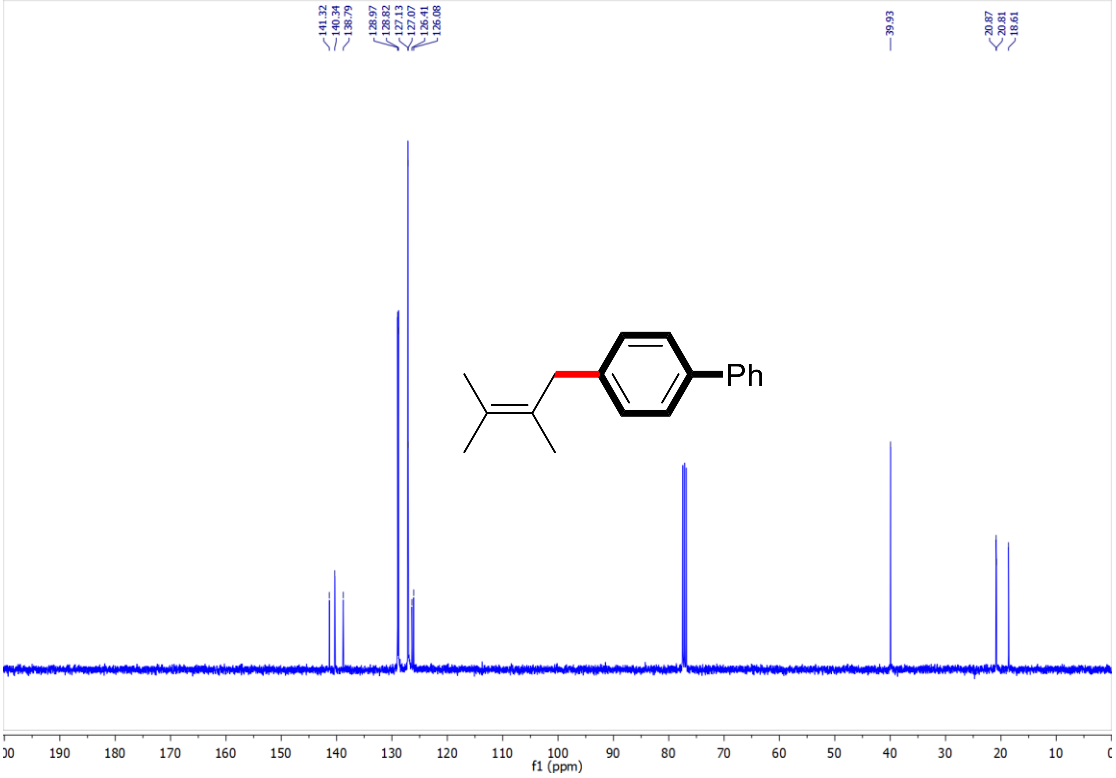
141.32
140.34
138.79
128.97
128.82
127.13
127.07
126.41
126.08
39.93
20.87
20.81
18.61
Ph
f1 (ppm)
